# Supplementary material for: Cross-classified Multilevel Analysis of Individual Heterogeneity and Discriminatory Accuracy (MAIHDA) to evaluate hospital performance: the case of hospital differences in patient survival after acute myocardial infarction
Source: BMJ Open. 2020 Oct 23;10(10):e036130. doi: 10.1136/bmjopen-2019-036130 (PMC7590346; doi:10.1136/bmjopen-2019-036130)
Supplement: Supplementary data [file bmjopen-2019-036130supp001.pdf]

hospital	cons	rscategory	stratum	proportion	numerator	denominator
1	1	1	1	0.00	0	99
1	1	2	2	0.01	1	76
1	1	3	3	0.03	2	73
1	1	4	4	0.02	1	62
1	1	5	5	0.01	1	75
1	1	6	6	0.05	4	87
1	1	7	7	0.07	8	108
1	1	8	8	0.06	7	112
1	1	9	9	0.09	11	118
1	1	10	10	0.22	26	116
2	1	1	11	0.00	0	196
2	1	2	12	0.01	1	179
2	1	3	13	0.01	1	154
2	1	4	14	0.03	4	136
2	1	5	15	0.04	7	158
2	1	6	16	0.04	7	159
2	1	7	17	0.06	9	152
2	1	8	18	0.14	27	189
2	1	9	19	0.18	34	193
2	1	10	20	0.22	42	195
3	1	1	21	0.01	3	249
3	1	2	22	0.02	4	189
3	1	3	23	0.02	3	172
3	1	4	24	0.04	7	186
3	1	5	25	0.07	10	149
3	1	6	26	0.07	11	166
3	1	7	27	0.09	12	129
3	1	8	28	0.16	20	128
3	1	9	29	0.22	21	94
3	1	10	30	0.31	29	95
4	1	1	31	0.01	1	99
4	1	2	32	0.01	1	69
4	1	3	33	0.03	2	76
4	1	4	34	0.04	3	72
4	1	5	35	0.05	4	78
4	1	6	36	0.10	9	88
4	1	7	37	0.08	7	92
4	1	8	38	0.12	10	86
4	1	9	39	0.21	19	91
4	1	10	40	0.20	25	122
5	1	1	41	0.00	0	127
5	1	2	42	0.00	0	97
5	1	3	43	0.02	2	113
5	1	4	44	0.00	0	100
5	1	5	45	0.00	0	93
5	1	6	46	0.05	5	102

5	1	7	47	0.05	6	110
5	1	8	48	0.06	9	145
5	1	9	49	0.07	10	140
5	1	10	50	0.24	35	147
6	1	1	51	0.00	0	26
6	1	2	52	0.00	0	22
6	1	3	53	0.03	1	30
6	1	4	54	0.05	2	37
6	1	5	55	0.09	2	22
6	1	6	56	0.05	2	37
6	1	7	57	0.06	2	32
6	1	8	58	0.13	4	32
6	1	9	59	0.11	3	28
6	1	10	60	0.22	8	37
7	1	1	61	0.00	0	26
7	1	2	62	0.00	0	19
7	1	3	63	0.06	1	18
7	1	4	64	0.00	0	23
7	1	5	65	0.12	3	26
7	1	6	66	0.07	2	27
7	1	7	67	0.00	0	26
7	1	8	68	0.14	5	37
7	1	9	69	0.28	10	36
7	1	10	70	0.22	7	32
8	1	1	71	0.00	0	157
8	1	2	72	0.02	4	167
8	1	3	73	0.01	1	147
8	1	4	74	0.05	6	133
8	1	5	75	0.04	5	135
8	1	6	76	0.07	9	123
8	1	7	77	0.03	5	154
8	1	8	78	0.13	16	126
8	1	9	79	0.09	12	139
8	1	10	80	0.29	39	133
9	1	1	81	0.00	0	27
9	1	2	82	0.00	0	23
9	1	3	83	0.00	0	14
9	1	4	84	0.00	0	7
9	1	5	85	0.07	1	14
9	1	6	86	0.00	0	15
9	1	7	87	0.00	0	7
9	1	8	88	0.08	1	13
9	1	9	89	0.00	0	11
9	1	10	90	0.21	3	14
10	1	1	91	0.00	0	62
10	1	2	92	0.03	3	93
10	1	3	93	0.01	1	84

10	1	4	94	0.01	1	73
10	1	5	95	0.04	3	78
10	1	6	96	0.07	6	90
10	1	7	97	0.07	6	87
10	1	8	98	0.23	14	62
10	1	9	99	0.16	11	69
10	1	10	100	0.34	18	53
11	1	1	101	0.04	1	24
11	1	2	102	0.04	1	28
11	1	3	103	0.03	1	37
11	1	4	104	0.00	0	50
11	1	5	105	0.03	1	37
11	1	6	106	0.07	2	28
11	1	7	107	0.08	3	38
11	1	8	108	0.06	2	31
11	1	9	109	0.04	1	27
11	1	10	110	0.40	6	15
12	1	1	111	0.00	0	10
12	1	2	112	0.00	0	19
12	1	3	113	0.00	0	13
12	1	4	114	0.00	0	28
12	1	5	115	0.00	0	14
12	1	6	116	0.07	2	28
12	1	7	117	0.14	3	21
12	1	8	118	0.00	0	18
12	1	9	119	0.24	4	17
12	1	10	120	0.14	2	14
13	1	1	121	0.00	0	135
13	1	2	122	0.01	1	137
13	1	3	123	0.04	5	141
13	1	4	124	0.02	2	133
13	1	5	125	0.01	2	136
13	1	6	126	0.04	5	134
13	1	7	127	0.10	12	125
13	1	8	128	0.09	8	94
13	1	9	129	0.14	14	101
13	1	10	130	0.24	21	88
14	1	1	131	0.02	1	45
14	1	2	132	0.00	0	44
14	1	3	133	0.00	0	42
14	1	4	134	0.03	2	68
14	1	5	135	0.04	3	68
14	1	6	136	0.06	3	52
14	1	7	137	0.07	4	58
14	1	8	138	0.16	10	62
14	1	9	139	0.20	12	61
14	1	10	140	0.30	24	80

15	1	1	141	0.03	1	32
15	1	2	142	0.00	0	17
15	1	3	143	0.00	0	35
15	1	4	144	0.08	2	25
15	1	5	145	0.11	3	28
15	1	6	146	0.05	1	19
15	1	7	147	0.04	1	25
15	1	8	148	0.17	4	23
15	1	9	149	0.23	5	22
15	1	10	150	0.17	4	24
16	1	1	151	0.00	0	74
16	1	2	152	0.01	1	94
16	1	3	153	0.00	0	85
16	1	4	154	0.00	0	100
16	1	5	155	0.03	3	91
16	1	6	156	0.12	10	82
16	1	7	157	0.06	6	93
16	1	8	158	0.10	9	86
16	1	9	159	0.16	10	64
16	1	10	160	0.20	16	80
17	1	1	161	0.00	0	18
17	1	2	162	0.03	1	35
17	1	3	163	0.00	0	28
17	1	4	164	0.06	2	34
17	1	5	165	0.07	3	41
17	1	6	166	0.11	4	38
17	1	7	167	0.05	2	39
17	1	8	168	0.09	4	43
17	1	9	169	0.14	4	29
17	1	10	170	0.36	12	33
18	1	1	171	0.07	2	27
18	1	2	172	0.04	1	23
18	1	3	173	0.00	0	24
18	1	4	174	0.00	0	26
18	1	5	175	0.00	0	30
18	1	6	176	0.08	2	26
18	1	7	177	0.07	2	27
18	1	8	178	0.06	2	34
18	1	9	179	0.08	3	39
18	1	10	180	0.08	3	40
19	1	1	181	0.00	0	34
19	1	2	182	0.00	0	40
19	1	3	183	0.00	0	38
19	1	4	184	0.08	3	39
19	1	5	185	0.00	0	45
19	1	6	186	0.00	0	37
19	1	7	187	0.08	4	53

19	1	8	188	0.11	6	56
19	1	9	189	0.15	9	60
19	1	10	190	0.24	13	54
20	1	1	191	0.00	0	20
20	1	2	192	0.06	1	18
20	1	3	193	0.00	0	13
20	1	4	194	0.05	1	20
20	1	5	195	0.05	1	20
20	1	6	196	0.04	1	23
20	1	7	197	0.04	1	25
20	1	8	198	0.16	4	25
20	1	9	199	0.20	4	20
20	1	10	200	0.24	4	17
21	1	1	201	0.00	0	21
21	1	2	202	0.00	0	20
21	1	3	203	0.00	0	18
21	1	4	204	0.00	0	25
21	1	5	205	0.07	2	29
21	1	6	206	0.07	2	30
21	1	7	207	0.14	3	22
21	1	8	208	0.11	3	28
21	1	9	209	0.28	8	29
21	1	10	210	0.27	7	26
22	1	1	211	0.00	0	85
22	1	2	212	0.01	1	107
22	1	3	213	0.03	4	117
22	1	4	214	0.02	3	122
22	1	5	215	0.04	4	111
22	1	6	216	0.04	4	98
22	1	7	217	0.14	13	94
22	1	8	218	0.11	11	100
22	1	9	219	0.14	13	91
22	1	10	220	0.21	17	81
23	1	1	221	0.06	1	17
23	1	2	222	0.05	1	22
23	1	3	223	0.00	0	16
23	1	4	224	0.04	1	28
23	1	5	225	0.00	0	22
23	1	6	226	0.08	2	26
23	1	7	227	0.16	6	38
23	1	8	228	0.00	0	23
23	1	9	229	0.15	4	26
23	1	10	230	0.13	3	24
24	1	1	231	0.00	0	27
24	1	2	232	0.08	3	38
24	1	3	233	0.00	0	34
24	1	4	234	0.00	0	28

24	1	5	235	0.03	1	33
24	1	6	236	0.12	5	43
24	1	7	237	0.05	2	37
24	1	8	238	0.13	5	39
24	1	9	239	0.17	5	29
24	1	10	240	0.24	7	29
25	1	1	241	0.01	1	101
25	1	2	242	0.02	2	100
25	1	3	243	0.03	3	94
25	1	4	244	0.03	3	113
25	1	5	245	0.07	8	109
25	1	6	246	0.09	10	113
25	1	7	247	0.10	10	104
25	1	8	248	0.16	14	89
25	1	9	249	0.21	18	85
25	1	10	250	0.20	15	76
26	1	1	251	0.00	0	41
26	1	2	252	0.00	0	43
26	1	3	253	0.00	0	48
26	1	4	254	0.00	0	47
26	1	5	255	0.04	2	56
26	1	6	256	0.02	1	44
26	1	7	257	0.04	2	53
26	1	8	258	0.09	5	57
26	1	9	259	0.10	7	68
26	1	10	260	0.32	25	79
27	1	1	261	0.00	0	9
27	1	2	262	0.00	0	17
27	1	3	263	0.00	0	17
27	1	4	264	0.05	1	21
27	1	5	265	0.00	0	18
27	1	6	266	0.00	0	20
27	1	7	267	0.07	1	14
27	1	8	268	0.00	0	26
27	1	9	269	0.14	5	35
27	1	10	270	0.29	11	38
28	1	1	271	0.06	1	16
28	1	2	272	0.00	0	20
28	1	3	273	0.00	0	17
28	1	4	274	0.00	0	19
28	1	5	275	0.00	0	33
28	1	6	276	0.06	1	16
28	1	7	277	0.12	2	17
28	1	8	278	0.09	2	23
28	1	9	279	0.21	7	34
28	1	10	280	0.11	5	46
29	1	1	281	0.00	0	145

29	1	2	282	0.01	1	159
29	1	3	283	0.02	2	133
29	1	4	284	0.03	3	114
29	1	5	285	0.05	6	130
29	1	6	286	0.05	7	142
29	1	7	287	0.09	13	145
29	1	8	288	0.11	17	151
29	1	9	289	0.11	17	161
29	1	10	290	0.26	44	170
30	1	1	291	0.00	1	245
30	1	2	292	0.02	5	222
30	1	3	293	0.03	7	223
30	1	4	294	0.02	5	218
30	1	5	295	0.03	5	200
30	1	6	296	0.07	16	227
30	1	7	297	0.08	13	166
30	1	8	298	0.16	27	170
30	1	9	299	0.16	33	201
30	1	10	300	0.26	44	169
31	1	1	301	0.00	0	30
31	1	2	302	0.06	1	17
31	1	3	303	0.00	0	23
31	1	4	304	0.00	0	13
31	1	5	305	0.00	0	15
31	1	6	306	0.00	0	16
31	1	7	307	0.08	1	12
31	1	8	308	0.17	4	24
31	1	9	309	0.13	2	16
31	1	10	310	0.40	10	25
32	1	1	311	0.01	1	95
32	1	2	312	0.00	0	86
32	1	3	313	0.01	1	74
32	1	4	314	0.03	2	61
32	1	5	315	0.03	2	65
32	1	6	316	0.08	6	79
32	1	7	317	0.04	3	71
32	1	8	318	0.13	10	78
32	1	9	319	0.14	13	90
32	1	10	320	0.31	26	83
33	1	1	321	0.00	0	39
33	1	2	322	0.00	0	19
33	1	3	323	0.00	0	38
33	1	4	324	0.00	0	39
33	1	5	325	0.02	1	53
33	1	6	326	0.02	1	42
33	1	7	327	0.11	5	46
33	1	8	328	0.09	5	57

33	1	9	329	0.16	11	70
33	1	10	330	0.23	19	82
34	1	1	331	0.02	1	61
34	1	2	332	0.02	1	49
34	1	3	333	0.00	0	35
34	1	4	334	0.04	2	57
34	1	5	335	0.02	1	52
34	1	6	336	0.05	3	60
34	1	7	337	0.09	7	75
34	1	8	338	0.12	10	84
34	1	9	339	0.13	12	92
34	1	10	340	0.28	33	117
35	1	1	341	0.00	0	26
35	1	2	342	0.03	1	36
35	1	3	343	0.08	3	38
35	1	4	344	0.00	0	34
35	1	5	345	0.03	1	29
35	1	6	346	0.08	3	39
35	1	7	347	0.10	4	42
35	1	8	348	0.09	4	45
35	1	9	349	0.24	9	38
35	1	10	350	0.33	17	51
36	1	1	351	0.01	2	331
36	1	2	352	0.01	3	316
36	1	3	353	0.02	6	319
36	1	4	354	0.03	8	305
36	1	5	355	0.03	9	321
36	1	6	356	0.06	17	306
36	1	7	357	0.08	27	319
36	1	8	358	0.13	40	303
36	1	9	359	0.21	54	256
36	1	10	360	0.26	68	261
37	1	1	361	0.04	1	26
37	1	2	362	0.00	0	30
37	1	3	363	0.03	1	36
37	1	4	364	0.00	0	35
37	1	5	365	0.02	1	43
37	1	6	366	0.07	2	30
37	1	7	367	0.05	2	39
37	1	8	368	0.14	5	37
37	1	9	369	0.13	6	47
37	1	10	370	0.25	14	56
38	1	1	371	0.00	0	96
38	1	2	372	0.02	2	88
38	1	3	373	0.02	2	97
38	1	4	374	0.02	2	96
38	1	5	375	0.07	8	113

38	1	6	376	0.07	7	107
38	1	7	377	0.10	12	119
38	1	8	378	0.16	20	124
38	1	9	379	0.18	27	151
38	1	10	380	0.24	36	148
39	1	1	381	0.01	1	78
39	1	2	382	0.02	2	87
39	1	3	383	0.01	1	80
39	1	4	384	0.04	3	73
39	1	5	385	0.03	3	89
39	1	6	386	0.03	3	89
39	1	7	387	0.13	8	64
39	1	8	388	0.15	15	98
39	1	9	389	0.16	14	88
39	1	10	390	0.32	24	75
40	1	1	391	0.00	0	12
40	1	2	392	0.00	0	18
40	1	3	393	0.00	0	12
40	1	4	394	0.00	0	15
40	1	5	395	0.03	1	32
40	1	6	396	0.06	1	18
40	1	7	397	0.00	0	18
40	1	8	398	0.24	5	21
40	1	9	399	0.32	7	22
40	1	10	400	0.39	11	28
41	1	1	401	0.01	1	92
41	1	2	402	0.02	2	128
41	1	3	403	0.03	3	114
41	1	4	404	0.01	1	141
41	1	5	405	0.06	7	124
41	1	6	406	0.11	14	131
41	1	7	407	0.10	14	138
41	1	8	408	0.11	16	149
41	1	9	409	0.15	23	151
41	1	10	410	0.21	24	117
42	1	1	411	0.00	0	101
42	1	2	412	0.00	0	108
42	1	3	413	0.01	2	146
42	1	4	414	0.05	6	131
42	1	5	415	0.07	9	130
42	1	6	416	0.05	6	123
42	1	7	417	0.13	14	109
42	1	8	418	0.07	5	71
42	1	9	419	0.14	12	85
42	1	10	420	0.25	17	69
43	1	1	421	0.00	0	17
43	1	2	422	0.00	0	26

43	1	3	423	0.00	0	24
43	1	4	424	0.07	1	14
43	1	5	425	0.00	0	23
43	1	6	426	0.00	0	17
43	1	7	427	0.04	1	23
43	1	8	428	0.30	6	20
43	1	9	429	0.15	3	20
43	1	10	430	0.23	11	48
44	1	1	431	0.00	0	11
44	1	2	432	0.00	0	18
44	1	3	433	0.00	0	14
44	1	4	434	0.00	0	7
44	1	5	435	0.00	0	14
44	1	6	436	0.00	0	17
44	1	7	437	0.14	3	21
44	1	8	438	0.22	4	18
44	1	9	439	0.39	9	23
44	1	10	440	0.27	6	22
45	1	1	441	0.01	2	137
45	1	2	442	0.02	3	183
45	1	3	443	0.04	5	141
45	1	4	444	0.03	5	153
45	1	5	445	0.03	4	135
45	1	6	446	0.06	7	124
45	1	7	447	0.09	11	117
45	1	8	448	0.06	7	117
45	1	9	449	0.16	12	76
45	1	10	450	0.28	23	81
46	1	1	451	0.00	0	16
46	1	2	452	0.00	0	20
46	1	3	453	0.05	1	19
46	1	4	454	0.07	1	14
46	1	5	455	0.11	3	28
46	1	6	456	0.12	3	26
46	1	7	457	0.22	6	27
46	1	8	458	0.15	5	33
46	1	9	459	0.24	7	29
46	1	10	460	0.43	18	42
47	1	1	461	0.00	0	8
47	1	2	462	0.06	1	16
47	1	3	463	0.10	2	20
47	1	4	464	0.06	1	17
47	1	5	465	0.00	0	20
47	1	6	466	0.16	3	19
47	1	7	467	0.03	1	31
47	1	8	468	0.11	2	19
47	1	9	469	0.29	7	24

47	1	10	470	0.27	8	30
48	1	1	471	0.03	2	71
48	1	2	472	0.00	0	71
48	1	3	473	0.00	0	63
48	1	4	474	0.01	1	69
48	1	5	475	0.02	1	58
48	1	6	476	0.06	4	72
48	1	7	477	0.08	6	72
48	1	8	478	0.16	15	93
48	1	9	479	0.13	11	84
48	1	10	480	0.22	15	67
49	1	1	481	0.00	0	24
49	1	2	482	0.00	0	16
49	1	3	483	0.00	0	30
49	1	4	484	0.00	0	35
49	1	5	485	0.02	1	43
49	1	6	486	0.00	0	43
49	1	7	487	0.08	4	48
49	1	8	488	0.12	5	43
49	1	9	489	0.20	7	35
49	1	10	490	0.18	6	34
50	1	1	491	0.00	0	129
50	1	2	492	0.03	3	108
50	1	3	493	0.01	2	136
50	1	4	494	0.01	1	122
50	1	5	495	0.02	2	98
50	1	6	496	0.05	5	99
50	1	7	497	0.06	7	113
50	1	8	498	0.14	10	72
50	1	9	499	0.12	12	98
50	1	10	500	0.30	21	69
51	1	1	501	0.00	0	21
51	1	2	502	0.00	0	25
51	1	3	503	0.00	0	35
51	1	4	504	0.04	1	25
51	1	5	505	0.06	2	36
51	1	6	506	0.12	5	43
51	1	7	507	0.04	1	27
51	1	8	508	0.14	3	21
51	1	9	509	0.18	8	45
51	1	10	510	0.19	8	42
52	1	1	511	0.00	0	3
52	1	2	512	0.00	0	9
52	1	3	513	0.00	0	9
52	1	4	514	0.00	0	17
52	1	5	515	0.00	0	15
52	1	6	516	0.00	0	12

52	1	7	517	0.22	2	9
52	1	8	518	0.08	1	12
52	1	9	519	0.00	0	10
52	1	10	520	0.18	2	11
53	1	1	521	0.00	0	7
53	1	2	522	0.00	0	12
53	1	3	523	0.00	0	18
53	1	4	524	0.08	1	13
53	1	5	525	0.00	0	13
53	1	6	526	0.00	0	23
53	1	7	527	0.08	1	12
53	1	8	528	0.07	1	14
53	1	9	529	0.14	3	21
53	1	10	530	0.07	1	14
54	1	1	531	0.03	3	97
54	1	2	532	0.02	2	122
54	1	3	533	0.03	4	117
54	1	4	534	0.02	2	119
54	1	5	535	0.03	3	107
54	1	6	536	0.03	4	122
54	1	7	537	0.10	12	119
54	1	8	538	0.11	11	101
54	1	9	539	0.19	15	81
54	1	10	540	0.32	34	106
55	1	1	541	0.00	0	27
55	1	2	542	0.00	0	53
55	1	3	543	0.00	0	30
55	1	4	544	0.00	0	38
55	1	5	545	0.00	0	37
55	1	6	546	0.00	0	38
55	1	7	547	0.08	3	39
55	1	8	548	0.10	3	29
55	1	9	549	0.13	4	31
55	1	10	550	0.19	7	37
56	1	1	551	0.02	1	47
56	1	2	552	0.00	0	38
56	1	3	553	0.04	2	51
56	1	4	554	0.02	1	51
56	1	5	555	0.00	0	37
56	1	6	556	0.06	3	47
56	1	7	557	0.06	3	47
56	1	8	558	0.14	6	42
56	1	9	559	0.18	8	44
56	1	10	560	0.22	7	32
57	1	1	561	0.01	1	69
57	1	2	562	0.00	0	73
57	1	3	563	0.01	1	87

57	1	4	564	0.03	3	90
57	1	5	565	0.05	4	88
57	1	6	566	0.05	4	74
57	1	7	567	0.08	7	83
57	1	8	568	0.14	11	76
57	1	9	569	0.18	12	68
57	1	10	570	0.31	25	81
58	1	1	571	0.00	0	22
58	1	2	572	0.04	1	23
58	1	3	573	0.00	0	42
58	1	4	574	0.06	2	35
58	1	5	575	0.00	0	34
58	1	6	576	0.06	2	33
58	1	7	577	0.00	0	34
58	1	8	578	0.17	7	42
58	1	9	579	0.11	3	28
58	1	10	580	0.20	6	30
59	1	1	581	0.00	0	12
59	1	2	582	0.04	1	27
59	1	3	583	0.04	1	28
59	1	4	584	0.04	1	23
59	1	5	585	0.04	1	26
59	1	6	586	0.13	2	15
59	1	7	587	0.05	1	19
59	1	8	588	0.00	0	30
59	1	9	589	0.22	4	18
59	1	10	590	0.30	7	23
60	1	1	591	0.02	1	66
60	1	2	592	0.02	1	63
60	1	3	593	0.04	3	71
60	1	4	594	0.00	0	80
60	1	5	595	0.02	1	62
60	1	6	596	0.05	3	59
60	1	7	597	0.11	6	53
60	1	8	598	0.07	3	46
60	1	9	599	0.18	8	44
60	1	10	600	0.16	7	43
61	1	1	601	0.01	1	150
61	1	2	602	0.02	2	120
61	1	3	603	0.03	4	119
61	1	4	604	0.04	4	111
61	1	5	605	0.02	2	91
61	1	6	606	0.04	3	83
61	1	7	607	0.11	10	90
61	1	8	608	0.17	13	76
61	1	9	609	0.12	12	97
61	1	10	610	0.29	23	78

62	1	1	611	0.00	0	31
62	1	2	612	0.04	1	25
62	1	3	613	0.00	0	31
62	1	4	614	0.00	0	35
62	1	5	615	0.06	2	36
62	1	6	616	0.06	2	34
62	1	7	617	0.11	3	28
62	1	8	618	0.08	3	37
62	1	9	619	0.13	6	48
62	1	10	620	0.20	7	35
63	1	1	621	0.00	0	17
63	1	2	622	0.00	0	18
63	1	3	623	0.10	2	21
63	1	4	624	0.04	1	23
63	1	5	625	0.04	1	26
63	1	6	626	0.00	0	20
63	1	7	627	0.05	1	21
63	1	8	628	0.18	3	17
63	1	9	629	0.11	2	18
63	1	10	630	0.18	3	17
64	1	1	631	0.00	0	31
64	1	2	632	0.00	0	25
64	1	3	633	0.13	4	32
64	1	4	634	0.15	3	20
64	1	5	635	0.19	6	31
64	1	6	636	0.07	2	27
64	1	7	637	0.16	4	25
64	1	8	638	0.26	6	23
64	1	9	639	0.35	9	26
64	1	10	640	0.47	7	15
65	1	1	641	0.00	0	32
65	1	2	642	0.00	0	22
65	1	3	643	0.00	0	24
65	1	4	644	0.00	0	28
65	1	5	645	0.00	0	32
65	1	6	646	0.05	2	44
65	1	7	647	0.00	0	29
65	1	8	648	0.12	3	25
65	1	9	649	0.24	7	29
65	1	10	650	0.14	3	22
66	1	1	651	0.00	0	18
66	1	2	652	0.00	0	30
66	1	3	653	0.00	0	16
66	1	4	654	0.10	2	21
66	1	5	655	0.00	0	28
66	1	6	656	0.05	1	20
66	1	7	657	0.04	1	25

66	1	8	658	0.16	5	31
66	1	9	659	0.24	7	29
66	1	10	660	0.32	10	31
67	1	1	661	0.00	0	17
67	1	2	662	0.00	0	12
67	1	3	663	0.05	1	21
67	1	4	664	0.00	0	16
67	1	5	665	0.00	0	11
67	1	6	666	0.17	2	12
67	1	7	667	0.00	0	15
67	1	8	668	0.11	2	19
67	1	9	669	0.21	4	19
67	1	10	670	0.38	8	21
68	1	1	671	0.00	0	70
68	1	2	672	0.00	0	48
68	1	3	673	0.00	0	57
68	1	4	674	0.03	2	61
68	1	5	675	0.04	2	51
68	1	6	676	0.07	3	45
68	1	7	677	0.13	7	55
68	1	8	678	0.14	9	66
68	1	9	679	0.20	11	56
68	1	10	680	0.31	17	54
