# Supplementary material for: Cross-classified Multilevel Analysis of Individual Heterogeneity and Discriminatory Accuracy (MAIHDA) to evaluate hospital performance: the case of hospital differences in patient survival after acute myocardial infarction
Source: BMJ Open. 2020 Oct 23;10(10):e036130. doi: 10.1136/bmjopen-2019-036130 (PMC7590346; doi:10.1136/bmjopen-2019-036130)
Supplement: Supplementary data [file bmjopen-2019-036130supp002.pdf]

```
*****************************************************************************
* A Cross-Classified Multilevel Analysis of Individual Heterogeneity and
* Discriminatory Accuracy (MAIHDA) to evaluate hospital performance: The case of
* Hospital Differences in Patient Survival after Acute Myocardial Infarction
*****************************************************************************

* Set the working directory
cd "Y:\manuscripts-4-under-review\rodriguez-lopez2020bmiopen\2020-07-16"
// Edit the above folder directory to where the dataset is saved

* Set MLwiN path
global MLwiN_path "C:\Program Files\MLwiN v3.05\mlwin.exe"

* Set the output format of coefficient tables to two decimal places
set cformat %9.2f



*****************************************************************************
* Model 1
*****************************************************************************

* Load the data
use "Supplemental Material 1_AMIdatabase.dta", clear

* Fit model 1 by MQL1 to get parameter starting values
runmlwin proportion cons, ///
  level4(hospital: cons) ///
  level3(rscategory:) ///
```

```
  level2(stratum:) ///
  level1(stratum:) ///
  discrete(distribution(binomial) link(logit) denominator(denom) mql1) ///
  nopause

* Fit model 1 by MCMC to get final parameter estimates
runmlwin proportion cons, ///
  level4(hospital: cons, residuals(v, savechains("m1v.dta", replace))) ///
  level3(rscategory:) ///
  level2(stratum:) ///
  level1(stratum:) ///
  discrete(distribution(binomial) link(logit) denominator(denom)) ///
  mcmc(burnin(5000) chain(10000) thinning(10)  savechains("m1b.dta", replace)) ///
  initsprevious ///
  nopause

* Model predictions based on means of MCMC chains
rename v0* m1v*
predict m1xb, xb
generate m1xbv = m1xb + m1v
generate m1pxbv = invlogit(m1xbv)

* Compress and save the data
compress
save "m1.dta", replace

* VPC based on full MCMC chains
use "m1b.dta", clear
rename RP4_var_cons_ sigma2v
```

```
generate m1vpc_v = 100*sigma2v/(sigma2v + _pi^2/3)

mcmcsum m1vpc_v, variable


* Model predictions based on full MCMC chains

use "m1b.dta", clear

isid iteration

keep iteration FP1_cons

rename FP1_cons beta0

merge 1:m iteration using "m1v.dta", assert(match) keep(match) keepusing(hospital value)
nogenerate

rename value v

generate m1pxbv = 100*invlogit(beta + v)

gcollapse (mean) m1pxbvmn = m1pxbv (p2.5) m1pxbvlo = m1pxbv (p97.5) m1pxbvhi = m1pxbv,
by(hospital)

compress

save "m1pxbv", replace


* Residuals H - Histogram of the predicted hospital random effects based on means of MCMC
chains

use "m1.dta", clear

egen tag_hospital = tag(hospital)

histogram m1v if tag_hospital==1, width(0.1) start(-0.35) frequency addlabel ///

  normal ///

  kdensity kdenopts(lpattern(dash)) ///

  ytitle("Number of hospitals") ///

  ylabel(, angle(horizontal)) ///

  xtitle("Predicted hospital random effect") ///

  legend(on order(3 "Normal curve" 2 "Smoothed histogram") position(2) ring(0)) ///

  scheme(s1mono) ///

  xsize(7.25) ysize(4)
```

```
graph export "Figure S1 - Model 1 - Histogram of the predicted hospital random effects.png",
replace width(1000)

* ROC AUC based on means of MCMC chains
use "m1.dta", clear
keep hospital rscategory stratum numerator denominator m1xbv
expand denominator
sort stratum
generate patient = _n
order patient, after(stratum)
bysort stratum (patient): generate y = (numerator>=_n)
generate m1pxbv = invlogit(m1xbv)
roctab y m1pxbv
compress
save "m1roc.dta", replace



*****************************************************************************
* Model 2
*****************************************************************************

* Load the data
use "Supplemental Material 1_AMIdatabase.dta", clear

* Fit model 2 by MQL1 to get parameter starting values
runmlwin prop cons, ///
  level4(hospital: cons) ///
  level3(rscategory: cons) ///
```

```
  level2(stratum:) ///
  level1(stratum:) ///
  discrete(distribution(binomial) link(logit) denominator(denom)) ///
  nopause

* Fit model 2 by MCMC to get final parameter estimates
runmlwin prop cons, ///
  level4(hospital: cons, residuals(v, savechains("m2v.dta", replace))) ///
  level3(rscategory: cons, residuals(u, savechains("m2u.dta", replace))) ///
  level2(stratum:) ///
  level1(stratum:) ///
  discrete(distribution(binomial) link(logit) denominator(denom)) ///
  mcmc(cc burnin(5000) chain(10000) thinning(10) hc(3) savechains("m2b.dta", replace)) ///
  initsprevious nopause
rename v0* m2v*
rename u0* m2u*

* Model predictions based on means of MCMC chains
predict m2xb, xb
generate m2xbv = m2xb + m2v
generate m2xbu = m2xb + m2u
generate m2xbvu = m2xb + m2v + m2u
generate m2pxbv = invlogit(m2xbv)
generate m2pxbu = invlogit(m2xbu)
generate m2pxbvu = invlogit(m2xbvu)
generate m2pxbvlo = invlogit(m2xbv - 1.96*m2vse)
generate m2pxbvhi = invlogit(m2xbv + 1.96*m2vse)

* Compress and save the data
```

```
compress

save "m2.dta", replace


* Model predictions based on full MCMC chains

use "m2b.dta", clear

isid iteration

keep iteration FP1_cons

rename FP1_cons beta0

merge 1:m iteration using "m2v.dta", assert(match) keep(match) keepusing(hospital value)
nogenerate

rename value v

generate m2pxbv = 100*invlogit(beta + v)

gcollapse (mean) m2pxbvmn = m2pxbv (p2.5) m2pxbvlo = m2pxbv (p97.5) m2pxbvhi = m2pxbv,
by(hospital)

compress

save "m2pxbv", replace


* VPC based on full MCMC chains

use "m2b.dta", clear

rename RP4_var_cons_ sigma2v

rename RP3_var_cons_ sigma2u

generate m1vpc_v = 100*sigma2v/(sigma2v + sigma2u + _pi^2/3)

generate m1vpc_u = 100*sigma2u/(sigma2v + sigma2u + _pi^2/3)

mcmcsum m1vpc_v m1vpc_u, variable


* Residuals H - Histogram of the predicted hospital random effects based on means of MCMC
chains

use "m2.dta", clear

egen tag_hospital = tag(hospital)

histogram m2v if tag_hospital==1, width(0.1) start(-0.40) frequency addlabel ///
```

```
  normal ///
  kdensity kdenopts(lpattern(dash)) ///
  ytitle("Number of hospitals") ///
  ylabel(, angle(horizontal)) ///
  xtitle("Predicted hospital random effect") ///
  legend(on order(3 "Normal curve" 2 "Smoothed histogram") position(2) ring(0)) ///
  scheme(s1mono) ///
  xsize(7.25) ysize(4)
graph export "Figure S2 - Model 2 - Histogram of the predicted hospital random effects.png", replace width(1000)

* Residuals RS - Histogram of the predicted hospital random effects based on means of MCMC chains
use "m2.dta", clear
egen tag_rscategory = tag(rscategory)
histogram m2u if tag_rscategory==1, width(1) start(-3) frequency addlabel ///
  normal ///
  kdensity kdenopts(lpattern(dash)) ///
  ytitle("Number of RS categories") ///
  ylabel(, angle(horizontal)) ///
  xtitle("Predicted RS category random effect") ///
  legend(on order(3 "Normal curve" 2 "Smoothed histogram") position(2) ring(0)) ///
  scheme(s1mono) ///
  xsize(7.25) ysize(4)
graph export "Figure S3 - Model 2 - Histogram of the predicted RS category random effects.png", replace width(1000)

* Figure 2 based on full MCMC chains
use "m1.dta", replace
collapse (sum) numerator denominator, by(hospital)
```

```
isid hospital
generate percentage = 100*(numerator/denominator)
merge 1:m hospital using "m1pxbv.dta", assert(match) keep(match) nogenerate
merge 1:m hospital using "m2pxbv.dta", assert(match) keep(match) nogenerate
sort m2pxbvmn
generate rank = _n
twoway ///
  (scatter percentage rank, msymbol(circle) mfcolor(white) mlcolor(black)) ///
  (scatter m1pxbvmn rank, mcolor(gray) msymbol(circle)) ///
  (scatter m2pxbvmn rank, mcolor(black) msymbol(circle)) ///
  (rspike m2pxbvhi m2pxbvlo rank), ///
  ytitle("Absolute risk of 30-day mortality (%)") ///
  ylabel(, angle(horizontal)) ///
  xtitle("Hospital rank") ///
  xlabel(0(10)70) ///
  legend(off) ///
  scheme(s1mono) ///
  xsize(7.25) ysize(4)
graph export "Figure 2.png", replace width(1000)

* ROC AUC based on means of MCMC chains
use "m2.dta", clear
keep hospital rscategory stratum numerator denominator m2xbv m2xbu m2xbvu
expand denominator
sort stratum
generate patient = _n
order patient, after(stratum)
bysort stratum (patient): generate y = (numerator>=_n)
generate m2pxbv = invlogit(m2xbv)
```

```
generate m2pxbu = invlogit(m2xbu)

generate m2pxbvu = invlogit(m2xbvu)

roctab y m2pxbv

roctab y m2pxbu

roctab y m2pxbvu

compress

save "m2roc.dta", replace


* Figure 3 - Area under the Receiver Operating Characteristics Curve based on means of MCMC chains

use "m1roc.dta", clear

merge 1:1 patient using "m2roc.dta", assert(match) keep(match) nogenerate

roccomp y m1pxbv m2pxbv m2xbu m2xbvu, ///

  graph ///

  plot1opts(recast(line) lcolor(navy)) ///

  plot2opts(recast(line) lcolor(red)) ///

  plot3opts(recast(line) lcolor(green)) ///

  plot4opts(recast(line) lcolor(orange)) ///

  rlopts(lpattern(dash)) ///

  ytitle("True positive fraction") ///

  ylabel(, angle(horizontal)) ///

  xtitle("False positive fraction") ///

  legend(off) ///

  scheme(s1mono) ///

  xsize(7.25) ysize(4)

graph export "Figure 3 - Area under the Receiver Operating Characteristics Curve.png", replace width(1000)


*************************************************************************************

* Model 3. Traditional Random intercept model
```

```
********************************************************************************

* Load the data
use "Supplemental Material 1_AMIdatabase.dta", clear

* Fit random intercept by MQL1 to get parameter starting values
tab	rscategory,	gen(risk)
runmlwin proportion cons risk1-risk10, ///
  level3(hospital: cons) ///
  level2(stratum:) ///
  level1(stratum:) ///
  discrete(distribution(binomial) link(logit) denominator(denom) mql1) ///
  nopause

* Fit random intercept by MCMC to get final parameter estimates
runmlwin proportion cons risk1-risk10, ///
  level3(hospital: cons, residuals(v, savechains("m3v.dta", replace))) ///
  level2(stratum:) ///
  level1(stratum:) ///
  discrete(distribution(binomial) link(logit) denominator(denom)) ///
  mcmc(burnin(5000) chain(10000) thinning(10) hc(3)savechains("m3b.dta", replace)) ///
  initsprevious ///
  nopause

* ROC AUC based on means of MCMC chains
predict pr
gen prh=pr+v0
```

```
*Expand databse
expand denom
sort stratum
by stratum,sort: gen num=_n
by stratum: gen y=0 if numer<num
replace y=1 if y==.
gen Ph=invlogit(prh)

disp _N
gen prop =denom/43247
gen weight=int(1/prop)

roctab y Ph
roctab y Ph [fw=weight]

*****************************************************************************
* Interaction Effect
*****************************************************************************

* Load the data
use "Supplemental Material 1_AMIdatabase.dta", clear

* Sort hte data
sort hospital rscategory stratum

* Fit model 3 by MQL1 to get parameter starting values
runmlwin prop cons, ///
  level4(hospital: cons) ///
  level3(rscategory: cons) ///
```

```
  level2(stratum: cons) ///
  level1(stratum:) ///
  discrete(distribution(binomial) link(logit) denominator(denom)) ///
  nopause

* Fit model 3 by MCMC to get final parameter estimates
runmlwin prop cons, ///
  level4(hospital: cons) ///
  level3(rscategory: cons) ///
  level2(stratum: cons) ///
  level1(stratum:) ///
  discrete(distribution(binomial) link(logit) denominator(denom)) ///
  mcmc(cc burnin(5000) chain(10000) thinning(10) hc(3)) ///
  initsprevious nopause cformat(%9.3f)



******************************************************************************
exit
```
