# Supplementary material for: Cross-classified Multilevel Analysis of Individual Heterogeneity and Discriminatory Accuracy (MAIHDA) to evaluate hospital performance: the case of hospital differences in patient survival after acute myocardial infarction
Source: BMJ Open. 2020 Oct 23;10(10):e036130. doi: 10.1136/bmjopen-2019-036130 (PMC7590346; doi:10.1136/bmjopen-2019-036130)
Supplement: Supplementary data [file bmjopen-2019-036130supp003.pdf]

```
#------------------------------------------------------------------------------
# A Cross-Classified Multilevel Analysis of Individual Heterogeneity and
# Discriminatory Accuracy (MAIHDA) to evaluate hospital performance: The case of
# Hospital Differences in Patient Survival after Acute Myocardial Infarction
#------------------------------------------------------------------------------

# Load required packages
library(R2MLwiN)

# Set MLwiN path
options(MLwiN_path = 'C:/Program Files/MLwiN v3.05/')

# Load the dataset
mydata <- read.csv("Supplemental Material 1_AMIdatabase.csv")



#------------------------------------------------------------------------------
# Model 1
#------------------------------------------------------------------------------

# Fit Model 1
model1 <- runMLwiN(logit(proportion, denominator) ~ 1 + (1 | hospital),
          D = "Binomial",
          estoptions = list(EstM = 1,
                 mcmcMeth = list(burnin = 5000,
                      iterations = 10000)),
          data = mydata)
model1
```

```
#-------------------------------------------------------------------------------
# Model 2
#-------------------------------------------------------------------------------

# Fit Model 2
model2 <- runMLwiN(logit(proportion, denominator) ~ 1 + (1 | hospital)
                + (1 | rscategory),
                 D = "Binomial",
                 estoptions = list(EstM = 1, xc = TRUE,
                            mcmcMeth = list(burnin  = 5000,
                                      iterations = 10000),
                            mcmcOptions = list(hcen = 2)),
                 data = mydata)
model2


#------------------------------------------------------------------------------
```
