# Supplementary material for: Cross-classified Multilevel Analysis of Individual Heterogeneity and Discriminatory Accuracy (MAIHDA) to evaluate hospital performance: the case of hospital differences in patient survival after acute myocardial infarction
Source: BMJ Open. 2020 Oct 23;10(10):e036130. doi: 10.1136/bmjopen-2019-036130 (PMC7590346; doi:10.1136/bmjopen-2019-036130)
Supplement: Supplementary data [file bmjopen-2019-036130supp004.pdf]

hospital,cons,rscategory,stratum,proportion,numerator,denominator
1,1,1,1,0,0,99
1,1,2,2,.013157895,1,76
1,1,3,3,.02739726,2,73
1,1,4,4,.016129032,1,62
1,1,5,5,.013333334,1,75
1,1,6,6,.045977011,4,87
1,1,7,7,.074074075,8,108
1,1,8,8,.0625,7,112
1,1,9,9,.093220338,11,118
1,1,10,10,.22413793,26,116
2,1,1,11,0,0,196
2,1,2,12,.005586592,1,179
2,1,3,13,.0064935065,1,154
2,1,4,14,.029411765,4,136
2,1,5,15,.044303797,7,158
2,1,6,16,.044025157,7,159
2,1,7,17,.059210528,9,152
2,1,8,18,.14285715,27,189
2,1,9,19,.1761658,34,193
2,1,10,20,.21538462,42,195
3,1,1,21,.012048192,3,249
3,1,2,22,.021164021,4,189
3,1,3,23,.017441861,3,172
3,1,4,24,.03763441,7,186
3,1,5,25,.067114092,10,149
3,1,6,26,.066265061,11,166
3,1,7,27,.093023255,12,129
3,1,8,28,.15625,20,128
3,1,9,29,.22340426,21,94
3,1,10,30,.30526316,29,95
4,1,1,31,.01010101,1,99
4,1,2,32,.014492754,1,69
4,1,3,33,.02631579,2,76
4,1,4,34,.041666668,3,72
4,1,5,35,.051282052,4,78
4,1,6,36,.10227273,9,88
4,1,7,37,.076086953,7,92
4,1,8,38,.11627907,10,86
4,1,9,39,.20879121,19,91
4,1,10,40,.20491803,25,122
5,1,1,41,0,0,127
5,1,2,42,0,0,97
5,1,3,43,.017699115,2,113
5,1,4,44,0,0,100
5,1,5,45,0,0,93
5,1,6,46,.049019609,5,102

5,1,7,47,.054545455,6,110
5,1,8,48,.062068965,9,145
5,1,9,49,.071428575,10,140
5,1,10,50,.23809524,35,147
6,1,1,51,0,0,26
6,1,2,52,0,0,22
6,1,3,53,.033333335,1,30
6,1,4,54,.054054055,2,37
6,1,5,55,.090909094,2,22
6,1,6,56,.054054055,2,37
6,1,7,57,.0625,2,32
6,1,8,58,.125,4,32
6,1,9,59,.10714286,3,28
6,1,10,60,.21621622,8,37
7,1,1,61,0,0,26
7,1,2,62,0,0,19
7,1,3,63,.055555556,1,18
7,1,4,64,0,0,23
7,1,5,65,.11538462,3,26
7,1,6,66,.074074075,2,27
7,1,7,67,0,0,26
7,1,8,68,.13513513,5,37
7,1,9,69,.27777779,10,36
7,1,10,70,.21875,7,32
8,1,1,71,0,0,157
8,1,2,72,.023952097,4,167
8,1,3,73,.0068027209,1,147
8,1,4,74,.045112781,6,133
8,1,5,75,.037037037,5,135
8,1,6,76,.073170729,9,123
8,1,7,77,.032467533,5,154
8,1,8,78,.12698413,16,126
8,1,9,79,.086330935,12,139
8,1,10,80,.2932331,39,133
9,1,1,81,0,0,27
9,1,2,82,0,0,23
9,1,3,83,0,0,14
9,1,4,84,0,0,7
9,1,5,85,.071428575,1,14
9,1,6,86,0,0,15
9,1,7,87,0,0,7
9,1,8,88,.07692308,1,13
9,1,9,89,0,0,11
9,1,10,90,.21428572,3,14
10,1,1,91,0,0,62
10,1,2,92,.032258064,3,93
10,1,3,93,.011904762,1,84

10,1,4,94,.01369863,1,73
10,1,5,95,.03846154,3,78
10,1,6,96,.06666667,6,90
10,1,7,97,.068965517,6,87
10,1,8,98,.22580644,14,62
10,1,9,99,.1594203,11,69
10,1,10,100,.33962265,18,53
11,1,1,101,.041666668,1,24
11,1,2,102,.035714287,1,28
11,1,3,103,.027027028,1,37
11,1,4,104,0,0,50
11,1,5,105,.027027028,1,37
11,1,6,106,.071428575,2,28
11,1,7,107,.078947365,3,38
11,1,8,108,.064516127,2,31
11,1,9,109,.037037037,1,27
11,1,10,110,.40000001,6,15
12,1,1,111,0,0,10
12,1,2,112,0,0,19
12,1,3,113,0,0,13
12,1,4,114,0,0,28
12,1,5,115,0,0,14
12,1,6,116,.071428575,2,28
12,1,7,117,.14285715,3,21
12,1,8,118,0,0,18
12,1,9,119,.23529412,4,17
12,1,10,120,.14285715,2,14
13,1,1,121,0,0,135
13,1,2,122,.00729927,1,137
13,1,3,123,.035460994,5,141
13,1,4,124,.015037594,2,133
13,1,5,125,.014705882,2,136
13,1,6,126,.037313432,5,134
13,1,7,127,.096000001,12,125
13,1,8,128,.08510638,8,94
13,1,9,129,.13861386,14,101
13,1,10,130,.23863636,21,88
14,1,1,131,.022222223,1,45
14,1,2,132,0,0,44
14,1,3,133,0,0,42
14,1,4,134,.029411765,2,68
14,1,5,135,.044117648,3,68
14,1,6,136,.057692308,3,52
14,1,7,137,.068965517,4,58
14,1,8,138,.16129032,10,62
14,1,9,139,.19672132,12,61
14,1,10,140,.30000001,24,80

15,1,1,141,.03125,1,32
15,1,2,142,0,0,17
15,1,3,143,0,0,35
15,1,4,144,.079999998,2,25
15,1,5,145,.10714286,3,28
15,1,6,146,.052631579,1,19
15,1,7,147,.039999999,1,25
15,1,8,148,.17391305,4,23
15,1,9,149,.22727273,5,22
15,1,10,150,.16666667,4,24
16,1,1,151,0,0,74
16,1,2,152,.010638298,1,94
16,1,3,153,0,0,85
16,1,4,154,0,0,100
16,1,5,155,.032967035,3,91
16,1,6,156,.12195122,10,82
16,1,7,157,.064516127,6,93
16,1,8,158,.10465116,9,86
16,1,9,159,.15625,10,64
16,1,10,160,.2,16,80
17,1,1,161,0,0,18
17,1,2,162,.028571429,1,35
17,1,3,163,0,0,28
17,1,4,164,.05882353,2,34
17,1,5,165,.073170729,3,41
17,1,6,166,.10526316,4,38
17,1,7,167,.051282052,2,39
17,1,8,168,.093023255,4,43
17,1,9,169,.13793103,4,29
17,1,10,170,.36363637,12,33
18,1,1,171,.074074075,2,27
18,1,2,172,.043478262,1,23
18,1,3,173,0,0,24
18,1,4,174,0,0,26
18,1,5,175,0,0,30
18,1,6,176,.07692308,2,26
18,1,7,177,.074074075,2,27
18,1,8,178,.05882353,2,34
18,1,9,179,.07692308,3,39
18,1,10,180,.075000003,3,40
19,1,1,181,0,0,34
19,1,2,182,0,0,40
19,1,3,183,0,0,38
19,1,4,184,.07692308,3,39
19,1,5,185,0,0,45
19,1,6,186,0,0,37
19,1,7,187,.075471699,4,53

19,1,8,188,.10714286,6,56
19,1,9,189,.15000001,9,60
19,1,10,190,.24074075,13,54
20,1,1,191,0,0,20
20,1,2,192,.055555556,1,18
20,1,3,193,0,0,13
20,1,4,194,.050000001,1,20
20,1,5,195,.050000001,1,20
20,1,6,196,.043478262,1,23
20,1,7,197,.039999999,1,25
20,1,8,198,.16,4,25
20,1,9,199,.2,4,20
20,1,10,200,.23529412,4,17
21,1,1,201,0,0,21
21,1,2,202,0,0,20
21,1,3,203,0,0,18
21,1,4,204,0,0,25
21,1,5,205,.068965517,2,29
21,1,6,206,.06666667,2,30
21,1,7,207,.13636364,3,22
21,1,8,208,.10714286,3,28
21,1,9,209,.27586207,8,29
21,1,10,210,.26923078,7,26
22,1,1,211,0,0,85
22,1,2,212,.0093457941,1,107
22,1,3,213,.034188036,4,117
22,1,4,214,.024590164,3,122
22,1,5,215,.036036037,4,111
22,1,6,216,.040816326,4,98
22,1,7,217,.13829787,13,94
22,1,8,218,.11,11,100
22,1,9,219,.14285715,13,91
22,1,10,220,.20987654,17,81
23,1,1,221,.05882353,1,17
23,1,2,222,.045454547,1,22
23,1,3,223,0,0,16
23,1,4,224,.035714287,1,28
23,1,5,225,0,0,22
23,1,6,226,.07692308,2,26
23,1,7,227,.15789473,6,38
23,1,8,228,0,0,23
23,1,9,229,.15384616,4,26
23,1,10,230,.125,3,24
24,1,1,231,0,0,27
24,1,2,232,.078947365,3,38
24,1,3,233,0,0,34
24,1,4,234,0,0,28

24,1,5,235,.030303031,1,33
24,1,6,236,.11627907,5,43
24,1,7,237,.054054055,2,37
24,1,8,238,.12820514,5,39
24,1,9,239,.1724138,5,29
24,1,10,240,.24137931,7,29
25,1,1,241,.0099009899,1,101
25,1,2,242,.02,2,100
25,1,3,243,.031914894,3,94
25,1,4,244,.026548672,3,113
25,1,5,245,.073394492,8,109
25,1,6,246,.088495575,10,113
25,1,7,247,.096153848,10,104
25,1,8,248,.15730338,14,89
25,1,9,249,.21176471,18,85
25,1,10,250,.19736843,15,76
26,1,1,251,0,0,41
26,1,2,252,0,0,43
26,1,3,253,0,0,48
26,1,4,254,0,0,47
26,1,5,255,.035714287,2,56
26,1,6,256,.022727273,1,44
26,1,7,257,.03773585,2,53
26,1,8,258,.087719299,5,57
26,1,9,259,.10294118,7,68
26,1,10,260,.31645569,25,79
27,1,1,261,0,0,9
27,1,2,262,0,0,17
27,1,3,263,0,0,17
27,1,4,264,.047619049,1,21
27,1,5,265,0,0,18
27,1,6,266,0,0,20
27,1,7,267,.071428575,1,14
27,1,8,268,0,0,26
27,1,9,269,.14285715,5,35
27,1,10,270,.28947368,11,38
28,1,1,271,.0625,1,16
28,1,2,272,0,0,20
28,1,3,273,0,0,17
28,1,4,274,0,0,19
28,1,5,275,0,0,33
28,1,6,276,.0625,1,16
28,1,7,277,.11764706,2,17
28,1,8,278,.086956523,2,23
28,1,9,279,.20588236,7,34
28,1,10,280,.10869565,5,46
29,1,1,281,0,0,145

29,1,2,282,.006289308,1,159
29,1,3,283,.015037594,2,133
29,1,4,284,.02631579,3,114
29,1,5,285,.046153847,6,130
29,1,6,286,.049295776,7,142
29,1,7,287,.089655176,13,145
29,1,8,288,.11258278,17,151
29,1,9,289,.10559006,17,161
29,1,10,290,.25882354,44,170
30,1,1,291,.0040816325,1,245
30,1,2,292,.022522522,5,222
30,1,3,293,.031390134,7,223
30,1,4,294,.02293578,5,218
30,1,5,295,.025,5,200
30,1,6,296,.070484579,16,227
30,1,7,297,.078313254,13,166
30,1,8,298,.15882353,27,170
30,1,9,299,.1641791,33,201
30,1,10,300,.26035503,44,169
31,1,1,301,0,0,30
31,1,2,302,.05882353,1,17
31,1,3,303,0,0,23
31,1,4,304,0,0,13
31,1,5,305,0,0,15
31,1,6,306,0,0,16
31,1,7,307,.083333336,1,12
31,1,8,308,.16666667,4,24
31,1,9,309,.125,2,16
31,1,10,310,.40000001,10,25
32,1,1,311,.010526316,1,95
32,1,2,312,0,0,86
32,1,3,313,.013513514,1,74
32,1,4,314,.032786883,2,61
32,1,5,315,.030769231,2,65
32,1,6,316,.075949363,6,79
32,1,7,317,.04225352,3,71
32,1,8,318,.12820514,10,78
32,1,9,319,.14444445,13,90
32,1,10,320,.31325302,26,83
33,1,1,321,0,0,39
33,1,2,322,0,0,19
33,1,3,323,0,0,38
33,1,4,324,0,0,39
33,1,5,325,.018867925,1,53
33,1,6,326,.023809524,1,42
33,1,7,327,.10869565,5,46
33,1,8,328,.087719299,5,57

33,1,9,329,.15714286,11,70
33,1,10,330,.23170732,19,82
34,1,1,331,.016393442,1,61
34,1,2,332,.020408163,1,49
34,1,3,333,0,0,35
34,1,4,334,.03508772,2,57
34,1,5,335,.01923077,1,52
34,1,6,336,.050000001,3,60
34,1,7,337,.093333334,7,75
34,1,8,338,.11904762,10,84
34,1,9,339,.13043478,12,92
34,1,10,340,.2820513,33,117
35,1,1,341,0,0,26
35,1,2,342,.027777778,1,36
35,1,3,343,.078947365,3,38
35,1,4,344,0,0,34
35,1,5,345,.034482758,1,29
35,1,6,346,.07692308,3,39
35,1,7,347,.095238097,4,42
35,1,8,348,.088888891,4,45
35,1,9,349,.23684211,9,38
35,1,10,350,.33333334,17,51
36,1,1,351,.0060422961,2,331
36,1,2,352,.0094936704,3,316
36,1,3,353,.018808777,6,319
36,1,4,354,.026229508,8,305
36,1,5,355,.028037382,9,321
36,1,6,356,.055555556,17,306
36,1,7,357,.084639497,27,319
36,1,8,358,.1320132,40,303
36,1,9,359,.2109375,54,256
36,1,10,360,.2605364,68,261
37,1,1,361,.03846154,1,26
37,1,2,362,0,0,30
37,1,3,363,.027777778,1,36
37,1,4,364,0,0,35
37,1,5,365,.023255814,1,43
37,1,6,366,.06666667,2,30
37,1,7,367,.051282052,2,39
37,1,8,368,.13513513,5,37
37,1,9,369,.12765957,6,47
37,1,10,370,.25,14,56
38,1,1,371,0,0,96
38,1,2,372,.022727273,2,88
38,1,3,373,.020618556,2,97
38,1,4,374,.020833334,2,96
38,1,5,375,.07079646,8,113

38,1,6,376,.065420561,7,107
38,1,7,377,.10084034,12,119
38,1,8,378,.16129032,20,124
38,1,9,379,.17880794,27,151
38,1,10,380,.24324325,36,148
39,1,1,381,.012820513,1,78
39,1,2,382,.022988506,2,87
39,1,3,383,.0125,1,80
39,1,4,384,.04109589,3,73
39,1,5,385,.033707865,3,89
39,1,6,386,.033707865,3,89
39,1,7,387,.125,8,64
39,1,8,388,.15306123,15,98
39,1,9,389,.15909091,14,88
39,1,10,390,.31999999,24,75
40,1,1,391,0,0,12
40,1,2,392,0,0,18
40,1,3,393,0,0,12
40,1,4,394,0,0,15
40,1,5,395,.03125,1,32
40,1,6,396,.055555556,1,18
40,1,7,397,0,0,18
40,1,8,398,.23809524,5,21
40,1,9,399,.31818181,7,22
40,1,10,400,.39285713,11,28
41,1,1,401,.010869565,1,92
41,1,2,402,.015625,2,128
41,1,3,403,.02631579,3,114
41,1,4,404,.0070921984,1,141
41,1,5,405,.056451611,7,124
41,1,6,406,.10687023,14,131
41,1,7,407,.10144927,14,138
41,1,8,408,.10738255,16,149
41,1,9,409,.15231788,23,151
41,1,10,410,.20512821,24,117
42,1,1,411,0,0,101
42,1,2,412,0,0,108
42,1,3,413,.01369863,2,146
42,1,4,414,.045801528,6,131
42,1,5,415,.069230773,9,130
42,1,6,416,.048780486,6,123
42,1,7,417,.12844037,14,109
42,1,8,418,.070422538,5,71
42,1,9,419,.14117648,12,85
42,1,10,420,.24637681,17,69
43,1,1,421,0,0,17
43,1,2,422,0,0,26

43,1,3,423,0,0,24
43,1,4,424,.071428575,1,14
43,1,5,425,0,0,23
43,1,6,426,0,0,17
43,1,7,427,.043478262,1,23
43,1,8,428,.30000001,6,20
43,1,9,429,.15000001,3,20
43,1,10,430,.22916667,11,48
44,1,1,431,0,0,11
44,1,2,432,0,0,18
44,1,3,433,0,0,14
44,1,4,434,0,0,7
44,1,5,435,0,0,14
44,1,6,436,0,0,17
44,1,7,437,.14285715,3,21
44,1,8,438,.22222222,4,18
44,1,9,439,.39130434,9,23
44,1,10,440,.27272728,6,22
45,1,1,441,.01459854,2,137
45,1,2,442,.016393442,3,183
45,1,3,443,.035460994,5,141
45,1,4,444,.03267974,5,153
45,1,5,445,.029629629,4,135
45,1,6,446,.056451611,7,124
45,1,7,447,.094017096,11,117
45,1,8,448,.05982906,7,117
45,1,9,449,.15789473,12,76
45,1,10,450,.28395063,23,81
46,1,1,451,0,0,16
46,1,2,452,0,0,20
46,1,3,453,.052631579,1,19
46,1,4,454,.071428575,1,14
46,1,5,455,.10714286,3,28
46,1,6,456,.11538462,3,26
46,1,7,457,.22222222,6,27
46,1,8,458,.15151516,5,33
46,1,9,459,.24137931,7,29
46,1,10,460,.42857143,18,42
47,1,1,461,0,0,8
47,1,2,462,.0625,1,16
47,1,3,463,.1,2,20
47,1,4,464,.05882353,1,17
47,1,5,465,0,0,20
47,1,6,466,.15789473,3,19
47,1,7,467,.032258064,1,31
47,1,8,468,.10526316,2,19
47,1,9,469,.29166666,7,24

47,1,10,470,.26666668,8,30
48,1,1,471,.028169014,2,71
48,1,2,472,0,0,71
48,1,3,473,0,0,63
48,1,4,474,.014492754,1,69
48,1,5,475,.017241379,1,58
48,1,6,476,.055555556,4,72
48,1,7,477,.083333336,6,72
48,1,8,478,.16129032,15,93
48,1,9,479,.13095239,11,84
48,1,10,480,.2238806,15,67
49,1,1,481,0,0,24
49,1,2,482,0,0,16
49,1,3,483,0,0,30
49,1,4,484,0,0,35
49,1,5,485,.023255814,1,43
49,1,6,486,0,0,43
49,1,7,487,.083333336,4,48
49,1,8,488,.11627907,5,43
49,1,9,489,.2,7,35
49,1,10,490,.17647059,6,34
50,1,1,491,0,0,129
50,1,2,492,.027777778,3,108
50,1,3,493,.014705882,2,136
50,1,4,494,.0081967209,1,122
50,1,5,495,.020408163,2,98
50,1,6,496,.05050505,5,99
50,1,7,497,.061946902,7,113
50,1,8,498,.1388889,10,72
50,1,9,499,.12244898,12,98
50,1,10,500,.30434781,21,69
51,1,1,501,0,0,21
51,1,2,502,0,0,25
51,1,3,503,0,0,35
51,1,4,504,.039999999,1,25
51,1,5,505,.055555556,2,36
51,1,6,506,.11627907,5,43
51,1,7,507,.037037037,1,27
51,1,8,508,.14285715,3,21
51,1,9,509,.17777778,8,45
51,1,10,510,.19047619,8,42
52,1,1,511,0,0,3
52,1,2,512,0,0,9
52,1,3,513,0,0,9
52,1,4,514,0,0,17
52,1,5,515,0,0,15
52,1,6,516,0,0,12

52,1,7,517,.22222222,2,9
52,1,8,518,.083333336,1,12
52,1,9,519,0,0,10
52,1,10,520,.18181819,2,11
53,1,1,521,0,0,7
53,1,2,522,0,0,12
53,1,3,523,0,0,18
53,1,4,524,.07692308,1,13
53,1,5,525,0,0,13
53,1,6,526,0,0,23
53,1,7,527,.083333336,1,12
53,1,8,528,.071428575,1,14
53,1,9,529,.14285715,3,21
53,1,10,530,.071428575,1,14
54,1,1,531,.030927835,3,97
54,1,2,532,.016393442,2,122
54,1,3,533,.034188036,4,117
54,1,4,534,.016806724,2,119
54,1,5,535,.028037382,3,107
54,1,6,536,.032786883,4,122
54,1,7,537,.10084034,12,119
54,1,8,538,.10891089,11,101
54,1,9,539,.18518518,15,81
54,1,10,540,.32075471,34,106
55,1,1,541,0,0,27
55,1,2,542,0,0,53
55,1,3,543,0,0,30
55,1,4,544,0,0,38
55,1,5,545,0,0,37
55,1,6,546,0,0,38
55,1,7,547,.07692308,3,39
55,1,8,548,.10344828,3,29
55,1,9,549,.12903225,4,31
55,1,10,550,.1891892,7,37
56,1,1,551,.021276595,1,47
56,1,2,552,0,0,38
56,1,3,553,.039215688,2,51
56,1,4,554,.019607844,1,51
56,1,5,555,0,0,37
56,1,6,556,.063829787,3,47
56,1,7,557,.063829787,3,47
56,1,8,558,.14285715,6,42
56,1,9,559,.18181819,8,44
56,1,10,560,.21875,7,32
57,1,1,561,.014492754,1,69
57,1,2,562,0,0,73
57,1,3,563,.011494253,1,87

57,1,4,564,.033333335,3,90
57,1,5,565,.045454547,4,88
57,1,6,566,.054054055,4,74
57,1,7,567,.084337346,7,83
57,1,8,568,.14473684,11,76
57,1,9,569,.17647059,12,68
57,1,10,570,.30864197,25,81
58,1,1,571,0,0,22
58,1,2,572,.043478262,1,23
58,1,3,573,0,0,42
58,1,4,574,.057142857,2,35
58,1,5,575,0,0,34
58,1,6,576,.060606062,2,33
58,1,7,577,0,0,34
58,1,8,578,.16666667,7,42
58,1,9,579,.10714286,3,28
58,1,10,580,.2,6,30
59,1,1,581,0,0,12
59,1,2,582,.037037037,1,27
59,1,3,583,.035714287,1,28
59,1,4,584,.043478262,1,23
59,1,5,585,.03846154,1,26
59,1,6,586,.13333334,2,15
59,1,7,587,.052631579,1,19
59,1,8,588,0,0,30
59,1,9,589,.22222222,4,18
59,1,10,590,.30434781,7,23
60,1,1,591,.015151516,1,66
60,1,2,592,.015873017,1,63
60,1,3,593,.04225352,3,71
60,1,4,594,0,0,80
60,1,5,595,.016129032,1,62
60,1,6,596,.050847456,3,59
60,1,7,597,.11320755,6,53
60,1,8,598,.065217391,3,46
60,1,9,599,.18181819,8,44
60,1,10,600,.1627907,7,43
61,1,1,601,.0066666668,1,150
61,1,2,602,.016666668,2,120
61,1,3,603,.033613447,4,119
61,1,4,604,.036036037,4,111
61,1,5,605,.021978023,2,91
61,1,6,606,.036144577,3,83
61,1,7,607,.11111111,10,90
61,1,8,608,.17105263,13,76
61,1,9,609,.12371134,12,97
61,1,10,610,.29487181,23,78

62,1,1,611,0,0,31
62,1,2,612,.039999999,1,25
62,1,3,613,0,0,31
62,1,4,614,0,0,35
62,1,5,615,.055555556,2,36
62,1,6,616,.05882353,2,34
62,1,7,617,.10714286,3,28
62,1,8,618,.081081077,3,37
62,1,9,619,.125,6,48
62,1,10,620,.2,7,35
63,1,1,621,0,0,17
63,1,2,622,0,0,18
63,1,3,623,.095238097,2,21
63,1,4,624,.043478262,1,23
63,1,5,625,.03846154,1,26
63,1,6,626,0,0,20
63,1,7,627,.047619049,1,21
63,1,8,628,.17647059,3,17
63,1,9,629,.11111111,2,18
63,1,10,630,.17647059,3,17
64,1,1,631,0,0,31
64,1,2,632,0,0,25
64,1,3,633,.125,4,32
64,1,4,634,.15000001,3,20
64,1,5,635,.19354838,6,31
64,1,6,636,.074074075,2,27
64,1,7,637,.16,4,25
64,1,8,638,.26086956,6,23
64,1,9,639,.34615386,9,26
64,1,10,640,.46666667,7,15
65,1,1,641,0,0,32
65,1,2,642,0,0,22
65,1,3,643,0,0,24
65,1,4,644,0,0,28
65,1,5,645,0,0,32
65,1,6,646,.045454547,2,44
65,1,7,647,0,0,29
65,1,8,648,.12,3,25
65,1,9,649,.24137931,7,29
65,1,10,650,.13636364,3,22
66,1,1,651,0,0,18
66,1,2,652,0,0,30
66,1,3,653,0,0,16
66,1,4,654,.095238097,2,21
66,1,5,655,0,0,28
66,1,6,656,.050000001,1,20
66,1,7,657,.039999999,1,25

66,1,8,658,.16129032,5,31
66,1,9,659,.24137931,7,29
66,1,10,660,.32258064,10,31
67,1,1,661,0,0,17
67,1,2,662,0,0,12
67,1,3,663,.047619049,1,21
67,1,4,664,0,0,16
67,1,5,665,0,0,11
67,1,6,666,.16666667,2,12
67,1,7,667,0,0,15
67,1,8,668,.10526316,2,19
67,1,9,669,.21052632,4,19
67,1,10,670,.38095239,8,21
68,1,1,671,0,0,70
68,1,2,672,0,0,48
68,1,3,673,0,0,57
68,1,4,674,.032786883,2,61
68,1,5,675,.039215688,2,51
68,1,6,676,.06666667,3,45
68,1,7,677,.12727273,7,55
68,1,8,678,.13636364,9,66
68,1,9,679,.19642857,11,56
68,1,10,680,.31481481,17,54
