# Supplementary material for: Cross-classified Multilevel Analysis of Individual Heterogeneity and Discriminatory Accuracy (MAIHDA) to evaluate hospital performance: the case of hospital differences in patient survival after acute myocardial infarction
Source: BMJ Open. 2020 Oct 23;10(10):e036130. doi: 10.1136/bmjopen-2019-036130 (PMC7590346; doi:10.1136/bmjopen-2019-036130)
Supplement: Author's manuscript [file bmjopen-2019-036130.draft_revisions.pdf]

BMJ Open is committed to open peer review. As part of this commitment we make the peer review history of every article we publish publicly available.

When an article is published we post the peer reviewers' comments and the authors' responses online. We also post the versions of the paper that were used during peer review. These are the versions that the peer review comments apply to.

The versions of the paper that follow are the versions that were submitted during the peer review process. They are not the versions of record or the final published versions. They should not be cited or distributed as the published version of this manuscript.

BMJ Open is an open access journal and the full, final, typeset and author-corrected version of record of the manuscript is available on our site with no access controls, subscription charges or pay-per-view fees (http://bmjopen.bmj.com).

If you have any questions on BMJ Open's open peer review process please email info.bmjopen@bmj.com

BMJ Open

A Cross-Classified Multilevel Analysis of Individual Heterogeneity and Discriminatory Accuracy (MAIHDA) to evaluate hospital performance: The case of Hospital Differences in Patient Survival after Acute Myocardial Infarction

Journal:	*BMJ Open*
Manuscript ID	bmjopen-2019-036130
Article Type:	Original research
Date Submitted by the Author:	04-Dec-2019

Complete List of Authors:	Rodriguez-Lopez, Merida; Pontificia Universidad Javeriana - Cali,<br>Merlo, Juan; Lund University, Faculty of Medicine, Social Epidemiology<br>Perez-Vicente, Raquel; Lund University, Faculty of Medicine, Social Epidemiology<br>Austin, Peter; University of Toronto<br>Leckie, George; University of Bristol, Multilevel Modelling

Keywords:	EPIDEMIOLOGY, Quality in health care < HEALTH SERVICES ADMINISTRATION & MANAGEMENT, Adult cardiology < CARDIOLOGY

SCHOLARONE™
Manuscripts

I, the Submitting Author has the right to grant and does grant on behalf of all authors of the Work (as defined in the below author licence), an exclusive licence and/or a non-exclusive licence for contributions from authors who are: i) UK Crown employees; ii) where BMJ has agreed a CC-BY licence shall apply, and/or iii) in accordance with the terms applicable for US Federal Government officers or employees acting as part of their official duties; on a worldwide, perpetual, irrevocable, royalty-free basis to BMJ Publishing Group Ltd ("BMJ") its licensees and where the relevant Journal is co-owned by BMJ to the co-owners of the Journal, to publish the Work in this journal and any other BMJ products and to exploit all rights, as set out in our licence.

The Submitting Author accepts and understands that any supply made under these terms is made by BMJ to the Submitting Author unless you are acting as an employee on behalf of your employer or a postgraduate student of an affiliated institution which is paying any applicable article publishing charge ("APC") for Open Access articles. Where the Submitting Author wishes to make the Work available on an Open Access basis (and intends to pay the relevant APC), the terms of reuse of such Open Access shall be governed by a Creative Commons licence – details of these licences and which Creative Commons licence will apply to this Work are set out in our licence referred to above.

Other than as permitted in any relevant BMJ Author's Self Archiving Policies, I confirm this Work has not been accepted for publication elsewhere, is not being considered for publication elsewhere and does not duplicate material already published. I confirm all authors consent to publication of this Work and authorise the granting of this licence.

For peer review only

A Cross-Classified Multilevel Analysis of Individual Heterogeneity and Discriminatory Accuracy (MAIHDA) to evaluate hospital performance:

The case of Hospital Differences in Patient Survival after Acute Myocardial Infarction

Merida Rodriguez-Lopez[1,2]

Juan Merlo[1,3]

Raquel Perez-Vicente [1]

Peter C. Austin[4,5,6]

George Leckie [1,7]

[1] Unit for Social Epidemiology, Faculty of Medicine, Lund University, Malmö, Sweden

[2] Pontificia Universidad Javeriana seccional Cali, Colombia

[3] Center for Primary Health Care Research, Region Skåne, Malmö, Sweden

[4] ICES, Toronto, Ontario, Canada

[5] Institute of Health Management, Policy and Evaluation, University of Toronto, Ontario, Canada

[6] Schulich Heart Research Program, Sunnybrook Research Institute, Toronto, Ontario, Canada

[7] Centre for Multilevel Modelling, University of Bristol, Bristol, UK

Short title: Hospital Performance by MAIHDA

Corresponding author: merida.rdguez@gmail.com

Patient consent: Not required.

For peer review only

Abstract

Objective

To describe a novel strategy, Multilevel Analysis of Individual Heterogeneity and Discriminatory Accuracy (MAIHDA) to evaluate hospital performance, by analyzing differences in 30-day mortality after a first-ever acute myocardial infarction (AMI) in Sweden.

.

Methods

To demonstrate our approach, we included 43,247 patients admitted to 68 Swedish hospitals between 2007-2009, with a first-ever AMI. We classified the patients into 10 categories according to a risk score (RS) for 30-day mortality and created 680 strata defined by combining hospital and RS categories. In the first step, we applied a hierarchical multilevel logistic regression model with patients nested within hospitals. In a second step, and in order to adjust for patient-mix, we performed a cross-classified multilevel model with both RS categories and hospitals as random effects. We evaluate hospital performance by combining this information with a selected benchmark value for 30-day mortality of less than 6%.

Results

In the cross-classified multilevel analysis the overall RS adjusted hospital 30-day mortality in Sweden was 4.8% and the between-hospital variation was very small (VPC= 0.9%, AUC= 0.54). The benchmark value was therefore achieved by all hospitals. However, as expected, there were large differences between the RS categories (VPC= 38.4%, AUC= 0.77)

Conclusions

MAIHDA is a useful tool to evaluate hospital performance. The benefit of this novel approach to adjusting for patient RS is that it allowed us to estimate separate variance partition coefficients (VPCs) and area under the ROC curve (AUC) statistics to simultaneously evaluate the influence of RS categories and hospital differences on mortality. At the time of our analysis, all hospitals in Sweden were performing homogenously well. That is, the benchmark target for 30-day mortality was fully achieved and there were not relevant hospital differences. Therefore, possible quality interventions should be universal and oriented to maintain the high hospital quality of care.

Keywords: Multilevel analysis, Health Evaluation, Decision Making, ROC curve, Variance analysis

Strengths and limitations of this study

We provide a new analytical tool for analysing hospital performance based on multilevel analysis of individual heterogeneity and discriminatory accuracy (MAIHDA).

Cross-classified MAIHDA disentangles the specific role of the patient-mix vs. the hospital when analyzing quality outcomes.

MAIHDA allows analysts to identify whether target or universal interventions are most appropriate to improve the quality of care.

For peer review only - http://bmjopen.bmj.com/site/about/guidelines.xhtml

We provide a three-step strategy to achieve a complete analysis of hospital performance. However, more elaborated strategies are also possible.

Source of Funding: This work was supported by grants from Swedish Research Council (#2013-2484, # 2017-01321. Principal investigator: JM).

Conflicts of Interest: None declared

For peer review only

Introduction

Hospital effects

When evaluating institutional (e.g., hospital) performance in health care, traditional studies make two implicit assumptions. First, it is assumed that over and above patient characteristics, the hospital context exerts a general, shared effect on all patients at the hospital. This general hospital-context effect is argued to reflect the influence of many factors, for instance, hospital administration, access to resources, specialized knowledge, implementation of methods for disease management, and adoption of guidelines and pathways for patient treatments. Second, it is often assumed that the general hospital-context effect can be measured by quantifying differences between hospital averages in certain quality indicators. Therefore, the focus of the analysis is based on the interpretation of tables, funnel plots, control charts, 'league tables' or similar, where hospitals are ranked according to different quality indicators such as their average 30-day mortality after acute myocardial infarction (AMI) (1). Occasionally such analyses are accompanied by an estimation of the reliability and suitability of the ranking ("*rankability*") (2), but more often than not the focus of analysis remains on hospital averages.

Multilevel Analysis of Individual Heterogeneity and Discriminatory Accuracy (MAIHDA)

Recently, MAIHDA has been proposed as a novel strategy for evaluating hospital performance (3). In contrast with most traditional studies, hospital MAIHDA simultaneously focuses on both hospital averages *and* patient heterogeneity around such averages. In MAIHDA, the fundamental statement is that patient and hospital variation should not be analysed separately. Rather, we need to consider that the total individual outcome variance can be partitioned into variance components operating at different levels of analysis (4). From this perspective, hospital differences are not measured as the difference between hospital averages, but as the hospital general contextual effect (GCE). That is, the share of the total individual variance in patient outcomes that is at the hospital level. This definition aligns with that for the variance partition coefficient (VPC) in multilevel modelling. The greater the GCE, the more important the hospital context is to explain the individual outcome distribution (5-7). This idea is also closely related to the notion of discriminatory accuracy developed for the evaluation of the performance of prognostic and screening markers in medicine (8, 9). It is therefore possible to also use measures of discriminatory accuracy such as the area under the Receiving Operator Characteristics curve (AUC), to quantify the hospital GCE (10). See elsewhere for an extended explanation of the GCE concept (3, 7, 11). In this article, we argue that the systematic application of measures of variance and discriminatory accuracy is of fundamental relevance for meaningful performance evaluations (3, 5, 11-13).

Cross-classified MAIHDA

Hospital comparisons are usually adjusted for "patient-mix" using a risk score (RS). In traditional multilevel analysis of hospital performance, patient RS effects are modelled as fixed effects (e.g., by entering the set of RS categories as a series of dummy variables) while the hospital effects are modelled as random effects. In contrast, in the cross-classified MAIHDA approach both the RS and the hospitals are modelled as random effects. Readers familiar with the traditional application of multilevel modelling may query the treatment of RS categories as

For peer review only - http://bmjopen.bmj.com/site/about/guidelines.xhtml

random effects. For example, while we can think of the hospitals as a sample drawn from the set of all possible hospitals, it proves harder to conceptualize the RS categories in this way as there is not a large population of RS categories from which they are drawn. This is, however, a philosophical, rather than a practical question. In fact, when studying hospitals in a country the hospitals are never a sample of an infinite super population of hospitals but a concrete set of facilities in a specific setting. Furthermore, many multilevel studies observe and analyze all the hospitals in a country in their data, and the total number of hospitals may not prove that large, yet here too the hospital effects will be treated as random effects. As discussed by Snijders and Bosker, when defining the random intercept model (14), p. 45, the random effects model can be applied even when the idea of an infinite superpopulation is less evident. This approach is currently being applied when performing intersectional MAIHDA in social epidemiology (15).

The cross-classified approach provides several advantages over the traditional hierarchical multilevel approach. First, the cross-classified MAIHDA is parsimonious as it includes only one random parameter for the n RS categories rather than the $n-1$ dummy variables as in the fixed effects approach. Second, the cross-classified approach provides separate VPCs and AUCs for RS categories and for the hospital, allowing their magnitude to be contrasted. Thus, in contrast to the fixed-effects approach, it allows the importance of patient-mix vs. the hospital effects to be communicated on a common metric. In addition, hospital MAIHDA provides all the usual advantages of multilevel models. For instance, by providing reliability weighted hospital averages (shrunken residuals), it reduces the concern of monitoring outcome measures based on small hospital caseloads which otherwise may lead to extreme and unstable hospital rankings and, therefore, unreliable performance evaluation (16, 17). Both hierarchical and cross-classified MAIHDA are nowadays easy to implement in available software such as MLwiN that can be run from both within Stata (runmlwin) (18) and within R (R2MLwiN) (19).

For peer review only - http://bmjopen.bmj.com/site/about/guidelines.xhtml

Finally, for binary patient outcomes, such as 30-day mortality after AMI, multilevel analyses can be performed using a simple contingency table or matrix with strata defined by combinations of the hospitals and the RS categories capturing patient-mix. The only information required for the analysis is the overall number of patients and the number of AMI cases in each hospital-RS stratum. This aggregated approach maintains the joint distribution of the hospitals-RS information and provides the same model results (parameter estimates, standard errors, fit statistics and predictions) as when analysing the underlying individual level data. The aggregated approach also allows computationally efficient (fast) estimation as it allows analysing thousands of patients' outcomes using a dataset consisting of just a few hundred strata. A further benefit of the aggregated approach is that the data can be shared since its aggregated presentation reduces ethical problems of confidentiality (statistical disclosure is not at risk). This in turn, improves the transparency of the research and facilitates the replication of the analysis and encourages the sharing of data to compare hospital performance between different settings.

The aim of this study

The aim of this study was to demonstrate a novel statistical approach (MAIHDA) to evaluate hospital performance and to orientate stakeholders to make assertive data informed decisions using a three-step framework. We do so by analyzing differences in 30-day mortality among patients admitted to the Swedish hospital with a first-ever AMI between 2007-2009.

For peer review only - http://bmjopen.bmj.com/site/about/guidelines.xhtml

Methods

Study Population

This is a cross-sectional study. We used information from the Swedish Patient Register (20) and from the Cause of Death Register (21) (National Board of Health and Welfare) as well as from Population Register (22) (Statistics Sweden). To ensure the anonymity of the subjects, the Swedish authorities transformed the personal identification numbers of the individuals (23) into arbitrary personal numbers before delivering the research databases to us, and we linked the databases using the anonymized identification number.

From the research database we identified all 47,462 patients with a discharge diagnosis of AMI coded as I21 according the 10th edition of the International Classification of Diseases (ICD), admitted to Swedish hospitals between 2007 and 2009 and being 45 to 80 years old. The flow diagram of the study population is shown in Figure 1.

Figure 1. Flow diagram showing the selection of patients with first diagnosis of acute myocardial infarction (AMI) 2007-2009 who were included in the study population.

Ethical statement

This research was done without patient involvement. The Regional Ethics Review Board in southern Sweden (# 2012/637) as well as the data safety committees from the National Board of Health and Welfare and from Statistics Sweden approved the construction of the database used in this study.

Data accessibility

The original databases used in our study are available from the Swedish National Board of Health and Welfare and Statistics. In Sweden, register data are protected by strict rules of confidentiality (24) but can be made available for research after a special review that includes

For peer review only

approval of the research project by both an Ethics Committee and the authorities' own data safety committees. The Swedish authorities under the Ministry of Health and Social Affairs do not provide individual level data to researchers abroad. Instead, they normally advise researchers in other countries to cooperate with Swedish colleagues and analyze data in collaboration according to standard legal provisions and procedures.

However, in the approach we propose, it is technically possible to perform the analysis using a simple table defined by hospital and categories of risk score. The aggregated information as well as the additional encryption of the hospital name fully anonymized the table, which prevents the backwards identification of individuals even when very few patients are in a single cell of the table. Therefore, to increase transparency and facilitate the replication of our analysis we provide the table as a Stata dataset (Supplementary material S1) and a fully annotated Stata do-file to allow the replication of the analyses (Supplementary material S2).

Assessment of variables

Patient outcome

The study outcome was all-cause mortality within 30-days after admission to the hospital (coded yes vs. no) due to AMI, in any location.

Risk score for mortality

An inherent difficulty when investigating quality outcome indicators such as mortality is the threat of confounding originated by patient-mix. The geographical areas covered by the hospital may vary in the demographical and disease characteristics of the patients. Furthermore, patients with a worse prognosis may be channeled to certain hospitals providing specialized care and this selection of patients will further confound the evaluations of hospitals differences. To reduce this form of confounding we computed a RS for 30-day mortality in the sample of AMI patients. Initially we selected a priori 40 variables including sex (man vs. woman), age in years

For peer review only - http://bmjopen.bmj.com/site/about/guidelines.xhtml

(as a quadratic function) and several previous diseases (ICD-10 codes) registered in the Swedish Patient Register. We then modeled 30-day mortality using a single-level logistic regression in STATA 14 (Statacorp, College Station, Texas, US), and stepwise variable selection with a significance level of 0.10 and 0.05 for removal and inclusion of variables, respectively. The variables retained in the final model were, age, arrhythmia (I48-I49), cancer (C1-D4), diseases of the cerebral arteries (I6), chronic diseases of the lower respiratory tract (J4), diabetes (E10-E14), digestive diseases (K0-K9), other types of heart disease (I3-I5), hypertension (I10-I13 I15), hearth failure (I50), injury (S00-T14), ischemic coronary artery disease (I20-I25), lung cancer (C34), mental diseases (F0-F9), and peripheral vascular disease (I74 I80) as well as respiratory diseases (J0-J9). Finally, we obtained the predicted probability of 30-day mortality and defined it as our patient risk score (RS). We discretized the RS into 10 categories using the decile values of the RS distribution. We chose deciles to provide enough granulation of the continuous RS variable and enough categories to be included as a random effect in the multilevel model.

Statistical analyses

We analysed 30-day mortality among 43,247 patients admitted to 68 Swedish hospitals between 2007-2009, with a first-ever AMI. We classified the patients into 10 RS categories for 30-day mortality and created 680 strata defined by combining hospital and RS categories. In the first step (model 1), we applied a traditional hierarchical multilevel logistic regression model with patients clustered within hospitals. In a second step (model 2), and in order to adjust for patient-mix, we performed a cross-classified multilevel model of patient outcomes with both RS categories and hospitals random effects (25). We estimated the variance partition coefficient (VPC) and the area under the ROC curve (AUC) to evaluate differences between RS categories and between hospitals in a common way.

For peer review only - http://bmjopen.bmj.com/site/about/guidelines.xhtml

Estimation methods

We performed the estimations using Markov Chain Monte Carlo (MCMC) methods, with diffuse (vague, flat, or minimally informative) prior distributions for all parameters. We used quasilikelihood methods to provide starting values for all parameters. For each model, the burn-in length was 5,000 iterations. Visual assessments of the parameter chains and standard MCMC convergence diagnostics suggested that the monitored chains had converged. We ran the model for a further 10,000 monitoring iterations and used the resulting parameter chains from the MCMC to construct 95% credible intervals (CI) for all model predictions to communicate statistical uncertainty (Supplementary material S2).

Model 1: Unadjusted multilevel analysis

Model 1 was a multilevel logistic regression for patient mortality where we only include a hospital random effect to account for the variation in mortality rates across hospitals. Let, y_j denote the number of deaths in the hospital j ($j = 1,\ldots,68$). The model can then be written as

$$y_j \sim \text{Binomial}(n_j, \pi_j)$$
$$\text{logit}\,(\pi_j) \equiv \log\left(\frac{\pi_j}{1-\pi_j}\right) = \beta_0 + H_j$$
$$H_j \sim N(0, \sigma_H^2)$$

Formula 1

where n_j denotes the total number of patients in hospital j, π_j denotes the probability of death for patients in that hospital, β_0 denotes the intercept or precision weighted grand mean, that in logistic regression represents the average of the hospital-specific shrunken (i.e., reliability-weighted) residuals values, and H_j denotes the hospital-specific random effects for the 68 hospitals (26). The random effects are assumed to be normally distributed with zero mean and between-hospital variance σ_H^2.

The purpose of this model was to evaluate unadjusted hospital differences in average mortality

For peer review only - http://bmjopen.bmj.com/site/about/guidelines.xhtml

risk. For this aim, we (A) ranked the hospitals according to their mortality risk; and (B) complemented this information by quantifying the size of the GCE.

A) Ranking of the hospitals

To rank hospitals according to their *unadjusted* mortality rates, we predict the absolute risk (AR_j) of 30-day mortality and its 95% credible interval (CI) in each hospital. To do so, we first transformed the predicted logit of 30-day mortality into proportions (π_j) as follows

$$AR_H \equiv \pi_j \equiv \text{logit}^{-1}(\beta_0 + H_j) = \frac{\exp(\beta_0 + H_j)}{1 + \exp(\beta_0 + H_j)}$$

Formula 2

B) Measuring the hospital GCE.

We estimate the hospital GCE by means of two measures:

(i) The Variance Partition Coefficient for the hospital level (VPC_H)

The VPC_H can be calculated based on the latent response formulation of the model which is an approach widely adopted today in multilevel applied work (27-31) .

$$VPC_H = \frac{\sigma_H^2}{\sigma_H^2 + \frac{\pi^2}{3}}$$

Formula 3

Where $\frac{\pi^2}{3} \cong 3.29$ denotes the variance of a standard logistic distribution. We then multiply the VPC_H by 100 and interpret it as a percentage.

The VPC_H, quantifies the share of the total individual differences in the latent propensity of 30-day mortality that is at the hospital level. The VPC_H embraces the influence of the hospital context on the patient outcome without identifying any specific hospital information. However, the VPC_H, may also reflect differences in patient-mix between hospitals. In any case, theVPC_H,

For peer review only - http://bmjopen.bmj.com/site/about/guidelines.xhtml

represents the hospital ceiling effect or potential maximum influence of the hospital attended. In the absence of confounding by patient-mix, the higher the VPC_H, the higher the hospital GCE is. In other words, the more relevant the hospital context is for understanding individual variation in the latent risk for 30-day mortality.

(ii) The area under the receiver operating characteristics curve for the hospital (AUC_H)

A well-known measure of discriminatory accuracy is the AUC (10, 11, 32). In our case the hospital AUC_H measures how well the model predicted probabilities based on the attended hospitals distinguish between two outcome categories (death within 30 days or survival). The AUC_H is constructed by plotting the true positive fraction (TPF) against the false positive fraction (FPF) for different thresholds of the predicted probabilities. The AUC takes a value between 1 and 0.5 where 1 is perfect discrimination and 0.5 would be equally as informative as flipping a coin (i.e., the hospital information has no discriminatory accuracy).

We calculated the AUC_H and to account for the different number of patients in each hospital we also calculated the weighted AUC (AUC_{wH}) where every patient was weighted by the inverse of the number of patients at his/her hospital. In our study both the unweighted and the weighted AUCs were almost identical so we only present the unweighted AUC_H.

Model 2: Patient-mix adjusted multilevel analysis

Model 2 was a cross-classified multilevel logistic regression (25) for patient mortality where we include both hospital and RS category random effects to simultaneously account for the variation in mortality rates across both hospitals and RS categories and to therefore adjust the hospital effects for patient-mix. The model can be written as:

$$y_j \sim \mathrm{Binomial}(n_j, \pi_j)$$

$$\mathrm{logit}\,(\pi_{jk}) \equiv \log\left(\frac{\pi_{jk}}{1-\pi_{jk}}\right) = \beta_0 + H_j + RS_k$$

$$H_j \sim N(0, \sigma_H^2)$$

For peer review only - http://bmjopen.bmj.com/site/about/guidelines.xhtml

$$RS_k \sim N(0, \sigma^2_{RS})$$

Formula 4

where RS_k denotes the random effect associated with RS categories k ($k = 1,...,10$), which are assumed to be normally distributed with zero mean and between-RS variance σ^2_{RS}.

The predicted logit of 30-day mortality was transformed into proportions. The mortality rates from this model are standardized and represents the rate that each hospital would have experienced if all hospitals had treated the same patients, in our case a patient with an average RS value in the population or $RS_k = 0$.

The purpose of model 2 was to evaluate patient-mix adjusted hospital differences in average mortality risk. Therefore, and analogously to model 1, we ranked the hospitals according to their RS adjusted mortality risk and complemented this information by quantifying the size of the hospital GCE net of the observed patient-mix influence.

As a measure of the patient-mix adjusted hospital GCE, we obtained the hospital VPC_H as

$$\mathrm{VPC}_H = \frac{\sigma^2_H}{\sigma^2_H + \sigma^2_{RS} + \frac{\pi^2}{3}}$$

Formula 5

We also calculated the VPC for the RS category (VPC_{RS}) as

$$VPC_{RS} = \frac{\sigma^2_{RS}}{\sigma^2_H + \sigma^2_{RS} + \frac{\pi^2}{3}}$$

Formula 6

The adjusted VPC_H and VPC_{RS} inform on the share of the total individual variance in the latent propensity of 30-day mortality that is at the hospital and at the RS category level respectively, net of the influence of the other factor. Both measures are estimated on the same scale and can therefore be directly compared to evaluate the relative relevance of hospital versus patient-mix information when it comes to understanding patient differences in the latent propensity of death.

For peer review only - http://bmjopen.bmj.com/site/about/guidelines.xhtml

We also calculated the adjusted AUC for the hospital level (AUC_H) and for RS category levels (AUC_{RS}) including their specific random effects when calculating the predicted probabilities.

Software

All models were run in MLwiN 3.02 (33) called from Stata using the *runmlwin* command (18). We note that MLwiN can equally be called from within R using the sister R2MLwiN package (19) and so our analysis can also be replicated by readers in that statistical package.

Evaluating hospital performance with MAIHDA

The present cross-classified MAIHDA framework extends that which was described in a previous publication aimed to the evaluation of geographical differences in health outcomes (34). The framework proposes three steps that need be considered to achieve a complete analysis of hospital performance. However, more elaborated strategies are of course also possible, and the presented framework is open for modifications and extensions. The application of the framework in our study was as follows

Step 1. Identifying a benchmark value and evaluating the adjusted hospital mortality rates against it.

When evaluating hospital performance, we need to identify a benchmark value expressing a tolerable average level of 30-day mortality in the population of AMI patients. However, the selection of a specific benchmark is often difficult and arbitrary. We can use an *internal benchmark* defined as the β_0 obtained in model 2 (*Formula 4*). That is, the mortality rate in a hospital with an average mortality ($H_j = 0$) treating patients with an average RS ($RS_k = 0$). This choice is meaningful since comparing with a national average seems "fair" and being RS adjusted, tertiary care hospitals with more severe cases do not unfairly push the hospital effect towards a higher value as in the crude average rate. However, being an adjusted rate the value does not necessarily resemble the crude rates and can only be used for relative comparisons.

For peer review only - http://bmjopen.bmj.com/site/about/guidelines.xhtml

Other adjustments are possible such as computing adjusted hospital mortality rates by holding RS_k value equal to the 90th percentile of the RS random effect distribution. Another possibility would be to calculate the adjusted hospital rates that would arise if each hospital treated a nationally representative sample of patients. However, statistically, this would be more complex to do as this would require integrating out the RS random effect (via simulation) and so we do not pursue this here.

We could also use and *external benchmark* by applying a RS equation for 30-day mortality obtained in another country or in an international collaboration. This approach seems worthy for international comparisons, but it may also be inappropriate if the RS equation does not properly account for population demographical and co-morbidities differences in risk, and the diagnostic criteria are not fully standardized.

Finally, we could use an arbitrary rate based on previous evidence and proven expertise. Considering the data published by the Organisation for Economic Co-operation and Development (OECD) (35) and a recent review article (36), we decide a RS adjusted 30-day mortality of less than 6% as a desirable target value for the purposes of this illustrative application.

We defined three categories of achievement in relation to the benchmark, *fully reached target (≤6%), closely reached target value (*6%-8%), and *insufficiently reached target value (*≥8%). We used caterpillar graphs to compare the RS adjusted hospital rates in relation to the benchmark value of 6%.

Step 2. Quantifying the size of the hospital differences using the VPC and the AUC

Currently there is no official guidance for assessing the magnitude of the VPC or the AUC in the context of studying hospital differences in RS adjusted hospital 30-day mortality but a practical proposal is described in Table 1. This table also shows the corresponding AUC values

For peer review only - http://bmjopen.bmj.com/site/about/guidelines.xhtml

according to the simulated relationship between the AUC and VPC published elsewhere (34). The proposed values are based on the authors' own experience, but further discussion is encouraged to arrive at a standard classification. Furthermore, different standards may ultimately be required and developed for different outcomes in different contexts and at different points in time. For instance, Hosmer and Lemeshow (37) suggested that AUC of 0.70 to 0.80 are 'acceptable', 0.80 to 0.90 'excellent' and 0.9 or above 'outstanding', while AUC of 0.50 suggests discrimination by chance e.g. similar to tossing a coin to decide death or alive.

Step 3. Interpreting results to evaluate performance

The two primary questions for the hospital performance evaluation were, (i) has the benchmark value been insufficiently, closely, or fully reached? (ii) are there substantial differences between the hospitals, or do they perform homogenously? To answer both questions, we created a framework (Table 1) with 15 scenarios combining information on the benchmark value achievement and the size of hospital differences based on model 2.

In the best scenario (scenario A) the desired target level has been fully achieved overall (averaging across all hospitals the adjusted mortality rate is less than 6%), and hospital differences are effectively absent (the Hospital GCE is effectively absent). The conclusion would be that all hospitals have performed similarly well. In the worst scenario (scenario C) the desired target level has not been achieved overall, and between-hospital differences are again absent. The conclusion would be that all hospitals have performed similarly but poorly. Observe that in both scenarios A and C, interventions only targeted to specific hospitals are not justifiable. Rather any intervention should be universal (i.e., directed to all hospitals) as in both scenarios all hospitals are preforming similarly. In scenario A, the intervention would be oriented to *maintaining* the overall high quality while in scenario C, the objective would be to improve the quality in *all* hospitals.

For peer review only - http://bmjopen.bmj.com/site/about/guidelines.xhtml

The interpretation of the scenarios in the lowest corners of the table (M and O) is driven by the very large size of the hospital differences. For example, in scenario M some hospitals may not have achieved the target level even though the average 30-day mortality is below the benchmark value. In contrast, in scenario O, some hospitals may have achieved the target level even though the target has not been achieved overall. In these scenarios (M and O) targeted hospitals interventions are justified to focus especially on those hospitals operating above the benchmark value.

Results

Table 2 describes the estimation sample. The mean age of the 43,247 patients with a first-ever AMI was close to 67 years and only one third of patients were female. As expected, mortality was strongly related to the initial mortality risk of the patient so the patients in the highest RS category present a mortality risk about 36 times higher than in the low RS group (25.5% vs. 0.7%).

In the crude, unadjusted analysis based on simple proportions (i.e., obtained without multilevel modeling), 8.18% of the patients died within a 30-day period after admission to the hospital and the crude proportions ranged from 3.45% to 17.32% across hospitals, as indicated in figure 2 (white circles). However, accounting for the reliability of the hospital information in the hierarchical multilevel analysis (Model 1), the mean unadjusted hospital rate from the multilevel model was 8.00% and ranged between 6.23% and 11.67% (grey circles). Thus, the multilevel model was able to extract the systematic variation of interest between hospitals from the observed variation which additionally includes a substantial statistical noise component due to the relatively small patient caseloads per hospitals (from a statistical sample size perspective). After further adjustment for patient-mix variation across hospitals in the cross-classified

For peer review only - http://bmjopen.bmj.com/site/about/guidelines.xhtml

For peer review only

multilevel model (model 2) the mean hospital 30-day mortality was 4.88% and varied only between 3.58% and 7.59% (black circles) and the 95% credible intervals (grey vertical lines) considerably overlap each other. Thus, once we perform reliability weighted estimations and adjust for hospital differences in patient mix, very little variation in performance remains across hospitals. These adjusted mortality rates represent hospital rates as if all hospital treated the same patient with average RS (i.e., $RS_k = 0$), which is a suitable approach for evaluating hospital performance.

Table 3 informs on the results from the multilevel variance analysis used to estimate the GCE. In model 1, the VPC_H was 1.06%, and the AUC_H = 0.56. Adjustment for patient-mix by including the RS categories in the cross-classified model 2 reduced the value of the VPC_H= 0.93%, and slightly decreased the initial AUC_H= 0.54. Therefore, the hospital differences were *absent*, suggesting that hospital attended is not a driving factor in determining patient mortality. In contrast, information on the RS value of the patients was much more relevant than information on the hospital where they were treated. The VPC_{RS} was very large (i.e., 38.4%) and the AUC_{RS}= 0.77. Figure 3 clearly depicts the differential discriminatory accuracies of the hospital and RS random effects.

Figure 2. Crude (white circles), reliability weighted (grey circles) and both reliability weighted and patient-mix adjusted (black circles and confidence intervals) differences in absolute risk of 30-day mortality between hospitals in the population of 43,247 patients with a first-ever acute myocardial infarction in 2007-2009, in Sweden.

Figure 3. Area under the Receiver Operating Characteristics Curve (AUC) constructed using the predicted probability of 30-day mortality obtained from multilevel logistic regression

For peer review only - http://bmjopen.bmj.com/site/about/guidelines.xhtml

analysis with 47,462 patients with a discharge diagnosis of acute myocardial infarction (AMI) in 2007-2009 in Sweden. The AUC for the unadjusted hospitals was obtained from model 1 with patient nested within hospitals (Blue line). The rest of the curves are from model 2, a cross-classified multilevel model with patients nested within hospitals and categories (decile groups) of risk-score (RS) for 30-day mortality. (Red line: patient mix adjusted hospitals, Orange line: Risk score category, Green line: Total AUC)

For peer review only

Discussion

Analyzing 30-day mortality after AMI in Sweden, we illustrate the MAIHDA approach to auditing hospital performance. By considering both the size of the hospital GCE and the RS adjusted hospital 30-day mortality rates in relation to a pre-set benchmark value, we were able to perform a more nuanced evaluation of hospital performance as compared with traditional methods exclusively based on differences between hospital averages.

Following the framework presented in table 1, our results corresponds with the scenario A and indicate that, at the time of our analysis, all hospitals in Sweden were performing homogenously well. That is, the overall 30-day mortality (i.e., 4.8%) was under the benchmark value of ≤6% and there were not relevant hospital differences (VPC_H= 0.93% and AUC_H= 0.54). The ranking of hospitals in our study (Figure 2) also shows that no hospitals could be statistically distinguished with any degree of certainty from the overall average mortality.

We have found similar low VPC values when investigating hospital differences in mortality after AMI admission in Ontario, Canada (38) and in Sweden (39) as well as mortality after heart failure in Sweden (40) and in Denmark (3). The low hospital GCE suggests universal instead of targeted interventions, as all hospitals perform homogenously. There may be, however, other

For peer review only - http://bmjopen.bmj.com/site/about/guidelines.xhtml

patient outcomes where the hospital GCE would be much larger. For instance, when auditing adherence with guidelines for statin prescription (41, 42) or process quality indicators of diabetes care like albuminuria analysis (43).

The evaluation of institutional performance using VPC is not new (12, 39, 44, 45). Normand (46) states that when evaluating hospital performance if the VPC is zero *there are no hospital quality differences, that is, the chance that a patient experiences an event after being treated is the same regardless of the hospital (p. 33)*. This idea is also explicitly expressed by the committee assigned to set statistical guidelines for assessing hospital performance in USA (47). The share of the total variance that is at the hospital level is crucial for evaluating performance. However, this fundamental concept needs be applied more extensively. Today, it is recognized that multilevel models (hierarchical or mixed-effect models) are the preferred methodology for provider profiling. However, the substantive analysis of components of variance still receives little attention and most studies only consider multilevel modeling for its capacity to account for the clustering of patients within hospitals in order to obtain "correct standard errors" on regression coefficients and odds ratios. Some authors even conclude that hospital averages (odds ratios, observed/expected values) obtained from multilevel analyses gives similar results compared to traditional logistic regressions analyses. This situation is interpreted as an argument for keeping the traditional logistic regression as the standard method for performing risk adjustment of hospital quality comparisons (48-50). However, we do not agree with this opinion. The first reason is that the fixed effects approach does not explicitly inform on components of variance. The second reason is that the equivalence between traditional and multilevel regression results only occurs when the hospital GCE (i.e., the clustering) is low and the number of patients at the hospitals is very high (i.e., reliable estimation of hospital averages) (26). In other words, traditional non-multilevel analyses give similar results to the multilevel analysis only when the hospital differences are not relevant (i.e., low VPC) and the patient load

For peer review only - http://bmjopen.bmj.com/site/about/guidelines.xhtml

is very large in every hospital (which is rarely the case). In addition, hospital level variables appear paradoxically more statistically "significant" when the hospital level is less relevant (i.e., low VPC) (7). Information on the size of the hospital GCE is, therefore, fundamental for a sound analysis of hospital performance.

In this study we have applied the AUC to evaluate the hospital GCE. The AUC is a measure of discriminatory accuracy (DA) frequently used for gauging the performance of prognostic and screening markers in medicine (8, 9) but it can also be used to quantify hospital GCE (10, 34). So far, many epidemiologists may not be familiar with the use of measures of components of variance like the VPC for binary outcomes (30). However, the AUC measure is well established in clinical and health care epidemiology and the information they give is relatively easy to interpret and communicate. From this perspective, the evaluation of hospital performance resembles a screening test and so we must therefore know the discriminatory accuracy of, for instance, a "league table" to make informed decisions.

Analyzing outcome indicators always requires adjustment for confounding (i.e., patient-mix). We perform this adjustment by an innovative strategy that use hospitals and decile groups of RS in a cross-classified multilevel analysis. A key advantage of this approach is it allowed a directed comparison of the importance of patient case mix and hospital effects in explaining variation in patient outcomes across hospital. We did not use existing patient-mix adjustment scores such as the Charlson Risk Score (51), the Elixhauser Score (52), or the CMS Hierarchical Condition Category "CMS-HCC" risk adjuster (53). Our aim was not primarily to create a new risk score equation but only to perform a parsimonious patient-mix adjustment where the RS summarizes a large number of variables into a single construct.

In summary, we illustrate the MAIHDA approach to auditing hospital performance using a three-step strategy. We argue that it is necessary to consider both the size of the hospital GCE and the RS adjusted 30-day mortality in relation to a pre-set benchmark value. Our results

For peer review only - http://bmjopen.bmj.com/site/about/guidelines.xhtml

indicate that, at the time of our analysis, all hospitals in Sweden were performing homogenously well. That is, there were not relevant hospital differences and the benchmark target for 30-day mortality rate after admission for AMI was well achieved. Therefore, possible quality interventions should be universal and oriented to maintain the high hospital quality of care.

Funding

The Swedish Research Council (Vetenskapsrådet) supported this work through the project "Multilevel Analyses of Individual Heterogeneity: innovative concepts and methodological approaches in Public Health and Social Epidemiology": (#2017-01321, PI: Juan Merlo). Dr. Austin is supported by a Mid-Career Investigator award from the Heart and Stroke Foundation.

Contributors:

JM had the initiative of the study and acquired the data.

JM and M R-L wrote the original manuscript.

M R-L and RP-V performed the analyses in coordination with JM.

PA and GL provided advanced statistical support.

All the authors have contributed to the design of the study, developed the methodology, direction of the analyses, interpretation of the results and drafting of the manuscript

All authors have revised the last version of the manuscript.

References

1. Chung SC, Sundstrom J, Gale CP, James S, Deanfield J, Wallentin L, et al. Comparison of hospital variation in acute myocardial infarction care and outcome between Sweden and United Kingdom: population based cohort study using nationwide clinical registries. BMJ. 2015;351:h3913.
2. van Dishoeck AM, Lingsma HF, Mackenbach JP, Steyerberg EW. Random variation and rankability of hospitals using outcome indicators. BMJ Qual Saf. 2011;20(10):869-74.
3. Ghith N, Wagner P, Frolich A, Merlo J. Short Term Survival after Admission for Heart Failure in Sweden: Applying Multilevel Analyses of Discriminatory Accuracy to Evaluate Institutional Performance. PLoS One. 2016;11(2):e0148187.

For peer review only - http://bmjopen.bmj.com/site/about/guidelines.xhtml

4. Merlo J. Invited commentary: multilevel analysis of individual heterogeneity-a fundamental critique of the current probabilistic risk factor epidemiology. Am J Epidemiol. 2014;180(2):208-12; discussion 13-4.
5. Austin PC, Merlo J. Intermediate and advanced topics in multilevel logistic regression analysis. Stat Med. 2017;36(20):3257-77.
6. Merlo J, Viciana-Fernandez FJ, Ramiro-Farinas D, LDAP RG. Bringing the individual back to small-area variation studies: A multilevel analysis of all-cause mortality in Andalusia, Spain. Social Science & Medicine. 2012;75(8):1477-87.
7. Merlo J, Wagner P, Austin PC, Subramanian SV, Leckie G. General and specific contextual effects in multilevel regression analyses and their paradoxical relationship: A conceptual tutorial. SSM Popul Health. 2018;5:33-7.
8. Khoury MJ, Newill CA, Chase GA. Epidemiologic evaluation of screening for risk factors: application to genetic screening. Am J Public Health. 1985;75(10):1204-8.
9. Pepe MS, Janes H, Longton G, Leisenring W, Newcomb P. Limitations of the odds ratio in gauging the performance of a diagnostic, prognostic, or screening marker. Am J Epidemiol. 2004;159(9):882-90.
10. Wagner P, Merlo J. Measures of discriminatory accuracy in multilevel analysis. European Journal of Epidemiology. 2013;28(1, Supplement):135.
11. Merlo J, Wagner P, Ghith N, Leckie G. An Original Stepwise Multilevel Logistic Regression Analysis of Discriminatory Accuracy: The Case of Neighbourhoods and Health. PLoS One. 2016;11(4):e0153778.
12. Merlo J, Ostergren PO, Broms K, Bjorck-Linne A, Liedholm H. Survival after initial hospitalisation for heart failure: a multilevel analysis of patients in Swedish acute care hospitals. J Epidemiol Community Health. 2001;55(5):323-9.
13. Van Leeuwen W, Nilsson S, Merlo J. Mother's country of birth and prescription of psychotropic medication in Swedish adolescents: a life course approach. BMJ Open. 2012;2(5).
14. Snijders TAB, Bosker RJ. Multilevel analysis: an introduction to basic and advanced multilevel modeling. 1 ed. Thousand Oaks, California: SAGE Publications; 1999 1999///.
15. Merlo J. Multilevel analysis of individual heterogeneity and discriminatory accuracy (MAIHDA) within an intersectional framework. Soc Sci Med. 2018;203:74-80.
16. Krell RW, Hozain A, Kao LS, Dimick JB. Reliability of risk-adjusted outcomes for profiling hospital surgical quality. JAMA surgery. 2014;149(5):467-74.
17. Goldstein H, Spiegelhalter D. League Tables and Their Limitations: Statistical Issues in Comparisons of Institutional Performance. Journal of the Royal Statistical Society, SocA. 1996;159:385-443.
18. Leckie G, Charlton C. runmlwin: A Program to Run the

MLwiN Multilevel Modeling Software from within Stata. Journal of

Statistical Software. 2013;52(11):1-40.
19. Zhang Z, Parker R, Charlton C, Leckie G, Browne WJ. R2MLwiN - A program to run the MLwiN multilevel modelling software from within R. Journal of Statistical Software, . 2016;72(10):1-43.
20. Ludvigsson JF, Andersson E, Ekbom A, Feychting M, Kim JL, Reuterwall C, et al. External review and validation of the Swedish national inpatient register. BMC Public Health. 2011;11:450.
21. Brooke HL, Talback M, Hornblad J, Johansson LA, Ludvigsson JF, Druid H, et al. The Swedish cause of death register. Eur J Epidemiol. 2017;32(9):765-73.
22. Ludvigsson JF, Almqvist C, Bonamy AK, Ljung R, Michaelsson K, Neovius M, et al. Registers of the Swedish total population and their use in medical research. Eur J Epidemiol. 2016;31(2):125-36.

For peer review only

23. Ludvigsson JF, Otterblad-Olausson P, Pettersson BU, Ekbom A. The Swedish personal identity number: possibilities and pitfalls in healthcare and medical research. Eur J Epidemiol. 2009;24(11):659-67.
24. Public Access to Information and Secrecy Act: Government Offices of Sweden; 2009 [Available from: http://www.government.se/information-material/2009/09/public-access-to-information-and-secrecy-act/.
25. Leckie G. Cross-classified Multilevel Models - Concepts. LEMMA VLE Module 12, 1-60. URL: http://wwwbristolacuk/cmm/learning/online-course/. 2013.
26. Evans CR, Leckie G, Merlo J. Multilevel versus single-level regression for the analysis of multilevel information:The case of quantitative intersectional analysis. Social Science & Medicine. 2019.
27. Goldstein H, Browne WJ, Rashbash J. Partitioning variation in multilevel models. Understanding Statistics: Statistical Issues in Psychology, Education, and the Social Sciences. 2002;1(4):223-31.
28. Merlo J, Yang M, Chaix B, Lynch J, Rastam L. A brief conceptual tutorial on multilevel analysis in social epidemiology: investigating contextual phenomena in different groups of people. J Epidemiol Community Health. 2005;59(9):729-36.
29. Merlo J, Chaix B, Yang M, Lynch J, Rastam L. A brief conceptual tutorial of multilevel analysis in social epidemiology: linking the statistical concept of clustering to the idea of contextual phenomenon. J Epidemiol Community Health. 2005;59(6):443-9.
30. Merlo J, Chaix B, Ohlsson H, Beckman A, Johnell K, Hjerpe P, et al. A brief conceptual tutorial of multilevel analysis in social epidemiology: using measures of clustering in multilevel logistic regression to investigate contextual phenomena. J Epidemiol Community Health. 2006;60(4):290-7.
31. Goldstein H, Browne W, Rasbash J. Partitioning variation in generalised linear multilevel models. Understanding Statistics. 2002;1:223-32.
32. Wagner P, Merlo J. Discriminatory accuracy of a random effect in multilevel logistic regression. 20th IEA World Congress of Epidemiology (WCE2014). 2014.
33. Charlton C, Rasbash J, Browne WJ, Healy M, Cameron B. MLwiN Version 3.00. Centre for Multilevel Modelling, University of Bristol.; 2017.
34. Merlo J, Wagner P, Leckie G. A simple multilevel approach for analysing geographical inequalities in public health reports: The case of municipality differences in obesity. Health Place. 2019;58:102145.
35. OECD. Mortality following acute myocardial infarction (AMI). 2017. In: Health at a Glance 2017: OECD Indicators, [Internet]. Paris: OECD Publishing,.
36. Anderson JL, Morrow DA. Acute Myocardial Infarction. The New England journal of medicine. 2017;376(21):2053-64.
37. Hosmer DW, Lemeshow S. Applied logistic regression. 2nd ed. New York: Wiley; 2000. xii, 373 p. p.
38. Austin PC, Wagner P, Merlo J. The median hazard ratio: a useful measure of variance and general contextual effects in multilevel survival analysis. Stat Med. 2017;36(6):928-38.
39. Merlo J, Hakansson A, Beckman A, Lindblad U, Lindstrom M, Gerdtham UG, et al. [Equally good care of myocardial infarction in Sweden today. Geographic differences in mortality are without significance for the individual patient]. Lakartidningen. 2005;102(1-2):20-3.
40. Merlo J, Ostergren P, Broms K, Bjorck-Linne A, Liedholm H. Survival after initial hospitalisation for heart failure: a multilevel analysis of patients in Swedish acute care hospitals. Journal of Epidemiology and Community Health. 2001;55(5):323-9.
41. Hjerpe P, Ohlsson H, Lindblad U, Bostrom KB, Merlo J. Understanding adherence to therapeutic guidelines: a multilevel analysis of statin prescription in the Skaraborg Primary Care Database. Eur J Clin Pharmacol. 2010.

For peer review only - http://bmjopen.bmj.com/site/about/guidelines.xhtml

42. Ohlsson H, Lindblad U, Lithman T, Ericsson B, Gerdtham UG, Melander A, et al. Understanding adherence to official guidelines on statin prescribing in primary health care--a multi-level methodological approach. Eur J Clin Pharmacol. 2005;61(9):657-65.
43. Ghith N, Merlo J, Frolich A. Albuminuria measurement in diabetic care: a multilevel analysis measuring the influence of accreditation on institutional performance. BMJ Open Qual. 2019;8(1):e000449.
44. Ohlsson H, Librero J, Sundquist J, Sundquist K, Merlo J. Performance evaluations and league tables: do they capture variation between organizational units? An analysis of 5 Swedish pharmacological performance indicators. Med Care. 2011;49(3):327-31.
45. Merlo J, Gerdtham UG, Eckerlund I, Hakansson S, Otterblad-Olausson P, Pakkanen M, et al. Hospital level of care and neonatal mortality in low- and high-risk deliveries: reassessing the question in Sweden by multilevel analysis. Med Care. 2005;43(11):1092-100.
46. Normand SLT, Ash SA, Fienberg SE, Stukel TA, Utts J, Louis TA. League Tables for Hospital Comparisons. Annu Rev Stat App. 2016;3:21-50.
47. Ash AS, Fienberg E, Louis A, Norm S-lT, Stukel A, Utts PJ. STATISTICAL ISSUES IN ASSESSING HOSPITAL PERFORMANCE Commissioned by the Committee of Presidents of Statistical Societies The COPSS-CMS White Paper Committee. 2011.
48. Cohen ME, Dimick JB, Bilimoria KY, Ko CY, Richards K, Hall BL. Risk adjustment in the American College of Surgeons National Surgical Quality Improvement Program: a comparison of logistic versus hierarchical modeling. J Am Coll Surg. 2009;209(6):687-93.
49. Glance LG, Dick A, Osler TM, Li Y, Mukamel DB. Impact of changing the statistical methodology on hospital and surgeon ranking - The case of the new York state cardiac surgery report card. Medical Care. 2006;44(4):311-9.
50. Alexandrescu R, Jen MH, Bottle A, Jarman B, Aylin P. Logistic versus hierarchical modeling: an analysis of a statewide inpatient sample. J Am Coll Surg. 2011;213(3):392-401.
51. Charlson ME, Pompei P, Ales KL, MacKenzie CR. A new method of classifying prognostic comorbidity in longitudinal studies: Development and validation. Journal of Chronic Diseases. 1987;40(5):373-83.
52. Elixhauser A, Steiner C, Harris DR, Coffey RM. Comorbidity Measures for Use with Administrative Data. Medical Care. 1998;36(1):8-27.
53. Li P, Kim M, Doshi J. Comparison of the performance of the CMS Hierarchical Condition Category (CMS-HCC) risk adjuster with the charlson and elixhauser comorbidity measures in predicting mortality. BMC Health Services Research. 2010;10(1):245.

For peer review only

For peer review only - http://bmjopen.bmj.com/site/about/guidelines.xhtml

Table 1. Framework for performing hospital comparisons of 30-day mortality after acute myocardial infarction (AMI).

Size of hospital differences (Hospital GCE)			Benchmark value achievement		
			Full	Close	Insufficient
	VPC_H (%)	AUC_H	< 6%	6% - 8%	> 8%
Absent	0 to 1	0.50 to 0.55	A	B	C
Small	1 to 5	0.55 to 0.61	D	E	F
Moderate	5 to 10	0.61 to 0.66	G	H	I
large	10 to 20	0.66 to 0.72	J	K	L
Very large	>20	>0.72	M	N	O

For peer review only

For peer review only - http://bmjopen.bmj.com/site/about/guidelines.xhtml

Table 2. Characteristics of the population of 43,247 patients with a first-ever acute myocardial infarction (AMI) cared for at 68 Swedish hospitals in 2007-2009 and categorized in 10 groups by deciles of risk score for 30-day mortality (RS).

Age, *mean (SD)*	67.3 (9.0)	
Female *(%)*	33	
Number of hospitals	68	
Number of patients at the hospital, *Median (min-max)*	636 (107 - 3037)	
	Mean RS (%)	Observed mortality (
Risk Score for 30-day mortality (RS)	7.8	7.8
Decile groups of RS		
1st	1.7	0.7
2nd	2.3	1.4
3rd	2.9	2.0
4th	3.5	2.5
5th	4.2	3.7
6th	5.3	5.9
7th	6.9	8.2
8th	9.5	12.2
9th	14.0	16.1
10th	28.0	25.5

nly

For peer review only - http://bmjopen.bmj.com/site/about/guidelines.xhtml

Table 3. Multilevel modelling of 30-day mortality in 43,247 patients with a first-ever acute myocardial infarction (AMI) in 2008-2009 in Sweden. Model 1 includes only a random effect for the hospital, model 2 is a cross-classified multilevel analysis including both hospital and risk score (RS) categories as random effects.

		Model 1	Model 2
Overall 30-day mortality mean (min – max), %		8.00 (6.23 –11.67)	4.88 (3.58 –7.59)
Variance			
	Hospital	0.04 (0.02 – 0.05)	0.03 (0.02 – 0.05)
	RS category		2.05 (0.87 – 6.49)
VPC (%)			
	Hospital	1.06(0.60 – 1.60)	0.93 (0.53 – 1.53)
	RS category		38.44 (20.94–66.35)
AUC_H			
	Hospital	0.56 (0.55 – 0.57)	0.54 (0.53 – 0.55)
	RS category		0.77 (0.77 – 0.78)
	Total	0.56 (0.55 – 0.57)	0.78 (0.77 – 0.79)

VPC: Variance Partition Coefficient, AUC: Area Under the Receiver Operating Characteristics Curve.

For peer review only

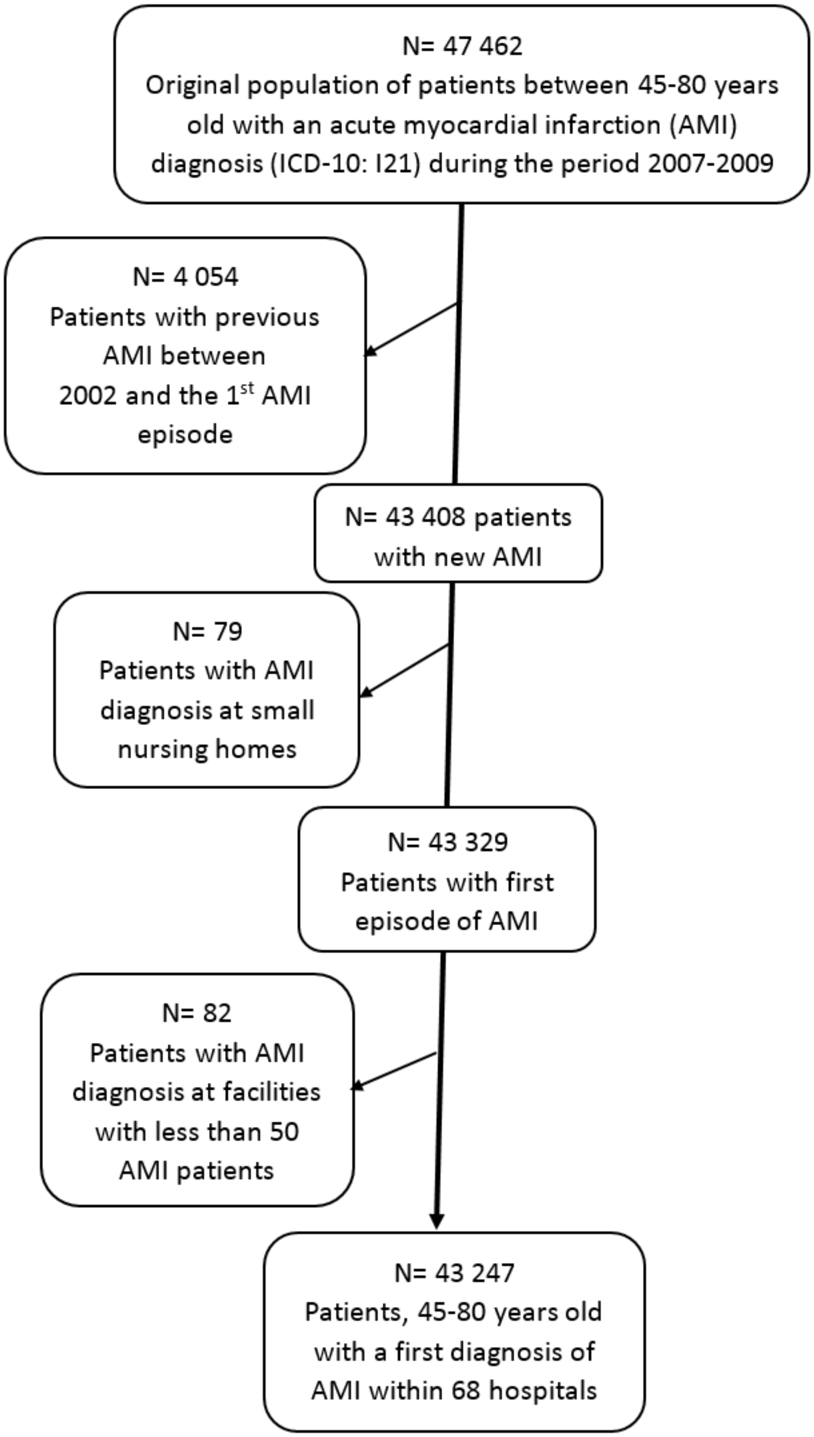
N= 47 462
Original population of patients between 45-80 years old with an acute myocardial infarction (AMI) diagnosis (ICD-10: I21) during the period 2007-2009
N= 4 054
Patients with previous AMI between 2002 and the 1st AMI episode
N= 43 408 patients with new AMI
N= 79
Patients with AMI diagnosis at small nursing homes
N= 43 329
Patients with first episode of AMI
N= 82
Patients with AMI diagnosis at facilities with less than 50 AMI patients
N= 43 247
Patients, 45-80 years old with a first diagnosis of AMI within 68 hospitals

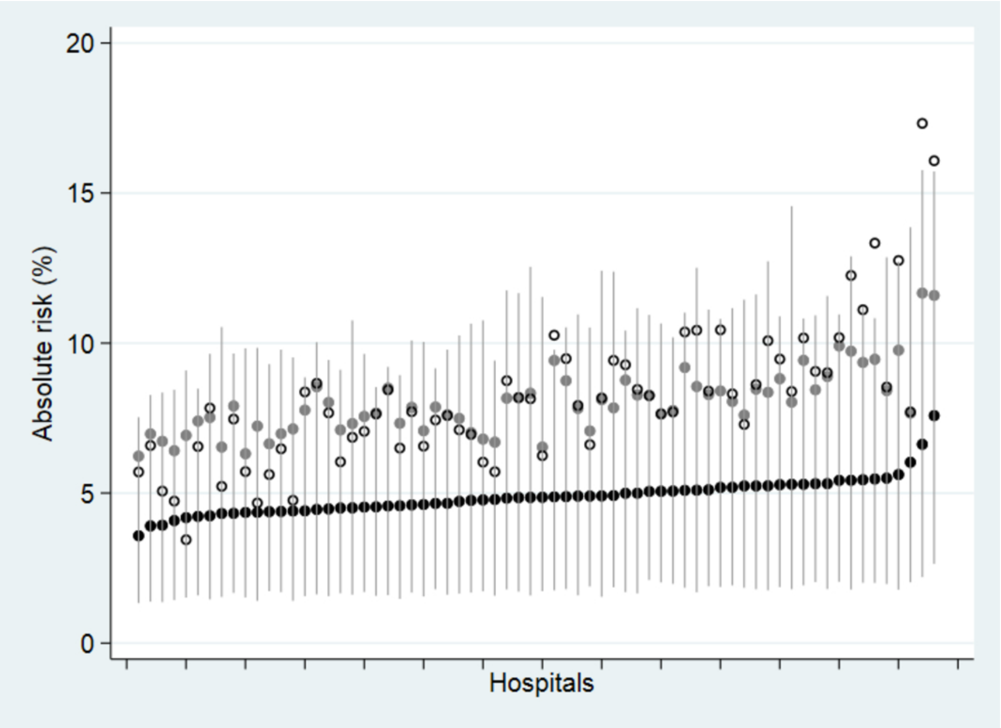

Crude (white circles), reliability weighted (grey circles) and both reliability weighted and patient-mix adjusted (black circles and confidence intervals) differences in absolute risk of 30-day mortality between hospitals in the population of 43,247 patients with a first-ever acute myocardial infarction in 2007-2009, in Sweden.

90x65mm (300 x 300 DPI)

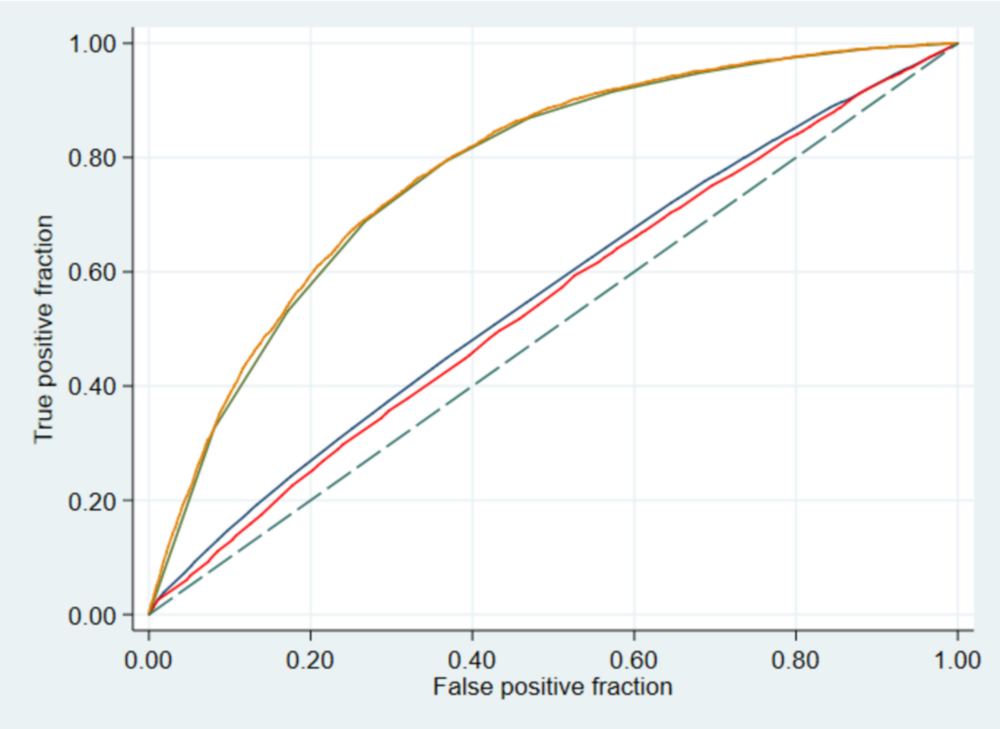

Area under the Receiver Operating Characteristics Curve (AUC) constructed using the predicted probability of 30-day mortality obtained from multilevel logistic regression analysis with 47,462 patients with a discharge diagnosis of acute myocardial infarction (AMI) in 2007-2009 in Sweden. The AUC for the unadjusted hospitals was obtained from model 1 with patient nested within hospitals (Blue line). The rest of the curves are from model 2, a cross-classified multilevel model with patients nested within hospitals and categories (decile groups) of risk-score (RS) for 30-day mortality. (Red line: patient mix adjusted hospitals, Orange line: Risk score category, Green line: Total AUC)

90x65mm (300 x 300 DPI)

hospital_new	risk10	stratum	proportion	numerator	denom	risk10_1	risk10_2	risk10_3	risk10_4	risk10_5	risk10_6	risk10_7	risk10_8	risk10_9	risk10_10
1	1	1	0	0	99	1	0	0	0	0	0	0	0	0	0
1	2	2	0,0132	1	76	0	1	0	0	0	0	0	0	0	0
1	3	3	0,0274	2	73	0	0	1	0	0	0	0	0	0	0
1	4	4	0,0161	1	62	0	0	0	1	0	0	0	0	0	0
1	5	5	0,0133	1	75	0	0	0	0	1	0	0	0	0	0
1	6	6	0,046	4	87	0	0	0	0	0	1	0	0	0	0
1	7	7	0,0741	8	108	0	0	0	0	0	0	1	0	0	0
1	8	8	0,0625	7	112	0	0	0	0	0	0	0	1	0	0
1	9	9	0,0932	11	118	0	0	0	0	0	0	0	0	1	0
1	10	10	0,2241	26	116	0	0	0	0	0	0	0	0	0	1
2	1	11	0	0	196	1	0	0	0	0	0	0	0	0	0
2	2	12	0,0056	1	179	0	1	0	0	0	0	0	0	0	0
2	3	13	0,0065	1	154	0	0	1	0	0	0	0	0	0	0
2	4	14	0,0294	4	136	0	0	0	1	0	0	0	0	0	0
2	5	15	0,0443	7	158	0	0	0	0	1	0	0	0	0	0
2	6	16	0,044	7	159	0	0	0	0	0	1	0	0	0	0
2	7	17	0,0592	9	152	0	0	0	0	0	0	1	0	0	0
2	8	18	0,1429	27	189	0	0	0	0	0	0	0	1	0	0
2	9	19	0,1762	34	193	0	0	0	0	0	0	0	0	1	0
2	10	20	0,2154	42	195	0	0	0	0	0	0	0	0	0	1
3	1	21	0,012	3	249	1	0	0	0	0	0	0	0	0	0
3	2	22	0,0212	4	189	0	1	0	0	0	0	0	0	0	0
3	3	23	0,0174	3	172	0	0	1	0	0	0	0	0	0	0
3	4	24	0,0376	7	186	0	0	0	1	0	0	0	0	0	0
3	5	25	0,0671	10	149	0	0	0	0	1	0	0	0	0	0
3	6	26	0,0663	11	166	0	0	0	0	0	1	0	0	0	0
3	7	27	0,093	12	129	0	0	0	0	0	0	1	0	0	0
3	8	28	0,1563	20	128	0	0	0	0	0	0	0	1	0	0
3	9	29	0,2234	21	94	0	0	0	0	0	0	0	0	1	0
3	10	30	0,3053	29	95	0	0	0	0	0	0	0	0	0	1
4	1	31	0,0101	1	99	1	0	0	0	0	0	0	0	0	0
4	2	32	0,0145	1	69	0	1	0	0	0	0	0	0	0	0
4	3	33	0,0263	2	76	0	0	1	0	0	0	0	0	0	0
4	4	34	0,0417	3	72	0	0	0	1	0	0	0	0	0	0
4	5	35	0,0513	4	78	0	0	0	0	1	0	0	0	0	0
4	6	36	0,1023	9	88	0	0	0	0	0	1	0	0	0	0
4	7	37	0,0761	7	92	0	0	0	0	0	0	1	0	0	0
4	8	38	0,1163	10	86	0	0	0	0	0	0	0	1	0	0
4	9	39	0,2088	19	91	0	0	0	0	0	0	0	0	1	0
4	10	40	0,2049	25	122	0	0	0	0	0	0	0	0	0	1
5	1	41	0	0	127	1	0	0	0	0	0	0	0	0	0
5	2	42	0	0	97	0	1	0	0	0	0	0	0	0	0
5	3	43	0,0177	2	113	0	0	1	0	0	0	0	0	0	0
5	4	44	0	0	100	0	0	0	1	0	0	0	0	0	0
5	5	45	0	0	93	0	0	0	0	1	0	0	0	0	0
5	6	46	0,049	5	102	0	0	0	0	0	1	0	0	0	0
5	7	47	0,0545	6	110	0	0	0	0	0	0	1	0	0	0
5	8	48	0,0621	9	145	0	0	0	0	0	0	0	1	0	0
5	9	49	0,0714	10	140	0	0	0	0	0	0	0	0	1	0
5	10	50	0,2381	35	147	0	0	0	0	0	0	0	0	0	1

For peer review only

6	1	51	0	0	26	1	0	0	0	0	0	0	0	0	0
6	2	52	0	0	22	0	1	0	0	0	0	0	0	0	0
6	3	53	0,0333	1	30	0	0	1	0	0	0	0	0	0	0
6	4	54	0,0541	2	37	0	0	0	1	0	0	0	0	0	0
6	5	55	0,0909	2	22	0	0	0	0	1	0	0	0	0	0
6	6	56	0,0541	2	37	0	0	0	0	0	1	0	0	0	0
6	7	57	0,0625	2	32	0	0	0	0	0	0	1	0	0	0
6	8	58	0,125	4	32	0	0	0	0	0	0	0	1	0	0
6	9	59	0,1071	3	28	0	0	0	0	0	0	0	0	1	0
6	10	60	0,2162	8	37	0	0	0	0	0	0	0	0	0	1
7	1	61	0	0	26	1	0	0	0	0	0	0	0	0	0
7	2	62	0	0	19	0	1	0	0	0	0	0	0	0	0
7	3	63	0,0556	1	18	0	0	1	0	0	0	0	0	0	0
7	4	64	0	0	23	0	0	0	1	0	0	0	0	0	0
7	5	65	0,1154	3	26	0	0	0	0	1	0	0	0	0	0
7	6	66	0,0741	2	27	0	0	0	0	0	1	0	0	0	0
7	7	67	0	0	26	0	0	0	0	0	0	1	0	0	0
7	8	68	0,1351	5	37	0	0	0	0	0	0	0	1	0	0
7	9	69	0,2778	10	36	0	0	0	0	0	0	0	0	1	0
7	10	70	0,2188	7	32	0	0	0	0	0	0	0	0	0	1
8	1	71	0	0	157	1	0	0	0	0	0	0	0	0	0
8	2	72	0,024	4	167	0	1	0	0	0	0	0	0	0	0
8	3	73	0,0068	1	147	0	0	1	0	0	0	0	0	0	0
8	4	74	0,0451	6	133	0	0	0	1	0	0	0	0	0	0
8	5	75	0,037	5	135	0	0	0	0	1	0	0	0	0	0
8	6	76	0,0732	9	123	0	0	0	0	0	1	0	0	0	0
8	7	77	0,0325	5	154	0	0	0	0	0	0	1	0	0	0
8	8	78	0,127	16	126	0	0	0	0	0	0	0	1	0	0
8	9	79	0,0863	12	139	0	0	0	0	0	0	0	0	1	0
8	10	80	0,2932	39	133	0	0	0	0	0	0	0	0	0	1
9	1	81	0	0	27	1	0	0	0	0	0	0	0	0	0
9	2	82	0	0	23	0	1	0	0	0	0	0	0	0	0
9	3	83	0	0	14	0	0	1	0	0	0	0	0	0	0
9	4	84	0	0	7	0	0	0	1	0	0	0	0	0	0
9	5	85	0,0714	1	14	0	0	0	0	1	0	0	0	0	0
9	6	86	0	0	15	0	0	0	0	0	1	0	0	0	0
9	7	87	0	0	7	0	0	0	0	0	0	1	0	0	0
9	8	88	0,0769	1	13	0	0	0	0	0	0	0	1	0	0
9	9	89	0	0	11	0	0	0	0	0	0	0	0	1	0
9	10	90	0,2143	3	14	0	0	0	0	0	0	0	0	0	1
10	1	91	0	0	62	1	0	0	0	0	0	0	0	0	0
10	2	92	0,0323	3	93	0	1	0	0	0	0	0	0	0	0
10	3	93	0,0119	1	84	0	0	1	0	0	0	0	0	0	0
10	4	94	0,0137	1	73	0	0	0	1	0	0	0	0	0	0
10	5	95	0,0385	3	78	0	0	0	0	1	0	0	0	0	0
10	6	96	0,0667	6	90	0	0	0	0	0	1	0	0	0	0
10	7	97	0,069	6	87	0	0	0	0	0	0	1	0	0	0
10	8	98	0,2258	14	62	0	0	0	0	0	0	0	1	0	0
10	9	99	0,1594	11	69	0	0	0	0	0	0	0	0	1	0
10	10	100	0,3396	18	53	0	0	0	0	0	0	0	0	0	1
11	1	101	0,0417	1	24	1	0	0	0	0	0	0	0	0	0
11	2	102	0,0357	1	28	0	1	0	0	0	0	0	0	0	0

For peer review only

11	3	103	0,027	1	37	0	0	1	0	0	0	0	0	0	0
11	4	104	0	0	50	0	0	0	1	0	0	0	0	0	0
11	5	105	0,027	1	37	0	0	0	0	1	0	0	0	0	0
11	6	106	0,0714	2	28	0	0	0	0	0	1	0	0	0	0
11	7	107	0,0789	3	38	0	0	0	0	0	0	1	0	0	0
11	8	108	0,0645	2	31	0	0	0	0	0	0	0	1	0	0
11	9	109	0,037	1	27	0	0	0	0	0	0	0	0	1	0
11	10	110	0,4	6	15	0	0	0	0	0	0	0	0	0	1
12	1	111	0	0	10	1	0	0	0	0	0	0	0	0	0
12	2	112	0	0	19	0	1	0	0	0	0	0	0	0	0
12	3	113	0	0	13	0	0	1	0	0	0	0	0	0	0
12	4	114	0	0	28	0	0	0	1	0	0	0	0	0	0
12	5	115	0	0	14	0	0	0	0	1	0	0	0	0	0
12	6	116	0,0714	2	28	0	0	0	0	0	1	0	0	0	0
12	7	117	0,1429	3	21	0	0	0	0	0	0	1	0	0	0
12	8	118	0	0	18	0	0	0	0	0	0	0	1	0	0
12	9	119	0,2353	4	17	0	0	0	0	0	0	0	0	1	0
12	10	120	0,1429	2	14	0	0	0	0	0	0	0	0	0	1
13	1	121	0	0	135	1	0	0	0	0	0	0	0	0	0
13	2	122	0,0073	1	137	0	1	0	0	0	0	0	0	0	0
13	3	123	0,0355	5	141	0	0	1	0	0	0	0	0	0	0
13	4	124	0,015	2	133	0	0	0	1	0	0	0	0	0	0
13	5	125	0,0147	2	136	0	0	0	0	1	0	0	0	0	0
13	6	126	0,0373	5	134	0	0	0	0	0	1	0	0	0	0
13	7	127	0,096	12	125	0	0	0	0	0	0	1	0	0	0
13	8	128	0,0851	8	94	0	0	0	0	0	0	0	1	0	0
13	9	129	0,1386	14	101	0	0	0	0	0	0	0	0	1	0
13	10	130	0,2386	21	88	0	0	0	0	0	0	0	0	0	1
14	1	131	0,0222	1	45	1	0	0	0	0	0	0	0	0	0
14	2	132	0	0	44	0	1	0	0	0	0	0	0	0	0
14	3	133	0	0	42	0	0	1	0	0	0	0	0	0	0
14	4	134	0,0294	2	68	0	0	0	1	0	0	0	0	0	0
14	5	135	0,0441	3	68	0	0	0	0	1	0	0	0	0	0
14	6	136	0,0577	3	52	0	0	0	0	0	1	0	0	0	0
14	7	137	0,069	4	58	0	0	0	0	0	0	1	0	0	0
14	8	138	0,1613	10	62	0	0	0	0	0	0	0	1	0	0
14	9	139	0,1967	12	61	0	0	0	0	0	0	0	0	1	0
14	10	140	0,3	24	80	0	0	0	0	0	0	0	0	0	1
15	1	141	0,0313	1	32	1	0	0	0	0	0	0	0	0	0
15	2	142	0	0	17	0	1	0	0	0	0	0	0	0	0
15	3	143	0	0	35	0	0	1	0	0	0	0	0	0	0
15	4	144	0,08	2	25	0	0	0	1	0	0	0	0	0	0
15	5	145	0,1071	3	28	0	0	0	0	1	0	0	0	0	0
15	6	146	0,0526	1	19	0	0	0	0	0	1	0	0	0	0
15	7	147	0,04	1	25	0	0	0	0	0	0	1	0	0	0
15	8	148	0,1739	4	23	0	0	0	0	0	0	0	1	0	0
15	9	149	0,2273	5	22	0	0	0	0	0	0	0	0	1	0
15	10	150	0,1667	4	24	0	0	0	0	0	0	0	0	0	1
16	1	151	0	0	74	1	0	0	0	0	0	0	0	0	0
16	2	152	0,0106	1	94	0	1	0	0	0	0	0	0	0	0
16	3	153	0	0	85	0	0	1	0	0	0	0	0	0	0
16	4	154	0	0	100	0	0	0	1	0	0	0	0	0	0

For peer review only

16	5	155	0,033	3	91	0	0	0	0	1	0	0	0	0	0
16	6	156	0,122	10	82	0	0	0	0	0	1	0	0	0	0
16	7	157	0,0645	6	93	0	0	0	0	0	0	1	0	0	0
16	8	158	0,1047	9	86	0	0	0	0	0	0	0	1	0	0
16	9	159	0,1563	10	64	0	0	0	0	0	0	0	0	1	0
16	10	160	0,2	16	80	0	0	0	0	0	0	0	0	0	1
17	1	161	0	0	18	1	0	0	0	0	0	0	0	0	0
17	2	162	0,0286	1	35	0	1	0	0	0	0	0	0	0	0
17	3	163	0	0	28	0	0	1	0	0	0	0	0	0	0
17	4	164	0,0588	2	34	0	0	0	1	0	0	0	0	0	0
17	5	165	0,0732	3	41	0	0	0	0	1	0	0	0	0	0
17	6	166	0,1053	4	38	0	0	0	0	0	1	0	0	0	0
17	7	167	0,0513	2	39	0	0	0	0	0	0	1	0	0	0
17	8	168	0,093	4	43	0	0	0	0	0	0	0	1	0	0
17	9	169	0,1379	4	29	0	0	0	0	0	0	0	0	1	0
17	10	170	0,3636	12	33	0	0	0	0	0	0	0	0	0	1
18	1	171	0,0741	2	27	1	0	0	0	0	0	0	0	0	0
18	2	172	0,0435	1	23	0	1	0	0	0	0	0	0	0	0
18	3	173	0	0	24	0	0	1	0	0	0	0	0	0	0
18	4	174	0	0	26	0	0	0	1	0	0	0	0	0	0
18	5	175	0	0	30	0	0	0	0	1	0	0	0	0	0
18	6	176	0,0769	2	26	0	0	0	0	0	1	0	0	0	0
18	7	177	0,0741	2	27	0	0	0	0	0	0	1	0	0	0
18	8	178	0,0588	2	34	0	0	0	0	0	0	0	1	0	0
18	9	179	0,0769	3	39	0	0	0	0	0	0	0	0	1	0
18	10	180	0,075	3	40	0	0	0	0	0	0	0	0	0	1
19	1	181	0	0	34	1	0	0	0	0	0	0	0	0	0
19	2	182	0	0	40	0	1	0	0	0	0	0	0	0	0
19	3	183	0	0	38	0	0	1	0	0	0	0	0	0	0
19	4	184	0,0769	3	39	0	0	0	1	0	0	0	0	0	0
19	5	185	0	0	45	0	0	0	0	1	0	0	0	0	0
19	6	186	0	0	37	0	0	0	0	0	1	0	0	0	0
19	7	187	0,0755	4	53	0	0	0	0	0	0	1	0	0	0
19	8	188	0,1071	6	56	0	0	0	0	0	0	0	1	0	0
19	9	189	0,15	9	60	0	0	0	0	0	0	0	0	1	0
19	10	190	0,2407	13	54	0	0	0	0	0	0	0	0	0	1
20	1	191	0	0	20	1	0	0	0	0	0	0	0	0	0
20	2	192	0,0556	1	18	0	1	0	0	0	0	0	0	0	0
20	3	193	0	0	13	0	0	1	0	0	0	0	0	0	0
20	4	194	0,05	1	20	0	0	0	1	0	0	0	0	0	0
20	5	195	0,05	1	20	0	0	0	0	1	0	0	0	0	0
20	6	196	0,0435	1	23	0	0	0	0	0	1	0	0	0	0
20	7	197	0,04	1	25	0	0	0	0	0	0	1	0	0	0
20	8	198	0,16	4	25	0	0	0	0	0	0	0	1	0	0
20	9	199	0,2	4	20	0	0	0	0	0	0	0	0	1	0
20	10	200	0,2353	4	17	0	0	0	0	0	0	0	0	0	1
21	1	201	0	0	21	1	0	0	0	0	0	0	0	0	0
21	2	202	0	0	20	0	1	0	0	0	0	0	0	0	0
21	3	203	0	0	18	0	0	1	0	0	0	0	0	0	0
21	4	204	0	0	25	0	0	0	1	0	0	0	0	0	0
21	5	205	0,069	2	29	0	0	0	0	1	0	0	0	0	0
21	6	206	0,0667	2	30	0	0	0	0	0	1	0	0	0	0

For peer review only

21	7	207	0,1364	3	22	0	0	0	0	0	0	1	0	0	0
21	8	208	0,1071	3	28	0	0	0	0	0	0	0	1	0	0
21	9	209	0,2759	8	29	0	0	0	0	0	0	0	0	1	0
21	10	210	0,2692	7	26	0	0	0	0	0	0	0	0	0	1
22	1	211	0	0	85	1	0	0	0	0	0	0	0	0	0
22	2	212	0,0093	1	107	0	1	0	0	0	0	0	0	0	0
22	3	213	0,0342	4	117	0	0	1	0	0	0	0	0	0	0
22	4	214	0,0246	3	122	0	0	0	1	0	0	0	0	0	0
22	5	215	0,036	4	111	0	0	0	0	1	0	0	0	0	0
22	6	216	0,0408	4	98	0	0	0	0	0	1	0	0	0	0
22	7	217	0,1383	13	94	0	0	0	0	0	0	1	0	0	0
22	8	218	0,11	11	100	0	0	0	0	0	0	0	1	0	0
22	9	219	0,1429	13	91	0	0	0	0	0	0	0	0	1	0
22	10	220	0,2099	17	81	0	0	0	0	0	0	0	0	0	1
23	1	221	0,0588	1	17	1	0	0	0	0	0	0	0	0	0
23	2	222	0,0455	1	22	0	1	0	0	0	0	0	0	0	0
23	3	223	0	0	16	0	0	1	0	0	0	0	0	0	0
23	4	224	0,0357	1	28	0	0	0	1	0	0	0	0	0	0
23	5	225	0	0	22	0	0	0	0	1	0	0	0	0	0
23	6	226	0,0769	2	26	0	0	0	0	0	1	0	0	0	0
23	7	227	0,1579	6	38	0	0	0	0	0	0	1	0	0	0
23	8	228	0	0	23	0	0	0	0	0	0	0	1	0	0
23	9	229	0,1538	4	26	0	0	0	0	0	0	0	0	1	0
23	10	230	0,125	3	24	0	0	0	0	0	0	0	0	0	1
24	1	231	0	0	27	1	0	0	0	0	0	0	0	0	0
24	2	232	0,0789	3	38	0	1	0	0	0	0	0	0	0	0
24	3	233	0	0	34	0	0	1	0	0	0	0	0	0	0
24	4	234	0	0	28	0	0	0	1	0	0	0	0	0	0
24	5	235	0,0303	1	33	0	0	0	0	1	0	0	0	0	0
24	6	236	0,1163	5	43	0	0	0	0	0	1	0	0	0	0
24	7	237	0,0541	2	37	0	0	0	0	0	0	1	0	0	0
24	8	238	0,1282	5	39	0	0	0	0	0	0	0	1	0	0
24	9	239	0,1724	5	29	0	0	0	0	0	0	0	0	1	0
24	10	240	0,2414	7	29	0	0	0	0	0	0	0	0	0	1
25	1	241	0,0099	1	101	1	0	0	0	0	0	0	0	0	0
25	2	242	0,02	2	100	0	1	0	0	0	0	0	0	0	0
25	3	243	0,0319	3	94	0	0	1	0	0	0	0	0	0	0
25	4	244	0,0265	3	113	0	0	0	1	0	0	0	0	0	0
25	5	245	0,0734	8	109	0	0	0	0	1	0	0	0	0	0
25	6	246	0,0885	10	113	0	0	0	0	0	1	0	0	0	0
25	7	247	0,0962	10	104	0	0	0	0	0	0	1	0	0	0
25	8	248	0,1573	14	89	0	0	0	0	0	0	0	1	0	0
25	9	249	0,2118	18	85	0	0	0	0	0	0	0	0	1	0
25	10	250	0,1974	15	76	0	0	0	0	0	0	0	0	0	1
26	1	251	0	0	41	1	0	0	0	0	0	0	0	0	0
26	2	252	0	0	43	0	1	0	0	0	0	0	0	0	0
26	3	253	0	0	48	0	0	1	0	0	0	0	0	0	0
26	4	254	0	0	47	0	0	0	1	0	0	0	0	0	0
26	5	255	0,0357	2	56	0	0	0	0	1	0	0	0	0	0
26	6	256	0,0227	1	44	0	0	0	0	0	1	0	0	0	0
26	7	257	0,0377	2	53	0	0	0	0	0	0	1	0	0	0
26	8	258	0,0877	5	57	0	0	0	0	0	0	0	1	0	0

For peer review only

26	9	259	0,1029	7	68	0	0	0	0	0	0	0	0	1	0
26	10	260	0,3165	25	79	0	0	0	0	0	0	0	0	0	1
27	1	261	0	0	9	1	0	0	0	0	0	0	0	0	0
27	2	262	0	0	17	0	1	0	0	0	0	0	0	0	0
27	3	263	0	0	17	0	0	1	0	0	0	0	0	0	0
27	4	264	0,0476	1	21	0	0	0	1	0	0	0	0	0	0
27	5	265	0	0	18	0	0	0	0	1	0	0	0	0	0
27	6	266	0	0	20	0	0	0	0	0	1	0	0	0	0
27	7	267	0,0714	1	14	0	0	0	0	0	0	1	0	0	0
27	8	268	0	0	26	0	0	0	0	0	0	0	1	0	0
27	9	269	0,1429	5	35	0	0	0	0	0	0	0	0	1	0
27	10	270	0,2895	11	38	0	0	0	0	0	0	0	0	0	1
28	1	271	0,0625	1	16	1	0	0	0	0	0	0	0	0	0
28	2	272	0	0	20	0	1	0	0	0	0	0	0	0	0
28	3	273	0	0	17	0	0	1	0	0	0	0	0	0	0
28	4	274	0	0	19	0	0	0	1	0	0	0	0	0	0
28	5	275	0	0	33	0	0	0	0	1	0	0	0	0	0
28	6	276	0,0625	1	16	0	0	0	0	0	1	0	0	0	0
28	7	277	0,1176	2	17	0	0	0	0	0	0	1	0	0	0
28	8	278	0,087	2	23	0	0	0	0	0	0	0	1	0	0
28	9	279	0,2059	7	34	0	0	0	0	0	0	0	0	1	0
28	10	280	0,1087	5	46	0	0	0	0	0	0	0	0	0	1
29	1	281	0	0	145	1	0	0	0	0	0	0	0	0	0
29	2	282	0,0063	1	159	0	1	0	0	0	0	0	0	0	0
29	3	283	0,015	2	133	0	0	1	0	0	0	0	0	0	0
29	4	284	0,0263	3	114	0	0	0	1	0	0	0	0	0	0
29	5	285	0,0462	6	130	0	0	0	0	1	0	0	0	0	0
29	6	286	0,0493	7	142	0	0	0	0	0	1	0	0	0	0
29	7	287	0,0897	13	145	0	0	0	0	0	0	1	0	0	0
29	8	288	0,1126	17	151	0	0	0	0	0	0	0	1	0	0
29	9	289	0,1056	17	161	0	0	0	0	0	0	0	0	1	0
29	10	290	0,2588	44	170	0	0	0	0	0	0	0	0	0	1
30	1	291	0,0041	1	245	1	0	0	0	0	0	0	0	0	0
30	2	292	0,0225	5	222	0	1	0	0	0	0	0	0	0	0
30	3	293	0,0314	7	223	0	0	1	0	0	0	0	0	0	0
30	4	294	0,0229	5	218	0	0	0	1	0	0	0	0	0	0
30	5	295	0,025	5	200	0	0	0	0	1	0	0	0	0	0
30	6	296	0,0705	16	227	0	0	0	0	0	1	0	0	0	0
30	7	297	0,0783	13	166	0	0	0	0	0	0	1	0	0	0
30	8	298	0,1588	27	170	0	0	0	0	0	0	0	1	0	0
30	9	299	0,1642	33	201	0	0	0	0	0	0	0	0	1	0
30	10	300	0,2604	44	169	0	0	0	0	0	0	0	0	0	1
31	1	301	0	0	30	1	0	0	0	0	0	0	0	0	0
31	2	302	0,0588	1	17	0	1	0	0	0	0	0	0	0	0
31	3	303	0	0	23	0	0	1	0	0	0	0	0	0	0
31	4	304	0	0	13	0	0	0	1	0	0	0	0	0	0
31	5	305	0	0	15	0	0	0	0	1	0	0	0	0	0
31	6	306	0	0	16	0	0	0	0	0	1	0	0	0	0
31	7	307	0,0833	1	12	0	0	0	0	0	0	1	0	0	0
31	8	308	0,1667	4	24	0	0	0	0	0	0	0	1	0	0
31	9	309	0,125	2	16	0	0	0	0	0	0	0	0	1	0
31	10	310	0,4	10	25	0	0	0	0	0	0	0	0	0	1

For peer review only

32	1	311	0,0105	1	95	1	0	0	0	0	0	0	0	0	0
32	2	312	0	0	86	0	1	0	0	0	0	0	0	0	0
32	3	313	0,0135	1	74	0	0	1	0	0	0	0	0	0	0
32	4	314	0,0328	2	61	0	0	0	1	0	0	0	0	0	0
32	5	315	0,0308	2	65	0	0	0	0	1	0	0	0	0	0
32	6	316	0,0759	6	79	0	0	0	0	0	1	0	0	0	0
32	7	317	0,0423	3	71	0	0	0	0	0	0	1	0	0	0
32	8	318	0,1282	10	78	0	0	0	0	0	0	0	1	0	0
32	9	319	0,1444	13	90	0	0	0	0	0	0	0	0	1	0
32	10	320	0,3133	26	83	0	0	0	0	0	0	0	0	0	1
33	1	321	0	0	39	1	0	0	0	0	0	0	0	0	0
33	2	322	0	0	19	0	1	0	0	0	0	0	0	0	0
33	3	323	0	0	38	0	0	1	0	0	0	0	0	0	0
33	4	324	0	0	39	0	0	0	1	0	0	0	0	0	0
33	5	325	0,0189	1	53	0	0	0	0	1	0	0	0	0	0
33	6	326	0,0238	1	42	0	0	0	0	0	1	0	0	0	0
33	7	327	0,1087	5	46	0	0	0	0	0	0	1	0	0	0
33	8	328	0,0877	5	57	0	0	0	0	0	0	0	1	0	0
33	9	329	0,1571	11	70	0	0	0	0	0	0	0	0	1	0
33	10	330	0,2317	19	82	0	0	0	0	0	0	0	0	0	1
34	1	331	0,0164	1	61	1	0	0	0	0	0	0	0	0	0
34	2	332	0,0204	1	49	0	1	0	0	0	0	0	0	0	0
34	3	333	0	0	35	0	0	1	0	0	0	0	0	0	0
34	4	334	0,0351	2	57	0	0	0	1	0	0	0	0	0	0
34	5	335	0,0192	1	52	0	0	0	0	1	0	0	0	0	0
34	6	336	0,05	3	60	0	0	0	0	0	1	0	0	0	0
34	7	337	0,0933	7	75	0	0	0	0	0	0	1	0	0	0
34	8	338	0,119	10	84	0	0	0	0	0	0	0	1	0	0
34	9	339	0,1304	12	92	0	0	0	0	0	0	0	0	1	0
34	10	340	0,2821	33	117	0	0	0	0	0	0	0	0	0	1
35	1	341	0	0	26	1	0	0	0	0	0	0	0	0	0
35	2	342	0,0278	1	36	0	1	0	0	0	0	0	0	0	0
35	3	343	0,0789	3	38	0	0	1	0	0	0	0	0	0	0
35	4	344	0	0	34	0	0	0	1	0	0	0	0	0	0
35	5	345	0,0345	1	29	0	0	0	0	1	0	0	0	0	0
35	6	346	0,0769	3	39	0	0	0	0	0	1	0	0	0	0
35	7	347	0,0952	4	42	0	0	0	0	0	0	1	0	0	0
35	8	348	0,0889	4	45	0	0	0	0	0	0	0	1	0	0
35	9	349	0,2368	9	38	0	0	0	0	0	0	0	0	1	0
35	10	350	0,3333	17	51	0	0	0	0	0	0	0	0	0	1
36	1	351	0,006	2	331	1	0	0	0	0	0	0	0	0	0
36	2	352	0,0095	3	316	0	1	0	0	0	0	0	0	0	0
36	3	353	0,0188	6	319	0	0	1	0	0	0	0	0	0	0
36	4	354	0,0262	8	305	0	0	0	1	0	0	0	0	0	0
36	5	355	0,028	9	321	0	0	0	0	1	0	0	0	0	0
36	6	356	0,0556	17	306	0	0	0	0	0	1	0	0	0	0
36	7	357	0,0846	27	319	0	0	0	0	0	0	1	0	0	0
36	8	358	0,132	40	303	0	0	0	0	0	0	0	1	0	0
36	9	359	0,2109	54	256	0	0	0	0	0	0	0	0	1	0
36	10	360	0,2605	68	261	0	0	0	0	0	0	0	0	0	1
37	1	361	0,0385	1	26	1	0	0	0	0	0	0	0	0	0
37	2	362	0	0	30	0	1	0	0	0	0	0	0	0	0

For peer review only

37	3	363	0,0278	1	36	0	0	1	0	0	0	0	0	0	0
37	4	364	0	0	35	0	0	0	1	0	0	0	0	0	0
37	5	365	0,0233	1	43	0	0	0	0	1	0	0	0	0	0
37	6	366	0,0667	2	30	0	0	0	0	0	1	0	0	0	0
37	7	367	0,0513	2	39	0	0	0	0	0	0	1	0	0	0
37	8	368	0,1351	5	37	0	0	0	0	0	0	0	1	0	0
37	9	369	0,1277	6	47	0	0	0	0	0	0	0	0	1	0
37	10	370	0,25	14	56	0	0	0	0	0	0	0	0	0	1
38	1	371	0	0	96	1	0	0	0	0	0	0	0	0	0
38	2	372	0,0227	2	88	0	1	0	0	0	0	0	0	0	0
38	3	373	0,0206	2	97	0	0	1	0	0	0	0	0	0	0
38	4	374	0,0208	2	96	0	0	0	1	0	0	0	0	0	0
38	5	375	0,0708	8	113	0	0	0	0	1	0	0	0	0	0
38	6	376	0,0654	7	107	0	0	0	0	0	1	0	0	0	0
38	7	377	0,1008	12	119	0	0	0	0	0	0	1	0	0	0
38	8	378	0,1613	20	124	0	0	0	0	0	0	0	1	0	0
38	9	379	0,1788	27	151	0	0	0	0	0	0	0	0	1	0
38	10	380	0,2432	36	148	0	0	0	0	0	0	0	0	0	1
39	1	381	0,0128	1	78	1	0	0	0	0	0	0	0	0	0
39	2	382	0,023	2	87	0	1	0	0	0	0	0	0	0	0
39	3	383	0,0125	1	80	0	0	1	0	0	0	0	0	0	0
39	4	384	0,0411	3	73	0	0	0	1	0	0	0	0	0	0
39	5	385	0,0337	3	89	0	0	0	0	1	0	0	0	0	0
39	6	386	0,0337	3	89	0	0	0	0	0	1	0	0	0	0
39	7	387	0,125	8	64	0	0	0	0	0	0	1	0	0	0
39	8	388	0,1531	15	98	0	0	0	0	0	0	0	1	0	0
39	9	389	0,1591	14	88	0	0	0	0	0	0	0	0	1	0
39	10	390	0,32	24	75	0	0	0	0	0	0	0	0	0	1
40	1	391	0	0	12	1	0	0	0	0	0	0	0	0	0
40	2	392	0	0	18	0	1	0	0	0	0	0	0	0	0
40	3	393	0	0	12	0	0	1	0	0	0	0	0	0	0
40	4	394	0	0	15	0	0	0	1	0	0	0	0	0	0
40	5	395	0,0313	1	32	0	0	0	0	1	0	0	0	0	0
40	6	396	0,0556	1	18	0	0	0	0	0	1	0	0	0	0
40	7	397	0	0	18	0	0	0	0	0	0	1	0	0	0
40	8	398	0,2381	5	21	0	0	0	0	0	0	0	1	0	0
40	9	399	0,3182	7	22	0	0	0	0	0	0	0	0	1	0
40	10	400	0,3929	11	28	0	0	0	0	0	0	0	0	0	1
41	1	401	0,0109	1	92	1	0	0	0	0	0	0	0	0	0
41	2	402	0,0156	2	128	0	1	0	0	0	0	0	0	0	0
41	3	403	0,0263	3	114	0	0	1	0	0	0	0	0	0	0
41	4	404	0,0071	1	141	0	0	0	1	0	0	0	0	0	0
41	5	405	0,0565	7	124	0	0	0	0	1	0	0	0	0	0
41	6	406	0,1069	14	131	0	0	0	0	0	1	0	0	0	0
41	7	407	0,1014	14	138	0	0	0	0	0	0	1	0	0	0
41	8	408	0,1074	16	149	0	0	0	0	0	0	0	1	0	0
41	9	409	0,1523	23	151	0	0	0	0	0	0	0	0	1	0
41	10	410	0,2051	24	117	0	0	0	0	0	0	0	0	0	1
42	1	411	0	0	101	1	0	0	0	0	0	0	0	0	0
42	2	412	0	0	108	0	1	0	0	0	0	0	0	0	0
42	3	413	0,0137	2	146	0	0	1	0	0	0	0	0	0	0
42	4	414	0,0458	6	131	0	0	0	1	0	0	0	0	0	0

For peer review only

42	5	415	0,0692	9	130	0	0	0	0	1	0	0	0	0	0
42	6	416	0,0488	6	123	0	0	0	0	0	1	0	0	0	0
42	7	417	0,1284	14	109	0	0	0	0	0	0	1	0	0	0
42	8	418	0,0704	5	71	0	0	0	0	0	0	0	1	0	0
42	9	419	0,1412	12	85	0	0	0	0	0	0	0	0	1	0
42	10	420	0,2464	17	69	0	0	0	0	0	0	0	0	0	1
43	1	421	0	0	17	1	0	0	0	0	0	0	0	0	0
43	2	422	0	0	26	0	1	0	0	0	0	0	0	0	0
43	3	423	0	0	24	0	0	1	0	0	0	0	0	0	0
43	4	424	0,0714	1	14	0	0	0	1	0	0	0	0	0	0
43	5	425	0	0	23	0	0	0	0	1	0	0	0	0	0
43	6	426	0	0	17	0	0	0	0	0	1	0	0	0	0
43	7	427	0,0435	1	23	0	0	0	0	0	0	1	0	0	0
43	8	428	0,3	6	20	0	0	0	0	0	0	0	1	0	0
43	9	429	0,15	3	20	0	0	0	0	0	0	0	0	1	0
43	10	430	0,2292	11	48	0	0	0	0	0	0	0	0	0	1
44	1	431	0	0	11	1	0	0	0	0	0	0	0	0	0
44	2	432	0	0	18	0	1	0	0	0	0	0	0	0	0
44	3	433	0	0	14	0	0	1	0	0	0	0	0	0	0
44	4	434	0	0	7	0	0	0	1	0	0	0	0	0	0
44	5	435	0	0	14	0	0	0	0	1	0	0	0	0	0
44	6	436	0	0	17	0	0	0	0	0	1	0	0	0	0
44	7	437	0,1429	3	21	0	0	0	0	0	0	1	0	0	0
44	8	438	0,2222	4	18	0	0	0	0	0	0	0	1	0	0
44	9	439	0,3913	9	23	0	0	0	0	0	0	0	0	1	0
44	10	440	0,2727	6	22	0	0	0	0	0	0	0	0	0	1
45	1	441	0,0146	2	137	1	0	0	0	0	0	0	0	0	0
45	2	442	0,0164	3	183	0	1	0	0	0	0	0	0	0	0
45	3	443	0,0355	5	141	0	0	1	0	0	0	0	0	0	0
45	4	444	0,0327	5	153	0	0	0	1	0	0	0	0	0	0
45	5	445	0,0296	4	135	0	0	0	0	1	0	0	0	0	0
45	6	446	0,0565	7	124	0	0	0	0	0	1	0	0	0	0
45	7	447	0,094	11	117	0	0	0	0	0	0	1	0	0	0
45	8	448	0,0598	7	117	0	0	0	0	0	0	0	1	0	0
45	9	449	0,1579	12	76	0	0	0	0	0	0	0	0	1	0
45	10	450	0,284	23	81	0	0	0	0	0	0	0	0	0	1
46	1	451	0	0	16	1	0	0	0	0	0	0	0	0	0
46	2	452	0	0	20	0	1	0	0	0	0	0	0	0	0
46	3	453	0,0526	1	19	0	0	1	0	0	0	0	0	0	0
46	4	454	0,0714	1	14	0	0	0	1	0	0	0	0	0	0
46	5	455	0,1071	3	28	0	0	0	0	1	0	0	0	0	0
46	6	456	0,1154	3	26	0	0	0	0	0	1	0	0	0	0
46	7	457	0,2222	6	27	0	0	0	0	0	0	1	0	0	0
46	8	458	0,1515	5	33	0	0	0	0	0	0	0	1	0	0
46	9	459	0,2414	7	29	0	0	0	0	0	0	0	0	1	0
46	10	460	0,4286	18	42	0	0	0	0	0	0	0	0	0	1
47	1	461	0	0	8	1	0	0	0	0	0	0	0	0	0
47	2	462	0,0625	1	16	0	1	0	0	0	0	0	0	0	0
47	3	463	0,1	2	20	0	0	1	0	0	0	0	0	0	0
47	4	464	0,0588	1	17	0	0	0	1	0	0	0	0	0	0
47	5	465	0	0	20	0	0	0	0	1	0	0	0	0	0
47	6	466	0,1579	3	19	0	0	0	0	0	1	0	0	0	0

For peer review only

47	7	467	0,0323	1	31	0	0	0	0	0	0	1	0	0	0
47	8	468	0,1053	2	19	0	0	0	0	0	0	0	1	0	0
47	9	469	0,2917	7	24	0	0	0	0	0	0	0	0	1	0
47	10	470	0,2667	8	30	0	0	0	0	0	0	0	0	0	1
48	1	471	0,0282	2	71	1	0	0	0	0	0	0	0	0	0
48	2	472	0	0	71	0	1	0	0	0	0	0	0	0	0
48	3	473	0	0	63	0	0	1	0	0	0	0	0	0	0
48	4	474	0,0145	1	69	0	0	0	1	0	0	0	0	0	0
48	5	475	0,0172	1	58	0	0	0	0	1	0	0	0	0	0
48	6	476	0,0556	4	72	0	0	0	0	0	1	0	0	0	0
48	7	477	0,0833	6	72	0	0	0	0	0	0	1	0	0	0
48	8	478	0,1613	15	93	0	0	0	0	0	0	0	1	0	0
48	9	479	0,131	11	84	0	0	0	0	0	0	0	0	1	0
48	10	480	0,2239	15	67	0	0	0	0	0	0	0	0	0	1
49	1	481	0	0	24	1	0	0	0	0	0	0	0	0	0
49	2	482	0	0	16	0	1	0	0	0	0	0	0	0	0
49	3	483	0	0	30	0	0	1	0	0	0	0	0	0	0
49	4	484	0	0	35	0	0	0	1	0	0	0	0	0	0
49	5	485	0,0233	1	43	0	0	0	0	1	0	0	0	0	0
49	6	486	0	0	43	0	0	0	0	0	1	0	0	0	0
49	7	487	0,0833	4	48	0	0	0	0	0	0	1	0	0	0
49	8	488	0,1163	5	43	0	0	0	0	0	0	0	1	0	0
49	9	489	0,2	7	35	0	0	0	0	0	0	0	0	1	0
49	10	490	0,1765	6	34	0	0	0	0	0	0	0	0	0	1
50	1	491	0	0	129	1	0	0	0	0	0	0	0	0	0
50	2	492	0,0278	3	108	0	1	0	0	0	0	0	0	0	0
50	3	493	0,0147	2	136	0	0	1	0	0	0	0	0	0	0
50	4	494	0,0082	1	122	0	0	0	1	0	0	0	0	0	0
50	5	495	0,0204	2	98	0	0	0	0	1	0	0	0	0	0
50	6	496	0,0505	5	99	0	0	0	0	0	1	0	0	0	0
50	7	497	0,0619	7	113	0	0	0	0	0	0	1	0	0	0
50	8	498	0,1389	10	72	0	0	0	0	0	0	0	1	0	0
50	9	499	0,1224	12	98	0	0	0	0	0	0	0	0	1	0
50	10	500	0,3043	21	69	0	0	0	0	0	0	0	0	0	1
51	1	501	0	0	21	1	0	0	0	0	0	0	0	0	0
51	2	502	0	0	25	0	1	0	0	0	0	0	0	0	0
51	3	503	0	0	35	0	0	1	0	0	0	0	0	0	0
51	4	504	0,04	1	25	0	0	0	1	0	0	0	0	0	0
51	5	505	0,0556	2	36	0	0	0	0	1	0	0	0	0	0
51	6	506	0,1163	5	43	0	0	0	0	0	1	0	0	0	0
51	7	507	0,037	1	27	0	0	0	0	0	0	1	0	0	0
51	8	508	0,1429	3	21	0	0	0	0	0	0	0	1	0	0
51	9	509	0,1778	8	45	0	0	0	0	0	0	0	0	1	0
51	10	510	0,1905	8	42	0	0	0	0	0	0	0	0	0	1
52	1	511	0	0	3	1	0	0	0	0	0	0	0	0	0
52	2	512	0	0	9	0	1	0	0	0	0	0	0	0	0
52	3	513	0	0	9	0	0	1	0	0	0	0	0	0	0
52	4	514	0	0	17	0	0	0	1	0	0	0	0	0	0
52	5	515	0	0	15	0	0	0	0	1	0	0	0	0	0
52	6	516	0	0	12	0	0	0	0	0	1	0	0	0	0
52	7	517	0,2222	2	9	0	0	0	0	0	0	1	0	0	0
52	8	518	0,0833	1	12	0	0	0	0	0	0	0	1	0	0

For peer review only

52	9	519	0	0	10	0	0	0	0	0	0	0	0	1	0
52	10	520	0,1818	2	11	0	0	0	0	0	0	0	0	0	1
53	1	521	0	0	7	1	0	0	0	0	0	0	0	0	0
53	2	522	0	0	12	0	1	0	0	0	0	0	0	0	0
53	3	523	0	0	18	0	0	1	0	0	0	0	0	0	0
53	4	524	0,0769	1	13	0	0	0	1	0	0	0	0	0	0
53	5	525	0	0	13	0	0	0	0	1	0	0	0	0	0
53	6	526	0	0	23	0	0	0	0	0	1	0	0	0	0
53	7	527	0,0833	1	12	0	0	0	0	0	0	1	0	0	0
53	8	528	0,0714	1	14	0	0	0	0	0	0	0	1	0	0
53	9	529	0,1429	3	21	0	0	0	0	0	0	0	0	1	0
53	10	530	0,0714	1	14	0	0	0	0	0	0	0	0	0	1
54	1	531	0,0309	3	97	1	0	0	0	0	0	0	0	0	0
54	2	532	0,0164	2	122	0	1	0	0	0	0	0	0	0	0
54	3	533	0,0342	4	117	0	0	1	0	0	0	0	0	0	0
54	4	534	0,0168	2	119	0	0	0	1	0	0	0	0	0	0
54	5	535	0,028	3	107	0	0	0	0	1	0	0	0	0	0
54	6	536	0,0328	4	122	0	0	0	0	0	1	0	0	0	0
54	7	537	0,1008	12	119	0	0	0	0	0	0	1	0	0	0
54	8	538	0,1089	11	101	0	0	0	0	0	0	0	1	0	0
54	9	539	0,1852	15	81	0	0	0	0	0	0	0	0	1	0
54	10	540	0,3208	34	106	0	0	0	0	0	0	0	0	0	1
55	1	541	0	0	27	1	0	0	0	0	0	0	0	0	0
55	2	542	0	0	53	0	1	0	0	0	0	0	0	0	0
55	3	543	0	0	30	0	0	1	0	0	0	0	0	0	0
55	4	544	0	0	38	0	0	0	1	0	0	0	0	0	0
55	5	545	0	0	37	0	0	0	0	1	0	0	0	0	0
55	6	546	0	0	38	0	0	0	0	0	1	0	0	0	0
55	7	547	0,0769	3	39	0	0	0	0	0	0	1	0	0	0
55	8	548	0,1034	3	29	0	0	0	0	0	0	0	1	0	0
55	9	549	0,129	4	31	0	0	0	0	0	0	0	0	1	0
55	10	550	0,1892	7	37	0	0	0	0	0	0	0	0	0	1
56	1	551	0,0213	1	47	1	0	0	0	0	0	0	0	0	0
56	2	552	0	0	38	0	1	0	0	0	0	0	0	0	0
56	3	553	0,0392	2	51	0	0	1	0	0	0	0	0	0	0
56	4	554	0,0196	1	51	0	0	0	1	0	0	0	0	0	0
56	5	555	0	0	37	0	0	0	0	1	0	0	0	0	0
56	6	556	0,0638	3	47	0	0	0	0	0	1	0	0	0	0
56	7	557	0,0638	3	47	0	0	0	0	0	0	1	0	0	0
56	8	558	0,1429	6	42	0	0	0	0	0	0	0	1	0	0
56	9	559	0,1818	8	44	0	0	0	0	0	0	0	0	1	0
56	10	560	0,2188	7	32	0	0	0	0	0	0	0	0	0	1
57	1	561	0,0145	1	69	1	0	0	0	0	0	0	0	0	0
57	2	562	0	0	73	0	1	0	0	0	0	0	0	0	0
57	3	563	0,0115	1	87	0	0	1	0	0	0	0	0	0	0
57	4	564	0,0333	3	90	0	0	0	1	0	0	0	0	0	0
57	5	565	0,0455	4	88	0	0	0	0	1	0	0	0	0	0
57	6	566	0,0541	4	74	0	0	0	0	0	1	0	0	0	0
57	7	567	0,0843	7	83	0	0	0	0	0	0	1	0	0	0
57	8	568	0,1447	11	76	0	0	0	0	0	0	0	1	0	0
57	9	569	0,1765	12	68	0	0	0	0	0	0	0	0	1	0
57	10	570	0,3086	25	81	0	0	0	0	0	0	0	0	0	1

58	1	571	0	0	22	1	0	0	0	0	0	0	0	0	0
58	2	572	0,0435	1	23	0	1	0	0	0	0	0	0	0	0
58	3	573	0	0	42	0	0	1	0	0	0	0	0	0	0
58	4	574	0,0571	2	35	0	0	0	1	0	0	0	0	0	0
58	5	575	0	0	34	0	0	0	0	1	0	0	0	0	0
58	6	576	0,0606	2	33	0	0	0	0	0	1	0	0	0	0
58	7	577	0	0	34	0	0	0	0	0	0	1	0	0	0
58	8	578	0,1667	7	42	0	0	0	0	0	0	0	1	0	0
58	9	579	0,1071	3	28	0	0	0	0	0	0	0	0	1	0
58	10	580	0,2	6	30	0	0	0	0	0	0	0	0	0	1
59	1	581	0	0	12	1	0	0	0	0	0	0	0	0	0
59	2	582	0,037	1	27	0	1	0	0	0	0	0	0	0	0
59	3	583	0,0357	1	28	0	0	1	0	0	0	0	0	0	0
59	4	584	0,0435	1	23	0	0	0	1	0	0	0	0	0	0
59	5	585	0,0385	1	26	0	0	0	0	1	0	0	0	0	0
59	6	586	0,1333	2	15	0	0	0	0	0	1	0	0	0	0
59	7	587	0,0526	1	19	0	0	0	0	0	0	1	0	0	0
59	8	588	0	0	30	0	0	0	0	0	0	0	1	0	0
59	9	589	0,2222	4	18	0	0	0	0	0	0	0	0	1	0
59	10	590	0,3043	7	23	0	0	0	0	0	0	0	0	0	1
60	1	591	0,0152	1	66	1	0	0	0	0	0	0	0	0	0
60	2	592	0,0159	1	63	0	1	0	0	0	0	0	0	0	0
60	3	593	0,0423	3	71	0	0	1	0	0	0	0	0	0	0
60	4	594	0	0	80	0	0	0	1	0	0	0	0	0	0
60	5	595	0,0161	1	62	0	0	0	0	1	0	0	0	0	0
60	6	596	0,0508	3	59	0	0	0	0	0	1	0	0	0	0
60	7	597	0,1132	6	53	0	0	0	0	0	0	1	0	0	0
60	8	598	0,0652	3	46	0	0	0	0	0	0	0	1	0	0
60	9	599	0,1818	8	44	0	0	0	0	0	0	0	0	1	0
60	10	600	0,1628	7	43	0	0	0	0	0	0	0	0	0	1
61	1	601	0,0067	1	150	1	0	0	0	0	0	0	0	0	0
61	2	602	0,0167	2	120	0	1	0	0	0	0	0	0	0	0
61	3	603	0,0336	4	119	0	0	1	0	0	0	0	0	0	0
61	4	604	0,036	4	111	0	0	0	1	0	0	0	0	0	0
61	5	605	0,022	2	91	0	0	0	0	1	0	0	0	0	0
61	6	606	0,0361	3	83	0	0	0	0	0	1	0	0	0	0
61	7	607	0,1111	10	90	0	0	0	0	0	0	1	0	0	0
61	8	608	0,1711	13	76	0	0	0	0	0	0	0	1	0	0
61	9	609	0,1237	12	97	0	0	0	0	0	0	0	0	1	0
61	10	610	0,2949	23	78	0	0	0	0	0	0	0	0	0	1
62	1	611	0	0	31	1	0	0	0	0	0	0	0	0	0
62	2	612	0,04	1	25	0	1	0	0	0	0	0	0	0	0
62	3	613	0	0	31	0	0	1	0	0	0	0	0	0	0
62	4	614	0	0	35	0	0	0	1	0	0	0	0	0	0
62	5	615	0,0556	2	36	0	0	0	0	1	0	0	0	0	0
62	6	616	0,0588	2	34	0	0	0	0	0	1	0	0	0	0
62	7	617	0,1071	3	28	0	0	0	0	0	0	1	0	0	0
62	8	618	0,0811	3	37	0	0	0	0	0	0	0	1	0	0
62	9	619	0,125	6	48	0	0	0	0	0	0	0	0	1	0
62	10	620	0,2	7	35	0	0	0	0	0	0	0	0	0	1
63	1	621	0	0	17	1	0	0	0	0	0	0	0	0	0
63	2	622	0	0	18	0	1	0	0	0	0	0	0	0	0

For peer review only

63	3	623	0,0952	2	21	0	0	1	0	0	0	0	0	0	0
63	4	624	0,0435	1	23	0	0	0	1	0	0	0	0	0	0
63	5	625	0,0385	1	26	0	0	0	0	1	0	0	0	0	0
63	6	626	0	0	20	0	0	0	0	0	1	0	0	0	0
63	7	627	0,0476	1	21	0	0	0	0	0	0	1	0	0	0
63	8	628	0,1765	3	17	0	0	0	0	0	0	0	1	0	0
63	9	629	0,1111	2	18	0	0	0	0	0	0	0	0	1	0
63	10	630	0,1765	3	17	0	0	0	0	0	0	0	0	0	1
64	1	631	0	0	31	1	0	0	0	0	0	0	0	0	0
64	2	632	0	0	25	0	1	0	0	0	0	0	0	0	0
64	3	633	0,125	4	32	0	0	1	0	0	0	0	0	0	0
64	4	634	0,15	3	20	0	0	0	1	0	0	0	0	0	0
64	5	635	0,1935	6	31	0	0	0	0	1	0	0	0	0	0
64	6	636	0,0741	2	27	0	0	0	0	0	1	0	0	0	0
64	7	637	0,16	4	25	0	0	0	0	0	0	1	0	0	0
64	8	638	0,2609	6	23	0	0	0	0	0	0	0	1	0	0
64	9	639	0,3462	9	26	0	0	0	0	0	0	0	0	1	0
64	10	640	0,4667	7	15	0	0	0	0	0	0	0	0	0	1
65	1	641	0	0	32	1	0	0	0	0	0	0	0	0	0
65	2	642	0	0	22	0	1	0	0	0	0	0	0	0	0
65	3	643	0	0	24	0	0	1	0	0	0	0	0	0	0
65	4	644	0	0	28	0	0	0	1	0	0	0	0	0	0
65	5	645	0	0	32	0	0	0	0	1	0	0	0	0	0
65	6	646	0,0455	2	44	0	0	0	0	0	1	0	0	0	0
65	7	647	0	0	29	0	0	0	0	0	0	1	0	0	0
65	8	648	0,12	3	25	0	0	0	0	0	0	0	1	0	0
65	9	649	0,2414	7	29	0	0	0	0	0	0	0	0	1	0
65	10	650	0,1364	3	22	0	0	0	0	0	0	0	0	0	1
66	1	651	0	0	18	1	0	0	0	0	0	0	0	0	0
66	2	652	0	0	30	0	1	0	0	0	0	0	0	0	0
66	3	653	0	0	16	0	0	1	0	0	0	0	0	0	0
66	4	654	0,0952	2	21	0	0	0	1	0	0	0	0	0	0
66	5	655	0	0	28	0	0	0	0	1	0	0	0	0	0
66	6	656	0,05	1	20	0	0	0	0	0	1	0	0	0	0
66	7	657	0,04	1	25	0	0	0	0	0	0	1	0	0	0
66	8	658	0,1613	5	31	0	0	0	0	0	0	0	1	0	0
66	9	659	0,2414	7	29	0	0	0	0	0	0	0	0	1	0
66	10	660	0,3226	10	31	0	0	0	0	0	0	0	0	0	1
67	1	661	0	0	17	1	0	0	0	0	0	0	0	0	0
67	2	662	0	0	12	0	1	0	0	0	0	0	0	0	0
67	3	663	0,0476	1	21	0	0	1	0	0	0	0	0	0	0
67	4	664	0	0	16	0	0	0	1	0	0	0	0	0	0
67	5	665	0	0	11	0	0	0	0	1	0	0	0	0	0
67	6	666	0,1667	2	12	0	0	0	0	0	1	0	0	0	0
67	7	667	0	0	15	0	0	0	0	0	0	1	0	0	0
67	8	668	0,1053	2	19	0	0	0	0	0	0	0	1	0	0
67	9	669	0,2105	4	19	0	0	0	0	0	0	0	0	1	0
67	10	670	0,381	8	21	0	0	0	0	0	0	0	0	0	1
68	1	671	0	0	70	1	0	0	0	0	0	0	0	0	0
68	2	672	0	0	48	0	1	0	0	0	0	0	0	0	0
68	3	673	0	0	57	0	0	1	0	0	0	0	0	0	0
68	4	674	0,0328	2	61	0	0	0	1	0	0	0	0	0	0

For peer review only

68	5	675	0,0392	2	51	0	0	0	0	1	0	0	0	0	0
68	6	676	0,0667	3	45	0	0	0	0	0	1	0	0	0	0
68	7	677	0,1273	7	55	0	0	0	0	0	0	1	0	0	0
68	8	678	0,1364	9	66	0	0	0	0	0	0	0	1	0	0
68	9	679	0,1964	11	56	0	0	0	0	0	0	0	0	1	0
68	10	680	0,3148	17	54	0	0	0	0	0	0	0	0	0	1

For peer review only

```
use AMI_short30days, replace

sort hospital_new stratum

global MLwiN_path "C:\Program Files\MLwiN v3.02\mlwin.exe"


******************

******Model 1******

******************


* Fit model 1

runmlwin proportion cons , ///

  level3 (hospital_new:cons)  ///

  level2(stratum: ) ///

  level1(stratum:) ///

  discrete(distribution(binomial) link(logit) denom(denom)) ///

  nopause


* Fit model 1  by MCMC

runmlwin proportion cons   , ///

  level3 (hospital_new:cons, residuals(u))  ///

  level2(stratum: ) ///

  level1(stratum:) ///

  discrete(distribution(binomial) link(logit) denom (denom)) ///

  mcmc(burnin(100) chain(100) thinning(1) ///

     ) initsprevious ///

  nopause
```

```
*ROC AUC
predict pr
gen pre=pr+u0


*expand databse
expand denom
sort stratum
by stratum,sort: gen num=_n
by stratum: gen y=0 if numer<num
replace y=1 if y==.
gen p1=invlogit(pre)
disp _N
gen prop =denom/43247
gen weight=int(1/prop)

roctab y p1
roctab y p1 [fw=weight]


******************
******Model 2******
******************


use AMI_short30days, replace
sort hospital_new risk10  stratum
global MLwiN_path "C:\Program Files\MLwiN v3.02\mlwin.exe"
```

For peer review only

```
* Fit model 2

runmlwin prop cons, ///
  level4(hospital_new: cons) ///
  level3(risk10: cons) ///
  level2(stratum: cons) ///
  level1(stratum:) ///
  discrete(distribution(binomial) link(logit) denom(denom)) ///
  nopause

* Fit model 2  by MCMC

runmlwin prop cons, ///
  level4(hospital_new: cons, residuals(v, savechains("v2.dta", replace))) ///
  level3(risk10: cons, residuals(u, savechains("u2.dta", replace))) ///
  level2(stratum:) ///)
  level1(stratum:) ///
  discrete(distribution(binomial) link(logit) denom(denom)) ///
  mcmc(cc burnin(100) chain(100) thin(1) hc(3) savechains("b2.dta", replace)) ///
  initsprevious nopause

*ROC AUC
predict pr
gen prr=pr+u0
gen prh=pr+v0
gen prt=pr+u0+v0
```

For peer review only

```
*expand databse
expand denom
sort stratum
by stratum,sort: gen num=_n
by stratum: gen y=0 if numer<num
replace y=1 if y==.
gen Ph=invlogit(prh)
gen Pr=invlogit(prr)
gen Pt=invlogit(prt)

disp _N
gen prop =denom/43247
gen weight=int(1/prop)


roctab y Ph
roctab y Pr
roctab y Pt

roctab y Ph [fw=weight]
roctab y Pr [fw=weight]
roctab y Pt [fw=weight]
```

For peer review only

STROBE 2007 (v4) Statement—Checklist of items that should be included in reports of *cross-sectional studies*

Section/Topic	Item #	Recommendation	Reported on page #
**Title and abstract**	1	(*a*) Indicate the study's design with a commonly used term in the title or the abstract	2
		(*b*) Provide in the abstract an informative and balanced summary of what was done and what was found	2
**Introduction**			
Background/rationale	2	Explain the scientific background and rationale for the investigation being reported	3-6
Objectives	3	State specific objectives, including any prespecified hypotheses	6
**Methods**			
Study design	4	Present key elements of study design early in the paper	7
Setting	5	Describe the setting, locations, and relevant dates, including periods of recruitment, exposure, follow-up, and data collection	7
Participants	6	(*a*) Give the eligibility criteria, and the sources and methods of selection of participants	7
Variables	7	Clearly define all outcomes, exposures, predictors, potential confounders, and effect modifiers. Give diagnostic criteria, if applicable	8-9
Data sources/ measurement	8*	For each variable of interest, give sources of data and details of methods of assessment (measurement). Describe comparability of assessment methods if there is more than one group	8-9
Bias	9	Describe any efforts to address potential sources of bias	NA
Study size	10	Explain how the study size was arrived at	7
Quantitative variables	11	Explain how quantitative variables were handled in the analyses. If applicable, describe which groupings were chosen and why	9
Statistical methods	12	(*a*) Describe all statistical methods, including those used to control for confounding	8-17
		(*b*) Describe any methods used to examine subgroups and interactions	8
		(*c*) Explain how missing data were addressed	NA
		(*d*) If applicable, describe analytical methods taking account of sampling strategy	NA
		(*e*) Describe any sensitivity analyses	12-13
**Results**			

For peer review only

Participants	13*	(a) Report numbers of individuals at each stage of study—eg numbers potentially eligible, examined for eligibility, confirmed eligible, included in the study, completing follow-up, and analysed	Figure 1
		(b) Give reasons for non-participation at each stage	NA
		(c) Consider use of a flow diagram	Figure 1
Descriptive data	14*	(a) Give characteristics of study participants (eg demographic, clinical, social) and information on exposures and potential confounders	17, Table 2
		(b) Indicate number of participants with missing data for each variable of interest	NA
Outcome data	15*	Report numbers of outcome events or summary measures	17
Main results	16	(*a*) Give unadjusted estimates and, if applicable, confounder-adjusted estimates and their precision (eg, 95% confidence interval). Make clear which confounders were adjusted for and why they were included	18, Table 3
		(*b*) Report category boundaries when continuous variables were categorized	Table 2
		(*c*) If relevant, consider translating estimates of relative risk into absolute risk for a meaningful time period	NA
Other analyses	17	Report other analyses done—eg analyses of subgroups and interactions, and sensitivity analyses	18
**Discussion**			
Key results	18	Summarise key results with reference to study objectives	19
Limitations	19	Discuss limitations of the study, taking into account sources of potential bias or imprecision. Discuss both direction and magnitude of any potential bias	14, 21
Interpretation	20	Give a cautious overall interpretation of results considering objectives, limitations, multiplicity of analyses, results from similar studies, and other relevant evidence	20-21
Generalisability	21	Discuss the generalisability (external validity) of the study results	21
**Other information**			
Funding	22	Give the source of funding and the role of the funders for the present study and, if applicable, for the original study on which the present article is based	22

*Give information separately for cases and controls in case-control studies and, if applicable, for exposed and unexposed groups in cohort and cross-sectional studies.

Note: An Explanation and Elaboration article discusses each checklist item and gives methodological background and published examples of transparent reporting. The STROBE checklist is best used in conjunction with this article (freely available on the Web sites of PLoS Medicine at http://www.plosmedicine.org/, Annals of Internal Medicine at http://www.annals.org/, and Epidemiology at http://www.epidem.com/). Information on the STROBE Initiative is available at www.strobe-statement.org.

For peer review only

BMJ Open

A Cross-Classified Multilevel Analysis of Individual Heterogeneity and Discriminatory Accuracy (MAIHDA) to evaluate hospital performance: The case of Hospital Differences in Patient Survival after Acute Myocardial Infarction

Journal:	*BMJ Open*
Manuscript ID	bmjopen-2019-036130.R1
Article Type:	Original research
Date Submitted by the Author:	10-Aug-2020
Complete List of Authors:	Rodriguez-Lopez, Merida; Pontificia Universidad Javeriana - Cali,<br>Merlo, Juan; Lund University, Faculty of Medicine, Social Epidemiology<br>Perez-Vicente, Raquel; Lund University, Faculty of Medicine, Social Epidemiology<br>Austin, Peter; University of Toronto<br>Leckie, George; University of Bristol, Multilevel Modelling
**Primary Subject Heading**:	Epidemiology
Secondary Subject Heading:	Health services research, Public health, Cardiovascular medicine, Research methods
Keywords:	EPIDEMIOLOGY, Quality in health care < HEALTH SERVICES ADMINISTRATION & MANAGEMENT, Adult cardiology < CARDIOLOGY

SCHOLARONE™
Manuscripts

BMJ

I, the Submitting Author has the right to grant and does grant on behalf of all authors of the Work (as defined in the below author licence), an exclusive licence and/or a non-exclusive licence for contributions from authors who are: i) UK Crown employees; ii) where BMJ has agreed a CC-BY licence shall apply, and/or iii) in accordance with the terms applicable for US Federal Government officers or employees acting as part of their official duties; on a worldwide, perpetual, irrevocable, royalty-free basis to BMJ Publishing Group Ltd ("BMJ") its licensees and where the relevant Journal is co-owned by BMJ to the co-owners of the Journal, to publish the Work in this journal and any other BMJ products and to exploit all rights, as set out in our licence.

The Submitting Author accepts and understands that any supply made under these terms is made by BMJ to the Submitting Author unless you are acting as an employee on behalf of your employer or a postgraduate student of an affiliated institution which is paying any applicable article publishing charge ("APC") for Open Access articles. Where the Submitting Author wishes to make the Work available on an Open Access basis (and intends to pay the relevant APC), the terms of reuse of such Open Access shall be governed by a Creative Commons licence – details of these licences and which Creative Commons licence will apply to this Work are set out in our licence referred to above.

Other than as permitted in any relevant BMJ Author's Self Archiving Policies, I confirm this Work has not been accepted for publication elsewhere, is not being considered for publication elsewhere and does not duplicate material already published. I confirm all authors consent to publication of this Work and authorise the granting of this licence.

For peer review only

A Cross-Classified Multilevel Analysis of Individual Heterogeneity and Discriminatory Accuracy (MAIHDA) to evaluate hospital performance:

The case of Hospital Differences in Patient Survival after Acute Myocardial Infarction

Merida Rodriguez-Lopez[1,2]

Juan Merlo[1,3]

Raquel Perez-Vicente [1]

Peter C. Austin[4,5,6]

George Leckie [1,7]

For peer review only

[1] Unit for Social Epidemiology, Faculty of Medicine, Lund University, Malmö, Sweden

[2] Pontificia Universidad Javeriana seccional Cali, Colombia

[3] Center for Primary Health Care Research, Region Skåne, Malmö, Sweden

[4] ICES, Toronto, Ontario, Canada

[5] Institute of Health Management, Policy and Evaluation, University of Toronto, Ontario, Canada

[6] Schulich Heart Research Program, Sunnybrook Research Institute, Toronto, Ontario, Canada

[7] Centre for Multilevel Modelling, University of Bristol, Bristol, UK

Short title: Hospital Performance by MAIHDA

Corresponding author:
Dr. Merida Rodriguez-Lopez
merida.rdguez@gmail.com
Pontificia Universidad Javeriana-Cali, Colombia
Calle 18 No.118 - 250
Cali, Valle del Cauca, CO

Patient consent: Not required.

Abstract

Objective: To describe a novel strategy, Multilevel Analysis of Individual Heterogeneity and Discriminatory Accuracy (MAIHDA) to evaluate hospital performance, by analyzing differences in 30-day mortality after a first-ever acute myocardial infarction (AMI) in Sweden.

Design: Cross-classified study

Setting: 68 Swedish hospitals

Participants: 43,247 patients admitted between 2007-2009, with a first-ever AMI.

Primary and secondary outcome measures: We evaluate hospital performance by analyzing differences in 30-day mortality after a first-ever AMI using a cross-classified multilevel analysis We classified the patients into 10 categories according to a risk score (RS) for 30-day mortality and created 680 strata defined by combining hospital and RS categories.

Results: In the cross-classified multilevel analysis the overall RS adjusted hospital 30-day mortality in Sweden was 4.78% and the between-hospital variation was very small (Variance Partition coefficient (VPC)= 0.70%, Area under the curve (AUC)= 0.54). The benchmark value was therefore achieved by all hospitals. However, as expected, there were large differences between the RS categories (VPC= 34.13%, AUC= 0.77)

Conclusions: MAIHDA is a useful tool to evaluate hospital performance. The benefit of this novel approach to adjusting for patient RS is that it allowed one to estimate separate VPCs and AUC statistics to simultaneously evaluate the influence of RS categories and hospital differences on mortality. At the time of our analysis, all hospitals in Sweden were performing homogenously well. That is, the benchmark target for 30-day mortality was fully achieved and there were not relevant hospital differences. Therefore, possible quality interventions should be universal and oriented to maintain the high hospital quality of care.

Keywords: Multilevel analysis, Health Evaluation, Decision Making, ROC curve, Variance analysis

Strengths and limitations of this study

We provide a new analytical tool for analysing hospital performance based on multilevel analysis of individual heterogeneity and discriminatory accuracy (MAIHDA).

Cross-classified MAIHDA disentangles the specific role of the patient-mix vs. the hospital when analyzing quality outcomes.

We used a risk score to adjust for differences in patient-mix across hospitals. However, it is not a perfect instrument to quantify the true severity and mortality risk of a patient.

MAIHDA allows analysts to identify whether target or universal interventions are most appropriate to improve the quality of care.

We provide a three-step strategy to achieve a complete analysis of hospital performance. However, more elaborated strategies are also possible.

For peer review only - http://bmjopen.bmj.com/site/about/guidelines.xhtml

Introduction

Hospital effects

When evaluating institutional (e.g., hospital) performance in health care, traditional studies make two implicit assumptions. First, it is assumed that over and above patient characteristics, the hospital context exerts a general, shared effect on all patients at the hospital. This general hospital-context effect is argued to reflect the influence of many factors, for instance, hospital administration, access to resources, specialized knowledge, implementation of methods for disease management, and adoption of guidelines and pathways for patient treatments. Second, it is often assumed that the general hospital-context effect can be measured by quantifying differences between hospital averages in certain quality indicators. Therefore, the focus of the analysis is based on the interpretation of tables, funnel plots, control charts, 'league tables' or similar, where hospitals are ranked according to different quality indicators such as their average 30-day mortality after acute myocardial infarction (AMI) (1). Occasionally such analyses are accompanied by an estimation of the reliability of the ranking ("*rankability*") (2), but more often than not the focus of analysis remains on hospital averages.

Multilevel Analysis of Individual Heterogeneity and Discriminatory Accuracy (MAIHDA)

Recently, MAIHDA has been proposed as a novel strategy for evaluating hospital performance (3). In contrast with most traditional studies, hospital MAIHDA simultaneously focuses on both hospital averages *and* patient heterogeneity around such averages. In MAIHDA, the fundamental statement is that patient and hospital variation should not be analysed separately. Rather, we need to consider that the total individual outcome variance can be partitioned into variance components operating at different levels of analysis (4). From this perspective, hospital differences are not measured as the difference between hospital averages, but as the hospital general contextual effect (GCE). That is, the share of the total individual variance in patient

For peer review only - http://bmjopen.bmj.com/site/about/guidelines.xhtml

outcomes that is at the hospital level. This definition aligns with that for the variance partition coefficient (VPC) in multilevel modelling. The greater the GCE, the more important the hospital context is for explaining variation in individual outcomes (5-7). This idea is also closely related to the notion of discriminatory accuracy developed for the evaluation of the performance of prognostic and screening markers in medicine (8, 9). It is therefore possible to also use measures of discriminatory accuracy such as the area under the Receiving Operator Characteristics curve (AUC), to quantify the hospital GCE (10). See elsewhere for an extended explanation of the GCE concept (3, 7, 11). In this article, we argue that the systematic application of measures of variance and discriminatory accuracy is of fundamental relevance for meaningful performance evaluations (3, 5, 11-13).

Cross-classified MAIHDA

Hospital comparisons are usually adjusted for "patient-mix" using a risk score (RS). In traditional multilevel analysis of hospital performance, patient RS effects are modelled as fixed effects (e.g., by entering the set of RS categories as a series of dummy variables) while the hospital effects are modelled as random effects. In contrast, in the cross-classified MAIHDA approach both the RS and the hospitals are modelled as random effects. Readers familiar with the traditional application of multilevel modelling may query the treatment of RS categories as random effects. For example, while we can think of the hospitals as a sample drawn from the set of all possible hospitals, it proves harder to conceptualize the RS categories in this way as there is not a large population of RS categories from which they are drawn. This is, however, a philosophical, rather than a practical question. In fact, when studying hospitals in a country the hospitals are never a sample of an infinite super population of hospitals but a concrete set of facilities in a specific setting. Furthermore, many multilevel studies observe and analyze all the hospitals in a country in their data, and the total number of hospitals may not prove that large, yet here too the hospital effects will be treated as random effects. As discussed by Snijders and

For peer review only - http://bmjopen.bmj.com/site/about/guidelines.xhtml

Bosker, when defining the random intercept model (14), p. 45, the random effects model can be applied even when the idea of an infinite superpopulation is less evident. This approach is currently being applied when performing intersectional MAIHDA in social epidemiology (15).

The cross-classified approach provides several advantages over the traditional hierarchical multilevel approach. First, the cross-classified MAIHDA is parsimonious as it includes only one random parameter for the n RS categories rather than the $n-1$ dummy variables as in the fixed effects approach. Second, the cross-classified approach provides separate VPCs and AUCs for RS categories and for the hospital, allowing their magnitude to be contrasted. Thus, in contrast to the fixed-effects approach, it allows the importance of patient-mix vs. the hospital effects to be communicated on a common metric. In addition, hospital MAIHDA provides all the usual advantages of multilevel models. For instance, by providing reliability weighted hospital averages (shrunken residuals), it reduces the concern of monitoring outcome measures based on small hospital caseloads which otherwise may lead to extreme and unstable hospital rankings and, therefore, unreliable performance evaluation (16, 17). Both hierarchical and cross-classified MAIHDA are nowadays easy to implement in available software such as MLwiN that can be run from both within Stata (runmlwin) (18) and within R (R2MLwiN) (19).

Finally, for binary patient outcomes, such as 30-day mortality after AMI, multilevel analyses can be performed using a simple contingency table or matrix with strata defined by combinations of the hospitals and the RS categories capturing patient-mix. The only information required for the analysis is the overall number of patients and the number of AMI cases in each hospital-RS stratum. This aggregated approach maintains the joint distribution of the hospitals-RS information and provides the same model results (parameter estimates, standard errors, fit statistics and predictions) as when analysing the underlying individual level data. The aggregated approach also allows computationally efficient (fast) estimation as it allows analysing thousands of patients' outcomes using a dataset consisting of just a few

For peer review only - http://bmjopen.bmj.com/site/about/guidelines.xhtml

hundred strata. A further benefit of the aggregated approach is that the data can be shared since its aggregated presentation reduces ethical problems of confidentiality (statistical disclosure is not at risk). This in turn, improves the transparency of the research and facilitates the replication of the analysis and encourages the sharing of data to compare hospital performance between different settings.

The aim of this study

The aim of this study was to demonstrate a novel statistical approach (MAIHDA) to evaluate hospital performance and to orientate stakeholders to make assertive data informed decisions using a three-step framework. We do so by analyzing differences in 30-day mortality among patients admitted to the Swedish hospital with a first-ever AMI between 2007-2009.

Methods

Study Population

This is a cross-sectional study. We used information from the Swedish Patient Register (20) and from the Cause of Death Register (21) (National Board of Health and Welfare) as well as from Population Register (22) (Statistics Sweden). To ensure the anonymity of the subjects, the Swedish authorities transformed the personal identification numbers of the individuals (23) into arbitrary personal numbers before delivering the research databases to us, and we linked the databases using the anonymized identification number.

From the research database we identified all 47,462 patients with a discharge diagnosis of AMI coded as I21 according the 10th edition of the International Classification of Diseases (ICD), admitted to Swedish hospitals between 2007 and 2009 and being 45 to 80 years old. The flow diagram of the study population is shown in Figure 1.

Ethical statement

For peer review only

For peer review only - http://bmjopen.bmj.com/site/about/guidelines.xhtml

This research was done without patient involvement. The Regional Ethics Review Board in southern Sweden (# 2012/637) as well as the data safety committees from the National Board of Health and Welfare and from Statistics Sweden approved the construction of the database used in this study.

Data accessibility

The original databases used in our study are available from the Swedish National Board of Health and Welfare and Statistics. In Sweden, register data are protected by strict rules of confidentiality (24) but can be made available for research after a special review that includes approval of the research project by both an Ethics Committee and the authorities' own data safety committees. The Swedish authorities under the Ministry of Health and Social Affairs do not provide individual level data to researchers abroad. Instead, they normally advise researchers in other countries to cooperate with Swedish colleagues and analyze data in collaboration according to standard legal provisions and procedures.

However, in the approach we propose, it is technically possible to perform the analysis using a simple table defined by hospital and categories of risk score. The aggregated information as well as the additional encryption of the hospital name fully anonymized the table, which prevents the backwards identification of individuals even when very few patients are in a single cell of the table. Therefore, to increase transparency and facilitate the replication of our analysis we provide the table as a Stata dataset (Supplementary material S1) and a fully annotated Stata do-file to allow the replication of the analyses (Supplementary material S2). We also provide the table as a CSV file along with an R Script (Supplemental material S3, S4).

Assessment of variables

Patient outcome

For peer review only - http://bmjopen.bmj.com/site/about/guidelines.xhtml

The study outcome was all-cause mortality within 30-days after admission to the hospital (coded yes vs. no) due to AMI.

Risk score for mortality

An inherent difficulty when investigating quality outcome indicators such as mortality is the threat of confounding due to differences in patient-mix across hospitals. The geographical areas covered by the hospital may vary in the demographical and disease characteristics of the patients. Furthermore, patients with a worse prognosis may be channeled to certain hospitals providing specialized care and this selection of patients will further confound the evaluations of hospitals differences. To reduce this form of confounding we computed a RS for 30-day mortality in the sample of AMI patients. Initially we selected a priori 40 variables including sex (man vs. woman), age in years (as a quadratic function) and several previous diseases (ICD-10 codes) registered in the Swedish Patient Register. We then modeled 30-day mortality using a single-level logistic regression in STATA 14 (Statacorp, College Station, Texas, US), and stepwise variable selection with a significance level of 0.10 and 0.05 for removal and inclusion of variables, respectively. The variables retained in the final model were, age, arrhythmia (I48-I49), cancer (C1-D4), diseases of the cerebral arteries (I6), chronic diseases of the lower respiratory tract (J4), diabetes (E10-E14), digestive diseases (K0-K9), other types of heart disease (I3-I5), hypertension (I10-I13 I15), hearth failure (I50), injury (S00-T14), ischemic coronary artery disease (I20-I25), lung cancer (C34), mental diseases (F0-F9), and peripheral vascular disease (I74 I80) as well as respiratory diseases (J0-J9). Finally, we obtained the predicted probability of 30-day mortality and defined it as our patient risk score (RS). We discretized the RS into 10 categories using the decile values of the RS distribution. We chose deciles to provide enough granulation of the continuous RS variable and enough categories to be included as a random effect in the multilevel model.

For peer review only - http://bmjopen.bmj.com/site/about/guidelines.xhtml

Statistical analyses

We analysed 30-day mortality among 43,247 patients admitted to 68 Swedish hospitals between 2007-2009, with a first-ever AMI. We classified the patients into 10 RS categories for 30-day mortality and created 680 strata defined by combining hospital and RS categories. In the first step (model 1), we applied a traditional hierarchical multilevel logistic regression model with patients clustered within hospitals. In a second step (model 2), and in order to adjust for patient-mix, we performed a cross-classified multilevel model of patient outcomes with both RS categories and hospitals random effects (25). We estimated the VPC and the AUC to evaluate differences between RS categories and between hospitals in a common way.

Estimation methods

We performed the estimations using Markov Chain Monte Carlo (MCMC) methods, with diffuse (vague, flat, or minimally informative) prior distributions for all parameters. We used quasilikelihood methods to provide starting values for all parameters. For each model, the burn-in length was 5,000 iterations. We ran the model for a further 10,000 monitoring iterations and used the resulting parameter chains from the MCMC to construct 95% credible intervals (CI) for all model predictions to communicate statistical uncertainty (Supplementary material S2). Visual assessments of the parameter chains and standard MCMC convergence diagnostics suggested that the monitored chains had converged.

Model 1: Unadjusted multilevel analysis

Model 1 was a multilevel logistic regression for patient mortality where we only include a hospital random effect to account for the variation in mortality rates across hospitals. Let, y_j denote the number of deaths in hospital j ($j = 1,...,68$). The model can then be written as

$$y_j \sim \text{Binomial}(n_j, \pi_j)$$

For peer review only - http://bmjopen.bmj.com/site/about/guidelines.xhtml

$$\text{logit}\,(\pi_j) \equiv \log\left(\frac{\pi_j}{1-\pi_j}\right) = \beta_0 + H_j$$
$$H_j \sim N(0,\sigma_H^2)$$

Formula 1

where n_j denotes the total number of patients in hospital j, π_j denotes the probability of death for patients in that hospital, β_0 denotes the intercept or precision weighted grand mean, that in logistic regression represents the average of the hospital-specific shrunken (i.e., reliability-weighted) residuals values, and H_j denotes the hospital-specific random effects for the 68 hospitals (26). The random effects are assumed to be normally distributed with zero mean and between-hospital variance σ_H^2.

The purpose of this model was to evaluate unadjusted hospital differences in average mortality risk. For this aim, we (A) ranked the hospitals according to their mortality risk; and (B) complemented this information by quantifying the size of the GCE.

A) Ranking of the hospitals

To rank hospitals according to their *unadjusted* mortality rates, we predict the absolute risk (AR_j) of 30-day mortality and its 95% CI in each hospital. To do so, we first transformed the predicted logit of 30-day mortality into proportions (π_j) as follows

$$\text{AR}_H \equiv \pi_j \equiv \text{logit}^{-1}\,(\beta_0 + H_j) = \frac{\exp\,(\beta_0 + H_j)}{1 + \exp\,(\beta_0 + H_j)}$$

Formula 2

B) Measuring the hospital GCE.

We estimate the hospital GCE by means of two measures:

(i) The Variance Partition Coefficient for the hospital level (VPC_H)

The VPC_H can be calculated based on the latent response formulation of the model which is an approach widely adopted today in multilevel applied work (27-31) .

For peer review only - http://bmjopen.bmj.com/site/about/guidelines.xhtml

$$\text{VPC}_H = \frac{\sigma_H^2}{\sigma_H^2 + \frac{\pi^2}{3}}$$

Formula 3

Where $\frac{\pi^2}{3} \cong 3.29$ denotes the variance of a standard logistic distribution. We then multiply the VPC_H by 100 and interpret it as a percentage.

The VPC_H, quantifies the share of the total individual differences in the latent propensity of 30-day mortality that is at the hospital level. The VPC_H embraces the influence of the hospital context on the patient outcome without identifying any specific hospital information. However, the VPC_H, may also reflect differences in patient-mix between hospitals. In any case, theVPC_H, represents the hospital ceiling effect or potential maximum influence of the hospital attended. In the absence of confounding by patient-mix, the higher the VPC_H, the higher the hospital GCE is. In other words, the more relevant the hospital context is for understanding individual variation in the latent risk for 30-day mortality.

(ii) The area under the receiver operating characteristics curve for the hospital (AUC_H)

A well-known measure of discriminatory accuracy is the AUC (10, 11, 32). In our case the hospital AUC_H measures how well the model predicted probabilities based on the attended hospitals distinguish between two outcome categories (death within 30 days or survival). The AUC_H is constructed by plotting the true positive fraction (TPF) against the false positive fraction (FPF) for different thresholds of the predicted probabilities. The AUC takes a value between 1 and 0.5 where 1 is perfect discrimination and 0.5 would be equally as informative as flipping a coin (i.e., the hospital information has no discriminatory accuracy).

We calculated the AUC_H and to account for the different number of patients in each hospital we also calculated the weighted AUC (AUC_{wH}) where every patient was weighted by the inverse

For peer review only - http://bmjopen.bmj.com/site/about/guidelines.xhtml

of the number of patients at his/her hospital. In our study both the unweighted and the weighted AUCs were almost identical so we only present the unweighted AUC_H.

Model 2: Patient-mix adjusted multilevel analysis

Model 2 was a cross-classified multilevel logistic regression (25) for patient mortality in which we included both hospital and RS category random effects to simultaneously account for the variation in mortality across both hospitals and RS categories and to therefore adjust the hospital effects for patient-mix. The model can be written as:

$$y_j \sim \mathrm{Binomial}(n_j, \pi_j)$$
$$\mathrm{logit}\,(\pi_{jk}) \equiv \log\left(\frac{\pi_{jk}}{1-\pi_{jk}}\right) = \beta_0 + H_j + RS_k$$
$$H_j \sim N(0, \sigma_H^2)$$
$$RS_k \sim N(0, \sigma_{RS}^2)$$

Formula 4

where RS_k denotes the random effect associated with RS categories k ($k = 1,\ldots,10$), which are assumed to be normally distributed with zero mean and between-RS variance σ_{RS}^2.

The predicted logit of 30-day mortality was transformed into proportions. The mortality rates from this model are standardized and represents the rate that each hospital would have experienced if all hospitals had treated the same patients, in our case a patient with an average RS value in the population or $RS_k = 0$.

The purpose of model 2 was to evaluate patient-mix adjusted hospital differences in average mortality risk. Therefore, and analogously to model 1, we ranked the hospitals according to their RS adjusted mortality risk and complemented this information by quantifying the size of the hospital GCE net of the observed patient-mix influence. Visual inspection of the hospital and RS category predicted random effects showed the random effect normality assumptions were satisfied. (Supplementary material S2). As a measure of the patient-mix adjusted hospital

For peer review only - http://bmjopen.bmj.com/site/about/guidelines.xhtml

GCE, we obtained the hospital VPC_H as

$$\mathrm{VPC}_H = \frac{\sigma_H^2}{\sigma_H^2 + \sigma_{RS}^2 + \frac{\pi^2}{3}}$$

Formula 5

We also calculated the VPC for the RS category (VPC_{RS}) as

$$VPC_{RS} = \frac{\sigma_{RS}^2}{\sigma_H^2 + \sigma_{RS}^2 + \frac{\pi^2}{3}}$$

Formula 6

The adjusted VPC_H and VPC_{RS} inform on the share of the total individual variance in the latent propensity of 30-day mortality that is at the hospital and at the RS category level respectively, net of the influence of the other factor. Both measures are estimated on the same scale and can therefore be directly compared to evaluate the relative relevance of hospital versus patient-mix information when it comes to understanding patient differences in the latent propensity of death. We also calculated the adjusted AUC for the hospital level (AUC_H) and for RS category levels (AUC_{RS}) including their specific random effects when calculating the predicted probabilities.

While patients with relatively mild conditions may have similarly good outcomes regardless of where they are treated, outcomes of the most complex patients may be affected by hospital performance. We therefore fitted a cross-classified model including a random interaction effect between the Hospital and the RS (Supplementary material S2). That is, we allowed the effect that a Hospital has on their patients to vary according to the RS classification of their patients and vice versa. However, the resulting interaction classification variance was very low, suggesting that hospital attended and patient RS have additive effects on the log-odds of AMI. Consequently, we based our analysis on model 1 and 2.

For peer review only - http://bmjopen.bmj.com/site/about/guidelines.xhtml

Software

All models were run in MLwiN 3.05 (33) called from Stata using the *runmlwin* command (18). We note that MLwiN can equally be called from within R using the sister R2MLwiN package (19) and so our analysis can also be replicated by readers in that statistical package.

Evaluating hospital performance with MAIHDA

The present cross-classified MAIHDA framework extends that which was described in a previous publication aimed to the evaluation of geographical differences in health outcomes (34). The framework proposes three steps that need be considered to achieve a complete analysis of hospital performance. However, more elaborated strategies are of course also possible, and the presented framework is open for modifications and extensions. The application of the framework in our study was as follows

Step 1. Identifying a benchmark value and evaluating the adjusted hospital mortality rates against it.

When evaluating hospital performance, we need to identify a benchmark value expressing a tolerable average level of 30-day mortality in the population of AMI patients. However, the selection of a specific benchmark is often difficult and arbitrary. We can use an *internal benchmark* defined as the β_0 obtained in model 2 (*Formula 4*). That is, the mortality rate in a hospital with an average mortality ($H_j = 0$) treating patients with an average RS ($RS_k = 0$). This choice is meaningful since comparing with a national average seems "fair" and being RS adjusted, tertiary care hospitals with more severe cases do not unfairly push the hospital effect towards a higher value as in the crude average rate. However, being an adjusted rate the value does not necessarily resemble the crude rates and can only be used for relative comparisons. Other adjustments are possible such as computing adjusted hospital mortality rates by holding RS_k value equal to the 90th percentile of the RS random effect distribution. Another possibility

For peer review only - http://bmjopen.bmj.com/site/about/guidelines.xhtml

would be to calculate the adjusted hospital rates that would arise if each hospital treated a nationally representative sample of patients. However, statistically, this would be more complex to do as this would require integrating out the RS random effect (via simulation) and so we do not pursue this here.

We could also use and *external benchmark* by applying a RS equation for 30-day mortality obtained in another country or in an international collaboration. This approach seems worthy for international comparisons, but it may also be inappropriate if the RS equation does not properly account for population demographical and co-morbidities differences in risk, and the diagnostic criteria are not fully standardized. Finally, we could use an arbitrary rate based on previous evidence and proven expertise. Considering the data published by the Organisation for Economic Co-operation and Development (OECD) (35) and a recent review article (36), we decide a RS adjusted 30-day mortality of less than 6% as a desirable target value for the purposes of this illustrative application. We defined three categories of achievement in relation to the benchmark, fully reached target (≤6%), closely reached target value (6%-8%), and insufficiently reached target value (≥8%). We used caterpillar graphs to compare the RS adjusted hospital rates in relation to the benchmark value of 6%.

Step 2. Quantifying the size of the hospital differences using the VPC and the AUC

Currently there is no official guidance for assessing the magnitude of the VPC or the AUC in the context of studying hospital differences in RS adjusted hospital 30-day mortality but a practical proposal is described in Table 1. This table also shows the corresponding AUC values according to the simulated relationship between the AUC and VPC published elsewhere (34). The proposed values are based on the authors' own experience, but further discussion is encouraged to arrive at a standard classification. Furthermore, different standards may ultimately be required and developed for different outcomes in different contexts and at

For peer review only - http://bmjopen.bmj.com/site/about/guidelines.xhtml

different points in time. For instance, Hosmer and Lemeshow (37) suggested that AUC of 0.70 to 0.80 are 'acceptable', 0.80 to 0.90 'excellent' and 0.9 or above 'outstanding', while AUC of 0.50 suggests discrimination by chance e.g. similar to tossing a coin to decide death or alive.

Step 3. Interpreting results to evaluate performance

The two primary questions for the hospital performance evaluation were, (i) has the benchmark value been insufficiently, closely, or fully reached? (ii) are there substantial differences between the hospitals, or do they perform homogenously? To answer both questions, we created a framework (Table 1) with 15 scenarios combining information on the benchmark value achievement and the size of hospital differences based on model 2.

In the best scenario (scenario A) the desired target level has been fully achieved overall (averaging across all hospitals the adjusted mortality rate is less than 6%), and hospital differences are effectively absent (the Hospital GCE is effectively absent). The conclusion would be that all hospitals have performed similarly well. In the worst scenario (scenario C) the desired target level has not been achieved overall, and between-hospital differences are again absent. The conclusion would be that all hospitals have performed similarly but poorly. Observe that in both scenarios A and C, interventions only targeted to specific hospitals are not justifiable. Rather any intervention should be universal (i.e., directed to all hospitals) as in both scenarios all hospitals are performing similarly. In scenario A, the intervention would be oriented to *maintaining* the overall high quality while in scenario C, the objective would be to improve the quality in *all* hospitals.

The interpretation of the scenarios in the lowest corners of the table (M and O) is driven by the very large size of the hospital differences. For example, in scenario M some hospitals may not have achieved the target level even though the average 30-day mortality is below the benchmark value. In contrast, in scenario O, some hospitals may have achieved the target level even though

For peer review only - http://bmjopen.bmj.com/site/about/guidelines.xhtml

the target has not been achieved overall. In these scenarios (M and O) targeted hospitals interventions are justified to focus especially on those hospitals operating above the benchmark value.

Patient and public involvement: No patient and public involved

Results

Table 2 describes the estimation sample. The mean age of the 43,247 patients with a first-ever AMI was approximately to 67 years and only one third were female. As expected, mortality was strongly related to the RS of the patient, so that patients in the highest RS category present a mortality risk about 36 times higher than in the low RS group (25.5% vs. 0.7%).

In the crude, unadjusted analysis based on simple proportions (i.e., obtained without multilevel modeling), 8.18% of the patients died within a 30-day period after admission to the hospital and the crude proportions ranged from 3.45% to 17.32% across hospitals, as indicated in figure 2 (white circles). However, accounting for the reliability of the hospital information in the hierarchical multilevel analysis (Model 1), the mean unadjusted hospital rate from the multilevel model was 8.00% and ranged between 6.27% and 12.21% (grey circles). Thus, the multilevel model was able to extract the systematic variation of interest between hospitals from the observed variation which additionally includes a substantial statistical noise component due to the relatively small patient caseload per hospital (from a statistical sample size perspective). After further adjustment for patient-mix variation across hospitals in the cross-classified multilevel model (model 2) the mean hospital 30-day mortality was 4.78% and varied only between 3.44% and 7.48% (black circles) and the 95% credible intervals (grey vertical lines) considerably overlap each other. Thus, once we performed reliability weighted estimations and adjusted for hospital differences in patient mix, very little variation in performance remained

For peer review only - http://bmjopen.bmj.com/site/about/guidelines.xhtml

across hospitals. These adjusted mortality rates represent hospital rates as if all hospital treated the same patient with average RS (i.e., $RS_k = 0$), which is a suitable approach for evaluating hospital performance.

Table 3 reports the results from the multilevel variance analysis used to estimate the GCE. In model 1, the VPC_H was 1.20%, and the $AUC_H = 0.55$. Adjustment for patient-mix by including the RS categories in the cross-classified model 2 reduced the value of the VPC_H= 0.70%, and slightly decreased the initial AUC_H= 0.54. Therefore, the hospital differences were *absent*, suggesting that hospital attended is not a driving factor in determining patient mortality. In contrast, information on the RS value of the patients was much more relevant than information on the hospital in which they were treated. The VPC_{RS} was very large (i.e., 34.13%) and the AUC_{RS}= 0.77. Figure 3 clearly depicts the differential discriminatory accuracies of the hospital and RS random effects.

Discussion

Analyzing 30-day mortality after AMI in Sweden, we illustrate the MAIHDA approach to auditing hospital performance. By considering both the size of the hospital GCE and the RS adjusted hospital 30-day mortality rates in relation to a pre-set benchmark value, we were able to perform a more nuanced evaluation of hospital performance compared with traditional methods focused exclusively on differences between hospital averages.

Following the framework presented in table 1, our results corresponds with the scenario A and indicate that, at the time of our analysis, all hospitals in Sweden were performing homogenously well. That is, the overall 30-day mortality (i.e., 4.78%) was under the benchmark value of ≤6% and there were no relevant hospital differences (VPC_H= 0.70% and AUC_H= 0.54). The ranking

For peer review only - http://bmjopen.bmj.com/site/about/guidelines.xhtml

of hospitals in our study (Figure 2) also shows that no hospitals could be statistically distinguished with any degree of certainty from the overall average mortality.

We have found similar low VPC values when investigating hospital differences in mortality after AMI admission in Ontario, Canada (38) and in Sweden (39) as well as mortality after heart failure in Sweden (40) and in Denmark (3). The low hospital GCE suggests universal instead of targeted interventions, as all hospitals perform homogenously. There may be, however, other patient outcomes where the hospital GCE would be much larger. For instance, when auditing adherence with guidelines for statin prescription (41, 42) or process quality indicators of diabetes care like albuminuria analysis (43).

The evaluation of institutional performance using VPC is not new (12, 39, 44, 45). Normand (46) states that when evaluating hospital performance if the VPC is zero *there are no hospital quality differences, that is, the chance that a patient experiences an event after being treated is the same regardless of the hospital (p. 33)*. This idea is also explicitly expressed by the committee assigned to set statistical guidelines for assessing hospital performance in USA (47). The share of the total variance that is at the hospital level is crucial for evaluating performance. However, this fundamental concept needs be applied more extensively. Today, it is recognized that multilevel models (hierarchical or mixed-effect models) are the preferred methodology for provider profiling. However, the substantive analysis of components of variance still receives little attention and most studies only consider multilevel modeling for its capacity to account for the clustering of patients within hospitals in order to obtain "correct standard errors" on regression coefficients and odds ratios. Some authors even conclude that hospital averages (odds ratios, observed/expected values) obtained from multilevel analyses gives similar results compared to traditional logistic regressions analyses. This situation is interpreted as an argument for keeping the traditional logistic regression as the standard method for performing risk adjustment of hospital quality comparisons (48-50). However, we do not agree with this

For peer review only - http://bmjopen.bmj.com/site/about/guidelines.xhtml

opinion. The first reason is that the fixed effects approach does not explicitly inform on components of variance. The second reason is that the equivalence between traditional and multilevel regression results only occurs when the hospital GCE (i.e., the clustering) is low and the number of patients at the hospitals is very high (i.e., reliable estimation of hospital averages) (26). In other words, traditional non-multilevel analyses give similar results to the multilevel analysis only when the hospital differences are not relevant (i.e., low VPC) and the patient load is very large in every hospital (which is rarely the case). In addition, hospital level variables appear paradoxically more statistically "significant" when the hospital level is less relevant (i.e., low VPC) (7). Information on the size of the hospital GCE is, therefore, fundamental for a sound analysis of hospital performance.

In this study we have applied the AUC to evaluate the hospital GCE. The AUC is a measure of discriminatory accuracy frequently used for gauging the performance of prognostic and screening markers in medicine (8, 9) but it can also be used to quantify hospital GCE (10, 34). So far, many epidemiologists may not be familiar with the use of measures of components of variance like the VPC for binary outcomes (30). However, the AUC measure is well established in clinical and health care epidemiology and the information it gives is relatively easy to interpret and communicate. From this perspective, the evaluation of hospital performance resembles a screening test and so we must therefore know the discriminatory accuracy of, for instance, a "league table" to make informed decisions.

Analyzing outcome indicators always requires adjustment for confounding (i.e., patient-mix). We performed this adjustment using an innovative strategy that use hospitals and decile groups of RS in a cross-classified multilevel analysis. This approach provides a new option that could be very useful in some cases. In other cases, the classical inclusion of the patient-mix information as fixed effects may be more suitable. Both approaches provide similar results in terms of the hospital VPC, AUC, and model fit (Supplemental material S2). Then, the

For peer review only - http://bmjopen.bmj.com/site/about/guidelines.xhtml

investigator may judge which approach is most suitable for the research question. A key advantage of our approach is it allowed a directed comparison of the importance of patient case mix and hospital effects in explaining variation in patient outcomes across hospital. We were able to calculate and compare a VPC for RS categories with the VPC for hospitals. We did not use existing patient-mix adjustment scores such as the Charlson Risk Score (51), the Elixhauser Score (52), or the Center for Medicare & Medicaid Services Hierarchical Condition Category "CMS-HCC" risk adjuster (53). Our aim was not primarily to create a new risk score equation but only to perform a parsimonious patient-mix adjustment where the RS summarizes a large number of variables into a single construct.

This study has some limitations that need to be discussed. Unfortunately, the database for which we have ethical allowance for this study does not provide information on the severity of AMI or on revascularization procedures (e.g., PCI, CABG). The inclusion of these variables could possibly improve the RS. However, we believe this improvement would be small and unlikely to affect our conclusions. Additionally, the RS is not a perfect instrument to quantify the true severity and mortality risk of a patient. Nevertheless, the RS categories we use are strongly associated with mortality and the RS alone shows a high discriminatory accuracy. RS may reflect practice or coding patterns of hospitals. However, Sweden has a very homogenous health care system with centralized diagnostic rules which may reduce the risk of differential diagnosis setting. Finally, to explore the potential loss of information due to the categorization of RS into deciles, we performed a sensitivity analysis with 15 and 20 categories. The results were similar to those obtained in model 2 (data not shown)

In summary, we illustrate the MAIHDA approach to auditing hospital performance using a three-step strategy. We argue that it is necessary to consider both the size of the hospital GCE and the RS adjusted 30-day mortality in relation to a pre-set benchmark value. Our results indicate that, at the time of our analysis, all hospitals in Sweden were performing homogenously

For peer review only - http://bmjopen.bmj.com/site/about/guidelines.xhtml

well. That is, there were no meaningful hospital differences and the benchmark target for 30-day mortality rate after admission for AMI was well achieved. Therefore, possible quality interventions should be universal and oriented to maintain the high hospital quality of care.

Funding

The Swedish Research Council (Vetenskapsrådet) supported this work through the project "Multilevel Analyses of Individual Heterogeneity: innovative concepts and methodological approaches in Public Health and Social Epidemiology": (#2017-01321, PI: Juan Merlo). Dr. Austin received a personal Mid-Career Investigator award from the Heart and Stroke Foundation.

Contributors:

JM had the initiative of the study and acquired the data.

JM and M R-L wrote the original manuscript.

M R-L and RP-V performed the analyses in coordination with JM.

PA and GL provided advanced statistical support.

All the authors have contributed to the design of the study, developed the methodology, direction of the analyses, interpretation of the results and drafting of the manuscript

All authors have revised the last version of the manuscript.

Conflicts of Interest: None declared

Data availability statement: Data are available upon reasonable request.

For peer review only

Original data are available from the Swedish National Board of Health and Welfare and Statistics, after approval of a research project by an Ethical Committee and by the data safety committees of Swedish Authorities. However, it is possible to replicate the analyses performed, by using a table provided as supplementary information.

References

1. Chung SC, Sundstrom J, Gale CP, James S, Deanfield J, Wallentin L, et al. Comparison of hospital variation in acute myocardial infarction care and outcome between Sweden and United Kingdom: population based cohort study using nationwide clinical registries. BMJ. 2015;351:h3913.
2. van Dishoeck AM, Lingsma HF, Mackenbach JP, Steyerberg EW. Random variation and rankability of hospitals using outcome indicators. BMJ Qual Saf. 2011;20(10):869-74.
3. Ghith N, Wagner P, Frolich A, Merlo J. Short Term Survival after Admission for Heart Failure in Sweden: Applying Multilevel Analyses of Discriminatory Accuracy to Evaluate Institutional Performance. PLoS One. 2016;11(2):e0148187.
4. Merlo J. Invited commentary: multilevel analysis of individual heterogeneity-a fundamental critique of the current probabilistic risk factor epidemiology. Am J Epidemiol. 2014;180(2):208-12; discussion 13-4.
5. Austin PC, Merlo J. Intermediate and advanced topics in multilevel logistic regression analysis. Stat Med. 2017;36(20):3257-77.
6. Merlo J, Viciana-Fernandez FJ, Ramiro-Farinas D, LDAP RG. Bringing the individual back to small-area variation studies: A multilevel analysis of all-cause mortality in Andalusia, Spain. Social Science & Medicine. 2012;75(8):1477-87.
7. Merlo J, Wagner P, Austin PC, Subramanian SV, Leckie G. General and specific contextual effects in multilevel regression analyses and their paradoxical relationship: A conceptual tutorial. SSM Popul Health. 2018;5:33-7.
8. Khoury MJ, Newill CA, Chase GA. Epidemiologic evaluation of screening for risk factors: application to genetic screening. Am J Public Health. 1985;75(10):1204-8.
9. Pepe MS, Janes H, Longton G, Leisenring W, Newcomb P. Limitations of the odds ratio in gauging the performance of a diagnostic, prognostic, or screening marker. Am J Epidemiol. 2004;159(9):882-90.
10. Wagner P, Merlo J. Measures of discriminatory accuracy in multilevel analysis. European Journal of Epidemiology. 2013;28(1, Supplement):135.
11. Merlo J, Wagner P, Ghith N, Leckie G. An Original Stepwise Multilevel Logistic Regression Analysis of Discriminatory Accuracy: The Case of Neighbourhoods and Health. PLoS One. 2016;11(4):e0153778.
12. Merlo J, Ostergren PO, Broms K, Bjorck-Linne A, Liedholm H. Survival after initial hospitalisation for heart failure: a multilevel analysis of patients in Swedish acute care hospitals. J Epidemiol Community Health. 2001;55(5):323-9.
13. Van Leeuwen W, Nilsson S, Merlo J. Mother's country of birth and prescription of psychotropic medication in Swedish adolescents: a life course approach. BMJ Open. 2012;2(5).
14. Snijders TAB, Bosker RJ. Multilevel analysis: an introduction to basic and advanced multilevel modeling. 1 ed. Thousand Oaks, California: SAGE Publications; 1999 1999///.
15. Merlo J. Multilevel analysis of individual heterogeneity and discriminatory accuracy (MAIHDA) within an intersectional framework. Soc Sci Med. 2018;203:74-80.

For peer review only - http://bmjopen.bmj.com/site/about/guidelines.xhtml

16. Krell RW, Hozain A, Kao LS, Dimick JB. Reliability of risk-adjusted outcomes for profiling hospital surgical quality. JAMA surgery. 2014;149(5):467-74.
17. Goldstein H, Spiegelhalter D. League Tables and Their Limitations: Statistical Issues in Comparisons of Institutional Performance. Journal of the Royal Statistical Society, SocA. 1996;159:385-443.
18. Leckie G, Charlton C. runmlwin: A Program to Run the

MLwiN Multilevel Modeling Software from within Stata. Journal of

Statistical Software. 2013;52(11):1-40.
19. Zhang Z, Parker R, Charlton C, Leckie G, Browne WJ. R2MLwiN - A program to run the MLwiN multilevel modelling software from within R. Journal of Statistical Software, . 2016;72(10):1-43.
20. Ludvigsson JF, Andersson E, Ekbom A, Feychting M, Kim JL, Reuterwall C, et al. External review and validation of the Swedish national inpatient register. BMC Public Health. 2011;11:450.
21. Brooke HL, Talback M, Hornblad J, Johansson LA, Ludvigsson JF, Druid H, et al. The Swedish cause of death register. Eur J Epidemiol. 2017;32(9):765-73.
22. Ludvigsson JF, Almqvist C, Bonamy AK, Ljung R, Michaelsson K, Neovius M, et al. Registers of the Swedish total population and their use in medical research. Eur J Epidemiol. 2016;31(2):125-36.
23. Ludvigsson JF, Otterblad-Olausson P, Pettersson BU, Ekbom A. The Swedish personal identity number: possibilities and pitfalls in healthcare and medical research. Eur J Epidemiol. 2009;24(11):659-67.
24. Public Access to Information and Secrecy Act: Government Offices of Sweden; 2009 [Available from: http://www.government.se/information-material/2009/09/public-access-to-information-and-secrecy-act/.
25. Leckie G. Cross-classified Multilevel Models - Concepts. LEMMA VLE Module 12, 1-60. URL: http://wwwbristolacuk/cmm/learning/online-course/. 2013.
26. Evans CR, Leckie G, Merlo J. Multilevel versus single-level regression for the analysis of multilevel information:The case of quantitative intersectional analysis. Social Science & Medicine. 2019.
27. Goldstein H, Browne WJ, Rashbash J. Partitioning variation in multilevel models. Understanding Statistics: Statistical Issues in Psychology, Education, and the Social Sciences. 2002;1(4):223-31.
28. Merlo J, Yang M, Chaix B, Lynch J, Rastam L. A brief conceptual tutorial on multilevel analysis in social epidemiology: investigating contextual phenomena in different groups of people. J Epidemiol Community Health. 2005;59(9):729-36.
29. Merlo J, Chaix B, Yang M, Lynch J, Rastam L. A brief conceptual tutorial of multilevel analysis in social epidemiology: linking the statistical concept of clustering to the idea of contextual phenomenon. J Epidemiol Community Health. 2005;59(6):443-9.
30. Merlo J, Chaix B, Ohlsson H, Beckman A, Johnell K, Hjerpe P, et al. A brief conceptual tutorial of multilevel analysis in social epidemiology: using measures of clustering in multilevel logistic regression to investigate contextual phenomena. J Epidemiol Community Health. 2006;60(4):290-7.
31. Goldstein H, Browne W, Rasbash J. Partitioning variation in generalised linear multilevel models. Understanding Statistics. 2002;1:223-32.
32. Wagner P, Merlo J. Discriminatory accuracy of a random effect in multilevel logistic regression. 20th IEA World Congress of Epidemiology (WCE2014). 2014.
33. Charlton C, Rasbash J, Browne WJ, Healy M, Cameron B. MLwiN Version 3.00. Centre for Multilevel Modelling, University of Bristol.; 2017.

For peer review only - http://bmjopen.bmj.com/site/about/guidelines.xhtml

34. Merlo J, Wagner P, Leckie G. A simple multilevel approach for analysing geographical inequalities in public health reports: The case of municipality differences in obesity. Health Place. 2019;58:102145.
35. OECD. Mortality following acute myocardial infarction (AMI). 2017. In: Health at a Glance 2017: OECD Indicators, [Internet]. Paris: OECD Publishing,.
36. Anderson JL, Morrow DA. Acute Myocardial Infarction. The New England journal of medicine. 2017;376(21):2053-64.
37. Hosmer DW, Lemeshow S. Applied logistic regression. 2nd ed. New York: Wiley; 2000. xii, 373 p. p.
38. Austin PC, Wagner P, Merlo J. The median hazard ratio: a useful measure of variance and general contextual effects in multilevel survival analysis. Stat Med. 2017;36(6):928-38.
39. Merlo J, Hakansson A, Beckman A, Lindblad U, Lindstrom M, Gerdtham UG, et al. [Equally good care of myocardial infarction in Sweden today. Geographic differences in mortality are without significance for the individual patient]. Lakartidningen. 2005;102(1-2):20-3.
40. Merlo J, Ostergren P, Broms K, Bjorck-Linne A, Liedholm H. Survival after initial hospitalisation for heart failure: a multilevel analysis of patients in Swedish acute care hospitals. Journal of Epidemiology and Community Health. 2001;55(5):323-9.
41. Hjerpe P, Ohlsson H, Lindblad U, Bostrom KB, Merlo J. Understanding adherence to therapeutic guidelines: a multilevel analysis of statin prescription in the Skaraborg Primary Care Database. Eur J Clin Pharmacol. 2010.
42. Ohlsson H, Lindblad U, Lithman T, Ericsson B, Gerdtham UG, Melander A, et al. Understanding adherence to official guidelines on statin prescribing in primary health care--a multi-level methodological approach. Eur J Clin Pharmacol. 2005;61(9):657-65.
43. Ghith N, Merlo J, Frolich A. Albuminuria measurement in diabetic care: a multilevel analysis measuring the influence of accreditation on institutional performance. BMJ Open Qual. 2019;8(1):e000449.
44. Ohlsson H, Librero J, Sundquist J, Sundquist K, Merlo J. Performance evaluations and league tables: do they capture variation between organizational units? An analysis of 5 Swedish pharmacological performance indicators. Med Care. 2011;49(3):327-31.
45. Merlo J, Gerdtham UG, Eckerlund I, Hakansson S, Otterblad-Olausson P, Pakkanen M, et al. Hospital level of care and neonatal mortality in low- and high-risk deliveries: reassessing the question in Sweden by multilevel analysis. Med Care. 2005;43(11):1092-100.
46. Normand SLT, Ash SA, Fienberg SE, Stukel TA, Utts J, Louis TA. League Tables for Hospital Comparisons. Annu Rev Stat App. 2016;3:21-50.
47. Ash AS, Fienberg E, Louis A, Norm S-lT, Stukel A, Utts PJ. STATISTICAL ISSUES IN ASSESSING HOSPITAL PERFORMANCE Commissioned by the Committee of Presidents of Statistical Societies The COPSS-CMS White Paper Committee. 2011.
48. Cohen ME, Dimick JB, Bilimoria KY, Ko CY, Richards K, Hall BL. Risk adjustment in the American College of Surgeons National Surgical Quality Improvement Program: a comparison of logistic versus hierarchical modeling. J Am Coll Surg. 2009;209(6):687-93.
49. Glance LG, Dick A, Osler TM, Li Y, Mukamel DB. Impact of changing the statistical methodology on hospital and surgeon ranking - The case of the new York state cardiac surgery report card. Medical Care. 2006;44(4):311-9.
50. Alexandrescu R, Jen MH, Bottle A, Jarman B, Aylin P. Logistic versus hierarchical modeling: an analysis of a statewide inpatient sample. J Am Coll Surg. 2011;213(3):392-401.
51. Charlson ME, Pompei P, Ales KL, MacKenzie CR. A new method of classifying prognostic comorbidity in longitudinal studies: Development and validation. Journal of Chronic Diseases. 1987;40(5):373-83.
52. Elixhauser A, Steiner C, Harris DR, Coffey RM. Comorbidity Measures for Use with Administrative Data. Medical Care. 1998;36(1):8-27.

For peer review only - http://bmjopen.bmj.com/site/about/guidelines.xhtml

53. Li P, Kim M, Doshi J. Comparison of the performance of the CMS Hierarchical Condition Category (CMS-HCC) risk adjuster with the charlson and elixhauser comorbidity measures in predicting mortality. BMC Health Services Research. 2010;10(1):245.

For peer review only

For peer review only - http://bmjopen.bmj.com/site/about/guidelines.xhtml

Table 1. Framework for performing hospital comparisons of 30-day mortality after acute myocardial infarction (AMI).

Size of hospital differences (Hospital GCE)			Benchmark value achievement		
			Full	Close	Insufficient
	VPC_H (%)	AUC_H	< 6%	6% - 8%	> 8%
Absent	0 to 1	0.50 to 0.55	A	B	C
Small	1 to 5	0.55 to 0.61	D	E	F
Moderate	5 to 10	0.61 to 0.66	G	H	I
large	10 to 20	0.66 to 0.72	J	K	L
Very large	>20	>0.72	M	N	O

For peer review only

Table 2. Characteristics of the population of 43,247 patients with a first-ever acute myocardial infarction (AMI) cared for at 68 Swedish hospitals in 2007-2009 and categorized in 10 groups by deciles of risk score for 30-day mortality (RS).

Age, *mean (SD)*	67.3 (9.0)	
Female *(%)*	33	
Number of hospitals	68	
Number of patients at the hospital, *Median (min-max)*	636 (107 - 3037)	
	Mean RS (%)	Observed mortality [illegible]
Risk Score for 30-day mortality (RS)	7.8	7.8
Decile groups of RS		
1st	1.7	0.7
2nd	2.3	1.4
3rd	2.9	2.0
4th	3.5	2.5
5th	4.2	3.7
6th	5.3	5.9
7th	6.9	8.2
8th	9.5	12.2
9th	14.0	16.1
10th	28.0	25.5

nly

For peer review only - http://bmjopen.bmj.com/site/about/guidelines.xhtml

Table 3. Multilevel modelling of 30-day mortality in 43,247 patients with a first-ever acute myocardial infarction (AMI) in 2008-2009 in Sweden. Model 1 includes only a random effect for the hospital, model 2 is a cross-classified multilevel analysis including both hospital and risk score (RS) categories as random effects.

		Model 1	Model 2
Overall 30-day mortality mean (min – max), %		8.00 (6.27 –12.21)	4.78 (3.44 –7.48)
Variance			
	Hospital	0.04 (0.02 – 0.07)	0.03 (0.01 – 0.06)
	RS category		1.96 (0.66 – 4.53)
VPC (%)			
	Hospital	1.20(0.56 – 2.22)	0.70 (0.26–1.34)
	RS category		34.13 (16.64–57.77)
AUC_H			
	Hospital	0.55 (0.55 – 0.57)	0.54 (0.53 – 0.55)
	RS category		0.77 (0.77 – 0.78)
	Total	0.55 (0.55 – 0.57)	0.78 (0.77 – 0.79)

VPC: Variance Partition Coefficient, AUC: Area Under the Receiver Operating Characteristics Curve.

Figure legends an caption:

Figure 1. Flow diagram showing the selection of patients with first diagnosis of acute myocardial infarction (AMI) 2007-2009 who were included in the study population.

Figure 2. Crude (white circles), reliability weighted (grey circles) and both reliability weighted and patient-mix adjusted (black circles and confidence intervals) differences in absolute risk of 30-day mortality between hospitals in the population of 43,247 patients with a first-ever acute myocardial infarction in 2007-2009, in Sweden.

Figure 3. Area under the Receiver Operating Characteristics Curve (AUC) constructed using the predicted probability of 30-day mortality obtained from multilevel logistic regression analysis with 47,462 patients with a discharge diagnosis of acute myocardial infarction (AMI) in 2007-2009 in Sweden. The AUC for the unadjusted hospitals was obtained from model 1 with patient nested within hospitals (Blue line). The rest of the curves are from model 2, a cross-classified multilevel model with patients nested within hospitals and categories (decile groups) of risk-score (RS) for 30-day mortality. (Red line: patient mix adjusted hospitals, Orange line: Risk score category, Green line: Total AUC)

For peer review only - http://bmjopen.bmj.com/site/about/guidelines.xhtml

For peer review only

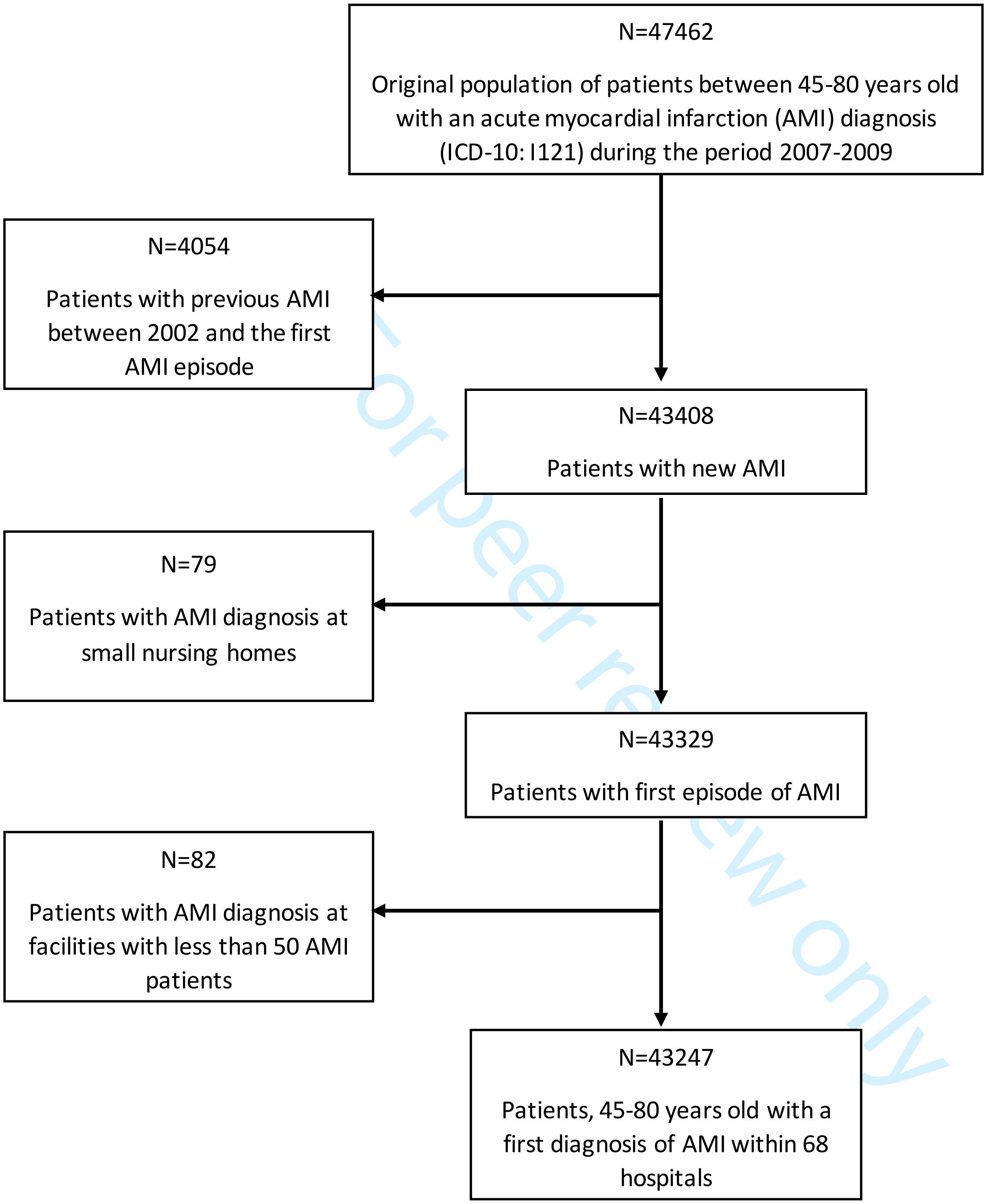

For peer review only - http://bmjopen.bmj.com/site/about/guidelines.xhtml

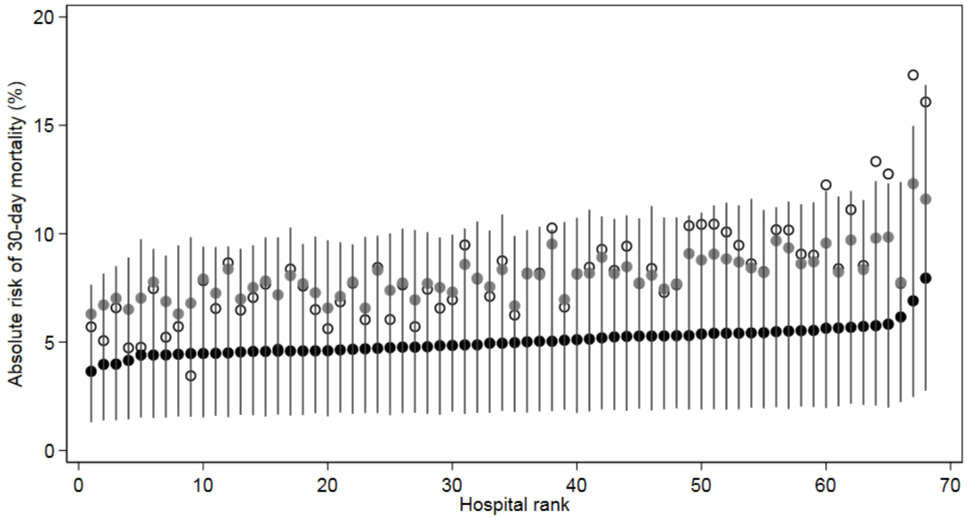

Crude (white circles), reliability weighted (grey circles) and both reliability weighted and patient-mix adjusted (black circles and confidence intervals) differences in absolute risk of 30-day mortality between hospitals in the population of 43,247 patients with a first-ever acute myocardial infarction in 2007-2009, in Sweden.

352x194mm (72 x 72 DPI)

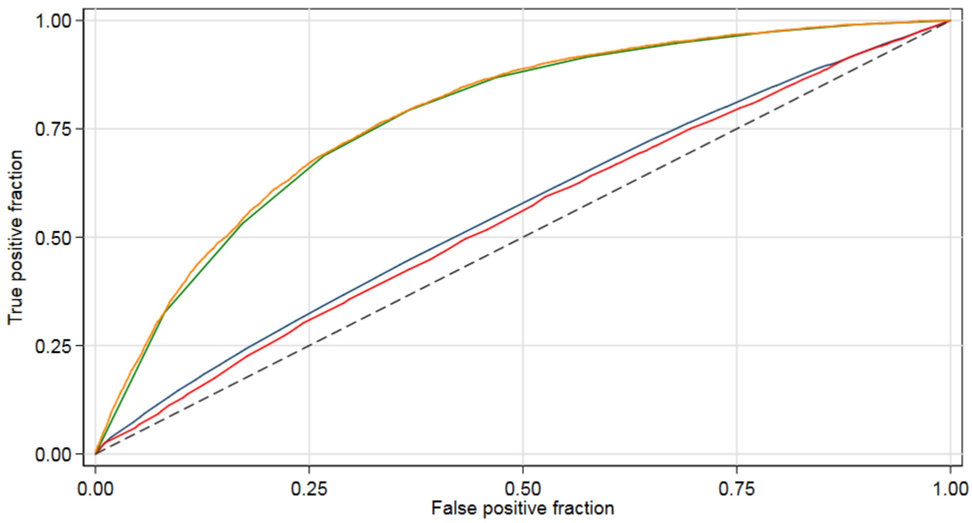

Area under the Receiver Operating Characteristics Curve (AUC) constructed using the predicted probability of 30-day mortality obtained from multilevel logistic regression analysis with 47,462 patients with a discharge diagnosis of acute myocardial infarction (AMI) in 2007-2009 in Sweden. The AUC for the unadjusted hospitals was obtained from model 1 with patient nested within hospitals (Blue line). The rest of the curves are from model 2, a cross-classified multilevel model with patients nested within hospitals and categories (decile groups) of risk-score (RS) for 30-day mortality. (Red line: patient mix adjusted hospitals, Orange line: Risk score category, Green line: Total AUC)

352x194mm (72 x 72 DPI)

hospital	cons	rscategory	stratum	proportion	numerator	denominator
1	1	1	1	0.00	0	99
1	1	2	2	0.01	1	76
1	1	3	3	0.03	2	73
1	1	4	4	0.02	1	62
1	1	5	5	0.01	1	75
1	1	6	6	0.05	4	87
1	1	7	7	0.07	8	108
1	1	8	8	0.06	7	112
1	1	9	9	0.09	11	118
1	1	10	10	0.22	26	116
2	1	1	11	0.00	0	196
2	1	2	12	0.01	1	179
2	1	3	13	0.01	1	154
2	1	4	14	0.03	4	136
2	1	5	15	0.04	7	158
2	1	6	16	0.04	7	159
2	1	7	17	0.06	9	152
2	1	8	18	0.14	27	189
2	1	9	19	0.18	34	193
2	1	10	20	0.22	42	195
3	1	1	21	0.01	3	249
3	1	2	22	0.02	4	189
3	1	3	23	0.02	3	172
3	1	4	24	0.04	7	186
3	1	5	25	0.07	10	149
3	1	6	26	0.07	11	166
3	1	7	27	0.09	12	129
3	1	8	28	0.16	20	128
3	1	9	29	0.22	21	94
3	1	10	30	0.31	29	95
4	1	1	31	0.01	1	99
4	1	2	32	0.01	1	69
4	1	3	33	0.03	2	76
4	1	4	34	0.04	3	72
4	1	5	35	0.05	4	78
4	1	6	36	0.10	9	88
4	1	7	37	0.08	7	92
4	1	8	38	0.12	10	86
4	1	9	39	0.21	19	91
4	1	10	40	0.20	25	122
5	1	1	41	0.00	0	127
5	1	2	42	0.00	0	97
5	1	3	43	0.02	2	113
5	1	4	44	0.00	0	100
5	1	5	45	0.00	0	93
5	1	6	46	0.05	5	102

For peer review only

5	1	7	47	0.05	6	110
5	1	8	48	0.06	9	145
5	1	9	49	0.07	10	140
5	1	10	50	0.24	35	147
6	1	1	51	0.00	0	26
6	1	2	52	0.00	0	22
6	1	3	53	0.03	1	30
6	1	4	54	0.05	2	37
6	1	5	55	0.09	2	22
6	1	6	56	0.05	2	37
6	1	7	57	0.06	2	32
6	1	8	58	0.13	4	32
6	1	9	59	0.11	3	28
6	1	10	60	0.22	8	37
7	1	1	61	0.00	0	26
7	1	2	62	0.00	0	19
7	1	3	63	0.06	1	18
7	1	4	64	0.00	0	23
7	1	5	65	0.12	3	26
7	1	6	66	0.07	2	27
7	1	7	67	0.00	0	26
7	1	8	68	0.14	5	37
7	1	9	69	0.28	10	36
7	1	10	70	0.22	7	32
8	1	1	71	0.00	0	157
8	1	2	72	0.02	4	167
8	1	3	73	0.01	1	147
8	1	4	74	0.05	6	133
8	1	5	75	0.04	5	135
8	1	6	76	0.07	9	123
8	1	7	77	0.03	5	154
8	1	8	78	0.13	16	126
8	1	9	79	0.09	12	139
8	1	10	80	0.29	39	133
9	1	1	81	0.00	0	27
9	1	2	82	0.00	0	23
9	1	3	83	0.00	0	14
9	1	4	84	0.00	0	7
9	1	5	85	0.07	1	14
9	1	6	86	0.00	0	15
9	1	7	87	0.00	0	7
9	1	8	88	0.08	1	13
9	1	9	89	0.00	0	11
9	1	10	90	0.21	3	14
10	1	1	91	0.00	0	62
10	1	2	92	0.03	3	93
10	1	3	93	0.01	1	84

For peer review only

10	1	4	94	0.01	1	73
10	1	5	95	0.04	3	78
10	1	6	96	0.07	6	90
10	1	7	97	0.07	6	87
10	1	8	98	0.23	14	62
10	1	9	99	0.16	11	69
10	1	10	100	0.34	18	53
11	1	1	101	0.04	1	24
11	1	2	102	0.04	1	28
11	1	3	103	0.03	1	37
11	1	4	104	0.00	0	50
11	1	5	105	0.03	1	37
11	1	6	106	0.07	2	28
11	1	7	107	0.08	3	38
11	1	8	108	0.06	2	31
11	1	9	109	0.04	1	27
11	1	10	110	0.40	6	15
12	1	1	111	0.00	0	10
12	1	2	112	0.00	0	19
12	1	3	113	0.00	0	13
12	1	4	114	0.00	0	28
12	1	5	115	0.00	0	14
12	1	6	116	0.07	2	28
12	1	7	117	0.14	3	21
12	1	8	118	0.00	0	18
12	1	9	119	0.24	4	17
12	1	10	120	0.14	2	14
13	1	1	121	0.00	0	135
13	1	2	122	0.01	1	137
13	1	3	123	0.04	5	141
13	1	4	124	0.02	2	133
13	1	5	125	0.01	2	136
13	1	6	126	0.04	5	134
13	1	7	127	0.10	12	125
13	1	8	128	0.09	8	94
13	1	9	129	0.14	14	101
13	1	10	130	0.24	21	88
14	1	1	131	0.02	1	45
14	1	2	132	0.00	0	44
14	1	3	133	0.00	0	42
14	1	4	134	0.03	2	68
14	1	5	135	0.04	3	68
14	1	6	136	0.06	3	52
14	1	7	137	0.07	4	58
14	1	8	138	0.16	10	62
14	1	9	139	0.20	12	61
14	1	10	140	0.30	24	80

For peer review only

15	1	1	141	0.03	1	32
15	1	2	142	0.00	0	17
15	1	3	143	0.00	0	35
15	1	4	144	0.08	2	25
15	1	5	145	0.11	3	28
15	1	6	146	0.05	1	19
15	1	7	147	0.04	1	25
15	1	8	148	0.17	4	23
15	1	9	149	0.23	5	22
15	1	10	150	0.17	4	24
16	1	1	151	0.00	0	74
16	1	2	152	0.01	1	94
16	1	3	153	0.00	0	85
16	1	4	154	0.00	0	100
16	1	5	155	0.03	3	91
16	1	6	156	0.12	10	82
16	1	7	157	0.06	6	93
16	1	8	158	0.10	9	86
16	1	9	159	0.16	10	64
16	1	10	160	0.20	16	80
17	1	1	161	0.00	0	18
17	1	2	162	0.03	1	35
17	1	3	163	0.00	0	28
17	1	4	164	0.06	2	34
17	1	5	165	0.07	3	41
17	1	6	166	0.11	4	38
17	1	7	167	0.05	2	39
17	1	8	168	0.09	4	43
17	1	9	169	0.14	4	29
17	1	10	170	0.36	12	33
18	1	1	171	0.07	2	27
18	1	2	172	0.04	1	23
18	1	3	173	0.00	0	24
18	1	4	174	0.00	0	26
18	1	5	175	0.00	0	30
18	1	6	176	0.08	2	26
18	1	7	177	0.07	2	27
18	1	8	178	0.06	2	34
18	1	9	179	0.08	3	39
18	1	10	180	0.08	3	40
19	1	1	181	0.00	0	34
19	1	2	182	0.00	0	40
19	1	3	183	0.00	0	38
19	1	4	184	0.08	3	39
19	1	5	185	0.00	0	45
19	1	6	186	0.00	0	37
19	1	7	187	0.08	4	53

For peer review only

19	1	8	188	0.11	6	56
19	1	9	189	0.15	9	60
19	1	10	190	0.24	13	54
20	1	1	191	0.00	0	20
20	1	2	192	0.06	1	18
20	1	3	193	0.00	0	13
20	1	4	194	0.05	1	20
20	1	5	195	0.05	1	20
20	1	6	196	0.04	1	23
20	1	7	197	0.04	1	25
20	1	8	198	0.16	4	25
20	1	9	199	0.20	4	20
20	1	10	200	0.24	4	17
21	1	1	201	0.00	0	21
21	1	2	202	0.00	0	20
21	1	3	203	0.00	0	18
21	1	4	204	0.00	0	25
21	1	5	205	0.07	2	29
21	1	6	206	0.07	2	30
21	1	7	207	0.14	3	22
21	1	8	208	0.11	3	28
21	1	9	209	0.28	8	29
21	1	10	210	0.27	7	26
22	1	1	211	0.00	0	85
22	1	2	212	0.01	1	107
22	1	3	213	0.03	4	117
22	1	4	214	0.02	3	122
22	1	5	215	0.04	4	111
22	1	6	216	0.04	4	98
22	1	7	217	0.14	13	94
22	1	8	218	0.11	11	100
22	1	9	219	0.14	13	91
22	1	10	220	0.21	17	81
23	1	1	221	0.06	1	17
23	1	2	222	0.05	1	22
23	1	3	223	0.00	0	16
23	1	4	224	0.04	1	28
23	1	5	225	0.00	0	22
23	1	6	226	0.08	2	26
23	1	7	227	0.16	6	38
23	1	8	228	0.00	0	23
23	1	9	229	0.15	4	26
23	1	10	230	0.13	3	24
24	1	1	231	0.00	0	27
24	1	2	232	0.08	3	38
24	1	3	233	0.00	0	34
24	1	4	234	0.00	0	28

For peer review only

24	1	5	235	0.03	1	33
24	1	6	236	0.12	5	43
24	1	7	237	0.05	2	37
24	1	8	238	0.13	5	39
24	1	9	239	0.17	5	29
24	1	10	240	0.24	7	29
25	1	1	241	0.01	1	101
25	1	2	242	0.02	2	100
25	1	3	243	0.03	3	94
25	1	4	244	0.03	3	113
25	1	5	245	0.07	8	109
25	1	6	246	0.09	10	113
25	1	7	247	0.10	10	104
25	1	8	248	0.16	14	89
25	1	9	249	0.21	18	85
25	1	10	250	0.20	15	76
26	1	1	251	0.00	0	41
26	1	2	252	0.00	0	43
26	1	3	253	0.00	0	48
26	1	4	254	0.00	0	47
26	1	5	255	0.04	2	56
26	1	6	256	0.02	1	44
26	1	7	257	0.04	2	53
26	1	8	258	0.09	5	57
26	1	9	259	0.10	7	68
26	1	10	260	0.32	25	79
27	1	1	261	0.00	0	9
27	1	2	262	0.00	0	17
27	1	3	263	0.00	0	17
27	1	4	264	0.05	1	21
27	1	5	265	0.00	0	18
27	1	6	266	0.00	0	20
27	1	7	267	0.07	1	14
27	1	8	268	0.00	0	26
27	1	9	269	0.14	5	35
27	1	10	270	0.29	11	38
28	1	1	271	0.06	1	16
28	1	2	272	0.00	0	20
28	1	3	273	0.00	0	17
28	1	4	274	0.00	0	19
28	1	5	275	0.00	0	33
28	1	6	276	0.06	1	16
28	1	7	277	0.12	2	17
28	1	8	278	0.09	2	23
28	1	9	279	0.21	7	34
28	1	10	280	0.11	5	46
29	1	1	281	0.00	0	145

For peer review only

29	1	2	282	0.01	1	159
29	1	3	283	0.02	2	133
29	1	4	284	0.03	3	114
29	1	5	285	0.05	6	130
29	1	6	286	0.05	7	142
29	1	7	287	0.09	13	145
29	1	8	288	0.11	17	151
29	1	9	289	0.11	17	161
29	1	10	290	0.26	44	170
30	1	1	291	0.00	1	245
30	1	2	292	0.02	5	222
30	1	3	293	0.03	7	223
30	1	4	294	0.02	5	218
30	1	5	295	0.03	5	200
30	1	6	296	0.07	16	227
30	1	7	297	0.08	13	166
30	1	8	298	0.16	27	170
30	1	9	299	0.16	33	201
30	1	10	300	0.26	44	169
31	1	1	301	0.00	0	30
31	1	2	302	0.06	1	17
31	1	3	303	0.00	0	23
31	1	4	304	0.00	0	13
31	1	5	305	0.00	0	15
31	1	6	306	0.00	0	16
31	1	7	307	0.08	1	12
31	1	8	308	0.17	4	24
31	1	9	309	0.13	2	16
31	1	10	310	0.40	10	25
32	1	1	311	0.01	1	95
32	1	2	312	0.00	0	86
32	1	3	313	0.01	1	74
32	1	4	314	0.03	2	61
32	1	5	315	0.03	2	65
32	1	6	316	0.08	6	79
32	1	7	317	0.04	3	71
32	1	8	318	0.13	10	78
32	1	9	319	0.14	13	90
32	1	10	320	0.31	26	83
33	1	1	321	0.00	0	39
33	1	2	322	0.00	0	19
33	1	3	323	0.00	0	38
33	1	4	324	0.00	0	39
33	1	5	325	0.02	1	53
33	1	6	326	0.02	1	42
33	1	7	327	0.11	5	46
33	1	8	328	0.09	5	57

For peer review only

33	1	9	329	0.16	11	70
33	1	10	330	0.23	19	82
34	1	1	331	0.02	1	61
34	1	2	332	0.02	1	49
34	1	3	333	0.00	0	35
34	1	4	334	0.04	2	57
34	1	5	335	0.02	1	52
34	1	6	336	0.05	3	60
34	1	7	337	0.09	7	75
34	1	8	338	0.12	10	84
34	1	9	339	0.13	12	92
34	1	10	340	0.28	33	117
35	1	1	341	0.00	0	26
35	1	2	342	0.03	1	36
35	1	3	343	0.08	3	38
35	1	4	344	0.00	0	34
35	1	5	345	0.03	1	29
35	1	6	346	0.08	3	39
35	1	7	347	0.10	4	42
35	1	8	348	0.09	4	45
35	1	9	349	0.24	9	38
35	1	10	350	0.33	17	51
36	1	1	351	0.01	2	331
36	1	2	352	0.01	3	316
36	1	3	353	0.02	6	319
36	1	4	354	0.03	8	305
36	1	5	355	0.03	9	321
36	1	6	356	0.06	17	306
36	1	7	357	0.08	27	319
36	1	8	358	0.13	40	303
36	1	9	359	0.21	54	256
36	1	10	360	0.26	68	261
37	1	1	361	0.04	1	26
37	1	2	362	0.00	0	30
37	1	3	363	0.03	1	36
37	1	4	364	0.00	0	35
37	1	5	365	0.02	1	43
37	1	6	366	0.07	2	30
37	1	7	367	0.05	2	39
37	1	8	368	0.14	5	37
37	1	9	369	0.13	6	47
37	1	10	370	0.25	14	56
38	1	1	371	0.00	0	96
38	1	2	372	0.02	2	88
38	1	3	373	0.02	2	97
38	1	4	374	0.02	2	96
38	1	5	375	0.07	8	113

For peer review only

38	1	6	376	0.07	7	107
38	1	7	377	0.10	12	119
38	1	8	378	0.16	20	124
38	1	9	379	0.18	27	151
38	1	10	380	0.24	36	148
39	1	1	381	0.01	1	78
39	1	2	382	0.02	2	87
39	1	3	383	0.01	1	80
39	1	4	384	0.04	3	73
39	1	5	385	0.03	3	89
39	1	6	386	0.03	3	89
39	1	7	387	0.13	8	64
39	1	8	388	0.15	15	98
39	1	9	389	0.16	14	88
39	1	10	390	0.32	24	75
40	1	1	391	0.00	0	12
40	1	2	392	0.00	0	18
40	1	3	393	0.00	0	12
40	1	4	394	0.00	0	15
40	1	5	395	0.03	1	32
40	1	6	396	0.06	1	18
40	1	7	397	0.00	0	18
40	1	8	398	0.24	5	21
40	1	9	399	0.32	7	22
40	1	10	400	0.39	11	28
41	1	1	401	0.01	1	92
41	1	2	402	0.02	2	128
41	1	3	403	0.03	3	114
41	1	4	404	0.01	1	141
41	1	5	405	0.06	7	124
41	1	6	406	0.11	14	131
41	1	7	407	0.10	14	138
41	1	8	408	0.11	16	149
41	1	9	409	0.15	23	151
41	1	10	410	0.21	24	117
42	1	1	411	0.00	0	101
42	1	2	412	0.00	0	108
42	1	3	413	0.01	2	146
42	1	4	414	0.05	6	131
42	1	5	415	0.07	9	130
42	1	6	416	0.05	6	123
42	1	7	417	0.13	14	109
42	1	8	418	0.07	5	71
42	1	9	419	0.14	12	85
42	1	10	420	0.25	17	69
43	1	1	421	0.00	0	17
43	1	2	422	0.00	0	26

For peer review only

43	1	3	423	0.00	0	24
43	1	4	424	0.07	1	14
43	1	5	425	0.00	0	23
43	1	6	426	0.00	0	17
43	1	7	427	0.04	1	23
43	1	8	428	0.30	6	20
43	1	9	429	0.15	3	20
43	1	10	430	0.23	11	48
44	1	1	431	0.00	0	11
44	1	2	432	0.00	0	18
44	1	3	433	0.00	0	14
44	1	4	434	0.00	0	7
44	1	5	435	0.00	0	14
44	1	6	436	0.00	0	17
44	1	7	437	0.14	3	21
44	1	8	438	0.22	4	18
44	1	9	439	0.39	9	23
44	1	10	440	0.27	6	22
45	1	1	441	0.01	2	137
45	1	2	442	0.02	3	183
45	1	3	443	0.04	5	141
45	1	4	444	0.03	5	153
45	1	5	445	0.03	4	135
45	1	6	446	0.06	7	124
45	1	7	447	0.09	11	117
45	1	8	448	0.06	7	117
45	1	9	449	0.16	12	76
45	1	10	450	0.28	23	81
46	1	1	451	0.00	0	16
46	1	2	452	0.00	0	20
46	1	3	453	0.05	1	19
46	1	4	454	0.07	1	14
46	1	5	455	0.11	3	28
46	1	6	456	0.12	3	26
46	1	7	457	0.22	6	27
46	1	8	458	0.15	5	33
46	1	9	459	0.24	7	29
46	1	10	460	0.43	18	42
47	1	1	461	0.00	0	8
47	1	2	462	0.06	1	16
47	1	3	463	0.10	2	20
47	1	4	464	0.06	1	17
47	1	5	465	0.00	0	20
47	1	6	466	0.16	3	19
47	1	7	467	0.03	1	31
47	1	8	468	0.11	2	19
47	1	9	469	0.29	7	24

For peer review only

47	1	10	470	0.27	8	30
48	1	1	471	0.03	2	71
48	1	2	472	0.00	0	71
48	1	3	473	0.00	0	63
48	1	4	474	0.01	1	69
48	1	5	475	0.02	1	58
48	1	6	476	0.06	4	72
48	1	7	477	0.08	6	72
48	1	8	478	0.16	15	93
48	1	9	479	0.13	11	84
48	1	10	480	0.22	15	67
49	1	1	481	0.00	0	24
49	1	2	482	0.00	0	16
49	1	3	483	0.00	0	30
49	1	4	484	0.00	0	35
49	1	5	485	0.02	1	43
49	1	6	486	0.00	0	43
49	1	7	487	0.08	4	48
49	1	8	488	0.12	5	43
49	1	9	489	0.20	7	35
49	1	10	490	0.18	6	34
50	1	1	491	0.00	0	129
50	1	2	492	0.03	3	108
50	1	3	493	0.01	2	136
50	1	4	494	0.01	1	122
50	1	5	495	0.02	2	98
50	1	6	496	0.05	5	99
50	1	7	497	0.06	7	113
50	1	8	498	0.14	10	72
50	1	9	499	0.12	12	98
50	1	10	500	0.30	21	69
51	1	1	501	0.00	0	21
51	1	2	502	0.00	0	25
51	1	3	503	0.00	0	35
51	1	4	504	0.04	1	25
51	1	5	505	0.06	2	36
51	1	6	506	0.12	5	43
51	1	7	507	0.04	1	27
51	1	8	508	0.14	3	21
51	1	9	509	0.18	8	45
51	1	10	510	0.19	8	42
52	1	1	511	0.00	0	3
52	1	2	512	0.00	0	9
52	1	3	513	0.00	0	9
52	1	4	514	0.00	0	17
52	1	5	515	0.00	0	15
52	1	6	516	0.00	0	12

For peer review only

52	1	7	517	0.22	2	9
52	1	8	518	0.08	1	12
52	1	9	519	0.00	0	10
52	1	10	520	0.18	2	11
53	1	1	521	0.00	0	7
53	1	2	522	0.00	0	12
53	1	3	523	0.00	0	18
53	1	4	524	0.08	1	13
53	1	5	525	0.00	0	13
53	1	6	526	0.00	0	23
53	1	7	527	0.08	1	12
53	1	8	528	0.07	1	14
53	1	9	529	0.14	3	21
53	1	10	530	0.07	1	14
54	1	1	531	0.03	3	97
54	1	2	532	0.02	2	122
54	1	3	533	0.03	4	117
54	1	4	534	0.02	2	119
54	1	5	535	0.03	3	107
54	1	6	536	0.03	4	122
54	1	7	537	0.10	12	119
54	1	8	538	0.11	11	101
54	1	9	539	0.19	15	81
54	1	10	540	0.32	34	106
55	1	1	541	0.00	0	27
55	1	2	542	0.00	0	53
55	1	3	543	0.00	0	30
55	1	4	544	0.00	0	38
55	1	5	545	0.00	0	37
55	1	6	546	0.00	0	38
55	1	7	547	0.08	3	39
55	1	8	548	0.10	3	29
55	1	9	549	0.13	4	31
55	1	10	550	0.19	7	37
56	1	1	551	0.02	1	47
56	1	2	552	0.00	0	38
56	1	3	553	0.04	2	51
56	1	4	554	0.02	1	51
56	1	5	555	0.00	0	37
56	1	6	556	0.06	3	47
56	1	7	557	0.06	3	47
56	1	8	558	0.14	6	42
56	1	9	559	0.18	8	44
56	1	10	560	0.22	7	32
57	1	1	561	0.01	1	69
57	1	2	562	0.00	0	73
57	1	3	563	0.01	1	87

For peer review only

57	1	4	564	0.03	3	90
57	1	5	565	0.05	4	88
57	1	6	566	0.05	4	74
57	1	7	567	0.08	7	83
57	1	8	568	0.14	11	76
57	1	9	569	0.18	12	68
57	1	10	570	0.31	25	81
58	1	1	571	0.00	0	22
58	1	2	572	0.04	1	23
58	1	3	573	0.00	0	42
58	1	4	574	0.06	2	35
58	1	5	575	0.00	0	34
58	1	6	576	0.06	2	33
58	1	7	577	0.00	0	34
58	1	8	578	0.17	7	42
58	1	9	579	0.11	3	28
58	1	10	580	0.20	6	30
59	1	1	581	0.00	0	12
59	1	2	582	0.04	1	27
59	1	3	583	0.04	1	28
59	1	4	584	0.04	1	23
59	1	5	585	0.04	1	26
59	1	6	586	0.13	2	15
59	1	7	587	0.05	1	19
59	1	8	588	0.00	0	30
59	1	9	589	0.22	4	18
59	1	10	590	0.30	7	23
60	1	1	591	0.02	1	66
60	1	2	592	0.02	1	63
60	1	3	593	0.04	3	71
60	1	4	594	0.00	0	80
60	1	5	595	0.02	1	62
60	1	6	596	0.05	3	59
60	1	7	597	0.11	6	53
60	1	8	598	0.07	3	46
60	1	9	599	0.18	8	44
60	1	10	600	0.16	7	43
61	1	1	601	0.01	1	150
61	1	2	602	0.02	2	120
61	1	3	603	0.03	4	119
61	1	4	604	0.04	4	111
61	1	5	605	0.02	2	91
61	1	6	606	0.04	3	83
61	1	7	607	0.11	10	90
61	1	8	608	0.17	13	76
61	1	9	609	0.12	12	97
61	1	10	610	0.29	23	78

For peer review only

62	1	1	611	0.00	0	31
62	1	2	612	0.04	1	25
62	1	3	613	0.00	0	31
62	1	4	614	0.00	0	35
62	1	5	615	0.06	2	36
62	1	6	616	0.06	2	34
62	1	7	617	0.11	3	28
62	1	8	618	0.08	3	37
62	1	9	619	0.13	6	48
62	1	10	620	0.20	7	35
63	1	1	621	0.00	0	17
63	1	2	622	0.00	0	18
63	1	3	623	0.10	2	21
63	1	4	624	0.04	1	23
63	1	5	625	0.04	1	26
63	1	6	626	0.00	0	20
63	1	7	627	0.05	1	21
63	1	8	628	0.18	3	17
63	1	9	629	0.11	2	18
63	1	10	630	0.18	3	17
64	1	1	631	0.00	0	31
64	1	2	632	0.00	0	25
64	1	3	633	0.13	4	32
64	1	4	634	0.15	3	20
64	1	5	635	0.19	6	31
64	1	6	636	0.07	2	27
64	1	7	637	0.16	4	25
64	1	8	638	0.26	6	23
64	1	9	639	0.35	9	26
64	1	10	640	0.47	7	15
65	1	1	641	0.00	0	32
65	1	2	642	0.00	0	22
65	1	3	643	0.00	0	24
65	1	4	644	0.00	0	28
65	1	5	645	0.00	0	32
65	1	6	646	0.05	2	44
65	1	7	647	0.00	0	29
65	1	8	648	0.12	3	25
65	1	9	649	0.24	7	29
65	1	10	650	0.14	3	22
66	1	1	651	0.00	0	18
66	1	2	652	0.00	0	30
66	1	3	653	0.00	0	16
66	1	4	654	0.10	2	21
66	1	5	655	0.00	0	28
66	1	6	656	0.05	1	20
66	1	7	657	0.04	1	25

For peer review only

66	1	8	658	0.16	5	31
66	1	9	659	0.24	7	29
66	1	10	660	0.32	10	31
67	1	1	661	0.00	0	17
67	1	2	662	0.00	0	12
67	1	3	663	0.05	1	21
67	1	4	664	0.00	0	16
67	1	5	665	0.00	0	11
67	1	6	666	0.17	2	12
67	1	7	667	0.00	0	15
67	1	8	668	0.11	2	19
67	1	9	669	0.21	4	19
67	1	10	670	0.38	8	21
68	1	1	671	0.00	0	70
68	1	2	672	0.00	0	48
68	1	3	673	0.00	0	57
68	1	4	674	0.03	2	61
68	1	5	675	0.04	2	51
68	1	6	676	0.07	3	45
68	1	7	677	0.13	7	55
68	1	8	678	0.14	9	66
68	1	9	679	0.20	11	56
68	1	10	680	0.31	17	54

For peer review only

```
********************************************************************************
* A Cross-Classified  Multilevel Analysis of Individual Heterogeneity and
* Discriminatory Accuracy (MAIHDA) to evaluate hospital performance: The case of
* Hospital Differences in Patient Survival after Acute Myocardial Infarction
********************************************************************************

* Set the working directory
cd "Y:\manuscripts-4-under-review\rodriguez-lopez2020bmiopen\2020-07-16"
// Edit the above folder directory to where the dataset is saved

* Set MLwiN path
global MLwiN_path "C:\Program Files\MLwiN v3.05\mlwin.exe"

* Set the output format of coefficient tables to two decimal places
set cformat %9.2f

********************************************************************************
* Model 1
********************************************************************************

* Load the data
use "Supplemental Material 1_AMIdatabase.dta", clear

* Fit model 1 by MQL1 to get parameter starting values
runmlwin proportion cons, ///
  level4(hospital: cons) ///
  level3(rscategory:) ///
```

For peer review only

```
  level2(stratum:) ///
  level1(stratum:) ///
  discrete(distribution(binomial) link(logit) denominator(denom) mql1) ///
  nopause

* Fit model 1 by MCMC to get final parameter estimates
runmlwin proportion cons, ///
  level4(hospital: cons, residuals(v, savechains("m1v.dta", replace))) ///
  level3(rscategory:) ///
  level2(stratum:) ///
  level1(stratum:) ///
  discrete(distribution(binomial) link(logit) denominator(denom)) ///
  mcmc(burnin(5000) chain(10000) thinning(10) savechains("m1b.dta", replace)) ///
  initsprevious ///
  nopause

* Model predictions based on means of MCMC chains
rename v0* m1v*
predict m1xb, xb
generate m1xbv = m1xb + m1v
generate m1pxbv = invlogit(m1xbv)

* Compress and save the data
compress
save "m1.dta", replace

* VPC based on full MCMC chains
use "m1b.dta", clear
rename RP4_var_cons_ sigma2v
```

```
generate m1vpc_v = 100*sigma2v/(sigma2v + _pi^2/3)

mcmcsum m1vpc_v, variable


* Model predictions based on full MCMC chains

use "m1b.dta", clear

isid iteration

keep iteration FP1_cons

rename FP1_cons beta0

merge 1:m iteration using "m1v.dta", assert(match) keep(match) keepusing(hospital value)
nogenerate

rename value v

generate m1pxbv = 100*invlogit(beta + v)

gcollapse (mean) m1pxbvmn = m1pxbv (p2.5) m1pxbvlo = m1pxbv (p97.5) m1pxbvhi = m1pxbv,
by(hospital)

compress

save "m1pxbv", replace


* Residuals H - Histogram of the predicted hospital random effects based on means of MCMC
chains

use "m1.dta", clear

egen tag_hospital = tag(hospital)

histogram m1v if tag_hospital==1, width(0.1) start(-0.35) frequency addlabel ///

  normal ///

  kdensity kdenopts(lpattern(dash)) ///

  ytitle("Number of hospitals") ///

  ylabel(, angle(horizontal)) ///

  xtitle("Predicted hospital random effect") ///

  legend(on order(3 "Normal curve" 2 "Smoothed histogram") position(2)  ring(0)) ///

  scheme(s1mono)  ///

  xsize(7.25) ysize(4)
```

For peer review only

```
graph export "Figure S1 - Model 1 - Histogram of the predicted hospital random effects.png", replace width(1000)

* ROC AUC based on means of MCMC chains
use "m1.dta", clear
keep hospital rscategory stratum numerator denominator m1xbv
expand denominator
sort stratum
generate patient = _n
order patient, after(stratum)
bysort stratum (patient): generate y = (numerator>=_n)
generate m1pxbv = invlogit(m1xbv)
roctab y m1pxbv
compress
save "m1roc.dta", replace

********************************************************************************
* Model 2
********************************************************************************

* Load the data
use "Supplemental Material 1_AMIdatabase.dta", clear

* Fit model 2 by MQL1 to get parameter starting values
runmlwin prop cons, ///
  level4(hospital: cons) ///
  level3(rscategory: cons) ///
```

```
  level2(stratum:) ///
  level1(stratum:) ///
  discrete(distribution(binomial) link(logit) denominator(denom)) ///
  nopause

* Fit model 2 by MCMC to get final parameter estimates
runmlwin prop cons, ///
  level4(hospital: cons, residuals(v, savechains("m2v.dta", replace))) ///
  level3(rscategory: cons, residuals(u, savechains("m2u.dta", replace))) ///
  level2(stratum:) ///
  level1(stratum:) ///
  discrete(distribution(binomial) link(logit) denominator(denom)) ///
  mcmc(cc burnin(5000) chain(10000) thinning(10) hc(3) savechains("m2b.dta", replace)) ///
  initsprevious nopause
rename v0* m2v*
rename u0* m2u*

* Model predictions based on means of MCMC chains
predict m2xb, xb
generate m2xbv = m2xb + m2v
generate m2xbu = m2xb + m2u
generate m2xbvu = m2xb + m2v + m2u
generate m2pxbv = invlogit(m2xbv)
generate m2pxbu = invlogit(m2xbu)
generate m2pxbvu = invlogit(m2xbvu)
generate m2pxbvlo = invlogit(m2xbv - 1.96*m2vse)
generate m2pxbvhi = invlogit(m2xbv + 1.96*m2vse)

* Compress and save the data
```

For peer review only

```
compress

save "m2.dta", replace


* Model predictions based on full MCMC chains

use "m2b.dta", clear

isid iteration

keep iteration FP1_cons

rename FP1_cons beta0

merge 1:m iteration using "m2v.dta", assert(match) keep(match) keepusing(hospital value)
nogenerate

rename value v

generate m2pxbv = 100*invlogit(beta + v)

gcollapse (mean) m2pxbvmn = m2pxbv (p2.5) m2pxbvlo = m2pxbv (p97.5) m2pxbvhi = m2pxbv,
by(hospital)

compress

save "m2pxbv", replace


* VPC based on full MCMC chains

use "m2b.dta", clear

rename RP4_var_cons_ sigma2v

rename RP3_var_cons_ sigma2u

generate m1vpc_v = 100*sigma2v/(sigma2v + sigma2u + _pi^2/3)

generate m1vpc_u = 100*sigma2u/(sigma2v + sigma2u + _pi^2/3)

mcmcsum m1vpc_v m1vpc_u, variable


* Residuals H - Histogram of the predicted hospital random effects based on means of MCMC
chains

use "m2.dta", clear

egen tag_hospital = tag(hospital)

histogram m2v if tag_hospital==1, width(0.1) start(-0.40) frequency addlabel ///
```

For peer review only

```
  normal ///
  kdensity kdenopts(lpattern(dash)) ///
  ytitle("Number of hospitals") ///
  ylabel(, angle(horizontal)) ///
  xtitle("Predicted hospital random effect") ///
  legend(on order(3 "Normal curve" 2 "Smoothed histogram") position(2) ring(0)) ///
  scheme(s1mono) ///
  xsize(7.25) ysize(4)
graph export "Figure S2 - Model 2 - Histogram of the predicted hospital random effects.png", replace width(1000)


* Residuals RS - Histogram of the predicted hospital random effects based on means of MCMC chains
use "m2.dta", clear
egen tag_rscategory = tag(rscategory)
histogram m2u if tag_rscategory==1, width(1) start(-3) frequency addlabel ///
  normal ///
  kdensity kdenopts(lpattern(dash)) ///
  ytitle("Number of RS categories") ///
  ylabel(, angle(horizontal)) ///
  xtitle("Predicted RS category random effect") ///
  legend(on order(3 "Normal curve" 2 "Smoothed histogram") position(2) ring(0)) ///
  scheme(s1mono) ///
  xsize(7.25) ysize(4)
graph export "Figure S3 - Model 2 - Histogram of the predicted RS category random effects.png", replace width(1000)


* Figure 2 based on full MCMC chains
use "m1.dta", replace
collapse (sum) numerator denominator, by(hospital)
```

For peer review only

```
isid hospital
generate percentage = 100*(numerator/denominator)
merge 1:m hospital using "m1pxbv.dta", assert(match) keep(match) nogenerate
merge 1:m hospital using "m2pxbv.dta", assert(match) keep(match) nogenerate
sort m2pxbvmn
generate rank = _n
twoway ///
  (scatter percentage rank, msymbol(circle) mfcolor(white) mlcolor(black)) ///
  (scatter m1pxbvmn rank, mcolor(gray) msymbol(circle)) ///
  (scatter m2pxbvmn rank, mcolor(black) msymbol(circle)) ///
  (rspike m2pxbvhi m2pxbvlo rank), ///
  ytitle("Absolute risk of 30-day mortality (%)") ///
  ylabel(, angle(horizontal)) ///
  xtitle("Hospital rank") ///
  xlabel(0(10)70) ///
  legend(off) ///
  scheme(s1mono) ///
  xsize(7.25) ysize(4)
graph export "Figure 2.png", replace width(1000)

* ROC AUC based on means of MCMC chains
use "m2.dta", clear
keep hospital rscategory stratum numerator denominator m2xbv m2xbu m2xbvu
expand denominator
sort stratum
generate patient = _n
order patient, after(stratum)
bysort stratum (patient): generate y = (numerator>=_n)
generate m2pxbv = invlogit(m2xbv)
```

```
generate m2pxbu = invlogit(m2xbu)

generate m2pxbvu = invlogit(m2xbvu)

roctab y m2pxbv

roctab y m2pxbu

roctab y m2pxbvu

compress

save "m2roc.dta", replace


* Figure 3 - Area under the Receiver Operating Characteristics Curve based on means of MCMC chains

use "m1roc.dta", clear

merge 1:1 patient using "m2roc.dta", assert(match) keep(match) nogenerate

roccomp y m1pxbv m2pxbv m2xbu m2xbvu, ///

 graph ///

 plot1opts(recast(line) lcolor(navy)) ///

 plot2opts(recast(line) lcolor(red)) ///

 plot3opts(recast(line) lcolor(green)) ///

 plot4opts(recast(line) lcolor(orange)) ///

 rlopts(lpattern(dash)) ///

 ytitle("True positive fraction") ///

 ylabel(, angle(horizontal)) ///

 xtitle("False positive fraction") ///

 legend(off) ///

 scheme(s1mono) ///

 xsize(7.25) ysize(4)

graph export "Figure 3 - Area under the Receiver Operating Characteristics Curve.png", replace width(1000)
```

For peer review only

```
*****************************************************************************
* Model 3 Interaction Effect
*****************************************************************************

* Load the data
use "Supplemental Material 1_AMIdatabase.dta", clear

* Sort hte data
sort hospital rscategory stratum

* Fit model 3 by MQL1 to get parameter starting values
runmlwin prop cons, ///
  level4(hospital: cons) ///
  level3(rscategory: cons) ///
  level2(stratum: cons) ///
  level1(stratum:) ///
  discrete(distribution(binomial) link(logit) denominator(denom)) ///
  nopause

* Fit model 3 by MCMC to get final parameter estimates
runmlwin prop cons, ///
  level4(hospital: cons) ///
  level3(rscategory: cons) ///
  level2(stratum: cons) ///
  level1(stratum:) ///
  discrete(distribution(binomial) link(logit) denominator(denom)) ///
  mcmc(cc burnin(5000) chain(10000) thinning(10) hc(3)) ///
  initsprevious nopause cformat(%9.3f)
```

For peer review only

```
********************************************************************************
* Traditional Random intercept model
********************************************************************************


* Load the data
use "Supplemental Material 1_AMIdatabase.dta", clear

* Fit random intercept by MQL1 to get parameter starting values
tab     rscategory,     gen(risk)
runmlwin proportion cons risk1-risk10, ///
  level4(hospital: cons) ///
  level3(rscategory:) ///
  level2(stratum:) ///
  level1(stratum:) ///
  discrete(distribution(binomial) link(logit) denominator(denom) mql1) ///
  nopause

* Fit random intercept by MCMC to get final parameter estimates
runmlwin proportion cons risk1-risk10, ///
  level4(hospital: cons, residuals(v, savechains("m1v.dta", replace))) ///
  level3(rscategory:) ///
  level2(stratum:) ///
  level1(stratum:) ///
  discrete(distribution(binomial) link(logit) denominator(denom)) ///
  mcmc(burnin(5000) chain(10000) thinning(10) savechains("m1b.dta", replace)) ///
  initsprevious ///
  nopause
```

For peer review only

```
* ROC AUC based on means of MCMC chains
predict pr
gen prh=pr+v0

*Expand databse
expand denom
sort stratum
by stratum,sort: gen num=_n
by stratum: gen y=0 if numer<num
replace y=1 if y==.
gen Ph=invlogit(prh)

disp _N
gen prop =denom/43247
gen weight=int(1/prop)

roctab y Ph
roctab y Ph [fw=weight]

*******************************************************************************
exit
```

For peer review only

```
#------------------------------------------------------------------------------
# A Cross-Classified  Multilevel Analysis of Individual Heterogeneity and
# Discriminatory Accuracy (MAIHDA) to evaluate hospital performance: The case of
# Hospital Differences in Patient Survival after Acute Myocardial Infarction
#------------------------------------------------------------------------------

# Load required packages
library(R2MLwiN)

# Set MLwiN path
options(MLwiN_path = 'C:/Program Files/MLwiN v3.05/')

# Load the dataset
mydata <- read.csv("Supplemental Material 1_AMIdatabase.csv")



#------------------------------------------------------------------------------
# Model 1
#------------------------------------------------------------------------------

# Fit Model 1
model1 <- runMLwiN(logit(proportion, denominator) ~ 1 + (1 | hospital),
              D = "Binomial",
              estoptions = list(EstM = 1,
                         mcmcMeth = list(burnin  = 5000,
                                   iterations = 10000)),
              data = mydata)
model1
```

For peer review only

```
#------------------------------------------------------------------------------
# Model 2
#------------------------------------------------------------------------------

# Fit Model 2
model2 <- runMLwiN(logit(proportion, denominator) ~ 1 + (1 | hospital)
                + (1 | rscategory),
                 D = "Binomial",
                 estoptions = list(EstM = 1, xc = TRUE,
                           mcmcMeth = list(burnin  = 5000,
                                   iterations = 10000),
                           mcmcOptions = list(hcen = 2)),
                 data = mydata)
model2

#---------------------------------------------------------------------------
```

For peer review only

```
hospital,cons,rscategory,stratum,proportion,numerator,denominator
1,1,1,1,0,0,99
1,1,2,2,.013157895,1,76
1,1,3,3,.02739726,2,73
1,1,4,4,.016129032,1,62
1,1,5,5,.013333334,1,75
1,1,6,6,.045977011,4,87
1,1,7,7,.074074075,8,108
1,1,8,8,.0625,7,112
1,1,9,9,.093220338,11,118
1,1,10,10,.22413793,26,116
2,1,1,11,0,0,196
2,1,2,12,.005586592,1,179
2,1,3,13,.0064935065,1,154
2,1,4,14,.029411765,4,136
2,1,5,15,.044303797,7,158
2,1,6,16,.044025157,7,159
2,1,7,17,.059210528,9,152
2,1,8,18,.14285715,27,189
2,1,9,19,.1761658,34,193
2,1,10,20,.21538462,42,195
3,1,1,21,.012048192,3,249
3,1,2,22,.021164021,4,189
3,1,3,23,.017441861,3,172
3,1,4,24,.03763441,7,186
3,1,5,25,.067114092,10,149
3,1,6,26,.066265061,11,166
3,1,7,27,.093023255,12,129
3,1,8,28,.15625,20,128
3,1,9,29,.22340426,21,94
3,1,10,30,.30526316,29,95
4,1,1,31,.01010101,1,99
4,1,2,32,.014492754,1,69
4,1,3,33,.02631579,2,76
4,1,4,34,.041666668,3,72
4,1,5,35,.051282052,4,78
4,1,6,36,.10227273,9,88
4,1,7,37,.076086953,7,92
4,1,8,38,.11627907,10,86
4,1,9,39,.20879121,19,91
4,1,10,40,.20491803,25,122
5,1,1,41,0,0,127
5,1,2,42,0,0,97
5,1,3,43,.017699115,2,113
5,1,4,44,0,0,100
5,1,5,45,0,0,93
5,1,6,46,.049019609,5,102
```

For peer review only

5,1,7,47,.054545455,6,110
5,1,8,48,.062068965,9,145
5,1,9,49,.071428575,10,140
5,1,10,50,.23809524,35,147
6,1,1,51,0,0,26
6,1,2,52,0,0,22
6,1,3,53,.033333335,1,30
6,1,4,54,.054054055,2,37
6,1,5,55,.090909094,2,22
6,1,6,56,.054054055,2,37
6,1,7,57,.0625,2,32
6,1,8,58,.125,4,32
6,1,9,59,.10714286,3,28
6,1,10,60,.21621622,8,37
7,1,1,61,0,0,26
7,1,2,62,0,0,19
7,1,3,63,.055555556,1,18
7,1,4,64,0,0,23
7,1,5,65,.11538462,3,26
7,1,6,66,.074074075,2,27
7,1,7,67,0,0,26
7,1,8,68,.13513513,5,37
7,1,9,69,.27777779,10,36
7,1,10,70,.21875,7,32
8,1,1,71,0,0,157
8,1,2,72,.023952097,4,167
8,1,3,73,.0068027209,1,147
8,1,4,74,.045112781,6,133
8,1,5,75,.037037037,5,135
8,1,6,76,.073170729,9,123
8,1,7,77,.032467533,5,154
8,1,8,78,.12698413,16,126
8,1,9,79,.086330935,12,139
8,1,10,80,.2932331,39,133
9,1,1,81,0,0,27
9,1,2,82,0,0,23
9,1,3,83,0,0,14
9,1,4,84,0,0,7
9,1,5,85,.071428575,1,14
9,1,6,86,0,0,15
9,1,7,87,0,0,7
9,1,8,88,.07692308,1,13
9,1,9,89,0,0,11
9,1,10,90,.21428572,3,14
10,1,1,91,0,0,62
10,1,2,92,.032258064,3,93
10,1,3,93,.011904762,1,84

For peer review only

10,1,4,94,.01369863,1,73
10,1,5,95,.03846154,3,78
10,1,6,96,.06666667,6,90
10,1,7,97,.068965517,6,87
10,1,8,98,.22580644,14,62
10,1,9,99,.1594203,11,69
10,1,10,100,.33962265,18,53
11,1,1,101,.041666668,1,24
11,1,2,102,.035714287,1,28
11,1,3,103,.027027028,1,37
11,1,4,104,0,0,50
11,1,5,105,.027027028,1,37
11,1,6,106,.071428575,2,28
11,1,7,107,.078947365,3,38
11,1,8,108,.064516127,2,31
11,1,9,109,.037037037,1,27
11,1,10,110,.40000001,6,15
12,1,1,111,0,0,10
12,1,2,112,0,0,19
12,1,3,113,0,0,13
12,1,4,114,0,0,28
12,1,5,115,0,0,14
12,1,6,116,.071428575,2,28
12,1,7,117,.14285715,3,21
12,1,8,118,0,0,18
12,1,9,119,.23529412,4,17
12,1,10,120,.14285715,2,14
13,1,1,121,0,0,135
13,1,2,122,.00729927,1,137
13,1,3,123,.035460994,5,141
13,1,4,124,.015037594,2,133
13,1,5,125,.014705882,2,136
13,1,6,126,.037313432,5,134
13,1,7,127,.096000001,12,125
13,1,8,128,.08510638,8,94
13,1,9,129,.13861386,14,101
13,1,10,130,.23863636,21,88
14,1,1,131,.022222223,1,45
14,1,2,132,0,0,44
14,1,3,133,0,0,42
14,1,4,134,.029411765,2,68
14,1,5,135,.044117648,3,68
14,1,6,136,.057692308,3,52
14,1,7,137,.068965517,4,58
14,1,8,138,.16129032,10,62
14,1,9,139,.19672132,12,61
14,1,10,140,.30000001,24,80

For peer review only

15,1,1,141,.03125,1,32
15,1,2,142,0,0,17
15,1,3,143,0,0,35
15,1,4,144,.079999998,2,25
15,1,5,145,.10714286,3,28
15,1,6,146,.052631579,1,19
15,1,7,147,.039999999,1,25
15,1,8,148,.17391305,4,23
15,1,9,149,.22727273,5,22
15,1,10,150,.16666667,4,24
16,1,1,151,0,0,74
16,1,2,152,.010638298,1,94
16,1,3,153,0,0,85
16,1,4,154,0,0,100
16,1,5,155,.032967035,3,91
16,1,6,156,.12195122,10,82
16,1,7,157,.064516127,6,93
16,1,8,158,.10465116,9,86
16,1,9,159,.15625,10,64
16,1,10,160,.2,16,80
17,1,1,161,0,0,18
17,1,2,162,.028571429,1,35
17,1,3,163,0,0,28
17,1,4,164,.05882353,2,34
17,1,5,165,.073170729,3,41
17,1,6,166,.10526316,4,38
17,1,7,167,.051282052,2,39
17,1,8,168,.093023255,4,43
17,1,9,169,.13793103,4,29
17,1,10,170,.36363637,12,33
18,1,1,171,.074074075,2,27
18,1,2,172,.043478262,1,23
18,1,3,173,0,0,24
18,1,4,174,0,0,26
18,1,5,175,0,0,30
18,1,6,176,.07692308,2,26
18,1,7,177,.074074075,2,27
18,1,8,178,.05882353,2,34
18,1,9,179,.07692308,3,39
18,1,10,180,.075000003,3,40
19,1,1,181,0,0,34
19,1,2,182,0,0,40
19,1,3,183,0,0,38
19,1,4,184,.07692308,3,39
19,1,5,185,0,0,45
19,1,6,186,0,0,37
19,1,7,187,.075471699,4,53

For peer review only

19,1,8,188,.10714286,6,56
19,1,9,189,.15000001,9,60
19,1,10,190,.24074075,13,54
20,1,1,191,0,0,20
20,1,2,192,.055555556,1,18
20,1,3,193,0,0,13
20,1,4,194,.050000001,1,20
20,1,5,195,.050000001,1,20
20,1,6,196,.043478262,1,23
20,1,7,197,.039999999,1,25
20,1,8,198,.16,4,25
20,1,9,199,.2,4,20
20,1,10,200,.23529412,4,17
21,1,1,201,0,0,21
21,1,2,202,0,0,20
21,1,3,203,0,0,18
21,1,4,204,0,0,25
21,1,5,205,.068965517,2,29
21,1,6,206,.06666667,2,30
21,1,7,207,.13636364,3,22
21,1,8,208,.10714286,3,28
21,1,9,209,.27586207,8,29
21,1,10,210,.26923078,7,26
22,1,1,211,0,0,85
22,1,2,212,.0093457941,1,107
22,1,3,213,.034188036,4,117
22,1,4,214,.024590164,3,122
22,1,5,215,.036036037,4,111
22,1,6,216,.040816326,4,98
22,1,7,217,.13829787,13,94
22,1,8,218,.11,11,100
22,1,9,219,.14285715,13,91
22,1,10,220,.20987654,17,81
23,1,1,221,.05882353,1,17
23,1,2,222,.045454547,1,22
23,1,3,223,0,0,16
23,1,4,224,.035714287,1,28
23,1,5,225,0,0,22
23,1,6,226,.07692308,2,26
23,1,7,227,.15789473,6,38
23,1,8,228,0,0,23
23,1,9,229,.15384616,4,26
23,1,10,230,.125,3,24
24,1,1,231,0,0,27
24,1,2,232,.078947365,3,38
24,1,3,233,0,0,34
24,1,4,234,0,0,28

For peer review only

24,1,5,235,.030303031,1,33
24,1,6,236,.11627907,5,43
24,1,7,237,.054054055,2,37
24,1,8,238,.12820514,5,39
24,1,9,239,.1724138,5,29
24,1,10,240,.24137931,7,29
25,1,1,241,.0099009899,1,101
25,1,2,242,.02,2,100
25,1,3,243,.031914894,3,94
25,1,4,244,.026548672,3,113
25,1,5,245,.073394492,8,109
25,1,6,246,.088495575,10,113
25,1,7,247,.096153848,10,104
25,1,8,248,.15730338,14,89
25,1,9,249,.21176471,18,85
25,1,10,250,.19736843,15,76
26,1,1,251,0,0,41
26,1,2,252,0,0,43
26,1,3,253,0,0,48
26,1,4,254,0,0,47
26,1,5,255,.035714287,2,56
26,1,6,256,.022727273,1,44
26,1,7,257,.03773585,2,53
26,1,8,258,.087719299,5,57
26,1,9,259,.10294118,7,68
26,1,10,260,.31645569,25,79
27,1,1,261,0,0,9
27,1,2,262,0,0,17
27,1,3,263,0,0,17
27,1,4,264,.047619049,1,21
27,1,5,265,0,0,18
27,1,6,266,0,0,20
27,1,7,267,.071428575,1,14
27,1,8,268,0,0,26
27,1,9,269,.14285715,5,35
27,1,10,270,.28947368,11,38
28,1,1,271,.0625,1,16
28,1,2,272,0,0,20
28,1,3,273,0,0,17
28,1,4,274,0,0,19
28,1,5,275,0,0,33
28,1,6,276,.0625,1,16
28,1,7,277,.11764706,2,17
28,1,8,278,.086956523,2,23
28,1,9,279,.20588236,7,34
28,1,10,280,.10869565,5,46
29,1,1,281,0,0,145

For peer review only

29,1,2,282,.006289308,1,159
29,1,3,283,.015037594,2,133
29,1,4,284,.02631579,3,114
29,1,5,285,.046153847,6,130
29,1,6,286,.049295776,7,142
29,1,7,287,.089655176,13,145
29,1,8,288,.11258278,17,151
29,1,9,289,.10559006,17,161
29,1,10,290,.25882354,44,170
30,1,1,291,.0040816325,1,245
30,1,2,292,.022522522,5,222
30,1,3,293,.031390134,7,223
30,1,4,294,.02293578,5,218
30,1,5,295,.025,5,200
30,1,6,296,.070484579,16,227
30,1,7,297,.078313254,13,166
30,1,8,298,.15882353,27,170
30,1,9,299,.1641791,33,201
30,1,10,300,.26035503,44,169
31,1,1,301,0,0,30
31,1,2,302,.05882353,1,17
31,1,3,303,0,0,23
31,1,4,304,0,0,13
31,1,5,305,0,0,15
31,1,6,306,0,0,16
31,1,7,307,.083333336,1,12
31,1,8,308,.16666667,4,24
31,1,9,309,.125,2,16
31,1,10,310,.40000001,10,25
32,1,1,311,.010526316,1,95
32,1,2,312,0,0,86
32,1,3,313,.013513514,1,74
32,1,4,314,.032786883,2,61
32,1,5,315,.030769231,2,65
32,1,6,316,.075949363,6,79
32,1,7,317,.04225352,3,71
32,1,8,318,.12820514,10,78
32,1,9,319,.14444445,13,90
32,1,10,320,.31325302,26,83
33,1,1,321,0,0,39
33,1,2,322,0,0,19
33,1,3,323,0,0,38
33,1,4,324,0,0,39
33,1,5,325,.018867925,1,53
33,1,6,326,.023809524,1,42
33,1,7,327,.10869565,5,46
33,1,8,328,.087719299,5,57

For peer review only

33,1,9,329,.15714286,11,70
33,1,10,330,.23170732,19,82
34,1,1,331,.016393442,1,61
34,1,2,332,.020408163,1,49
34,1,3,333,0,0,35
34,1,4,334,.03508772,2,57
34,1,5,335,.01923077,1,52
34,1,6,336,.050000001,3,60
34,1,7,337,.093333334,7,75
34,1,8,338,.11904762,10,84
34,1,9,339,.13043478,12,92
34,1,10,340,.2820513,33,117
35,1,1,341,0,0,26
35,1,2,342,.027777778,1,36
35,1,3,343,.078947365,3,38
35,1,4,344,0,0,34
35,1,5,345,.034482758,1,29
35,1,6,346,.07692308,3,39
35,1,7,347,.095238097,4,42
35,1,8,348,.088888891,4,45
35,1,9,349,.23684211,9,38
35,1,10,350,.33333334,17,51
36,1,1,351,.0060422961,2,331
36,1,2,352,.0094936704,3,316
36,1,3,353,.018808777,6,319
36,1,4,354,.026229508,8,305
36,1,5,355,.028037382,9,321
36,1,6,356,.055555556,17,306
36,1,7,357,.084639497,27,319
36,1,8,358,.1320132,40,303
36,1,9,359,.2109375,54,256
36,1,10,360,.2605364,68,261
37,1,1,361,.03846154,1,26
37,1,2,362,0,0,30
37,1,3,363,.027777778,1,36
37,1,4,364,0,0,35
37,1,5,365,.023255814,1,43
37,1,6,366,.06666667,2,30
37,1,7,367,.051282052,2,39
37,1,8,368,.13513513,5,37
37,1,9,369,.12765957,6,47
37,1,10,370,.25,14,56
38,1,1,371,0,0,96
38,1,2,372,.022727273,2,88
38,1,3,373,.020618556,2,97
38,1,4,374,.020833334,2,96
38,1,5,375,.07079646,8,113

For peer review only

38,1,6,376,.065420561,7,107
38,1,7,377,.10084034,12,119
38,1,8,378,.16129032,20,124
38,1,9,379,.17880794,27,151
38,1,10,380,.24324325,36,148
39,1,1,381,.012820513,1,78
39,1,2,382,.022988506,2,87
39,1,3,383,.0125,1,80
39,1,4,384,.04109589,3,73
39,1,5,385,.033707865,3,89
39,1,6,386,.033707865,3,89
39,1,7,387,.125,8,64
39,1,8,388,.15306123,15,98
39,1,9,389,.15909091,14,88
39,1,10,390,.31999999,24,75
40,1,1,391,0,0,12
40,1,2,392,0,0,18
40,1,3,393,0,0,12
40,1,4,394,0,0,15
40,1,5,395,.03125,1,32
40,1,6,396,.055555556,1,18
40,1,7,397,0,0,18
40,1,8,398,.23809524,5,21
40,1,9,399,.31818181,7,22
40,1,10,400,.39285713,11,28
41,1,1,401,.010869565,1,92
41,1,2,402,.015625,2,128
41,1,3,403,.02631579,3,114
41,1,4,404,.0070921984,1,141
41,1,5,405,.056451611,7,124
41,1,6,406,.10687023,14,131
41,1,7,407,.10144927,14,138
41,1,8,408,.10738255,16,149
41,1,9,409,.15231788,23,151
41,1,10,410,.20512821,24,117
42,1,1,411,0,0,101
42,1,2,412,0,0,108
42,1,3,413,.01369863,2,146
42,1,4,414,.045801528,6,131
42,1,5,415,.069230773,9,130
42,1,6,416,.048780486,6,123
42,1,7,417,.12844037,14,109
42,1,8,418,.070422538,5,71
42,1,9,419,.14117648,12,85
42,1,10,420,.24637681,17,69
43,1,1,421,0,0,17
43,1,2,422,0,0,26

For peer review only

43,1,3,423,0,0,24
43,1,4,424,.071428575,1,14
43,1,5,425,0,0,23
43,1,6,426,0,0,17
43,1,7,427,.043478262,1,23
43,1,8,428,.30000001,6,20
43,1,9,429,.15000001,3,20
43,1,10,430,.22916667,11,48
44,1,1,431,0,0,11
44,1,2,432,0,0,18
44,1,3,433,0,0,14
44,1,4,434,0,0,7
44,1,5,435,0,0,14
44,1,6,436,0,0,17
44,1,7,437,.14285715,3,21
44,1,8,438,.22222222,4,18
44,1,9,439,.39130434,9,23
44,1,10,440,.27272728,6,22
45,1,1,441,.01459854,2,137
45,1,2,442,.016393442,3,183
45,1,3,443,.035460994,5,141
45,1,4,444,.03267974,5,153
45,1,5,445,.029629629,4,135
45,1,6,446,.056451611,7,124
45,1,7,447,.094017096,11,117
45,1,8,448,.05982906,7,117
45,1,9,449,.15789473,12,76
45,1,10,450,.28395063,23,81
46,1,1,451,0,0,16
46,1,2,452,0,0,20
46,1,3,453,.052631579,1,19
46,1,4,454,.071428575,1,14
46,1,5,455,.10714286,3,28
46,1,6,456,.11538462,3,26
46,1,7,457,.22222222,6,27
46,1,8,458,.15151516,5,33
46,1,9,459,.24137931,7,29
46,1,10,460,.42857143,18,42
47,1,1,461,0,0,8
47,1,2,462,.0625,1,16
47,1,3,463,.1,2,20
47,1,4,464,.05882353,1,17
47,1,5,465,0,0,20
47,1,6,466,.15789473,3,19
47,1,7,467,.032258064,1,31
47,1,8,468,.10526316,2,19
47,1,9,469,.29166666,7,24

For peer review only

47,1,10,470,.26666668,8,30
48,1,1,471,.028169014,2,71
48,1,2,472,0,0,71
48,1,3,473,0,0,63
48,1,4,474,.014492754,1,69
48,1,5,475,.017241379,1,58
48,1,6,476,.055555556,4,72
48,1,7,477,.083333336,6,72
48,1,8,478,.16129032,15,93
48,1,9,479,.13095239,11,84
48,1,10,480,.2238806,15,67
49,1,1,481,0,0,24
49,1,2,482,0,0,16
49,1,3,483,0,0,30
49,1,4,484,0,0,35
49,1,5,485,.023255814,1,43
49,1,6,486,0,0,43
49,1,7,487,.083333336,4,48
49,1,8,488,.11627907,5,43
49,1,9,489,.2,7,35
49,1,10,490,.17647059,6,34
50,1,1,491,0,0,129
50,1,2,492,.027777778,3,108
50,1,3,493,.014705882,2,136
50,1,4,494,.0081967209,1,122
50,1,5,495,.020408163,2,98
50,1,6,496,.05050505,5,99
50,1,7,497,.061946902,7,113
50,1,8,498,.1388889,10,72
50,1,9,499,.12244898,12,98
50,1,10,500,.30434781,21,69
51,1,1,501,0,0,21
51,1,2,502,0,0,25
51,1,3,503,0,0,35
51,1,4,504,.039999999,1,25
51,1,5,505,.055555556,2,36
51,1,6,506,.11627907,5,43
51,1,7,507,.037037037,1,27
51,1,8,508,.14285715,3,21
51,1,9,509,.17777778,8,45
51,1,10,510,.19047619,8,42
52,1,1,511,0,0,3
52,1,2,512,0,0,9
52,1,3,513,0,0,9
52,1,4,514,0,0,17
52,1,5,515,0,0,15
52,1,6,516,0,0,12

For peer review only

52,1,7,517,.22222222,2,9
52,1,8,518,.083333336,1,12
52,1,9,519,0,0,10
52,1,10,520,.18181819,2,11
53,1,1,521,0,0,7
53,1,2,522,0,0,12
53,1,3,523,0,0,18
53,1,4,524,.07692308,1,13
53,1,5,525,0,0,13
53,1,6,526,0,0,23
53,1,7,527,.083333336,1,12
53,1,8,528,.071428575,1,14
53,1,9,529,.14285715,3,21
53,1,10,530,.071428575,1,14
54,1,1,531,.030927835,3,97
54,1,2,532,.016393442,2,122
54,1,3,533,.034188036,4,117
54,1,4,534,.016806724,2,119
54,1,5,535,.028037382,3,107
54,1,6,536,.032786883,4,122
54,1,7,537,.10084034,12,119
54,1,8,538,.10891089,11,101
54,1,9,539,.18518518,15,81
54,1,10,540,.32075471,34,106
55,1,1,541,0,0,27
55,1,2,542,0,0,53
55,1,3,543,0,0,30
55,1,4,544,0,0,38
55,1,5,545,0,0,37
55,1,6,546,0,0,38
55,1,7,547,.07692308,3,39
55,1,8,548,.10344828,3,29
55,1,9,549,.12903225,4,31
55,1,10,550,.1891892,7,37
56,1,1,551,.021276595,1,47
56,1,2,552,0,0,38
56,1,3,553,.039215688,2,51
56,1,4,554,.019607844,1,51
56,1,5,555,0,0,37
56,1,6,556,.063829787,3,47
56,1,7,557,.063829787,3,47
56,1,8,558,.14285715,6,42
56,1,9,559,.18181819,8,44
56,1,10,560,.21875,7,32
57,1,1,561,.014492754,1,69
57,1,2,562,0,0,73
57,1,3,563,.011494253,1,87

For peer review only

57,1,4,564,.033333335,3,90
57,1,5,565,.045454547,4,88
57,1,6,566,.054054055,4,74
57,1,7,567,.084337346,7,83
57,1,8,568,.14473684,11,76
57,1,9,569,.17647059,12,68
57,1,10,570,.30864197,25,81
58,1,1,571,0,0,22
58,1,2,572,.043478262,1,23
58,1,3,573,0,0,42
58,1,4,574,.057142857,2,35
58,1,5,575,0,0,34
58,1,6,576,.060606062,2,33
58,1,7,577,0,0,34
58,1,8,578,.16666667,7,42
58,1,9,579,.10714286,3,28
58,1,10,580,.2,6,30
59,1,1,581,0,0,12
59,1,2,582,.037037037,1,27
59,1,3,583,.035714287,1,28
59,1,4,584,.043478262,1,23
59,1,5,585,.03846154,1,26
59,1,6,586,.13333334,2,15
59,1,7,587,.052631579,1,19
59,1,8,588,0,0,30
59,1,9,589,.22222222,4,18
59,1,10,590,.30434781,7,23
60,1,1,591,.015151516,1,66
60,1,2,592,.015873017,1,63
60,1,3,593,.04225352,3,71
60,1,4,594,0,0,80
60,1,5,595,.016129032,1,62
60,1,6,596,.050847456,3,59
60,1,7,597,.11320755,6,53
60,1,8,598,.065217391,3,46
60,1,9,599,.18181819,8,44
60,1,10,600,.1627907,7,43
61,1,1,601,.0066666668,1,150
61,1,2,602,.016666668,2,120
61,1,3,603,.033613447,4,119
61,1,4,604,.036036037,4,111
61,1,5,605,.021978023,2,91
61,1,6,606,.036144577,3,83
61,1,7,607,.11111111,10,90
61,1,8,608,.17105263,13,76
61,1,9,609,.12371134,12,97
61,1,10,610,.29487181,23,78

For peer review only

62,1,1,611,0,0,31
62,1,2,612,.039999999,1,25
62,1,3,613,0,0,31
62,1,4,614,0,0,35
62,1,5,615,.055555556,2,36
62,1,6,616,.05882353,2,34
62,1,7,617,.10714286,3,28
62,1,8,618,.081081077,3,37
62,1,9,619,.125,6,48
62,1,10,620,.2,7,35
63,1,1,621,0,0,17
63,1,2,622,0,0,18
63,1,3,623,.095238097,2,21
63,1,4,624,.043478262,1,23
63,1,5,625,.03846154,1,26
63,1,6,626,0,0,20
63,1,7,627,.047619049,1,21
63,1,8,628,.17647059,3,17
63,1,9,629,.11111111,2,18
63,1,10,630,.17647059,3,17
64,1,1,631,0,0,31
64,1,2,632,0,0,25
64,1,3,633,.125,4,32
64,1,4,634,.15000001,3,20
64,1,5,635,.19354838,6,31
64,1,6,636,.074074075,2,27
64,1,7,637,.16,4,25
64,1,8,638,.26086956,6,23
64,1,9,639,.34615386,9,26
64,1,10,640,.46666667,7,15
65,1,1,641,0,0,32
65,1,2,642,0,0,22
65,1,3,643,0,0,24
65,1,4,644,0,0,28
65,1,5,645,0,0,32
65,1,6,646,.045454547,2,44
65,1,7,647,0,0,29
65,1,8,648,.12,3,25
65,1,9,649,.24137931,7,29
65,1,10,650,.13636364,3,22
66,1,1,651,0,0,18
66,1,2,652,0,0,30
66,1,3,653,0,0,16
66,1,4,654,.095238097,2,21
66,1,5,655,0,0,28
66,1,6,656,.050000001,1,20
66,1,7,657,.039999999,1,25

For peer review only

66,1,8,658,.16129032,5,31
66,1,9,659,.24137931,7,29
66,1,10,660,.32258064,10,31
67,1,1,661,0,0,17
67,1,2,662,0,0,12
67,1,3,663,.047619049,1,21
67,1,4,664,0,0,16
67,1,5,665,0,0,11
67,1,6,666,.16666667,2,12
67,1,7,667,0,0,15
67,1,8,668,.10526316,2,19
67,1,9,669,.21052632,4,19
67,1,10,670,.38095239,8,21
68,1,1,671,0,0,70
68,1,2,672,0,0,48
68,1,3,673,0,0,57
68,1,4,674,.032786883,2,61
68,1,5,675,.039215688,2,51
68,1,6,676,.06666667,3,45
68,1,7,677,.12727273,7,55
68,1,8,678,.13636364,9,66
68,1,9,679,.19642857,11,56
68,1,10,680,.31481481,17,54

For peer review only

STROBE 2007 (v4) Statement—Checklist of items that should be included in reports of *cross-sectional studies*

Section/Topic	Item #	Recommendation	Reported on page #
**Title and abstract**	1	(*a*) Indicate the study's design with a commonly used term in the title or the abstract	2
		(*b*) Provide in the abstract an informative and balanced summary of what was done and what was found	2
**Introduction**			
Background/rationale	2	Explain the scientific background and rationale for the investigation being reported	3-6
Objectives	3	State specific objectives, including any prespecified hypotheses	6
**Methods**			
Study design	4	Present key elements of study design early in the paper	7
Setting	5	Describe the setting, locations, and relevant dates, including periods of recruitment, exposure, follow-up, and data collection	7
Participants	6	(*a*) Give the eligibility criteria, and the sources and methods of selection of participants	7
Variables	7	Clearly define all outcomes, exposures, predictors, potential confounders, and effect modifiers. Give diagnostic criteria, if applicable	8-9
Data sources/ measurement	8*	For each variable of interest, give sources of data and details of methods of assessment (measurement). Describe comparability of assessment methods if there is more than one group	8-9
Bias	9	Describe any efforts to address potential sources of bias	NA
Study size	10	Explain how the study size was arrived at	7
Quantitative variables	11	Explain how quantitative variables were handled in the analyses. If applicable, describe which groupings were chosen and why	9
Statistical methods	12	(*a*) Describe all statistical methods, including those used to control for confounding	8-17
		(*b*) Describe any methods used to examine subgroups and interactions	8
		(*c*) Explain how missing data were addressed	NA
		(*d*) If applicable, describe analytical methods taking account of sampling strategy	NA
		(*e*) Describe any sensitivity analyses	12-13
**Results**			

For peer review only

Participants	13*	(a) Report numbers of individuals at each stage of study—eg numbers potentially eligible, examined for eligibility, confirmed eligible, included in the study, completing follow-up, and analysed	Figure 1
		(b) Give reasons for non-participation at each stage	NA
		(c) Consider use of a flow diagram	Figure 1
Descriptive data	14*	(a) Give characteristics of study participants (eg demographic, clinical, social) and information on exposures and potential confounders	17, Table 2
		(b) Indicate number of participants with missing data for each variable of interest	NA
Outcome data	15*	Report numbers of outcome events or summary measures	17
Main results	16	(*a*) Give unadjusted estimates and, if applicable, confounder-adjusted estimates and their precision (eg, 95% confidence interval). Make clear which confounders were adjusted for and why they were included	18, Table 3
		(*b*) Report category boundaries when continuous variables were categorized	Table 2
		(*c*) If relevant, consider translating estimates of relative risk into absolute risk for a meaningful time period	NA
Other analyses	17	Report other analyses done—eg analyses of subgroups and interactions, and sensitivity analyses	18
**Discussion**			
Key results	18	Summarise key results with reference to study objectives	19
Limitations	19	Discuss limitations of the study, taking into account sources of potential bias or imprecision. Discuss both direction and magnitude of any potential bias	14, 21
Interpretation	20	Give a cautious overall interpretation of results considering objectives, limitations, multiplicity of analyses, results from similar studies, and other relevant evidence	20-21
Generalisability	21	Discuss the generalisability (external validity) of the study results	21
**Other information**			
Funding	22	Give the source of funding and the role of the funders for the present study and, if applicable, for the original study on which the present article is based	22

*Give information separately for cases and controls in case-control studies and, if applicable, for exposed and unexposed groups in cohort and cross-sectional studies.

Note: An Explanation and Elaboration article discusses each checklist item and gives methodological background and published examples of transparent reporting. The STROBE checklist is best used in conjunction with this article (freely available on the Web sites of PLoS Medicine at http://www.plosmedicine.org/, Annals of Internal Medicine at http://www.annals.org/, and Epidemiology at http://www.epidem.com/). Information on the STROBE Initiative is available at www.strobe-statement.org.

For peer review only

BMJ Open

A Cross-Classified Multilevel Analysis of Individual Heterogeneity and Discriminatory Accuracy (MAIHDA) to evaluate hospital performance: The case of Hospital Differences in Patient Survival after Acute Myocardial Infarction

Journal:	*BMJ Open*
Manuscript ID	bmjopen-2019-036130.R2
Article Type:	Original research
Date Submitted by the Author:	12-Sep-2020

Complete List of Authors:	Rodriguez-Lopez, Merida; Pontificia Universidad Javeriana - Cali; Lunds University Faculty of Medicine, Unit for Social Epidemiology<br>Merlo, Juan; Lunds University Faculty of Medicine, Unit for Social Epidemiology; Center for Primary Health Care Research<br>Perez-Vicente, Raquel; Lunds University Faculty of Medicine, Unit for Social Epidemiology<br>Austin, Peter; University of Toronto, Institute of Health Management, Policy and Evaluation; Institute for Clinical Evaluative Sciences<br>Leckie, George; University of Bristol, Centre for Multilevel Modelling

**Primary Subject Heading**:	Epidemiology
Secondary Subject Heading:	Health services research, Public health, Cardiovascular medicine, Research methods
Keywords:	EPIDEMIOLOGY, Quality in health care < HEALTH SERVICES ADMINISTRATION & MANAGEMENT, Adult cardiology < CARDIOLOGY

SCHOLARONE™ Manuscripts

I, the Submitting Author has the right to grant and does grant on behalf of all authors of the Work (as defined in the below author licence), an exclusive licence and/or a non-exclusive licence for contributions from authors who are: i) UK Crown employees; ii) where BMJ has agreed a CC-BY licence shall apply, and/or iii) in accordance with the terms applicable for US Federal Government officers or employees acting as part of their official duties; on a worldwide, perpetual, irrevocable, royalty-free basis to BMJ Publishing Group Ltd ("BMJ") its licensees and where the relevant Journal is co-owned by BMJ to the co-owners of the Journal, to publish the Work in this journal and any other BMJ products and to exploit all rights, as set out in our licence.

The Submitting Author accepts and understands that any supply made under these terms is made by BMJ to the Submitting Author unless you are acting as an employee on behalf of your employer or a postgraduate student of an affiliated institution which is paying any applicable article publishing charge ("APC") for Open Access articles. Where the Submitting Author wishes to make the Work available on an Open Access basis (and intends to pay the relevant APC), the terms of reuse of such Open Access shall be governed by a Creative Commons licence – details of these licences and which Creative Commons licence will apply to this Work are set out in our licence referred to above.

Other than as permitted in any relevant BMJ Author's Self Archiving Policies, I confirm this Work has not been accepted for publication elsewhere, is not being considered for publication elsewhere and does not duplicate material already published. I confirm all authors consent to publication of this Work and authorise the granting of this licence.

For peer review only

A Cross-Classified Multilevel Analysis of Individual Heterogeneity and Discriminatory Accuracy (MAIHDA) to evaluate hospital performance:

The case of Hospital Differences in Patient Survival after Acute Myocardial Infarction

Merida Rodriguez-Lopez[1,2]

Juan Merlo[1,3]

Raquel Perez-Vicente [1]

Peter C. Austin[4,5,6]

George Leckie [1,7]

[1] Unit for Social Epidemiology, Faculty of Medicine, Lund University, Malmö, Sweden

[2] Pontificia Universidad Javeriana seccional Cali, Colombia

[3] Center for Primary Health Care Research, Region Skåne, Malmö, Sweden

[4] Institute for Clinical Evaluative Sciences, Toronto, Ontario, Canada

[5] Institute of Health Management, Policy and Evaluation, University of Toronto, Toronto, Ontario, Canada

[6] Schulich Heart Research Program, Sunnybrook Research Institute, Toronto, Ontario, Canada

[7] Centre for Multilevel Modelling, University of Bristol, Bristol, UK

Short title: Hospital Performance by MAIHDA

Corresponding author:
Dr. Merida Rodriguez-Lopez
merida.rdguez@gmail.com
Pontificia Universidad Javeriana-Cali, Colombia
Calle 18 No.118 - 250
Cali, Valle del Cauca, CO

Patient consent: Not required.

For peer review only - http://bmjopen.bmj.com/site/about/guidelines.xhtml

For peer review only

Abstract

Objective: To describe a novel strategy, Multilevel Analysis of Individual Heterogeneity and Discriminatory Accuracy (MAIHDA) to evaluate hospital performance, by analyzing differences in 30-day mortality after a first-ever acute myocardial infarction (AMI) in Sweden.

Design: Cross-classified study

Setting: 68 Swedish hospitals

Participants: 43,247 patients admitted between 2007-2009, with a first-ever AMI.

Primary and secondary outcome measures: We evaluate hospital performance by analyzing differences in 30-day mortality after a first-ever AMI using a cross-classified multilevel analysis We classified the patients into 10 categories according to a risk score (RS) for 30-day mortality and created 680 strata defined by combining hospital and RS categories.

Results: In the cross-classified multilevel analysis the overall RS adjusted hospital 30-day mortality in Sweden was 4.78% and the between-hospital variation was very small (Variance Partition coefficient (VPC)= 0.70%, Area under the curve (AUC)= 0.54). The benchmark value was therefore achieved by all hospitals. However, as expected, there were large differences between the RS categories (VPC= 34.13%, AUC= 0.77)

Conclusions: MAIHDA is a useful tool to evaluate hospital performance. The benefit of this novel approach to adjusting for patient RS is that it allowed one to estimate separate VPCs and AUC statistics to simultaneously evaluate the influence of RS categories and hospital differences on mortality. At the time of our analysis, all hospitals in Sweden were performing homogenously well. That is, the benchmark target for 30-day mortality was fully achieved and there were not relevant hospital differences. Therefore, possible quality interventions should be universal and oriented to maintain the high hospital quality of care.

Keywords: Multilevel analysis, Health Evaluation, Decision Making, ROC curve, Variance analysis

Strengths and limitations of this study

We provide a new analytical tool for analysing hospital performance based on multilevel analysis of individual heterogeneity and discriminatory accuracy (MAIHDA).

Cross-classified MAIHDA disentangles the specific role of the patient-mix vs. the hospital when analyzing quality outcomes.

We used a risk score to adjust for differences in patient-mix across hospitals. However, it is not a perfect instrument to quantify the true severity and mortality risk of a patient.

MAIHDA allows analysts to identify whether target or universal interventions are most appropriate to improve the quality of care.

We provide a three-step strategy to achieve a complete analysis of hospital performance. However, more elaborated strategies are also possible.

For peer review only

Introduction

Hospital effects

When evaluating institutional (e.g., hospital) performance in health care, traditional studies make two implicit assumptions. First, it is assumed that over and above patient characteristics, the hospital context exerts a general, shared effect on all patients at the hospital. This general hospital-context effect is argued to reflect the influence of many factors, for instance, hospital administration, access to resources, specialized knowledge, implementation of methods for disease management, and adoption of guidelines and pathways for patient treatments. Second, it is often assumed that the general hospital-context effect can be measured by quantifying differences between hospital averages in certain quality indicators. Therefore, the focus of the analysis is based on the interpretation of tables, funnel plots, control charts, 'league tables' or similar, where hospitals are ranked according to different quality indicators such as their average 30-day mortality after acute myocardial infarction (AMI) (1). Occasionally such analyses are accompanied by an estimation of the reliability of the ranking ("*rankability*") (2), but more often than not the focus of analysis remains on hospital averages.

Multilevel Analysis of Individual Heterogeneity and Discriminatory Accuracy (MAIHDA)

Recently, MAIHDA has been proposed as a novel strategy for evaluating hospital performance (3). In contrast with most traditional studies, hospital MAIHDA simultaneously focuses on both hospital averages *and* patient heterogeneity around such averages. In MAIHDA, the fundamental statement is that patient and hospital variation should not be analysed separately. Rather, we need to consider that the total individual outcome variance can be partitioned into variance components operating at different levels of analysis (4). From this perspective, hospital differences are not measured as the difference between hospital averages, but as the hospital general contextual effect (GCE). That is, the share of the total individual variance in patient

For peer review only - http://bmjopen.bmj.com/site/about/guidelines.xhtml

outcomes that is at the hospital level. This definition aligns with that for the variance partition coefficient (VPC) in multilevel modelling. The greater the GCE, the more important the hospital context is for explaining variation in individual outcomes (5-7). This idea is also closely related to the notion of discriminatory accuracy developed for the evaluation of the performance of prognostic and screening markers in medicine (8, 9). It is therefore possible to also use measures of discriminatory accuracy such as the area under the Receiving Operator Characteristics curve (AUC), to quantify the hospital GCE (10). See elsewhere for an extended explanation of the GCE concept (3, 7, 11). In this article, we argue that the systematic application of measures of variance and discriminatory accuracy is of fundamental relevance for meaningful performance evaluations (3, 5, 11-13).

Cross-classified MAIHDA

Hospital comparisons are usually adjusted for "patient-mix" using a risk score (RS). In traditional multilevel analysis of hospital performance, patient RS effects are modelled as fixed effects (e.g., by entering the set of RS categories as a series of dummy variables) while the hospital effects are modelled as random effects. In contrast, in the cross-classified MAIHDA approach both the RS and the hospitals are modelled as random effects. Readers familiar with the traditional application of multilevel modelling may query the treatment of RS categories as random effects. For example, while we can think of the hospitals as a sample drawn from the set of all possible hospitals, it proves harder to conceptualize the RS categories in this way as there is not a large population of RS categories from which they are drawn. This is, however, a philosophical, rather than a practical question. In fact, when studying hospitals in a country the hospitals are never a sample of an infinite super population of hospitals but a concrete set of facilities in a specific setting. Furthermore, many multilevel studies observe and analyze all the hospitals in a country in their data, and the total number of hospitals may not prove that large, yet here too the hospital effects will be treated as random effects. As discussed by Snijders and

For peer review only - http://bmjopen.bmj.com/site/about/guidelines.xhtml

Bosker, when defining the random intercept model (14), p. 45, the random effects model can be applied even when the idea of an infinite superpopulation is less evident. This approach is currently being applied when performing intersectional MAIHDA in social epidemiology (15).

The cross-classified approach provides several advantages over the traditional hierarchical multilevel approach. First, the cross-classified MAIHDA is parsimonious as it includes only one random parameter for the n RS categories rather than the $n-1$ dummy variables as in the fixed effects approach. Second, the cross-classified approach provides separate VPCs and AUCs for RS categories and for the hospital, allowing their magnitude to be contrasted. Thus, in contrast to the fixed-effects approach, it allows the importance of patient-mix vs. the hospital effects to be communicated on a common metric. In addition, hospital MAIHDA provides all the usual advantages of multilevel models. For instance, by providing reliability weighted hospital averages (shrunken residuals), it reduces the concern of monitoring outcome measures based on small hospital caseloads which otherwise may lead to extreme and unstable hospital rankings and, therefore, unreliable performance evaluation (16, 17). Both hierarchical and cross-classified MAIHDA are nowadays easy to implement in available software such as MLwiN that can be run from both within Stata (runmlwin) (18) and within R (R2MLwiN) (19).

Finally, for binary patient outcomes, such as 30-day mortality after AMI, multilevel analyses can be performed using a simple contingency table or matrix with strata defined by combinations of the hospitals and the RS categories capturing patient-mix. The only information required for the analysis is the overall number of patients and the number of AMI cases in each hospital-RS stratum. This aggregated approach maintains the joint distribution of the hospitals-RS information and provides the same model results (parameter estimates, standard errors, fit statistics and predictions) as when analysing the underlying individual level data. The aggregated approach also allows computationally efficient (fast) estimation as it allows analysing thousands of patients' outcomes using a dataset consisting of just a few

For peer review only - http://bmjopen.bmj.com/site/about/guidelines.xhtml

hundred strata. A further benefit of the aggregated approach is that the data can be shared since its aggregated presentation reduces ethical problems of confidentiality (statistical disclosure is not at risk). This in turn, improves the transparency of the research and facilitates the replication of the analysis and encourages the sharing of data to compare hospital performance between different settings.

The aim of this study

The aim of this study was to demonstrate a novel statistical approach (MAIHDA) to evaluate hospital performance and to orientate stakeholders to make assertive data informed decisions using a three-step framework. We do so by analyzing differences in 30-day mortality among patients admitted to the Swedish hospital with a first-ever AMI between 2007-2009.

Methods

Study Population

This is a cross-sectional study. We used information from the Swedish Patient Register (20) and from the Cause of Death Register (21) (National Board of Health and Welfare) as well as from Population Register (22) (Statistics Sweden). To ensure the anonymity of the subjects, the Swedish authorities transformed the personal identification numbers of the individuals (23) into arbitrary personal numbers before delivering the research databases to us, and we linked the databases using the anonymized identification number.

From the research database we identified all 47,462 patients with a discharge diagnosis of AMI coded as I21 according the 10th edition of the International Classification of Diseases (ICD), admitted to Swedish hospitals between 2007 and 2009 and being 45 to 80 years old. The flow diagram of the study population is shown in Figure 1.

Ethical statement

For peer review only - http://bmjopen.bmj.com/site/about/guidelines.xhtml

This research was done without patient involvement. The Regional Ethics Review Board in southern Sweden (# 2012/637) as well as the data safety committees from the National Board of Health and Welfare and from Statistics Sweden approved the construction of the database used in this study.

Data accessibility

The original databases used in our study are available from the Swedish National Board of Health and Welfare and Statistics. In Sweden, register data are protected by strict rules of confidentiality (24) but can be made available for research after a special review that includes approval of the research project by both an Ethics Committee and the authorities' own data safety committees. The Swedish authorities under the Ministry of Health and Social Affairs do not provide individual level data to researchers abroad. Instead, they normally advise researchers in other countries to cooperate with Swedish colleagues and analyze data in collaboration according to standard legal provisions and procedures.

However, in the approach we propose, it is technically possible to perform the analysis using a simple table defined by hospital and categories of risk score. The aggregated information as well as the additional encryption of the hospital name fully anonymized the table, which prevents the backwards identification of individuals even when very few patients are in a single cell of the table. Therefore, to increase transparency and facilitate the replication of our analysis we provide the table as a Stata dataset (Supplementary material S1) and a fully annotated Stata do-file to allow the replication of the analyses (Supplementary material S2). We also provide the table as a CSV file along with an R Script (Supplemental material S3, S4).

Assessment of variables

Patient outcome

For peer review only - http://bmjopen.bmj.com/site/about/guidelines.xhtml

The study outcome was all-cause mortality within 30-days after admission to the hospital (coded yes vs. no) due to AMI.

Risk score for mortality

An inherent difficulty when investigating quality outcome indicators such as mortality is the threat of confounding due to differences in patient-mix across hospitals. The geographical areas covered by the hospital may vary in the demographical and disease characteristics of the patients. Furthermore, patients with a worse prognosis may be channeled to certain hospitals providing specialized care and this selection of patients will further confound the evaluations of hospitals differences. To reduce this form of confounding we computed a RS for 30-day mortality in the sample of AMI patients. Initially we selected a priori 40 variables including sex (man vs. woman), age in years (as a quadratic function) and several previous diseases (ICD-10 codes) registered in the Swedish Patient Register. We then modeled 30-day mortality using a single-level logistic regression in STATA 14 (Statacorp, College Station, Texas, US), and stepwise variable selection with a significance level of 0.10 and 0.05 for removal and inclusion of variables, respectively. The variables retained in the final model were, age, arrhythmia (I48-I49), cancer (C1-D4), diseases of the cerebral arteries (I6), chronic diseases of the lower respiratory tract (J4), diabetes (E10-E14), digestive diseases (K0-K9), other types of heart disease (I3-I5), hypertension (I10-I13 I15), hearth failure (I50), injury (S00-T14), ischemic coronary artery disease (I20-I25), lung cancer (C34), mental diseases (F0-F9), and peripheral vascular disease (I74 I80) as well as respiratory diseases (J0-J9). Finally, we obtained the predicted probability of 30-day mortality and defined it as our patient risk score (RS). We discretized the RS into 10 categories using the decile values of the RS distribution. We chose deciles to provide enough granulation of the continuous RS variable and enough categories to be included as a random effect in the multilevel model.

For peer review only

For peer review only - http://bmjopen.bmj.com/site/about/guidelines.xhtml

Statistical analyses

We analysed 30-day mortality among 43,247 patients admitted to 68 Swedish hospitals between 2007-2009, with a first-ever AMI. We classified the patients into 10 RS categories for 30-day mortality and created 680 strata defined by combining hospital and RS categories. In the first step (model 1), we applied a traditional hierarchical multilevel logistic regression model with patients clustered within hospitals. In a second step (model 2), and in order to adjust for patient-mix, we performed a cross-classified multilevel model of patient outcomes with both RS categories and hospitals random effects (25). We estimated the VPC and the AUC to evaluate differences between RS categories and between hospitals in a common way.

Estimation methods

We performed the estimations using Markov Chain Monte Carlo (MCMC) methods, with diffuse (vague, flat, or minimally informative) prior distributions for all parameters. We used quasilikelihood methods to provide starting values for all parameters. For each model, the burn-in length was 5,000 iterations. We ran the model for a further 10,000 monitoring iterations and used the resulting parameter chains from the MCMC to construct 95% credible intervals (CI) for all model predictions to communicate statistical uncertainty (Supplementary material S2). Visual assessments of the parameter chains and standard MCMC convergence diagnostics suggested that the monitored chains had converged.

Model 1: Unadjusted multilevel analysis

Model 1 was a multilevel logistic regression for patient mortality where we only include a hospital random effect to account for the variation in mortality rates across hospitals. Let, y_j denote the number of deaths in hospital j ($j = 1,...,68$). The model can then be written as

$$y_j \sim \text{Binomial}(n_j, \pi_j)$$

For peer review only - http://bmjopen.bmj.com/site/about/guidelines.xhtml

$$\text{logit}\,(\pi_j) \equiv \log\left(\frac{\pi_j}{1-\pi_j}\right) = \beta_0 + H_j$$
$$H_j \sim N(0, \sigma_H^2)$$

Formula 1

where n_j denotes the total number of patients in hospital j, π_j denotes the probability of death for patients in that hospital, β_0 denotes the intercept or precision weighted grand mean, that in logistic regression represents the average of the hospital-specific shrunken (i.e., reliability-weighted) residuals values, and H_j denotes the hospital-specific random effects for the 68 hospitals (26). The random effects are assumed to be normally distributed with zero mean and between-hospital variance σ_H^2.

The purpose of this model was to evaluate unadjusted hospital differences in average mortality risk. For this aim, we (A) ranked the hospitals according to their mortality risk; and (B) complemented this information by quantifying the size of the GCE.

A) Ranking of the hospitals

To rank hospitals according to their *unadjusted* mortality rates, we predict the absolute risk (AR_j) of 30-day mortality and its 95% CI in each hospital. To do so, we first transformed the predicted logit of 30-day mortality into proportions (π_j) as follows

$$\text{AR}_H \equiv \pi_j \equiv \text{logit}^{-1}\,(\beta_0 + H_j) = \frac{\exp\,(\beta_0 + H_j)}{1 + \exp\,(\beta_0 + H_j)}$$

Formula 2

B) Measuring the hospital GCE.

We estimate the hospital GCE by means of two measures:

(i) The Variance Partition Coefficient for the hospital level (VPC_H)

The VPC_H can be calculated based on the latent response formulation of the model which is an approach widely adopted today in multilevel applied work (27-31) .

For peer review only - http://bmjopen.bmj.com/site/about/guidelines.xhtml

$$\mathrm{VPC}_H = \frac{\sigma_H^2}{\sigma_H^2 + \frac{\pi^2}{3}}$$

Formula 3

Where $\frac{\pi^2}{3} \cong 3.29$ denotes the variance of a standard logistic distribution. We then multiply the VPC_H by 100 and interpret it as a percentage.

The VPC_H, quantifies the share of the total individual differences in the latent propensity of 30-day mortality that is at the hospital level. The VPC_H embraces the influence of the hospital context on the patient outcome without identifying any specific hospital information. However, the VPC_H, may also reflect differences in patient-mix between hospitals. In any case, theVPC_H, represents the hospital ceiling effect or potential maximum influence of the hospital attended. In the absence of confounding by patient-mix, the higher the VPC_H, the higher the hospital GCE is. In other words, the more relevant the hospital context is for understanding individual variation in the latent risk for 30-day mortality.

(ii) The area under the receiver operating characteristics curve for the hospital (AUC_H)

A well-known measure of discriminatory accuracy is the AUC (10, 11, 32). In our case the hospital $\mathrm{AUC_H}$ measures how well the model predicted probabilities based on the attended hospitals distinguish between two outcome categories (death within 30 days or survival). The $\mathrm{AUC_H}$ is constructed by plotting the true positive fraction (TPF) against the false positive fraction (FPF) for different thresholds of the predicted probabilities. The AUC takes a value between 1 and 0.5 where 1 is perfect discrimination and 0.5 would be equally as informative as flipping a coin (i.e., the hospital information has no discriminatory accuracy).

We calculated the AUC_H and to account for the different number of patients in each hospital we also calculated the weighted AUC (AUC_{wH}) where every patient was weighted by the inverse

For peer review only - http://bmjopen.bmj.com/site/about/guidelines.xhtml

of the number of patients at his/her hospital. In our study both the unweighted and the weighted AUCs were almost identical so we only present the unweighted AUC_H.

Model 2: Patient-mix adjusted multilevel analysis

Model 2 was a cross-classified multilevel logistic regression (25) for patient mortality in which we included both hospital and RS category random effects to simultaneously account for the variation in mortality across both hospitals and RS categories and to therefore adjust the hospital effects for patient-mix. The model can be written as:

$$y_j \sim \mathrm{Binomial}(n_j, \pi_j)$$
$$\mathrm{logit}\,(\pi_{jk}) \equiv \log\left(\frac{\pi_{jk}}{1-\pi_{jk}}\right) = \beta_0 + H_j + RS_k$$
$$H_j \sim N(0, \sigma_H^2)$$
$$RS_k \sim N(0, \sigma_{RS}^2)$$

Formula 4

where RS_k denotes the random effect associated with RS categories k ($k = 1, \ldots, 10$), which are assumed to be normally distributed with zero mean and between-RS variance σ_{RS}^2.

The predicted logit of 30-day mortality was transformed into proportions. The mortality rates from this model are standardized and represents the rate that each hospital would have experienced if all hospitals had treated the same patients, in our case a patient with an average RS value in the population or $RS_k = 0$.

The purpose of model 2 was to evaluate patient-mix adjusted hospital differences in average mortality risk. Therefore, and analogously to model 1, we ranked the hospitals according to their RS adjusted mortality risk and complemented this information by quantifying the size of the hospital GCE net of the observed patient-mix influence. Visual inspection of the hospital and RS category predicted random effects showed the random effect normality assumptions were satisfied. (Supplementary material S2). As a measure of the patient-mix adjusted hospital

For peer review only

GCE, we obtained the hospital VPC_H as

$$\mathrm{VPC}_H = \frac{\sigma_H^2}{\sigma_H^2 + \sigma_{RS}^2 + \frac{\pi^2}{3}}$$

Formula 5

We also calculated the VPC for the RS category (VPC_{RS}) as

$$VPC_{RS} = \frac{\sigma_{RS}^2}{\sigma_H^2 + \sigma_{RS}^2 + \frac{\pi^2}{3}}$$

Formula 6

The adjusted VPC_H and VPC_{RS} inform on the share of the total individual variance in the latent propensity of 30-day mortality that is at the hospital and at the RS category level respectively, net of the influence of the other factor. Both measures are estimated on the same scale and can therefore be directly compared to evaluate the relative relevance of hospital versus patient-mix information when it comes to understanding patient differences in the latent propensity of death. We also calculated the adjusted AUC for the hospital level (AUC_H) and for RS category levels (AUC_{RS}) including their specific random effects when calculating the predicted probabilities. In order to compare the results from the cross-classified approach (model 2) with the traditional Hierarchical Random Intercept approach, we performed a third model including the RS categories as fixed effects (Supplementary material S2). The results from both models (2 and 3) were similar in terms of Bayesian Deviance Information Criterion (DIC), VPC and AUC.

While patients with relatively mild conditions may have similarly good outcomes regardless of where they are treated, outcomes of the most complex patients may be affected by hospital performance. We therefore fitted a cross-classified model including a random interaction effect between the Hospital and the RS (Supplementary material S2). That is, we allowed the effect that a Hospital has on their patients to vary according to the RS classification of their patients and vice versa. However, the resulting interaction classification variance was very low,

For peer review only - http://bmjopen.bmj.com/site/about/guidelines.xhtml

suggesting that hospital attended and patient RS have additive effects on the log-odds of AMI. Consequently, we based our analysis on model 1 and 2.

Software

All models were run in MLwiN 3.05 (33) called from Stata using the *runmlwin* command (18). We note that MLwiN can equally be called from within R using the sister R2MLwiN package (19) and so our analysis can also be replicated by readers in that statistical package.

Evaluating hospital performance with MAIHDA

The present cross-classified MAIHDA framework extends that which was described in a previous publication aimed to the evaluation of geographical differences in health outcomes (34). The framework proposes three steps that need be considered to achieve a complete analysis of hospital performance. However, more elaborated strategies are of course also possible, and the presented framework is open for modifications and extensions. The application of the framework in our study was as follows

Step 1. Identifying a benchmark value and evaluating the adjusted hospital mortality rates against it.

When evaluating hospital performance, we need to identify a benchmark value expressing a tolerable average level of 30-day mortality in the population of AMI patients. However, the selection of a specific benchmark is often difficult and arbitrary. We can use an *internal benchmark* defined as the β_0 obtained in model 2 (*Formula 4*). That is, the mortality rate in a hospital with an average mortality ($H_j = 0$) treating patients with an average RS ($RS_k = 0$). This choice is meaningful since comparing with a national average seems "fair" and being RS adjusted, tertiary care hospitals with more severe cases do not unfairly push the hospital effect towards a higher value as in the crude average rate. However, being an adjusted rate the value does not necessarily resemble the crude rates and can only be used for relative comparisons.

For peer review only - http://bmjopen.bmj.com/site/about/guidelines.xhtml

Other adjustments are possible such as computing adjusted hospital mortality rates by holding RS_k value equal to the 90th percentile of the RS random effect distribution. Another possibility would be to calculate the adjusted hospital rates that would arise if each hospital treated a nationally representative sample of patients. However, statistically, this would be more complex to do as this would require integrating out the RS random effect (via simulation) and so we do not pursue this here.

We could also use and *external benchmark* by applying a RS equation for 30-day mortality obtained in another country or in an international collaboration. This approach seems worthy for international comparisons, but it may also be inappropriate if the RS equation does not properly account for population demographical and co-morbidities differences in risk, and the diagnostic criteria are not fully standardized. Finally, we could use an arbitrary rate based on previous evidence and proven expertise. Considering the data published by the Organisation for Economic Co-operation and Development (OECD) (35) and a recent review article (36), we decide a RS adjusted 30-day mortality of less than 6% as a desirable target value for the purposes of this illustrative application. We defined three categories of achievement in relation to the benchmark, fully reached target (≤6%), closely reached target value (6%-8%), and insufficiently reached target value (≥8%). We used caterpillar graphs to compare the RS adjusted hospital rates in relation to the benchmark value of 6%.

Step 2. Quantifying the size of the hospital differences using the VPC and the AUC

Currently there is no official guidance for assessing the magnitude of the VPC or the AUC in the context of studying hospital differences in RS adjusted hospital 30-day mortality but a practical proposal is described in Table 1. This table also shows the corresponding AUC values according to the simulated relationship between the AUC and VPC published elsewhere (34). The proposed values are based on the authors' own experience, but further discussion is

For peer review only - http://bmjopen.bmj.com/site/about/guidelines.xhtml

encouraged to arrive at a standard classification. Furthermore, different standards may ultimately be required and developed for different outcomes in different contexts and at different points in time. For instance, Hosmer and Lemeshow (37) suggested that AUC of 0.70 to 0.80 are 'acceptable', 0.80 to 0.90 'excellent' and 0.9 or above 'outstanding', while AUC of 0.50 suggests discrimination by chance e.g. similar to tossing a coin to decide death or alive.

Step 3. Interpreting results to evaluate performance

The two primary questions for the hospital performance evaluation were, (i) has the benchmark value been insufficiently, closely, or fully reached? (ii) are there substantial differences between the hospitals, or do they perform homogenously? To answer both questions, we created a framework (Table 1) with 15 scenarios combining information on the benchmark value achievement and the size of hospital differences based on model 2.

In the best scenario (scenario A) the desired target level has been fully achieved overall (averaging across all hospitals the adjusted mortality rate is less than 6%), and hospital differences are effectively absent (the Hospital GCE is effectively absent). The conclusion would be that all hospitals have performed similarly well. In the worst scenario (scenario C) the desired target level has not been achieved overall, and between-hospital differences are again absent. The conclusion would be that all hospitals have performed similarly but poorly. Observe that in both scenarios A and C, interventions only targeted to specific hospitals are not justifiable. Rather any intervention should be universal (i.e., directed to all hospitals) as in both scenarios all hospitals are performing similarly. In scenario A, the intervention would be oriented to *maintaining* the overall high quality while in scenario C, the objective would be to improve the quality in *all* hospitals.

The interpretation of the scenarios in the lowest corners of the table (M and O) is driven by the very large size of the hospital differences. For example, in scenario M some hospitals may not

For peer review only - http://bmjopen.bmj.com/site/about/guidelines.xhtml

have achieved the target level even though the average 30-day mortality is below the benchmark value. In contrast, in scenario O, some hospitals may have achieved the target level even though the target has not been achieved overall. In these scenarios (M and O) targeted hospitals interventions are justified to focus especially on those hospitals operating above the benchmark value.

Patient and public involvement: No patient and public involved

For peer review only

Results

Table 2 describes the estimation sample. The mean age of the 43,247 patients with a first-ever AMI was approximately to 67 years and only one third were female. As expected, mortality was strongly related to the RS of the patient, so that patients in the highest RS category present a mortality risk about 36 times higher than in the low RS group (25.5% vs. 0.7%).

In the crude, unadjusted analysis based on simple proportions (i.e., obtained without multilevel modeling), 8.18% of the patients died within a 30-day period after admission to the hospital and the crude proportions ranged from 3.45% to 17.32% across hospitals, as indicated in figure 2 (white circles). However, accounting for the reliability of the hospital information in the hierarchical multilevel analysis (Model 1), the mean unadjusted hospital rate from the multilevel model was 8.00% and ranged between 6.27% and 12.21% (grey circles). Thus, the multilevel model was able to extract the systematic variation of interest between hospitals from the observed variation which additionally includes a substantial statistical noise component due to the relatively small patient caseload per hospital (from a statistical sample size perspective). After further adjustment for patient-mix variation across hospitals in the cross-classified multilevel model (model 2) the mean hospital 30-day mortality was 4.78% and varied only between 3.44% and 7.48% (black circles) and the 95% credible intervals (grey vertical lines)

For peer review only - http://bmjopen.bmj.com/site/about/guidelines.xhtml

considerably overlap each other. Thus, once we performed reliability weighted estimations and adjusted for hospital differences in patient mix, very little variation in performance remained across hospitals. These adjusted mortality rates represent hospital rates as if all hospital treated the same patient with average RS (i.e., RS_k = 0), which is a suitable approach for evaluating hospital performance.

Table 3 reports the results from the multilevel variance analysis used to estimate the GCE. In model 1, the VPC_H was 1.20%, and the AUC_H = 0.55. Adjustment for patient-mix by including the RS categories in the cross-classified model 2 reduced the value of the VPC_H= 0.70%, and slightly decreased the initial AUC_H= 0.54. Therefore, the hospital differences were *absent*, suggesting that hospital attended is not a driving factor in determining patient mortality. In contrast, information on the RS value of the patients was much more relevant than information on the hospital in which they were treated. The VPC_{RS} was very large (i.e., 34.13%) and the AUC_{RS}= 0.77. Figure 3 clearly depicts the differential discriminatory accuracies of the hospital and RS random effects. The hospital variance (0.03 (0.02-0.06)), total AUC (0.78 (0.77-0.79)) and Bayesian DIC (2334.2) results from model 3 were similar to model 2.

Discussion

Analyzing 30-day mortality after AMI in Sweden, we illustrate the MAIHDA approach to auditing hospital performance. By considering both the size of the hospital GCE and the RS adjusted hospital 30-day mortality rates in relation to a pre-set benchmark value, we were able to perform a more nuanced evaluation of hospital performance compared with traditional methods focused exclusively on differences between hospital averages.

Following the framework presented in table 1, our results corresponds with the scenario A and indicate that, at the time of our analysis, all hospitals in Sweden were performing homogenously

For peer review only - http://bmjopen.bmj.com/site/about/guidelines.xhtml

well. That is, the overall 30-day mortality (i.e., 4.78%) was under the benchmark value of ≤6% and there were no relevant hospital differences (VPC_H= 0.70% and AUC_H= 0.54). The ranking of hospitals in our study (Figure 2) also shows that no hospitals could be statistically distinguished with any degree of certainty from the overall average mortality.

We have found similar low VPC values when investigating hospital differences in mortality after AMI admission in Ontario, Canada (38) and in Sweden (39) as well as mortality after heart failure in Sweden (40) and in Denmark (3). The low hospital GCE suggests universal instead of targeted interventions, as all hospitals perform homogenously. There may be, however, other patient outcomes where the hospital GCE would be much larger. For instance, when auditing adherence with guidelines for statin prescription (41, 42) or process quality indicators of diabetes care like albuminuria analysis (43).

The evaluation of institutional performance using VPC is not new (12, 39, 44, 45). Normand (46) states that when evaluating hospital performance if the VPC is zero *there are no hospital quality differences, that is, the chance that a patient experiences an event after being treated is the same regardless of the hospital (p. 33)*. This idea is also explicitly expressed by the committee assigned to set statistical guidelines for assessing hospital performance in USA (47). The share of the total variance that is at the hospital level is crucial for evaluating performance. However, this fundamental concept needs be applied more extensively. Today, it is recognized that multilevel models (hierarchical or mixed-effect models) are the preferred methodology for provider profiling. However, the substantive analysis of components of variance still receives little attention and most studies only consider multilevel modeling for its capacity to account for the clustering of patients within hospitals in order to obtain "correct standard errors" on regression coefficients and odds ratios. Some authors even conclude that hospital averages (odds ratios, observed/expected values) obtained from multilevel analyses gives similar results compared to traditional logistic regressions analyses. This situation is interpreted as an

For peer review only - http://bmjopen.bmj.com/site/about/guidelines.xhtml

argument for keeping the traditional logistic regression as the standard method for performing risk adjustment of hospital quality comparisons (48-50). However, we do not agree with this opinion. The first reason is that the fixed effects approach does not explicitly inform on components of variance. The second reason is that the equivalence between traditional and multilevel regression results only occurs when the hospital GCE (i.e., the clustering) is low and the number of patients at the hospitals is very high (i.e., reliable estimation of hospital averages) (26). In other words, traditional non-multilevel analyses give similar results to the multilevel analysis only when the hospital differences are not relevant (i.e., low VPC) and the patient load is very large in every hospital (which is rarely the case). In addition, hospital level variables appear paradoxically more statistically "significant" when the hospital level is less relevant (i.e., low VPC) (7). Information on the size of the hospital GCE is, therefore, fundamental for a sound analysis of hospital performance.

In this study we have applied the AUC to evaluate the hospital GCE. The AUC is a measure of discriminatory accuracy frequently used for gauging the performance of prognostic and screening markers in medicine (8, 9) but it can also be used to quantify hospital GCE (10, 34). So far, many epidemiologists may not be familiar with the use of measures of components of variance like the VPC for binary outcomes (30). However, the AUC measure is well established in clinical and health care epidemiology and the information it gives is relatively easy to interpret and communicate. From this perspective, the evaluation of hospital performance resembles a screening test and so we must therefore know the discriminatory accuracy of, for instance, a "league table" to make informed decisions.

Analyzing outcome indicators always requires adjustment for confounding (i.e., patient-mix). We performed this adjustment using an innovative strategy that use hospitals and decile groups of RS in a cross-classified multilevel analysis. This approach provides a new option that could be very useful in some cases. In other cases, the classical inclusion of the patient-mix

For peer review only - http://bmjopen.bmj.com/site/about/guidelines.xhtml

information as fixed effects may be more suitable. Both approaches provide similar results in terms of the hospital VPC, AUC, and model fit. Then, the investigator may judge which approach is most suitable for the research question. A key advantage of our approach is it allowed a directed comparison of the importance of patient case mix and hospital effects in explaining variation in patient outcomes across hospital. We were able to calculate and compare a VPC for RS categories with the VPC for hospitals. We did not use existing patient-mix adjustment scores such as the Charlson Risk Score (51), the Elixhauser Score (52), or the Center for Medicare & Medicaid Services Hierarchical Condition Category "CMS-HCC" risk adjuster (53). Our aim was not primarily to create a new risk score equation but only to perform a parsimonious patient-mix adjustment where the RS summarizes a large number of variables into a single construct.

This study has some limitations that need to be discussed. Unfortunately, the database for which we have ethical allowance for this study does not provide information on the severity of AMI or on revascularization procedures (e.g., PCI, CABG). The inclusion of these variables could possibly improve the RS. However, we believe this improvement would be small and unlikely to affect our conclusions. Additionally, the RS is not a perfect instrument to quantify the true severity and mortality risk of a patient. Nevertheless, the RS categories we use are strongly associated with mortality and the RS alone shows a high discriminatory accuracy. RS may reflect practice or coding patterns of hospitals. However, Sweden has a very homogenous health care system with centralized diagnostic rules which may reduce the risk of differential diagnosis setting. Finally, to explore the potential loss of information due to the categorization of RS into deciles, we performed a sensitivity analysis with 15 and 20 categories. The results were similar to those obtained in model 2 (data not shown)

In summary, we illustrate the MAIHDA approach to auditing hospital performance using a three-step strategy. We argue that it is necessary to consider both the size of the hospital GCE

For peer review only - http://bmjopen.bmj.com/site/about/guidelines.xhtml

and the RS adjusted 30-day mortality in relation to a pre-set benchmark value. Our results indicate that, at the time of our analysis, all hospitals in Sweden were performing homogenously well. That is, there were no meaningful hospital differences and the benchmark target for 30-day mortality rate after admission for AMI was well achieved. Therefore, possible quality interventions should be universal and oriented to maintain the high hospital quality of care.

Funding

The Swedish Research Council (Vetenskapsrådet) supported this work through the project "Multilevel Analyses of Individual Heterogeneity: innovative concepts and methodological approaches in Public Health and Social Epidemiology": (#2017-01321, PI: Juan Merlo). Dr. Austin received a personal Mid-Career Investigator award from the Heart and Stroke Foundation.

Contributors:

JM had the initiative of the study and acquired the data.

JM and M R-L wrote the original manuscript.

M R-L and RP-V performed the analyses in coordination with JM.

PA and GL provided advanced statistical support.

All the authors have contributed to the design of the study, developed the methodology, direction of the analyses, interpretation of the results and drafting of the manuscript

All authors have revised the last version of the manuscript.

Conflicts of Interest: None declared

For peer review only - http://bmjopen.bmj.com/site/about/guidelines.xhtml

Data availability statement: Data are available upon reasonable request.

Original data are available from the Swedish National Board of Health and Welfare and Statistics, after approval of a research project by an Ethical Committee and by the data safety committees of Swedish Authorities. However, it is possible to replicate the analyses performed, by using a table provided as supplementary information.

References

1. Chung SC, Sundstrom J, Gale CP, James S, Deanfield J, Wallentin L, et al. Comparison of hospital variation in acute myocardial infarction care and outcome between Sweden and United Kingdom: population based cohort study using nationwide clinical registries. BMJ. 2015;351:h3913.
2. van Dishoeck AM, Lingsma HF, Mackenbach JP, Steyerberg EW. Random variation and rankability of hospitals using outcome indicators. BMJ Qual Saf. 2011;20(10):869-74.
3. Ghith N, Wagner P, Frolich A, Merlo J. Short Term Survival after Admission for Heart Failure in Sweden: Applying Multilevel Analyses of Discriminatory Accuracy to Evaluate Institutional Performance. PLoS One. 2016;11(2):e0148187.
4. Merlo J. Invited commentary: multilevel analysis of individual heterogeneity-a fundamental critique of the current probabilistic risk factor epidemiology. Am J Epidemiol. 2014;180(2):208-12; discussion 13-4.
5. Austin PC, Merlo J. Intermediate and advanced topics in multilevel logistic regression analysis. Stat Med. 2017;36(20):3257-77.
6. Merlo J, Viciana-Fernandez FJ, Ramiro-Farinas D, LDAP RG. Bringing the individual back to small-area variation studies: A multilevel analysis of all-cause mortality in Andalusia, Spain. Social Science & Medicine. 2012;75(8):1477-87.
7. Merlo J, Wagner P, Austin PC, Subramanian SV, Leckie G. General and specific contextual effects in multilevel regression analyses and their paradoxical relationship: A conceptual tutorial. SSM Popul Health. 2018;5:33-7.
8. Khoury MJ, Newill CA, Chase GA. Epidemiologic evaluation of screening for risk factors: application to genetic screening. Am J Public Health. 1985;75(10):1204-8.
9. Pepe MS, Janes H, Longton G, Leisenring W, Newcomb P. Limitations of the odds ratio in gauging the performance of a diagnostic, prognostic, or screening marker. Am J Epidemiol. 2004;159(9):882-90.
10. Wagner P, Merlo J. Measures of discriminatory accuracy in multilevel analysis. European Journal of Epidemiology. 2013;28(1, Supplement):135.
11. Merlo J, Wagner P, Ghith N, Leckie G. An Original Stepwise Multilevel Logistic Regression Analysis of Discriminatory Accuracy: The Case of Neighbourhoods and Health. PLoS One. 2016;11(4):e0153778.
12. Merlo J, Ostergren PO, Broms K, Bjorck-Linne A, Liedholm H. Survival after initial hospitalisation for heart failure: a multilevel analysis of patients in Swedish acute care hospitals. J Epidemiol Community Health. 2001;55(5):323-9.
13. Van Leeuwen W, Nilsson S, Merlo J. Mother's country of birth and prescription of psychotropic medication in Swedish adolescents: a life course approach. BMJ Open. 2012;2(5).
14. Snijders TAB, Bosker RJ. Multilevel analysis: an introduction to basic and advanced multilevel modeling. 1 ed. Thousand Oaks, California: SAGE Publications; 1999 1999///.

For peer review only - http://bmjopen.bmj.com/site/about/guidelines.xhtml

15. Merlo J. Multilevel analysis of individual heterogeneity and discriminatory accuracy (MAIHDA) within an intersectional framework. Soc Sci Med. 2018;203:74-80.
16. Krell RW, Hozain A, Kao LS, Dimick JB. Reliability of risk-adjusted outcomes for profiling hospital surgical quality. JAMA surgery. 2014;149(5):467-74.
17. Goldstein H, Spiegelhalter D. League Tables and Their Limitations: Statistical Issues in Comparisons of Institutional Performance. Journal of the Royal Statistical Society, SocA. 1996;159:385-443.
18. Leckie G, Charlton C. runmlwin: A Program to Run the

MLwiN Multilevel Modeling Software from within Stata. Journal of

Statistical Software. 2013;52(11):1-40.
19. Zhang Z, Parker R, Charlton C, Leckie G, Browne WJ. R2MLwiN - A program to run the MLwiN multilevel modelling software from within R. Journal of Statistical Software, . 2016;72(10):1-43.
20. Ludvigsson JF, Andersson E, Ekbom A, Feychting M, Kim JL, Reuterwall C, et al. External review and validation of the Swedish national inpatient register. BMC Public Health. 2011;11:450.
21. Brooke HL, Talback M, Hornblad J, Johansson LA, Ludvigsson JF, Druid H, et al. The Swedish cause of death register. Eur J Epidemiol. 2017;32(9):765-73.
22. Ludvigsson JF, Almqvist C, Bonamy AK, Ljung R, Michaelsson K, Neovius M, et al. Registers of the Swedish total population and their use in medical research. Eur J Epidemiol. 2016;31(2):125-36.
23. Ludvigsson JF, Otterblad-Olausson P, Pettersson BU, Ekbom A. The Swedish personal identity number: possibilities and pitfalls in healthcare and medical research. Eur J Epidemiol. 2009;24(11):659-67.
24. Public Access to Information and Secrecy Act: Government Offices of Sweden; 2009 [Available from: http://www.government.se/information-material/2009/09/public-access-to-information-and-secrecy-act/.
25. Leckie G. Cross-classified Multilevel Models - Concepts. LEMMA VLE Module 12, 1-60. URL: http://wwwbristolacuk/cmm/learning/online-course/. 2013.
26. Evans CR, Leckie G, Merlo J. Multilevel versus single-level regression for the analysis of multilevel information:The case of quantitative intersectional analysis. Social Science & Medicine. 2019.
27. Goldstein H, Browne WJ, Rashbash J. Partitioning variation in multilevel models. Understanding Statistics: Statistical Issues in Psychology, Education, and the Social Sciences. 2002;1(4):223-31.
28. Merlo J, Yang M, Chaix B, Lynch J, Rastam L. A brief conceptual tutorial on multilevel analysis in social epidemiology: investigating contextual phenomena in different groups of people. J Epidemiol Community Health. 2005;59(9):729-36.
29. Merlo J, Chaix B, Yang M, Lynch J, Rastam L. A brief conceptual tutorial of multilevel analysis in social epidemiology: linking the statistical concept of clustering to the idea of contextual phenomenon. J Epidemiol Community Health. 2005;59(6):443-9.
30. Merlo J, Chaix B, Ohlsson H, Beckman A, Johnell K, Hjerpe P, et al. A brief conceptual tutorial of multilevel analysis in social epidemiology: using measures of clustering in multilevel logistic regression to investigate contextual phenomena. J Epidemiol Community Health. 2006;60(4):290-7.
31. Goldstein H, Browne W, Rasbash J. Partitioning variation in generalised linear multilevel models. Understanding Statistics. 2002;1:223-32.
32. Wagner P, Merlo J. Discriminatory accuracy of a random effect in multilevel logistic regression. 20th IEA World Congress of Epidemiology (WCE2014). 2014.
33. Charlton C, Rasbash J, Browne WJ, Healy M, Cameron B. MLwiN Version 3.00. Centre for Multilevel Modelling, University of Bristol.; 2017.

For peer review only - http://bmjopen.bmj.com/site/about/guidelines.xhtml

34. Merlo J, Wagner P, Leckie G. A simple multilevel approach for analysing geographical inequalities in public health reports: The case of municipality differences in obesity. Health Place. 2019;58:102145.
35. OECD. Mortality following acute myocardial infarction (AMI). 2017. In: Health at a Glance 2017: OECD Indicators, [Internet]. Paris: OECD Publishing,.
36. Anderson JL, Morrow DA. Acute Myocardial Infarction. The New England journal of medicine. 2017;376(21):2053-64.
37. Hosmer DW, Lemeshow S. Applied logistic regression. 2nd ed. New York: Wiley; 2000. xii, 373 p. p.
38. Austin PC, Wagner P, Merlo J. The median hazard ratio: a useful measure of variance and general contextual effects in multilevel survival analysis. Stat Med. 2017;36(6):928-38.
39. Merlo J, Hakansson A, Beckman A, Lindblad U, Lindstrom M, Gerdtham UG, et al. [Equally good care of myocardial infarction in Sweden today. Geographic differences in mortality are without significance for the individual patient]. Lakartidningen. 2005;102(1-2):20-3.
40. Merlo J, Ostergren P, Broms K, Bjorck-Linne A, Liedholm H. Survival after initial hospitalisation for heart failure: a multilevel analysis of patients in Swedish acute care hospitals. Journal of Epidemiology and Community Health. 2001;55(5):323-9.
41. Hjerpe P, Ohlsson H, Lindblad U, Bostrom KB, Merlo J. Understanding adherence to therapeutic guidelines: a multilevel analysis of statin prescription in the Skaraborg Primary Care Database. Eur J Clin Pharmacol. 2010.
42. Ohlsson H, Lindblad U, Lithman T, Ericsson B, Gerdtham UG, Melander A, et al. Understanding adherence to official guidelines on statin prescribing in primary health care--a multi-level methodological approach. Eur J Clin Pharmacol. 2005;61(9):657-65.
43. Ghith N, Merlo J, Frolich A. Albuminuria measurement in diabetic care: a multilevel analysis measuring the influence of accreditation on institutional performance. BMJ Open Qual. 2019;8(1):e000449.
44. Ohlsson H, Librero J, Sundquist J, Sundquist K, Merlo J. Performance evaluations and league tables: do they capture variation between organizational units? An analysis of 5 Swedish pharmacological performance indicators. Med Care. 2011;49(3):327-31.
45. Merlo J, Gerdtham UG, Eckerlund I, Hakansson S, Otterblad-Olausson P, Pakkanen M, et al. Hospital level of care and neonatal mortality in low- and high-risk deliveries: reassessing the question in Sweden by multilevel analysis. Med Care. 2005;43(11):1092-100.
46. Normand SLT, Ash SA, Fienberg SE, Stukel TA, Utts J, Louis TA. League Tables for Hospital Comparisons. Annu Rev Stat App. 2016;3:21-50.
47. Ash AS, Fienberg E, Louis A, Norm S-lT, Stukel A, Utts PJ. STATISTICAL ISSUES IN ASSESSING HOSPITAL PERFORMANCE Commissioned by the Committee of Presidents of Statistical Societies The COPSS-CMS White Paper Committee. 2011.
48. Cohen ME, Dimick JB, Bilimoria KY, Ko CY, Richards K, Hall BL. Risk adjustment in the American College of Surgeons National Surgical Quality Improvement Program: a comparison of logistic versus hierarchical modeling. J Am Coll Surg. 2009;209(6):687-93.
49. Glance LG, Dick A, Osler TM, Li Y, Mukamel DB. Impact of changing the statistical methodology on hospital and surgeon ranking - The case of the new York state cardiac surgery report card. Medical Care. 2006;44(4):311-9.
50. Alexandrescu R, Jen MH, Bottle A, Jarman B, Aylin P. Logistic versus hierarchical modeling: an analysis of a statewide inpatient sample. J Am Coll Surg. 2011;213(3):392-401.
51. Charlson ME, Pompei P, Ales KL, MacKenzie CR. A new method of classifying prognostic comorbidity in longitudinal studies: Development and validation. Journal of Chronic Diseases. 1987;40(5):373-83.
52. Elixhauser A, Steiner C, Harris DR, Coffey RM. Comorbidity Measures for Use with Administrative Data. Medical Care. 1998;36(1):8-27.

For peer review only - http://bmjopen.bmj.com/site/about/guidelines.xhtml

53. Li P, Kim M, Doshi J. Comparison of the performance of the CMS Hierarchical Condition Category (CMS-HCC) risk adjuster with the charlson and elixhauser comorbidity measures in predicting mortality. BMC Health Services Research. 2010;10(1):245.

For peer review only

For peer review only - http://bmjopen.bmj.com/site/about/guidelines.xhtml

Table 1. Framework for performing hospital comparisons of 30-day mortality after acute myocardial infarction (AMI).

Size of hospital differences (Hospital GCE)			Benchmark value achievement		
			Full	Close	Insufficient
	VPC_H (%)	AUC_H	< 6%	6% - 8%	> 8%
Absent	0 to 1	0.50 to 0.55	A	B	C
Small	1 to 5	0.55 to 0.61	D	E	F
Moderate	5 to 10	0.61 to 0.66	G	H	I
large	10 to 20	0.66 to 0.72	J	K	L
Very large	>20	>0.72	M	N	O

For peer review only - http://bmjopen.bmj.com/site/about/guidelines.xhtml

Table 2. Characteristics of the population of 43,247 patients with a first-ever acute myocardial infarction (AMI) cared for at 68 Swedish hospitals in 2007-2009 and categorized in 10 groups by deciles of risk score for 30-day mortality (RS).

Age, *mean (SD)*	67.3 (9.0)	
Female *(%)*	33	
Number of hospitals	68	
Number of patients at the hospital, *Median (min-max)*	636 (107 - 3037)	
	Mean RS (%)	Observed mortality (
Risk Score for 30-day mortality (RS)	7.8	7.8
Decile groups of RS		
1st	1.7	0.7
2nd	2.3	1.4
3rd	2.9	2.0
4th	3.5	2.5
5th	4.2	3.7
6th	5.3	5.9
7th	6.9	8.2
8th	9.5	12.2
9th	14.0	16.1
10th	28.0	25.5

nly

For peer review only - http://bmjopen.bmj.com/site/about/guidelines.xhtml

Table 3. Multilevel modelling of 30-day mortality in 43,247 patients with a first-ever acute myocardial infarction (AMI) in 2008-2009 in Sweden. Model 1 includes only a random effect for the hospital, model 2 is a cross-classified multilevel analysis including both hospital and risk score (RS) categories as random effects.

		Model 1	Model 2
Overall 30-day mortality mean (min – max), %		8.00 (6.27 –12.21)	4.78 (3.44 –7.48)
Variance			
	Hospital	0.04 (0.02 – 0.07)	0.03 (0.01 – 0.06)
	RS category		1.96 (0.66 – 4.53)
VPC (%)			
	Hospital	1.20(0.56 – 2.22)	0.70 (0.26–1.34)
	RS category		34.13 (16.64–57.77)
AUC_H			
	Hospital	0.55 (0.55 – 0.57)	0.54 (0.53 – 0.55)
	RS category		0.77 (0.77 – 0.78)
	Total	0.55 (0.55 – 0.57)	0.78 (0.77 – 0.79)
DIC		5480.7	2333.5

VPC: Variance Partition Coefficient, AUC: Area Under the Receiver Operating Characteristics Curve. DIC: Deviance Information Criterion

Figure legends an caption:

Figure 1. Flow diagram showing the selection of patients with first diagnosis of acute myocardial infarction (AMI) 2007-2009 who were included in the study population.

Figure 2. Crude (white circles), reliability weighted (grey circles) and both reliability weighted and patient-mix adjusted (black circles and confidence intervals) differences in absolute risk of 30-day mortality between hospitals in the population of 43,247 patients with a first-ever acute myocardial infarction in 2007-2009, in Sweden.

Figure 3. Area under the Receiver Operating Characteristics Curve (AUC) constructed using the predicted probability of 30-day mortality obtained from multilevel logistic regression analysis with 47,462 patients with a discharge diagnosis of acute myocardial infarction (AMI) in 2007-2009 in Sweden. The AUC for the unadjusted hospitals was obtained from model 1 with patient nested within hospitals (Blue line). The rest of the curves are from model 2, a cross-classified multilevel model with patients nested within hospitals and categories (decile groups) of risk-score (RS) for 30-day mortality. (Red line: patient mix adjusted hospitals, Orange line: Risk score category, Green line: Total AUC)

For peer review only

For peer review only

For peer review only - http://bmjopen.bmj.com/site/about/guidelines.xhtml

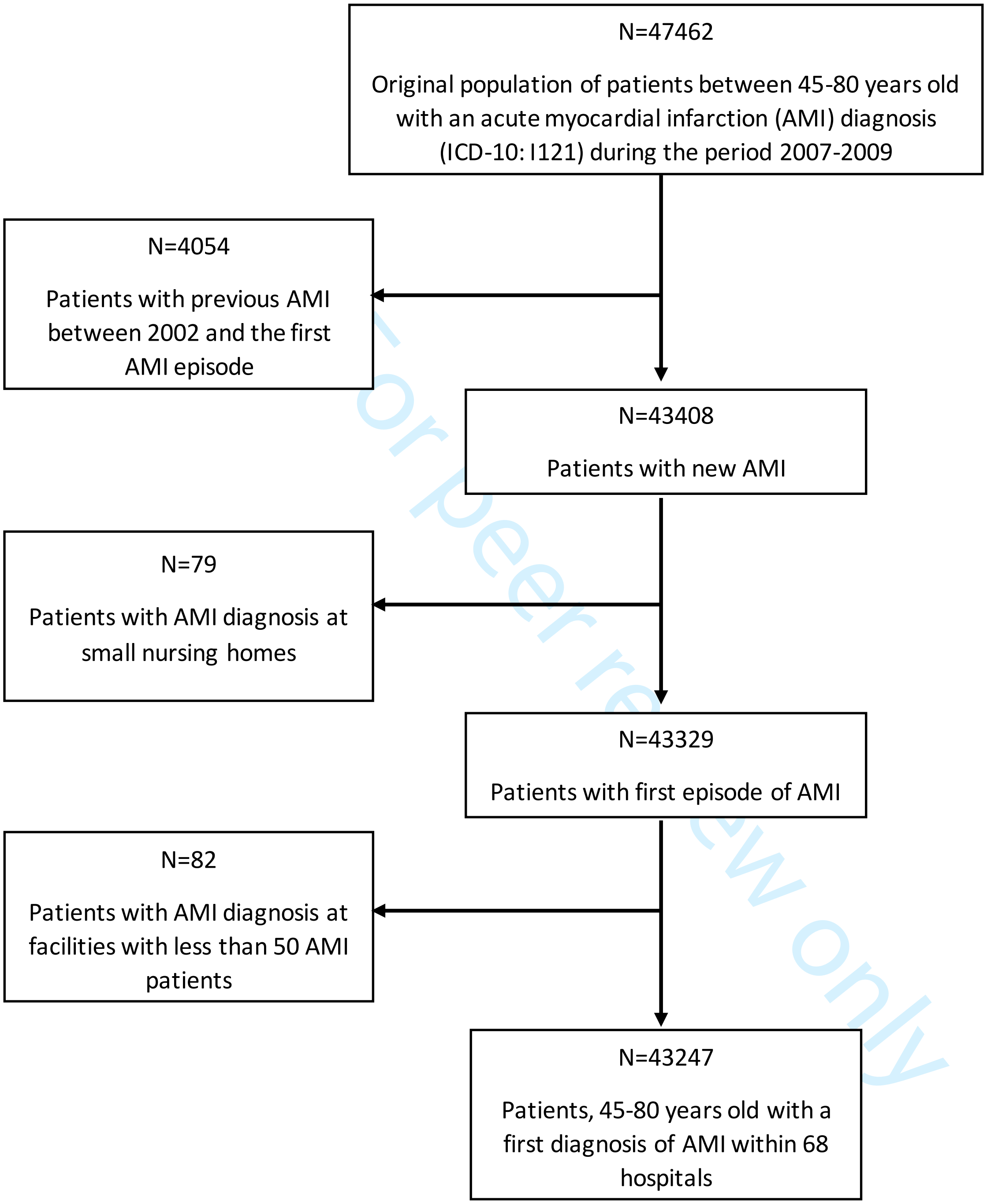
N=47462
Original population of patients between 45-80 years old with an acute myocardial infarction (AMI) diagnosis (ICD-10: I121) during the period 2007-2009
N=4054
Patients with previous AMI between 2002 and the first AMI episode
N=43408
Patients with new AMI
N=79
Patients with AMI diagnosis at small nursing homes
N=43329
Patients with first episode of AMI
N=82
Patients with AMI diagnosis at facilities with less than 50 AMI patients
N=43247
Patients, 45-80 years old with a first diagnosis of AMI within 68 hospitals

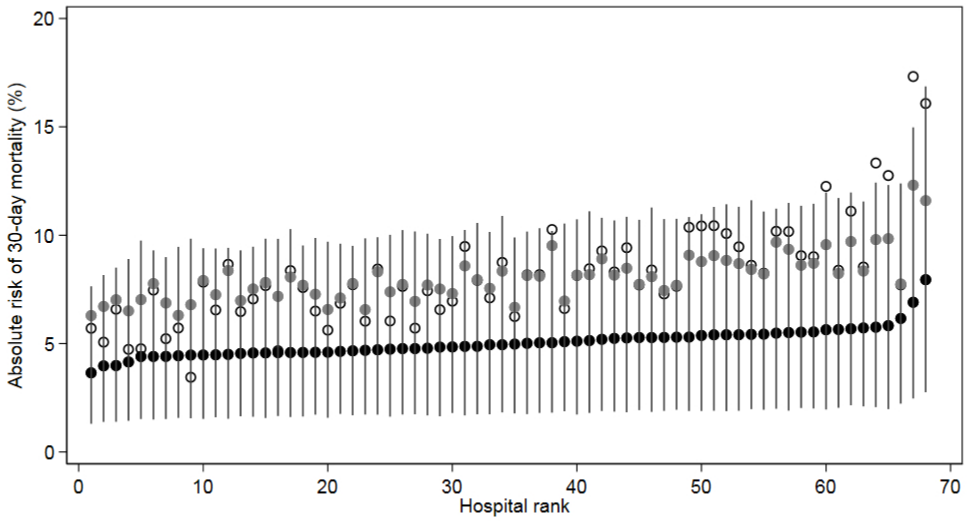

Crude (white circles), reliability weighted (grey circles) and both reliability weighted and patient-mix adjusted (black circles and confidence intervals) differences in absolute risk of 30-day mortality between hospitals in the population of 43,247 patients with a first-ever acute myocardial infarction in 2007-2009, in Sweden.

352x194mm (72 x 72 DPI)

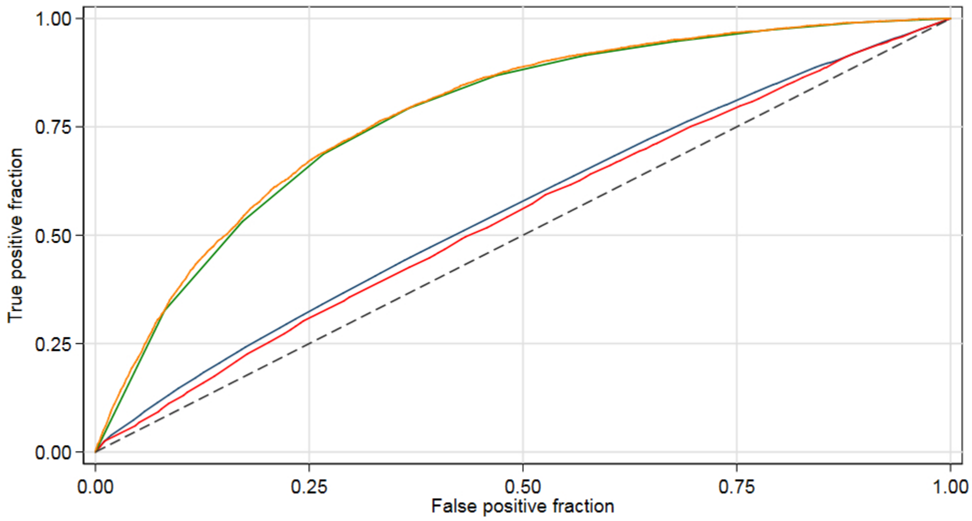

Area under the Receiver Operating Characteristics Curve (AUC) constructed using the predicted probability of 30-day mortality obtained from multilevel logistic regression analysis with 47,462 patients with a discharge diagnosis of acute myocardial infarction (AMI) in 2007-2009 in Sweden. The AUC for the unadjusted hospitals was obtained from model 1 with patient nested within hospitals (Blue line). The rest of the curves are from model 2, a cross-classified multilevel model with patients nested within hospitals and categories (decile groups) of risk-score (RS) for 30-day mortality. (Red line: patient mix adjusted hospitals, Orange line: Risk score category, Green line: Total AUC)

352x194mm (72 x 72 DPI)

hospital	cons	rscategory	stratum	proportion	numerator	denominator
1	1	1	1	0.00	0	99
1	1	2	2	0.01	1	76
1	1	3	3	0.03	2	73
1	1	4	4	0.02	1	62
1	1	5	5	0.01	1	75
1	1	6	6	0.05	4	87
1	1	7	7	0.07	8	108
1	1	8	8	0.06	7	112
1	1	9	9	0.09	11	118
1	1	10	10	0.22	26	116
2	1	1	11	0.00	0	196
2	1	2	12	0.01	1	179
2	1	3	13	0.01	1	154
2	1	4	14	0.03	4	136
2	1	5	15	0.04	7	158
2	1	6	16	0.04	7	159
2	1	7	17	0.06	9	152
2	1	8	18	0.14	27	189
2	1	9	19	0.18	34	193
2	1	10	20	0.22	42	195
3	1	1	21	0.01	3	249
3	1	2	22	0.02	4	189
3	1	3	23	0.02	3	172
3	1	4	24	0.04	7	186
3	1	5	25	0.07	10	149
3	1	6	26	0.07	11	166
3	1	7	27	0.09	12	129
3	1	8	28	0.16	20	128
3	1	9	29	0.22	21	94
3	1	10	30	0.31	29	95
4	1	1	31	0.01	1	99
4	1	2	32	0.01	1	69
4	1	3	33	0.03	2	76
4	1	4	34	0.04	3	72
4	1	5	35	0.05	4	78
4	1	6	36	0.10	9	88
4	1	7	37	0.08	7	92
4	1	8	38	0.12	10	86
4	1	9	39	0.21	19	91
4	1	10	40	0.20	25	122
5	1	1	41	0.00	0	127
5	1	2	42	0.00	0	97
5	1	3	43	0.02	2	113
5	1	4	44	0.00	0	100
5	1	5	45	0.00	0	93
5	1	6	46	0.05	5	102

For peer review only

5	1	7	47	0.05	6	110
5	1	8	48	0.06	9	145
5	1	9	49	0.07	10	140
5	1	10	50	0.24	35	147
6	1	1	51	0.00	0	26
6	1	2	52	0.00	0	22
6	1	3	53	0.03	1	30
6	1	4	54	0.05	2	37
6	1	5	55	0.09	2	22
6	1	6	56	0.05	2	37
6	1	7	57	0.06	2	32
6	1	8	58	0.13	4	32
6	1	9	59	0.11	3	28
6	1	10	60	0.22	8	37
7	1	1	61	0.00	0	26
7	1	2	62	0.00	0	19
7	1	3	63	0.06	1	18
7	1	4	64	0.00	0	23
7	1	5	65	0.12	3	26
7	1	6	66	0.07	2	27
7	1	7	67	0.00	0	26
7	1	8	68	0.14	5	37
7	1	9	69	0.28	10	36
7	1	10	70	0.22	7	32
8	1	1	71	0.00	0	157
8	1	2	72	0.02	4	167
8	1	3	73	0.01	1	147
8	1	4	74	0.05	6	133
8	1	5	75	0.04	5	135
8	1	6	76	0.07	9	123
8	1	7	77	0.03	5	154
8	1	8	78	0.13	16	126
8	1	9	79	0.09	12	139
8	1	10	80	0.29	39	133
9	1	1	81	0.00	0	27
9	1	2	82	0.00	0	23
9	1	3	83	0.00	0	14
9	1	4	84	0.00	0	7
9	1	5	85	0.07	1	14
9	1	6	86	0.00	0	15
9	1	7	87	0.00	0	7
9	1	8	88	0.08	1	13
9	1	9	89	0.00	0	11
9	1	10	90	0.21	3	14
10	1	1	91	0.00	0	62
10	1	2	92	0.03	3	93
10	1	3	93	0.01	1	84

For peer review only

10	1	4	94	0.01	1	73
10	1	5	95	0.04	3	78
10	1	6	96	0.07	6	90
10	1	7	97	0.07	6	87
10	1	8	98	0.23	14	62
10	1	9	99	0.16	11	69
10	1	10	100	0.34	18	53
11	1	1	101	0.04	1	24
11	1	2	102	0.04	1	28
11	1	3	103	0.03	1	37
11	1	4	104	0.00	0	50
11	1	5	105	0.03	1	37
11	1	6	106	0.07	2	28
11	1	7	107	0.08	3	38
11	1	8	108	0.06	2	31
11	1	9	109	0.04	1	27
11	1	10	110	0.40	6	15
12	1	1	111	0.00	0	10
12	1	2	112	0.00	0	19
12	1	3	113	0.00	0	13
12	1	4	114	0.00	0	28
12	1	5	115	0.00	0	14
12	1	6	116	0.07	2	28
12	1	7	117	0.14	3	21
12	1	8	118	0.00	0	18
12	1	9	119	0.24	4	17
12	1	10	120	0.14	2	14
13	1	1	121	0.00	0	135
13	1	2	122	0.01	1	137
13	1	3	123	0.04	5	141
13	1	4	124	0.02	2	133
13	1	5	125	0.01	2	136
13	1	6	126	0.04	5	134
13	1	7	127	0.10	12	125
13	1	8	128	0.09	8	94
13	1	9	129	0.14	14	101
13	1	10	130	0.24	21	88
14	1	1	131	0.02	1	45
14	1	2	132	0.00	0	44
14	1	3	133	0.00	0	42
14	1	4	134	0.03	2	68
14	1	5	135	0.04	3	68
14	1	6	136	0.06	3	52
14	1	7	137	0.07	4	58
14	1	8	138	0.16	10	62
14	1	9	139	0.20	12	61
14	1	10	140	0.30	24	80

For peer review only

15	1	1	141	0.03	1	32
15	1	2	142	0.00	0	17
15	1	3	143	0.00	0	35
15	1	4	144	0.08	2	25
15	1	5	145	0.11	3	28
15	1	6	146	0.05	1	19
15	1	7	147	0.04	1	25
15	1	8	148	0.17	4	23
15	1	9	149	0.23	5	22
15	1	10	150	0.17	4	24
16	1	1	151	0.00	0	74
16	1	2	152	0.01	1	94
16	1	3	153	0.00	0	85
16	1	4	154	0.00	0	100
16	1	5	155	0.03	3	91
16	1	6	156	0.12	10	82
16	1	7	157	0.06	6	93
16	1	8	158	0.10	9	86
16	1	9	159	0.16	10	64
16	1	10	160	0.20	16	80
17	1	1	161	0.00	0	18
17	1	2	162	0.03	1	35
17	1	3	163	0.00	0	28
17	1	4	164	0.06	2	34
17	1	5	165	0.07	3	41
17	1	6	166	0.11	4	38
17	1	7	167	0.05	2	39
17	1	8	168	0.09	4	43
17	1	9	169	0.14	4	29
17	1	10	170	0.36	12	33
18	1	1	171	0.07	2	27
18	1	2	172	0.04	1	23
18	1	3	173	0.00	0	24
18	1	4	174	0.00	0	26
18	1	5	175	0.00	0	30
18	1	6	176	0.08	2	26
18	1	7	177	0.07	2	27
18	1	8	178	0.06	2	34
18	1	9	179	0.08	3	39
18	1	10	180	0.08	3	40
19	1	1	181	0.00	0	34
19	1	2	182	0.00	0	40
19	1	3	183	0.00	0	38
19	1	4	184	0.08	3	39
19	1	5	185	0.00	0	45
19	1	6	186	0.00	0	37
19	1	7	187	0.08	4	53

For peer review only

19	1	8	188	0.11	6	56
19	1	9	189	0.15	9	60
19	1	10	190	0.24	13	54
20	1	1	191	0.00	0	20
20	1	2	192	0.06	1	18
20	1	3	193	0.00	0	13
20	1	4	194	0.05	1	20
20	1	5	195	0.05	1	20
20	1	6	196	0.04	1	23
20	1	7	197	0.04	1	25
20	1	8	198	0.16	4	25
20	1	9	199	0.20	4	20
20	1	10	200	0.24	4	17
21	1	1	201	0.00	0	21
21	1	2	202	0.00	0	20
21	1	3	203	0.00	0	18
21	1	4	204	0.00	0	25
21	1	5	205	0.07	2	29
21	1	6	206	0.07	2	30
21	1	7	207	0.14	3	22
21	1	8	208	0.11	3	28
21	1	9	209	0.28	8	29
21	1	10	210	0.27	7	26
22	1	1	211	0.00	0	85
22	1	2	212	0.01	1	107
22	1	3	213	0.03	4	117
22	1	4	214	0.02	3	122
22	1	5	215	0.04	4	111
22	1	6	216	0.04	4	98
22	1	7	217	0.14	13	94
22	1	8	218	0.11	11	100
22	1	9	219	0.14	13	91
22	1	10	220	0.21	17	81
23	1	1	221	0.06	1	17
23	1	2	222	0.05	1	22
23	1	3	223	0.00	0	16
23	1	4	224	0.04	1	28
23	1	5	225	0.00	0	22
23	1	6	226	0.08	2	26
23	1	7	227	0.16	6	38
23	1	8	228	0.00	0	23
23	1	9	229	0.15	4	26
23	1	10	230	0.13	3	24
24	1	1	231	0.00	0	27
24	1	2	232	0.08	3	38
24	1	3	233	0.00	0	34
24	1	4	234	0.00	0	28

For peer review only

24	1	5	235	0.03	1	33
24	1	6	236	0.12	5	43
24	1	7	237	0.05	2	37
24	1	8	238	0.13	5	39
24	1	9	239	0.17	5	29
24	1	10	240	0.24	7	29
25	1	1	241	0.01	1	101
25	1	2	242	0.02	2	100
25	1	3	243	0.03	3	94
25	1	4	244	0.03	3	113
25	1	5	245	0.07	8	109
25	1	6	246	0.09	10	113
25	1	7	247	0.10	10	104
25	1	8	248	0.16	14	89
25	1	9	249	0.21	18	85
25	1	10	250	0.20	15	76
26	1	1	251	0.00	0	41
26	1	2	252	0.00	0	43
26	1	3	253	0.00	0	48
26	1	4	254	0.00	0	47
26	1	5	255	0.04	2	56
26	1	6	256	0.02	1	44
26	1	7	257	0.04	2	53
26	1	8	258	0.09	5	57
26	1	9	259	0.10	7	68
26	1	10	260	0.32	25	79
27	1	1	261	0.00	0	9
27	1	2	262	0.00	0	17
27	1	3	263	0.00	0	17
27	1	4	264	0.05	1	21
27	1	5	265	0.00	0	18
27	1	6	266	0.00	0	20
27	1	7	267	0.07	1	14
27	1	8	268	0.00	0	26
27	1	9	269	0.14	5	35
27	1	10	270	0.29	11	38
28	1	1	271	0.06	1	16
28	1	2	272	0.00	0	20
28	1	3	273	0.00	0	17
28	1	4	274	0.00	0	19
28	1	5	275	0.00	0	33
28	1	6	276	0.06	1	16
28	1	7	277	0.12	2	17
28	1	8	278	0.09	2	23
28	1	9	279	0.21	7	34
28	1	10	280	0.11	5	46
29	1	1	281	0.00	0	145

For peer review only

29	1	2	282	0.01	1	159
29	1	3	283	0.02	2	133
29	1	4	284	0.03	3	114
29	1	5	285	0.05	6	130
29	1	6	286	0.05	7	142
29	1	7	287	0.09	13	145
29	1	8	288	0.11	17	151
29	1	9	289	0.11	17	161
29	1	10	290	0.26	44	170
30	1	1	291	0.00	1	245
30	1	2	292	0.02	5	222
30	1	3	293	0.03	7	223
30	1	4	294	0.02	5	218
30	1	5	295	0.03	5	200
30	1	6	296	0.07	16	227
30	1	7	297	0.08	13	166
30	1	8	298	0.16	27	170
30	1	9	299	0.16	33	201
30	1	10	300	0.26	44	169
31	1	1	301	0.00	0	30
31	1	2	302	0.06	1	17
31	1	3	303	0.00	0	23
31	1	4	304	0.00	0	13
31	1	5	305	0.00	0	15
31	1	6	306	0.00	0	16
31	1	7	307	0.08	1	12
31	1	8	308	0.17	4	24
31	1	9	309	0.13	2	16
31	1	10	310	0.40	10	25
32	1	1	311	0.01	1	95
32	1	2	312	0.00	0	86
32	1	3	313	0.01	1	74
32	1	4	314	0.03	2	61
32	1	5	315	0.03	2	65
32	1	6	316	0.08	6	79
32	1	7	317	0.04	3	71
32	1	8	318	0.13	10	78
32	1	9	319	0.14	13	90
32	1	10	320	0.31	26	83
33	1	1	321	0.00	0	39
33	1	2	322	0.00	0	19
33	1	3	323	0.00	0	38
33	1	4	324	0.00	0	39
33	1	5	325	0.02	1	53
33	1	6	326	0.02	1	42
33	1	7	327	0.11	5	46
33	1	8	328	0.09	5	57

For peer review only

33	1	9	329	0.16	11	70
33	1	10	330	0.23	19	82
34	1	1	331	0.02	1	61
34	1	2	332	0.02	1	49
34	1	3	333	0.00	0	35
34	1	4	334	0.04	2	57
34	1	5	335	0.02	1	52
34	1	6	336	0.05	3	60
34	1	7	337	0.09	7	75
34	1	8	338	0.12	10	84
34	1	9	339	0.13	12	92
34	1	10	340	0.28	33	117
35	1	1	341	0.00	0	26
35	1	2	342	0.03	1	36
35	1	3	343	0.08	3	38
35	1	4	344	0.00	0	34
35	1	5	345	0.03	1	29
35	1	6	346	0.08	3	39
35	1	7	347	0.10	4	42
35	1	8	348	0.09	4	45
35	1	9	349	0.24	9	38
35	1	10	350	0.33	17	51
36	1	1	351	0.01	2	331
36	1	2	352	0.01	3	316
36	1	3	353	0.02	6	319
36	1	4	354	0.03	8	305
36	1	5	355	0.03	9	321
36	1	6	356	0.06	17	306
36	1	7	357	0.08	27	319
36	1	8	358	0.13	40	303
36	1	9	359	0.21	54	256
36	1	10	360	0.26	68	261
37	1	1	361	0.04	1	26
37	1	2	362	0.00	0	30
37	1	3	363	0.03	1	36
37	1	4	364	0.00	0	35
37	1	5	365	0.02	1	43
37	1	6	366	0.07	2	30
37	1	7	367	0.05	2	39
37	1	8	368	0.14	5	37
37	1	9	369	0.13	6	47
37	1	10	370	0.25	14	56
38	1	1	371	0.00	0	96
38	1	2	372	0.02	2	88
38	1	3	373	0.02	2	97
38	1	4	374	0.02	2	96
38	1	5	375	0.07	8	113

For peer review only

38	1	6	376	0.07	7	107
38	1	7	377	0.10	12	119
38	1	8	378	0.16	20	124
38	1	9	379	0.18	27	151
38	1	10	380	0.24	36	148
39	1	1	381	0.01	1	78
39	1	2	382	0.02	2	87
39	1	3	383	0.01	1	80
39	1	4	384	0.04	3	73
39	1	5	385	0.03	3	89
39	1	6	386	0.03	3	89
39	1	7	387	0.13	8	64
39	1	8	388	0.15	15	98
39	1	9	389	0.16	14	88
39	1	10	390	0.32	24	75
40	1	1	391	0.00	0	12
40	1	2	392	0.00	0	18
40	1	3	393	0.00	0	12
40	1	4	394	0.00	0	15
40	1	5	395	0.03	1	32
40	1	6	396	0.06	1	18
40	1	7	397	0.00	0	18
40	1	8	398	0.24	5	21
40	1	9	399	0.32	7	22
40	1	10	400	0.39	11	28
41	1	1	401	0.01	1	92
41	1	2	402	0.02	2	128
41	1	3	403	0.03	3	114
41	1	4	404	0.01	1	141
41	1	5	405	0.06	7	124
41	1	6	406	0.11	14	131
41	1	7	407	0.10	14	138
41	1	8	408	0.11	16	149
41	1	9	409	0.15	23	151
41	1	10	410	0.21	24	117
42	1	1	411	0.00	0	101
42	1	2	412	0.00	0	108
42	1	3	413	0.01	2	146
42	1	4	414	0.05	6	131
42	1	5	415	0.07	9	130
42	1	6	416	0.05	6	123
42	1	7	417	0.13	14	109
42	1	8	418	0.07	5	71
42	1	9	419	0.14	12	85
42	1	10	420	0.25	17	69
43	1	1	421	0.00	0	17
43	1	2	422	0.00	0	26

For peer review only

43	1	3	423	0.00	0	24
43	1	4	424	0.07	1	14
43	1	5	425	0.00	0	23
43	1	6	426	0.00	0	17
43	1	7	427	0.04	1	23
43	1	8	428	0.30	6	20
43	1	9	429	0.15	3	20
43	1	10	430	0.23	11	48
44	1	1	431	0.00	0	11
44	1	2	432	0.00	0	18
44	1	3	433	0.00	0	14
44	1	4	434	0.00	0	7
44	1	5	435	0.00	0	14
44	1	6	436	0.00	0	17
44	1	7	437	0.14	3	21
44	1	8	438	0.22	4	18
44	1	9	439	0.39	9	23
44	1	10	440	0.27	6	22
45	1	1	441	0.01	2	137
45	1	2	442	0.02	3	183
45	1	3	443	0.04	5	141
45	1	4	444	0.03	5	153
45	1	5	445	0.03	4	135
45	1	6	446	0.06	7	124
45	1	7	447	0.09	11	117
45	1	8	448	0.06	7	117
45	1	9	449	0.16	12	76
45	1	10	450	0.28	23	81
46	1	1	451	0.00	0	16
46	1	2	452	0.00	0	20
46	1	3	453	0.05	1	19
46	1	4	454	0.07	1	14
46	1	5	455	0.11	3	28
46	1	6	456	0.12	3	26
46	1	7	457	0.22	6	27
46	1	8	458	0.15	5	33
46	1	9	459	0.24	7	29
46	1	10	460	0.43	18	42
47	1	1	461	0.00	0	8
47	1	2	462	0.06	1	16
47	1	3	463	0.10	2	20
47	1	4	464	0.06	1	17
47	1	5	465	0.00	0	20
47	1	6	466	0.16	3	19
47	1	7	467	0.03	1	31
47	1	8	468	0.11	2	19
47	1	9	469	0.29	7	24

For peer review only

47	1	10	470	0.27	8	30
48	1	1	471	0.03	2	71
48	1	2	472	0.00	0	71
48	1	3	473	0.00	0	63
48	1	4	474	0.01	1	69
48	1	5	475	0.02	1	58
48	1	6	476	0.06	4	72
48	1	7	477	0.08	6	72
48	1	8	478	0.16	15	93
48	1	9	479	0.13	11	84
48	1	10	480	0.22	15	67
49	1	1	481	0.00	0	24
49	1	2	482	0.00	0	16
49	1	3	483	0.00	0	30
49	1	4	484	0.00	0	35
49	1	5	485	0.02	1	43
49	1	6	486	0.00	0	43
49	1	7	487	0.08	4	48
49	1	8	488	0.12	5	43
49	1	9	489	0.20	7	35
49	1	10	490	0.18	6	34
50	1	1	491	0.00	0	129
50	1	2	492	0.03	3	108
50	1	3	493	0.01	2	136
50	1	4	494	0.01	1	122
50	1	5	495	0.02	2	98
50	1	6	496	0.05	5	99
50	1	7	497	0.06	7	113
50	1	8	498	0.14	10	72
50	1	9	499	0.12	12	98
50	1	10	500	0.30	21	69
51	1	1	501	0.00	0	21
51	1	2	502	0.00	0	25
51	1	3	503	0.00	0	35
51	1	4	504	0.04	1	25
51	1	5	505	0.06	2	36
51	1	6	506	0.12	5	43
51	1	7	507	0.04	1	27
51	1	8	508	0.14	3	21
51	1	9	509	0.18	8	45
51	1	10	510	0.19	8	42
52	1	1	511	0.00	0	3
52	1	2	512	0.00	0	9
52	1	3	513	0.00	0	9
52	1	4	514	0.00	0	17
52	1	5	515	0.00	0	15
52	1	6	516	0.00	0	12

For peer review only

52	1	7	517	0.22	2	9
52	1	8	518	0.08	1	12
52	1	9	519	0.00	0	10
52	1	10	520	0.18	2	11
53	1	1	521	0.00	0	7
53	1	2	522	0.00	0	12
53	1	3	523	0.00	0	18
53	1	4	524	0.08	1	13
53	1	5	525	0.00	0	13
53	1	6	526	0.00	0	23
53	1	7	527	0.08	1	12
53	1	8	528	0.07	1	14
53	1	9	529	0.14	3	21
53	1	10	530	0.07	1	14
54	1	1	531	0.03	3	97
54	1	2	532	0.02	2	122
54	1	3	533	0.03	4	117
54	1	4	534	0.02	2	119
54	1	5	535	0.03	3	107
54	1	6	536	0.03	4	122
54	1	7	537	0.10	12	119
54	1	8	538	0.11	11	101
54	1	9	539	0.19	15	81
54	1	10	540	0.32	34	106
55	1	1	541	0.00	0	27
55	1	2	542	0.00	0	53
55	1	3	543	0.00	0	30
55	1	4	544	0.00	0	38
55	1	5	545	0.00	0	37
55	1	6	546	0.00	0	38
55	1	7	547	0.08	3	39
55	1	8	548	0.10	3	29
55	1	9	549	0.13	4	31
55	1	10	550	0.19	7	37
56	1	1	551	0.02	1	47
56	1	2	552	0.00	0	38
56	1	3	553	0.04	2	51
56	1	4	554	0.02	1	51
56	1	5	555	0.00	0	37
56	1	6	556	0.06	3	47
56	1	7	557	0.06	3	47
56	1	8	558	0.14	6	42
56	1	9	559	0.18	8	44
56	1	10	560	0.22	7	32
57	1	1	561	0.01	1	69
57	1	2	562	0.00	0	73
57	1	3	563	0.01	1	87

For peer review only

57	1	4	564	0.03	3	90
57	1	5	565	0.05	4	88
57	1	6	566	0.05	4	74
57	1	7	567	0.08	7	83
57	1	8	568	0.14	11	76
57	1	9	569	0.18	12	68
57	1	10	570	0.31	25	81
58	1	1	571	0.00	0	22
58	1	2	572	0.04	1	23
58	1	3	573	0.00	0	42
58	1	4	574	0.06	2	35
58	1	5	575	0.00	0	34
58	1	6	576	0.06	2	33
58	1	7	577	0.00	0	34
58	1	8	578	0.17	7	42
58	1	9	579	0.11	3	28
58	1	10	580	0.20	6	30
59	1	1	581	0.00	0	12
59	1	2	582	0.04	1	27
59	1	3	583	0.04	1	28
59	1	4	584	0.04	1	23
59	1	5	585	0.04	1	26
59	1	6	586	0.13	2	15
59	1	7	587	0.05	1	19
59	1	8	588	0.00	0	30
59	1	9	589	0.22	4	18
59	1	10	590	0.30	7	23
60	1	1	591	0.02	1	66
60	1	2	592	0.02	1	63
60	1	3	593	0.04	3	71
60	1	4	594	0.00	0	80
60	1	5	595	0.02	1	62
60	1	6	596	0.05	3	59
60	1	7	597	0.11	6	53
60	1	8	598	0.07	3	46
60	1	9	599	0.18	8	44
60	1	10	600	0.16	7	43
61	1	1	601	0.01	1	150
61	1	2	602	0.02	2	120
61	1	3	603	0.03	4	119
61	1	4	604	0.04	4	111
61	1	5	605	0.02	2	91
61	1	6	606	0.04	3	83
61	1	7	607	0.11	10	90
61	1	8	608	0.17	13	76
61	1	9	609	0.12	12	97
61	1	10	610	0.29	23	78

For peer review only

62	1	1	611	0.00	0	31
62	1	2	612	0.04	1	25
62	1	3	613	0.00	0	31
62	1	4	614	0.00	0	35
62	1	5	615	0.06	2	36
62	1	6	616	0.06	2	34
62	1	7	617	0.11	3	28
62	1	8	618	0.08	3	37
62	1	9	619	0.13	6	48
62	1	10	620	0.20	7	35
63	1	1	621	0.00	0	17
63	1	2	622	0.00	0	18
63	1	3	623	0.10	2	21
63	1	4	624	0.04	1	23
63	1	5	625	0.04	1	26
63	1	6	626	0.00	0	20
63	1	7	627	0.05	1	21
63	1	8	628	0.18	3	17
63	1	9	629	0.11	2	18
63	1	10	630	0.18	3	17
64	1	1	631	0.00	0	31
64	1	2	632	0.00	0	25
64	1	3	633	0.13	4	32
64	1	4	634	0.15	3	20
64	1	5	635	0.19	6	31
64	1	6	636	0.07	2	27
64	1	7	637	0.16	4	25
64	1	8	638	0.26	6	23
64	1	9	639	0.35	9	26
64	1	10	640	0.47	7	15
65	1	1	641	0.00	0	32
65	1	2	642	0.00	0	22
65	1	3	643	0.00	0	24
65	1	4	644	0.00	0	28
65	1	5	645	0.00	0	32
65	1	6	646	0.05	2	44
65	1	7	647	0.00	0	29
65	1	8	648	0.12	3	25
65	1	9	649	0.24	7	29
65	1	10	650	0.14	3	22
66	1	1	651	0.00	0	18
66	1	2	652	0.00	0	30
66	1	3	653	0.00	0	16
66	1	4	654	0.10	2	21
66	1	5	655	0.00	0	28
66	1	6	656	0.05	1	20
66	1	7	657	0.04	1	25

For peer review only

66	1	8	658	0.16	5	31
66	1	9	659	0.24	7	29
66	1	10	660	0.32	10	31
67	1	1	661	0.00	0	17
67	1	2	662	0.00	0	12
67	1	3	663	0.05	1	21
67	1	4	664	0.00	0	16
67	1	5	665	0.00	0	11
67	1	6	666	0.17	2	12
67	1	7	667	0.00	0	15
67	1	8	668	0.11	2	19
67	1	9	669	0.21	4	19
67	1	10	670	0.38	8	21
68	1	1	671	0.00	0	70
68	1	2	672	0.00	0	48
68	1	3	673	0.00	0	57
68	1	4	674	0.03	2	61
68	1	5	675	0.04	2	51
68	1	6	676	0.07	3	45
68	1	7	677	0.13	7	55
68	1	8	678	0.14	9	66
68	1	9	679	0.20	11	56
68	1	10	680	0.31	17	54

For peer review only

```
**********************************************************************************
* A Cross-Classified Multilevel Analysis of Individual Heterogeneity and
* Discriminatory Accuracy (MAIHDA) to evaluate hospital performance: The case of
* Hospital Differences in Patient Survival after Acute Myocardial Infarction
**********************************************************************************

* Set the working directory
cd "Y:\manuscripts-4-under-review\rodriguez-lopez2020bmiopen\2020-07-16"
// Edit the above folder directory to where the dataset is saved

* Set MLwiN path
global MLwiN_path "C:\Program Files\MLwiN v3.05\mlwin.exe"

* Set the output format of coefficient tables to two decimal places
set cformat %9.2f




**********************************************************************************
* Model 1
**********************************************************************************

* Load the data
use "Supplemental Material 1_AMIdatabase.dta", clear

* Fit model 1 by MQL1 to get parameter starting values
runmlwin proportion cons, ///
  level4(hospital: cons) ///
  level3(rscategory:) ///
```

For peer review only

```
  level2(stratum:) ///
  level1(stratum:) ///
  discrete(distribution(binomial) link(logit) denominator(denom) mql1) ///
  nopause

* Fit model 1 by MCMC to get final parameter estimates
runmlwin proportion cons, ///
  level4(hospital: cons, residuals(v, savechains("m1v.dta", replace))) ///
  level3(rscategory:) ///
  level2(stratum:) ///
  level1(stratum:) ///
  discrete(distribution(binomial) link(logit) denominator(denom)) ///
  mcmc(burnin(5000) chain(10000) thinning(10)  savechains("m1b.dta", replace)) ///
  initsprevious ///
  nopause

* Model predictions based on means of MCMC chains
rename v0* m1v*
predict m1xb, xb
generate m1xbv = m1xb + m1v
generate m1pxbv = invlogit(m1xbv)

* Compress and save the data
compress
save "m1.dta", replace

* VPC based on full MCMC chains
use "m1b.dta", clear
rename RP4_var_cons_ sigma2v
```

For peer review only

```
generate m1vpc_v = 100*sigma2v/(sigma2v + _pi^2/3)

mcmcsum m1vpc_v, variable


* Model predictions based on full MCMC chains

use "m1b.dta", clear

isid iteration

keep iteration FP1_cons

rename FP1_cons beta0

merge 1:m iteration using "m1v.dta", assert(match) keep(match) keepusing(hospital value)
nogenerate

rename value v

generate m1pxbv = 100*invlogit(beta + v)

gcollapse (mean) m1pxbvmn = m1pxbv (p2.5) m1pxbvlo = m1pxbv (p97.5) m1pxbvhi = m1pxbv,
by(hospital)

compress

save "m1pxbv", replace


* Residuals H - Histogram of the predicted hospital random effects based on means of MCMC
chains

use "m1.dta", clear

egen tag_hospital = tag(hospital)

histogram m1v if tag_hospital==1, width(0.1) start(-0.35) frequency addlabel ///

  normal ///

  kdensity kdenopts(lpattern(dash)) ///

  ytitle("Number of hospitals") ///

  ylabel(, angle(horizontal)) ///

  xtitle("Predicted hospital random effect") ///

  legend(on order(3 "Normal curve" 2 "Smoothed histogram") position(2) ring(0)) ///

  scheme(s1mono) ///

  xsize(7.25) ysize(4)
```

For peer review only

```
graph export "Figure S1 - Model 1 - Histogram of the predicted hospital random effects.png",
replace width(1000)


* ROC AUC based on means of MCMC chains
use "m1.dta", clear
keep hospital rscategory stratum numerator denominator m1xbv
expand denominator
sort stratum
generate patient = _n
order patient, after(stratum)
bysort stratum (patient): generate y = (numerator>=_n)
generate m1pxbv = invlogit(m1xbv)
roctab y m1pxbv
compress
save "m1roc.dta", replace




******************************************************************************
* Model 2
******************************************************************************


* Load the data
use "Supplemental Material 1_AMIdatabase.dta", clear


* Fit model 2 by MQL1 to get parameter starting values
runmlwin prop cons, ///
  level4(hospital: cons) ///
  level3(rscategory: cons) ///
```

For peer review only

```
  level2(stratum:) ///
  level1(stratum:) ///
  discrete(distribution(binomial) link(logit) denominator(denom)) ///
  nopause

* Fit model 2 by MCMC to get final parameter estimates
runmlwin prop cons, ///
  level4(hospital: cons, residuals(v, savechains("m2v.dta", replace))) ///
  level3(rscategory: cons, residuals(u, savechains("m2u.dta", replace))) ///
  level2(stratum:) ///
  level1(stratum:) ///
  discrete(distribution(binomial) link(logit) denominator(denom)) ///
  mcmc(cc burnin(5000) chain(10000) thinning(10) hc(3) savechains("m2b.dta", replace)) ///
  initsprevious nopause
rename v0* m2v*
rename u0* m2u*

* Model predictions based on means of MCMC chains
predict m2xb, xb
generate m2xbv = m2xb + m2v
generate m2xbu = m2xb + m2u
generate m2xbvu = m2xb + m2v + m2u
generate m2pxbv = invlogit(m2xbv)
generate m2pxbu = invlogit(m2xbu)
generate m2pxbvu = invlogit(m2xbvu)
generate m2pxbvlo = invlogit(m2xbv - 1.96*m2vse)
generate m2pxbvhi = invlogit(m2xbv + 1.96*m2vse)

* Compress and save the data
```

For peer review only

```
compress

save "m2.dta", replace


* Model predictions based on full MCMC chains

use "m2b.dta", clear

isid iteration

keep iteration FP1_cons

rename FP1_cons beta0

merge 1:m iteration using "m2v.dta", assert(match) keep(match) keepusing(hospital value)
nogenerate

rename value v

generate m2pxbv = 100*invlogit(beta + v)

gcollapse (mean) m2pxbvmn = m2pxbv (p2.5) m2pxbvlo = m2pxbv (p97.5) m2pxbvhi = m2pxbv,
by(hospital)

compress

save "m2pxbv", replace


* VPC based on full MCMC chains

use "m2b.dta", clear

rename RP4_var_cons_ sigma2v

rename RP3_var_cons_ sigma2u

generate m1vpc_v = 100*sigma2v/(sigma2v + sigma2u + _pi^2/3)

generate m1vpc_u = 100*sigma2u/(sigma2v + sigma2u + _pi^2/3)

mcmcsum m1vpc_v m1vpc_u, variable


* Residuals H - Histogram of the predicted hospital random effects based on means of MCMC
chains

use "m2.dta", clear

egen tag_hospital = tag(hospital)

histogram m2v if tag_hospital==1, width(0.1) start(-0.40) frequency addlabel ///
```

For peer review only

```
  normal ///
  kdensity kdenopts(lpattern(dash)) ///
  ytitle("Number of hospitals") ///
  ylabel(, angle(horizontal)) ///
  xtitle("Predicted hospital random effect") ///
  legend(on order(3 "Normal curve" 2 "Smoothed histogram") position(2) ring(0)) ///
  scheme(s1mono) ///
  xsize(7.25) ysize(4)
graph export "Figure S2 - Model 2 - Histogram of the predicted hospital random effects.png", replace width(1000)


* Residuals RS - Histogram of the predicted hospital random effects based on means of MCMC chains
use "m2.dta", clear
egen tag_rscategory = tag(rscategory)
histogram m2u if tag_rscategory==1, width(1) start(-3) frequency addlabel ///
  normal ///
  kdensity kdenopts(lpattern(dash)) ///
  ytitle("Number of RS categories") ///
  ylabel(, angle(horizontal)) ///
  xtitle("Predicted RS category random effect") ///
  legend(on order(3 "Normal curve" 2 "Smoothed histogram") position(2) ring(0)) ///
  scheme(s1mono) ///
  xsize(7.25) ysize(4)
graph export "Figure S3 - Model 2 - Histogram of the predicted RS category random effects.png", replace width(1000)


* Figure 2 based on full MCMC chains
use "m1.dta", replace
collapse (sum) numerator denominator, by(hospital)
```

For peer review only

```
isid hospital
generate percentage = 100*(numerator/denominator)
merge 1:m hospital using "m1pxbv.dta", assert(match) keep(match) nogenerate
merge 1:m hospital using "m2pxbv.dta", assert(match) keep(match) nogenerate
sort m2pxbvmn
generate rank = _n
twoway ///
  (scatter percentage rank, msymbol(circle) mfcolor(white) mlcolor(black)) ///
  (scatter m1pxbvmn rank, mcolor(gray) msymbol(circle)) ///
  (scatter m2pxbvmn rank, mcolor(black) msymbol(circle)) ///
  (rspike m2pxbvhi m2pxbvlo rank), ///
  ytitle("Absolute risk of 30-day mortality (%)") ///
  ylabel(, angle(horizontal)) ///
  xtitle("Hospital rank") ///
  xlabel(0(10)70) ///
  legend(off) ///
  scheme(s1mono) ///
  xsize(7.25) ysize(4)
graph export "Figure 2.png", replace width(1000)

* ROC AUC based on means of MCMC chains
use "m2.dta", clear
keep hospital rscategory stratum numerator denominator m2xbv m2xbu m2xbvu
expand denominator
sort stratum
generate patient = _n
order patient, after(stratum)
bysort stratum (patient): generate y = (numerator>=_n)
generate m2pxbv = invlogit(m2xbv)
```

For peer review only

```
generate m2pxbu = invlogit(m2xbu)

generate m2pxbvu = invlogit(m2xbvu)

roctab y m2pxbv

roctab y m2pxbu

roctab y m2pxbvu

compress

save "m2roc.dta", replace


* Figure 3 - Area under the Receiver Operating Characteristics Curve based on means of MCMC chains

use "m1roc.dta", clear

merge 1:1 patient using "m2roc.dta", assert(match) keep(match) nogenerate

roccomp y m1pxbv m2pxbv m2xbu m2xbvu, ///

 graph ///

 plot1opts(recast(line) lcolor(navy)) ///

 plot2opts(recast(line) lcolor(red)) ///

 plot3opts(recast(line) lcolor(green)) ///

 plot4opts(recast(line) lcolor(orange)) ///

 rlopts(lpattern(dash)) ///

 ytitle("True positive fraction") ///

 ylabel(, angle(horizontal)) ///

 xtitle("False positive fraction") ///

 legend(off) ///

 scheme(s1mono) ///

 xsize(7.25) ysize(4)

graph export "Figure 3 - Area under the Receiver Operating Characteristics Curve.png", replace width(1000)


*****************************************************************************

* Model 3. Traditional Random intercept model
```

For peer review only

```
*****************************************************************************

* Load the data
use "Supplemental Material 1_AMIdatabase.dta", clear

* Fit random intercept by MQL1 to get parameter starting values
tab     rscategory,     gen(risk)
runmlwin proportion cons risk1-risk10, ///
  level3(hospital: cons) ///
  level2(stratum:) ///
  level1(stratum:) ///
  discrete(distribution(binomial) link(logit) denominator(denom) mql1) ///
  nopause

* Fit random intercept by MCMC to get final parameter estimates
runmlwin proportion cons risk1-risk10, ///
  level3(hospital: cons, residuals(v, savechains("m3v.dta", replace))) ///
  level2(stratum:) ///
  level1(stratum:) ///
  discrete(distribution(binomial) link(logit) denominator(denom)) ///
  mcmc(burnin(5000) chain(10000) thinning(10) hc(3)savechains("m3b.dta", replace)) ///
  initsprevious ///
  nopause

* ROC AUC based on means of MCMC chains
predict pr
gen prh=pr+v0
```

For peer review only

```
*Expand databse

expand denom

sort stratum

by stratum,sort: gen num=_n

by stratum: gen y=0 if numer<num

replace y=1 if y==.

gen Ph=invlogit(prh)


disp _N

gen prop =denom/43247

gen weight=int(1/prop)


roctab y Ph

roctab y Ph [fw=weight]


*****************************************************************************

* Interaction Effect

*****************************************************************************


* Load the data

use "Supplemental Material 1_AMIdatabase.dta", clear


* Sort hte data

sort hospital rscategory stratum


* Fit model 3 by MQL1 to get parameter starting values

runmlwin prop cons, ///

  level4(hospital: cons) ///

  level3(rscategory: cons) ///
```

For peer review only

```
  level2(stratum: cons) ///
  level1(stratum:) ///
  discrete(distribution(binomial) link(logit) denominator(denom)) ///
  nopause

* Fit model 3 by MCMC to get final parameter estimates
runmlwin prop cons, ///
  level4(hospital: cons) ///
  level3(rscategory: cons) ///
  level2(stratum: cons) ///
  level1(stratum:) ///
  discrete(distribution(binomial) link(logit) denominator(denom)) ///
  mcmc(cc burnin(5000) chain(10000) thinning(10) hc(3)) ///
  initsprevious nopause cformat(%9.3f)

*********************************************************************************
exit
```

For peer review only

```
#------------------------------------------------------------------------------
# A Cross-Classified Multilevel Analysis of Individual Heterogeneity and
# Discriminatory Accuracy (MAIHDA) to evaluate hospital performance: The case of
# Hospital Differences in Patient Survival after Acute Myocardial Infarction
#------------------------------------------------------------------------------

# Load required packages
library(R2MLwiN)

# Set MLwiN path
options(MLwiN_path = 'C:/Program Files/MLwiN v3.05/')

# Load the dataset
mydata <- read.csv("Supplemental Material 1_AMIdatabase.csv")



#------------------------------------------------------------------------------
# Model 1
#------------------------------------------------------------------------------

# Fit Model 1
model1 <- runMLwiN(logit(proportion, denominator) ~ 1 + (1 | hospital),
              D = "Binomial",
              estoptions = list(EstM = 1,
                           mcmcMeth = list(burnin = 5000,
                                      iterations = 10000)),
              data = mydata)
model1
```

For peer review only

```
#-----------------------------------------------------------------------------

# Model 2

#-----------------------------------------------------------------------------


# Fit Model 2

model2 <- runMLwiN(logit(proportion, denominator) ~ 1 + (1 | hospital)

                  + (1 | rscategory),

                    D = "Binomial",

                    estoptions = list(EstM = 1, xc = TRUE,

                                 mcmcMeth = list(burnin  = 5000,

                                        iterations = 10000),

                                 mcmcOptions = list(hcen = 2)),

                    data = mydata)

model2


#---------------------------------------------------------------------------
```

```
hospital,cons,rscategory,stratum,proportion,numerator,denominator
1,1,1,1,0,0,99
1,1,2,2,.013157895,1,76
1,1,3,3,.02739726,2,73
1,1,4,4,.016129032,1,62
1,1,5,5,.013333334,1,75
1,1,6,6,.045977011,4,87
1,1,7,7,.074074075,8,108
1,1,8,8,.0625,7,112
1,1,9,9,.093220338,11,118
1,1,10,10,.22413793,26,116
2,1,1,11,0,0,196
2,1,2,12,.005586592,1,179
2,1,3,13,.0064935065,1,154
2,1,4,14,.029411765,4,136
2,1,5,15,.044303797,7,158
2,1,6,16,.044025157,7,159
2,1,7,17,.059210528,9,152
2,1,8,18,.14285715,27,189
2,1,9,19,.1761658,34,193
2,1,10,20,.21538462,42,195
3,1,1,21,.012048192,3,249
3,1,2,22,.021164021,4,189
3,1,3,23,.017441861,3,172
3,1,4,24,.03763441,7,186
3,1,5,25,.067114092,10,149
3,1,6,26,.066265061,11,166
3,1,7,27,.093023255,12,129
3,1,8,28,.15625,20,128
3,1,9,29,.22340426,21,94
3,1,10,30,.30526316,29,95
4,1,1,31,.01010101,1,99
4,1,2,32,.014492754,1,69
4,1,3,33,.02631579,2,76
4,1,4,34,.041666668,3,72
4,1,5,35,.051282052,4,78
4,1,6,36,.10227273,9,88
4,1,7,37,.076086953,7,92
4,1,8,38,.11627907,10,86
4,1,9,39,.20879121,19,91
4,1,10,40,.20491803,25,122
5,1,1,41,0,0,127
5,1,2,42,0,0,97
5,1,3,43,.017699115,2,113
5,1,4,44,0,0,100
5,1,5,45,0,0,93
5,1,6,46,.049019609,5,102
```

For peer review only

5,1,7,47,.054545455,6,110
5,1,8,48,.062068965,9,145
5,1,9,49,.071428575,10,140
5,1,10,50,.23809524,35,147
6,1,1,51,0,0,26
6,1,2,52,0,0,22
6,1,3,53,.033333335,1,30
6,1,4,54,.054054055,2,37
6,1,5,55,.090909094,2,22
6,1,6,56,.054054055,2,37
6,1,7,57,.0625,2,32
6,1,8,58,.125,4,32
6,1,9,59,.10714286,3,28
6,1,10,60,.21621622,8,37
7,1,1,61,0,0,26
7,1,2,62,0,0,19
7,1,3,63,.055555556,1,18
7,1,4,64,0,0,23
7,1,5,65,.11538462,3,26
7,1,6,66,.074074075,2,27
7,1,7,67,0,0,26
7,1,8,68,.13513513,5,37
7,1,9,69,.27777779,10,36
7,1,10,70,.21875,7,32
8,1,1,71,0,0,157
8,1,2,72,.023952097,4,167
8,1,3,73,.0068027209,1,147
8,1,4,74,.045112781,6,133
8,1,5,75,.037037037,5,135
8,1,6,76,.073170729,9,123
8,1,7,77,.032467533,5,154
8,1,8,78,.12698413,16,126
8,1,9,79,.086330935,12,139
8,1,10,80,.2932331,39,133
9,1,1,81,0,0,27
9,1,2,82,0,0,23
9,1,3,83,0,0,14
9,1,4,84,0,0,7
9,1,5,85,.071428575,1,14
9,1,6,86,0,0,15
9,1,7,87,0,0,7
9,1,8,88,.07692308,1,13
9,1,9,89,0,0,11
9,1,10,90,.21428572,3,14
10,1,1,91,0,0,62
10,1,2,92,.032258064,3,93
10,1,3,93,.011904762,1,84

For peer review only

```
10,1,4,94,.01369863,1,73
10,1,5,95,.03846154,3,78
10,1,6,96,.06666667,6,90
10,1,7,97,.068965517,6,87
10,1,8,98,.22580644,14,62
10,1,9,99,.1594203,11,69
10,1,10,100,.33962265,18,53
11,1,1,101,.041666668,1,24
11,1,2,102,.035714287,1,28
11,1,3,103,.027027028,1,37
11,1,4,104,0,0,50
11,1,5,105,.027027028,1,37
11,1,6,106,.071428575,2,28
11,1,7,107,.078947365,3,38
11,1,8,108,.064516127,2,31
11,1,9,109,.037037037,1,27
11,1,10,110,.40000001,6,15
12,1,1,111,0,0,10
12,1,2,112,0,0,19
12,1,3,113,0,0,13
12,1,4,114,0,0,28
12,1,5,115,0,0,14
12,1,6,116,.071428575,2,28
12,1,7,117,.14285715,3,21
12,1,8,118,0,0,18
12,1,9,119,.23529412,4,17
12,1,10,120,.14285715,2,14
13,1,1,121,0,0,135
13,1,2,122,.00729927,1,137
13,1,3,123,.035460994,5,141
13,1,4,124,.015037594,2,133
13,1,5,125,.014705882,2,136
13,1,6,126,.037313432,5,134
13,1,7,127,.096000001,12,125
13,1,8,128,.08510638,8,94
13,1,9,129,.13861386,14,101
13,1,10,130,.23863636,21,88
14,1,1,131,.022222223,1,45
14,1,2,132,0,0,44
14,1,3,133,0,0,42
14,1,4,134,.029411765,2,68
14,1,5,135,.044117648,3,68
14,1,6,136,.057692308,3,52
14,1,7,137,.068965517,4,58
14,1,8,138,.16129032,10,62
14,1,9,139,.19672132,12,61
14,1,10,140,.30000001,24,80
```

For peer review only

15,1,1,141,.03125,1,32
15,1,2,142,0,0,17
15,1,3,143,0,0,35
15,1,4,144,.079999998,2,25
15,1,5,145,.10714286,3,28
15,1,6,146,.052631579,1,19
15,1,7,147,.039999999,1,25
15,1,8,148,.17391305,4,23
15,1,9,149,.22727273,5,22
15,1,10,150,.16666667,4,24
16,1,1,151,0,0,74
16,1,2,152,.010638298,1,94
16,1,3,153,0,0,85
16,1,4,154,0,0,100
16,1,5,155,.032967035,3,91
16,1,6,156,.12195122,10,82
16,1,7,157,.064516127,6,93
16,1,8,158,.10465116,9,86
16,1,9,159,.15625,10,64
16,1,10,160,.2,16,80
17,1,1,161,0,0,18
17,1,2,162,.028571429,1,35
17,1,3,163,0,0,28
17,1,4,164,.05882353,2,34
17,1,5,165,.073170729,3,41
17,1,6,166,.10526316,4,38
17,1,7,167,.051282052,2,39
17,1,8,168,.093023255,4,43
17,1,9,169,.13793103,4,29
17,1,10,170,.36363637,12,33
18,1,1,171,.074074075,2,27
18,1,2,172,.043478262,1,23
18,1,3,173,0,0,24
18,1,4,174,0,0,26
18,1,5,175,0,0,30
18,1,6,176,.07692308,2,26
18,1,7,177,.074074075,2,27
18,1,8,178,.05882353,2,34
18,1,9,179,.07692308,3,39
18,1,10,180,.075000003,3,40
19,1,1,181,0,0,34
19,1,2,182,0,0,40
19,1,3,183,0,0,38
19,1,4,184,.07692308,3,39
19,1,5,185,0,0,45
19,1,6,186,0,0,37
19,1,7,187,.075471699,4,53

For peer review only

19,1,8,188,.10714286,6,56
19,1,9,189,.15000001,9,60
19,1,10,190,.24074075,13,54
20,1,1,191,0,0,20
20,1,2,192,.055555556,1,18
20,1,3,193,0,0,13
20,1,4,194,.050000001,1,20
20,1,5,195,.050000001,1,20
20,1,6,196,.043478262,1,23
20,1,7,197,.039999999,1,25
20,1,8,198,.16,4,25
20,1,9,199,.2,4,20
20,1,10,200,.23529412,4,17
21,1,1,201,0,0,21
21,1,2,202,0,0,20
21,1,3,203,0,0,18
21,1,4,204,0,0,25
21,1,5,205,.068965517,2,29
21,1,6,206,.06666667,2,30
21,1,7,207,.13636364,3,22
21,1,8,208,.10714286,3,28
21,1,9,209,.27586207,8,29
21,1,10,210,.26923078,7,26
22,1,1,211,0,0,85
22,1,2,212,.0093457941,1,107
22,1,3,213,.034188036,4,117
22,1,4,214,.024590164,3,122
22,1,5,215,.036036037,4,111
22,1,6,216,.040816326,4,98
22,1,7,217,.13829787,13,94
22,1,8,218,.11,11,100
22,1,9,219,.14285715,13,91
22,1,10,220,.20987654,17,81
23,1,1,221,.05882353,1,17
23,1,2,222,.045454547,1,22
23,1,3,223,0,0,16
23,1,4,224,.035714287,1,28
23,1,5,225,0,0,22
23,1,6,226,.07692308,2,26
23,1,7,227,.15789473,6,38
23,1,8,228,0,0,23
23,1,9,229,.15384616,4,26
23,1,10,230,.125,3,24
24,1,1,231,0,0,27
24,1,2,232,.078947365,3,38
24,1,3,233,0,0,34
24,1,4,234,0,0,28

For peer review only

24,1,5,235,.030303031,1,33
24,1,6,236,.11627907,5,43
24,1,7,237,.054054055,2,37
24,1,8,238,.12820514,5,39
24,1,9,239,.1724138,5,29
24,1,10,240,.24137931,7,29
25,1,1,241,.0099009899,1,101
25,1,2,242,.02,2,100
25,1,3,243,.031914894,3,94
25,1,4,244,.026548672,3,113
25,1,5,245,.073394492,8,109
25,1,6,246,.088495575,10,113
25,1,7,247,.096153848,10,104
25,1,8,248,.15730338,14,89
25,1,9,249,.21176471,18,85
25,1,10,250,.19736843,15,76
26,1,1,251,0,0,41
26,1,2,252,0,0,43
26,1,3,253,0,0,48
26,1,4,254,0,0,47
26,1,5,255,.035714287,2,56
26,1,6,256,.022727273,1,44
26,1,7,257,.03773585,2,53
26,1,8,258,.087719299,5,57
26,1,9,259,.10294118,7,68
26,1,10,260,.31645569,25,79
27,1,1,261,0,0,9
27,1,2,262,0,0,17
27,1,3,263,0,0,17
27,1,4,264,.047619049,1,21
27,1,5,265,0,0,18
27,1,6,266,0,0,20
27,1,7,267,.071428575,1,14
27,1,8,268,0,0,26
27,1,9,269,.14285715,5,35
27,1,10,270,.28947368,11,38
28,1,1,271,.0625,1,16
28,1,2,272,0,0,20
28,1,3,273,0,0,17
28,1,4,274,0,0,19
28,1,5,275,0,0,33
28,1,6,276,.0625,1,16
28,1,7,277,.11764706,2,17
28,1,8,278,.086956523,2,23
28,1,9,279,.20588236,7,34
28,1,10,280,.10869565,5,46
29,1,1,281,0,0,145

For peer review only

29,1,2,282,.006289308,1,159
29,1,3,283,.015037594,2,133
29,1,4,284,.02631579,3,114
29,1,5,285,.046153847,6,130
29,1,6,286,.049295776,7,142
29,1,7,287,.089655176,13,145
29,1,8,288,.11258278,17,151
29,1,9,289,.10559006,17,161
29,1,10,290,.25882354,44,170
30,1,1,291,.0040816325,1,245
30,1,2,292,.022522522,5,222
30,1,3,293,.031390134,7,223
30,1,4,294,.02293578,5,218
30,1,5,295,.025,5,200
30,1,6,296,.070484579,16,227
30,1,7,297,.078313254,13,166
30,1,8,298,.15882353,27,170
30,1,9,299,.1641791,33,201
30,1,10,300,.26035503,44,169
31,1,1,301,0,0,30
31,1,2,302,.05882353,1,17
31,1,3,303,0,0,23
31,1,4,304,0,0,13
31,1,5,305,0,0,15
31,1,6,306,0,0,16
31,1,7,307,.083333336,1,12
31,1,8,308,.16666667,4,24
31,1,9,309,.125,2,16
31,1,10,310,.40000001,10,25
32,1,1,311,.010526316,1,95
32,1,2,312,0,0,86
32,1,3,313,.013513514,1,74
32,1,4,314,.032786883,2,61
32,1,5,315,.030769231,2,65
32,1,6,316,.075949363,6,79
32,1,7,317,.04225352,3,71
32,1,8,318,.12820514,10,78
32,1,9,319,.14444445,13,90
32,1,10,320,.31325302,26,83
33,1,1,321,0,0,39
33,1,2,322,0,0,19
33,1,3,323,0,0,38
33,1,4,324,0,0,39
33,1,5,325,.018867925,1,53
33,1,6,326,.023809524,1,42
33,1,7,327,.10869565,5,46
33,1,8,328,.087719299,5,57

For peer review only

33,1,9,329,.15714286,11,70
33,1,10,330,.23170732,19,82
34,1,1,331,.016393442,1,61
34,1,2,332,.020408163,1,49
34,1,3,333,0,0,35
34,1,4,334,.03508772,2,57
34,1,5,335,.01923077,1,52
34,1,6,336,.050000001,3,60
34,1,7,337,.093333334,7,75
34,1,8,338,.11904762,10,84
34,1,9,339,.13043478,12,92
34,1,10,340,.2820513,33,117
35,1,1,341,0,0,26
35,1,2,342,.027777778,1,36
35,1,3,343,.078947365,3,38
35,1,4,344,0,0,34
35,1,5,345,.034482758,1,29
35,1,6,346,.07692308,3,39
35,1,7,347,.095238097,4,42
35,1,8,348,.088888891,4,45
35,1,9,349,.23684211,9,38
35,1,10,350,.33333334,17,51
36,1,1,351,.0060422961,2,331
36,1,2,352,.0094936704,3,316
36,1,3,353,.018808777,6,319
36,1,4,354,.026229508,8,305
36,1,5,355,.028037382,9,321
36,1,6,356,.055555556,17,306
36,1,7,357,.084639497,27,319
36,1,8,358,.1320132,40,303
36,1,9,359,.2109375,54,256
36,1,10,360,.2605364,68,261
37,1,1,361,.03846154,1,26
37,1,2,362,0,0,30
37,1,3,363,.027777778,1,36
37,1,4,364,0,0,35
37,1,5,365,.023255814,1,43
37,1,6,366,.06666667,2,30
37,1,7,367,.051282052,2,39
37,1,8,368,.13513513,5,37
37,1,9,369,.12765957,6,47
37,1,10,370,.25,14,56
38,1,1,371,0,0,96
38,1,2,372,.022727273,2,88
38,1,3,373,.020618556,2,97
38,1,4,374,.020833334,2,96
38,1,5,375,.07079646,8,113

For peer review only

38,1,6,376,.065420561,7,107
38,1,7,377,.10084034,12,119
38,1,8,378,.16129032,20,124
38,1,9,379,.17880794,27,151
38,1,10,380,.24324325,36,148
39,1,1,381,.012820513,1,78
39,1,2,382,.022988506,2,87
39,1,3,383,.0125,1,80
39,1,4,384,.04109589,3,73
39,1,5,385,.033707865,3,89
39,1,6,386,.033707865,3,89
39,1,7,387,.125,8,64
39,1,8,388,.15306123,15,98
39,1,9,389,.15909091,14,88
39,1,10,390,.31999999,24,75
40,1,1,391,0,0,12
40,1,2,392,0,0,18
40,1,3,393,0,0,12
40,1,4,394,0,0,15
40,1,5,395,.03125,1,32
40,1,6,396,.055555556,1,18
40,1,7,397,0,0,18
40,1,8,398,.23809524,5,21
40,1,9,399,.31818181,7,22
40,1,10,400,.39285713,11,28
41,1,1,401,.010869565,1,92
41,1,2,402,.015625,2,128
41,1,3,403,.02631579,3,114
41,1,4,404,.0070921984,1,141
41,1,5,405,.056451611,7,124
41,1,6,406,.10687023,14,131
41,1,7,407,.10144927,14,138
41,1,8,408,.10738255,16,149
41,1,9,409,.15231788,23,151
41,1,10,410,.20512821,24,117
42,1,1,411,0,0,101
42,1,2,412,0,0,108
42,1,3,413,.01369863,2,146
42,1,4,414,.045801528,6,131
42,1,5,415,.069230773,9,130
42,1,6,416,.048780486,6,123
42,1,7,417,.12844037,14,109
42,1,8,418,.070422538,5,71
42,1,9,419,.14117648,12,85
42,1,10,420,.24637681,17,69
43,1,1,421,0,0,17
43,1,2,422,0,0,26

For peer review only

43,1,3,423,0,0,24
43,1,4,424,.071428575,1,14
43,1,5,425,0,0,23
43,1,6,426,0,0,17
43,1,7,427,.043478262,1,23
43,1,8,428,.30000001,6,20
43,1,9,429,.15000001,3,20
43,1,10,430,.22916667,11,48
44,1,1,431,0,0,11
44,1,2,432,0,0,18
44,1,3,433,0,0,14
44,1,4,434,0,0,7
44,1,5,435,0,0,14
44,1,6,436,0,0,17
44,1,7,437,.14285715,3,21
44,1,8,438,.22222222,4,18
44,1,9,439,.39130434,9,23
44,1,10,440,.27272728,6,22
45,1,1,441,.01459854,2,137
45,1,2,442,.016393442,3,183
45,1,3,443,.035460994,5,141
45,1,4,444,.03267974,5,153
45,1,5,445,.029629629,4,135
45,1,6,446,.056451611,7,124
45,1,7,447,.094017096,11,117
45,1,8,448,.05982906,7,117
45,1,9,449,.15789473,12,76
45,1,10,450,.28395063,23,81
46,1,1,451,0,0,16
46,1,2,452,0,0,20
46,1,3,453,.052631579,1,19
46,1,4,454,.071428575,1,14
46,1,5,455,.10714286,3,28
46,1,6,456,.11538462,3,26
46,1,7,457,.22222222,6,27
46,1,8,458,.15151516,5,33
46,1,9,459,.24137931,7,29
46,1,10,460,.42857143,18,42
47,1,1,461,0,0,8
47,1,2,462,.0625,1,16
47,1,3,463,.1,2,20
47,1,4,464,.05882353,1,17
47,1,5,465,0,0,20
47,1,6,466,.15789473,3,19
47,1,7,467,.032258064,1,31
47,1,8,468,.10526316,2,19
47,1,9,469,.29166666,7,24

For peer review only

47,1,10,470,.26666668,8,30
48,1,1,471,.028169014,2,71
48,1,2,472,0,0,71
48,1,3,473,0,0,63
48,1,4,474,.014492754,1,69
48,1,5,475,.017241379,1,58
48,1,6,476,.055555556,4,72
48,1,7,477,.083333336,6,72
48,1,8,478,.16129032,15,93
48,1,9,479,.13095239,11,84
48,1,10,480,.2238806,15,67
49,1,1,481,0,0,24
49,1,2,482,0,0,16
49,1,3,483,0,0,30
49,1,4,484,0,0,35
49,1,5,485,.023255814,1,43
49,1,6,486,0,0,43
49,1,7,487,.083333336,4,48
49,1,8,488,.11627907,5,43
49,1,9,489,.2,7,35
49,1,10,490,.17647059,6,34
50,1,1,491,0,0,129
50,1,2,492,.027777778,3,108
50,1,3,493,.014705882,2,136
50,1,4,494,.0081967209,1,122
50,1,5,495,.020408163,2,98
50,1,6,496,.05050505,5,99
50,1,7,497,.061946902,7,113
50,1,8,498,.1388889,10,72
50,1,9,499,.12244898,12,98
50,1,10,500,.30434781,21,69
51,1,1,501,0,0,21
51,1,2,502,0,0,25
51,1,3,503,0,0,35
51,1,4,504,.039999999,1,25
51,1,5,505,.055555556,2,36
51,1,6,506,.11627907,5,43
51,1,7,507,.037037037,1,27
51,1,8,508,.14285715,3,21
51,1,9,509,.17777778,8,45
51,1,10,510,.19047619,8,42
52,1,1,511,0,0,3
52,1,2,512,0,0,9
52,1,3,513,0,0,9
52,1,4,514,0,0,17
52,1,5,515,0,0,15
52,1,6,516,0,0,12

For peer review only

52,1,7,517,.22222222,2,9
52,1,8,518,.083333336,1,12
52,1,9,519,0,0,10
52,1,10,520,.18181819,2,11
53,1,1,521,0,0,7
53,1,2,522,0,0,12
53,1,3,523,0,0,18
53,1,4,524,.07692308,1,13
53,1,5,525,0,0,13
53,1,6,526,0,0,23
53,1,7,527,.083333336,1,12
53,1,8,528,.071428575,1,14
53,1,9,529,.14285715,3,21
53,1,10,530,.071428575,1,14
54,1,1,531,.030927835,3,97
54,1,2,532,.016393442,2,122
54,1,3,533,.034188036,4,117
54,1,4,534,.016806724,2,119
54,1,5,535,.028037382,3,107
54,1,6,536,.032786883,4,122
54,1,7,537,.10084034,12,119
54,1,8,538,.10891089,11,101
54,1,9,539,.18518518,15,81
54,1,10,540,.32075471,34,106
55,1,1,541,0,0,27
55,1,2,542,0,0,53
55,1,3,543,0,0,30
55,1,4,544,0,0,38
55,1,5,545,0,0,37
55,1,6,546,0,0,38
55,1,7,547,.07692308,3,39
55,1,8,548,.10344828,3,29
55,1,9,549,.12903225,4,31
55,1,10,550,.1891892,7,37
56,1,1,551,.021276595,1,47
56,1,2,552,0,0,38
56,1,3,553,.039215688,2,51
56,1,4,554,.019607844,1,51
56,1,5,555,0,0,37
56,1,6,556,.063829787,3,47
56,1,7,557,.063829787,3,47
56,1,8,558,.14285715,6,42
56,1,9,559,.18181819,8,44
56,1,10,560,.21875,7,32
57,1,1,561,.014492754,1,69
57,1,2,562,0,0,73
57,1,3,563,.011494253,1,87

For peer review only

57,1,4,564,.033333335,3,90
57,1,5,565,.045454547,4,88
57,1,6,566,.054054055,4,74
57,1,7,567,.084337346,7,83
57,1,8,568,.14473684,11,76
57,1,9,569,.17647059,12,68
57,1,10,570,.30864197,25,81
58,1,1,571,0,0,22
58,1,2,572,.043478262,1,23
58,1,3,573,0,0,42
58,1,4,574,.057142857,2,35
58,1,5,575,0,0,34
58,1,6,576,.060606062,2,33
58,1,7,577,0,0,34
58,1,8,578,.16666667,7,42
58,1,9,579,.10714286,3,28
58,1,10,580,.2,6,30
59,1,1,581,0,0,12
59,1,2,582,.037037037,1,27
59,1,3,583,.035714287,1,28
59,1,4,584,.043478262,1,23
59,1,5,585,.03846154,1,26
59,1,6,586,.13333334,2,15
59,1,7,587,.052631579,1,19
59,1,8,588,0,0,30
59,1,9,589,.22222222,4,18
59,1,10,590,.30434781,7,23
60,1,1,591,.015151516,1,66
60,1,2,592,.015873017,1,63
60,1,3,593,.04225352,3,71
60,1,4,594,0,0,80
60,1,5,595,.016129032,1,62
60,1,6,596,.050847456,3,59
60,1,7,597,.11320755,6,53
60,1,8,598,.065217391,3,46
60,1,9,599,.18181819,8,44
60,1,10,600,.1627907,7,43
61,1,1,601,.0066666668,1,150
61,1,2,602,.016666668,2,120
61,1,3,603,.033613447,4,119
61,1,4,604,.036036037,4,111
61,1,5,605,.021978023,2,91
61,1,6,606,.036144577,3,83
61,1,7,607,.11111111,10,90
61,1,8,608,.17105263,13,76
61,1,9,609,.12371134,12,97
61,1,10,610,.29487181,23,78

For peer review only

62,1,1,611,0,0,31
62,1,2,612,.039999999,1,25
62,1,3,613,0,0,31
62,1,4,614,0,0,35
62,1,5,615,.055555556,2,36
62,1,6,616,.05882353,2,34
62,1,7,617,.10714286,3,28
62,1,8,618,.081081077,3,37
62,1,9,619,.125,6,48
62,1,10,620,.2,7,35
63,1,1,621,0,0,17
63,1,2,622,0,0,18
63,1,3,623,.095238097,2,21
63,1,4,624,.043478262,1,23
63,1,5,625,.03846154,1,26
63,1,6,626,0,0,20
63,1,7,627,.047619049,1,21
63,1,8,628,.17647059,3,17
63,1,9,629,.11111111,2,18
63,1,10,630,.17647059,3,17
64,1,1,631,0,0,31
64,1,2,632,0,0,25
64,1,3,633,.125,4,32
64,1,4,634,.15000001,3,20
64,1,5,635,.19354838,6,31
64,1,6,636,.074074075,2,27
64,1,7,637,.16,4,25
64,1,8,638,.26086956,6,23
64,1,9,639,.34615386,9,26
64,1,10,640,.46666667,7,15
65,1,1,641,0,0,32
65,1,2,642,0,0,22
65,1,3,643,0,0,24
65,1,4,644,0,0,28
65,1,5,645,0,0,32
65,1,6,646,.045454547,2,44
65,1,7,647,0,0,29
65,1,8,648,.12,3,25
65,1,9,649,.24137931,7,29
65,1,10,650,.13636364,3,22
66,1,1,651,0,0,18
66,1,2,652,0,0,30
66,1,3,653,0,0,16
66,1,4,654,.095238097,2,21
66,1,5,655,0,0,28
66,1,6,656,.050000001,1,20
66,1,7,657,.039999999,1,25

For peer review only

66,1,8,658,.16129032,5,31
66,1,9,659,.24137931,7,29
66,1,10,660,.32258064,10,31
67,1,1,661,0,0,17
67,1,2,662,0,0,12
67,1,3,663,.047619049,1,21
67,1,4,664,0,0,16
67,1,5,665,0,0,11
67,1,6,666,.16666667,2,12
67,1,7,667,0,0,15
67,1,8,668,.10526316,2,19
67,1,9,669,.21052632,4,19
67,1,10,670,.38095239,8,21
68,1,1,671,0,0,70
68,1,2,672,0,0,48
68,1,3,673,0,0,57
68,1,4,674,.032786883,2,61
68,1,5,675,.039215688,2,51
68,1,6,676,.06666667,3,45
68,1,7,677,.12727273,7,55
68,1,8,678,.13636364,9,66
68,1,9,679,.19642857,11,56
68,1,10,680,.31481481,17,54

For peer review only

STROBE 2007 (v4) Statement—Checklist of items that should be included in reports of *cross-sectional studies*

Section/Topic	Item #	Recommendation	Reported on page #
**Title and abstract**	1	(*a*) Indicate the study's design with a commonly used term in the title or the abstract	2
		(*b*) Provide in the abstract an informative and balanced summary of what was done and what was found	2
**Introduction**			
Background/rationale	2	Explain the scientific background and rationale for the investigation being reported	3-6
Objectives	3	State specific objectives, including any prespecified hypotheses	6
**Methods**			
Study design	4	Present key elements of study design early in the paper	7
Setting	5	Describe the setting, locations, and relevant dates, including periods of recruitment, exposure, follow-up, and data collection	7
Participants	6	(*a*) Give the eligibility criteria, and the sources and methods of selection of participants	7
Variables	7	Clearly define all outcomes, exposures, predictors, potential confounders, and effect modifiers. Give diagnostic criteria, if applicable	8-9
Data sources/ measurement	8*	For each variable of interest, give sources of data and details of methods of assessment (measurement). Describe comparability of assessment methods if there is more than one group	8-9
Bias	9	Describe any efforts to address potential sources of bias	NA
Study size	10	Explain how the study size was arrived at	7
Quantitative variables	11	Explain how quantitative variables were handled in the analyses. If applicable, describe which groupings were chosen and why	9
Statistical methods	12	(*a*) Describe all statistical methods, including those used to control for confounding	8-17
		(*b*) Describe any methods used to examine subgroups and interactions	8
		(*c*) Explain how missing data were addressed	NA
		(*d*) If applicable, describe analytical methods taking account of sampling strategy	NA
		(*e*) Describe any sensitivity analyses	12-13
**Results**			

For peer review only

Participants	13*	(a) Report numbers of individuals at each stage of study—eg numbers potentially eligible, examined for eligibility, confirmed eligible, included in the study, completing follow-up, and analysed	Figure 1
		(b) Give reasons for non-participation at each stage	NA
		(c) Consider use of a flow diagram	Figure 1
Descriptive data	14*	(a) Give characteristics of study participants (eg demographic, clinical, social) and information on exposures and potential confounders	17, Table 2
		(b) Indicate number of participants with missing data for each variable of interest	NA
Outcome data	15*	Report numbers of outcome events or summary measures	17
Main results	16	(*a*) Give unadjusted estimates and, if applicable, confounder-adjusted estimates and their precision (eg, 95% confidence interval). Make clear which confounders were adjusted for and why they were included	18, Table 3
		(*b*) Report category boundaries when continuous variables were categorized	Table 2
		(*c*) If relevant, consider translating estimates of relative risk into absolute risk for a meaningful time period	NA
Other analyses	17	Report other analyses done—eg analyses of subgroups and interactions, and sensitivity analyses	18
**Discussion**			
Key results	18	Summarise key results with reference to study objectives	19
Limitations	19	Discuss limitations of the study, taking into account sources of potential bias or imprecision. Discuss both direction and magnitude of any potential bias	14, 21
Interpretation	20	Give a cautious overall interpretation of results considering objectives, limitations, multiplicity of analyses, results from similar studies, and other relevant evidence	20-21
Generalisability	21	Discuss the generalisability (external validity) of the study results	21
**Other information**			
Funding	22	Give the source of funding and the role of the funders for the present study and, if applicable, for the original study on which the present article is based	22

*Give information separately for cases and controls in case-control studies and, if applicable, for exposed and unexposed groups in cohort and cross-sectional studies.

Note: An Explanation and Elaboration article discusses each checklist item and gives methodological background and published examples of transparent reporting. The STROBE checklist is best used in conjunction with this article (freely available on the Web sites of PLoS Medicine at http://www.plosmedicine.org/, Annals of Internal Medicine at http://www.annals.org/, and Epidemiology at http://www.epidem.com/). Information on the STROBE Initiative is available at www.strobe-statement.org.

For peer review only
